# The effect of exercise training in people with pre-dialysis chronic kidney disease: a systematic review with meta-analysis

**DOI:** 10.1007/s40620-024-02081-9

**Published:** 2024-10-17

**Authors:** Annette Traise, Gudrun Dieberg, Melissa Jane Pearson, Neil Andrew Smart

**Affiliations:** Clinical Exercise Physiology, School of Science and Technology, University of New England, Armidale, NSW 2351 Australia

**Keywords:** Chronic kidney disease, Pre-dialysis, Exercise, Aerobic

## Abstract

**Background:**

Chronic kidney disease (CKD) is a global health issue with high mortality and economic costs. Exercise has potential benefits for pre-dialysis CKD management. This review examines the impact of exercise on CKD patients not on dialysis, focusing on improvement in various health parameters. Findings aim to inform the role of exercise in pre-dialysis CKD care.

**Methods:**

A systematic search of MEDLINE, EMBASE, the Cochrane Library of Controlled Trials, CINAHL, and SPORTDiscus, up to August 31, 2023, used key terms relating to pre-dialysis CKD and exercise. We pooled randomized controlled trials (RCTs) comparing exercise with usual care and conducted meta-analyses based on a random effects inverse variance model with the effect measure of mean difference.

**Results:**

Of 1162 identified studies, 37 RCTs met the inclusion criteria including 1248 participants. Significant improvements were identified for peak VO_2,_ mean difference [MD] (2.66 mL/kg/min; 95% confidence interval [CI] 1.48, 3.83; *p* < 0.00001); the 6-min walk (MD 58.83 m; 95% CI 35.26, 82.41; *p* < 0.00001), timed up and go (standardised mean difference − 0.35; 95% CI − 0.54, − 0.15; *p* = 0.0006), 2-min step (MD 57.48 steps; 95% CI 27.80, 87.16; *p* = 0.0001), and sit to stand tests (MD 4.55 repetitions; 95% CI 1.49, 7.60; *p* = 0.004); short form [SF]-36 general health (MD 4.26; 95% CI 0.04, 8.47; *p* = 0.05); SF-36 mental component summary (MD 1.84; 95% CI 0.18, 3.51; *p* = 0.03); estimated glomerular filtration rate (MD 2.19 mL/min/1.73 m^2^; 95% CI 0.97, 3.50; *p* = 0.001); serum cystatin-C (MD − 0.06 mg/L; 95% CI − 0.11, − 0.02; *p* = 0.004); resting heart rate (MD − 1.97 bpm; 95% CI − 3.84, − 0.11; *p* = 0.04); triglycerides (MD − 12.97mg/dL; 95% CI − 17.30, − 8.63; *p* < 0.00001); glycosylated haemoglobin (MD − 0.25%; 95% CI − 0.50, − 0.01; *p* = 0.04); waist circumference (MD − 3.12 cm; 95% CI − 4.37, − 1.86; *p* < 0.00001); and interleukin-6 (MD − 2.24 pg/mL; 95% CI − 3.87. − 0.61; *p* = 0.007).

**Conclusions:**

Analysis revealed improvements in aerobic capacity, functional ability, quality of life, estimated glomerular filtration rate, serum cystatin-C, resting heart rate, waist circumference, triglyceride, glycosylated haemoglobin, and interleukin-6 levels.

**Graphical abstract:**

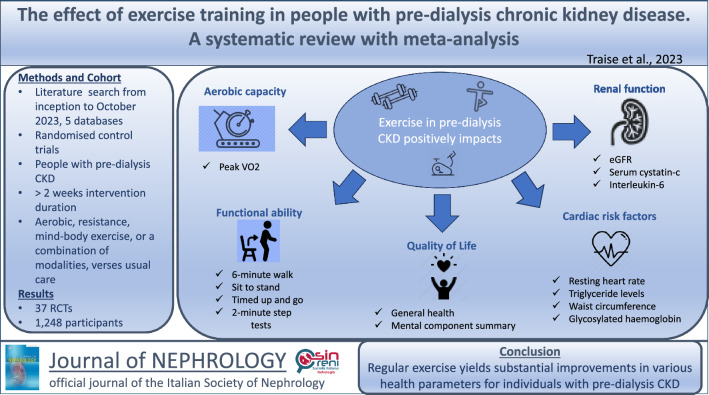

**Supplementary Information:**

The online version contains supplementary material available at 10.1007/s40620-024-02081-9.

## Introduction

Chronic kidney disease (CKD) is a preventable global health issue linked to high mortality and morbidity, comorbidities (cardiovascular disease, diabetes and hypertension) [1], and rising economic costs [2]. CKD is predicted to be the 5th leading global cause of death by 2040 [3]. CKD, defined by kidney abnormalities over 3 months, is classified by grade of estimated glomerular filtration rate (eGFR), and level of albuminuria [4]. There are 6 stages of CKD based on eGFR (G1, to G5) and 3 stages based on severity of albuminuria (A1 to A3). G1 relates to an eGFR of ≥ 90 mL/min/1.73 m^2^, G2 60–89 mL/min/1.73 m^2^, G3a 45–59 mL/min/1.73 m^2^, G3b 30–44 mL/min/1.73 m^2^, G4 15–29 mL/min/1.73 m^2^ and G5 < 15 mL/min/1.73 m^2^. The albuminuria categories relate to an albumin excretion rate A1 < 30 mg/24 h, A2 30–300 mg/24 h, and A3 > 300 mg/24 h. Prognosis and risk of progression varies depending on the severity of either eGFR or albuminuria, with higher stages of each indicating disease progression [5]. Pre-dialysis CKD is defined as the period between nephrological care and the beginning of dialysis, encompassing all stages [6].

The primary objective of pre-dialysis CKD treatment is to modify risk factors, manage comorbidity and delay progression [7]. A recent health analysis highlighted that the progression from CKD stages G1-G2 to stages G3a and b, and subsequently to stages G4 and G5, results in a 1.1–1.7-fold and 1.3–4.2-fold increase in costs, respectively, with estimated treatment costs ranging from US$20,000 to over US$100,000 per patient with kidney failure [2]. Interventions targeting modifiable risk factors during the pre-dialysis stages of CKD have the potential to delay or even prevent disease progression, offering considerable health economic benefits [7, 8].

Exercise, encompassing activities such as aerobic training, resistance training, mind–body activities like Tai Chi, or a combination of these modalities [9], has demonstrated its effectiveness as a treatment for CKD [10–12]. Integrating exercise as a primary preventative measure for individuals with pre-dialysis CKD can potentially alter the disease trajectory significantly [7, 11].

While preceding systematic reviews and meta-analyses [9, 12–17] have examined the effects of exercise on various health outcomes in individuals with pre-dialysis CKD, conflicting findings have often arisen, primarily due to discrepancies in inclusion criteria, analytical methods, and the array of assessed outcomes [9]. Recent publications [18–26] have emerged, providing new data for pooled analyses. In a review by Neale [27] several of these recent publications were identified, however, it is pertinent to note that the reported information did not expressly pertain to pre-dialysis exercise intervention.

The primary objective of this review was to investigate the potential impact of exercise training on aerobic capacity, functional ability, quality of life, renal function, cardiovascular risk factors and inflammatory markers, as applied to those diagnosed with CKD between stages G1 and pre-dialysis G5. Specifically, our study focused on those individuals in the pre-dialysis stage (we acknowledge some stage G5 individuals may not yet be undertaking dialysis and others may choose conservative care rather than dialysis), with an aim to draw meaningful comparisons between an intervention group undertaking exercise and another receiving usual care.

The Standardized Outcomes in Nephrology (SONG) Initiative Network [28] is an international effort aimed at defining core outcomes and outcome measures for a wide range of CKD research, including clinical trials. The SONG-CKD group, which focuses on those diagnosed with pre-dialysis CKD, has identified 36 outcomes for research within this patient group based on priorities shared by patients, caregivers, researchers, policy makers and relevant stakeholders. Our review incorporates several of these outcomes, including a number from the top ten, including kidney function, life participation (functional capacity), blood pressure (cardiovascular risk) and aspects of quality of life [28].

This systematic review and meta-analysis, which incorporates data extracted only from randomised control trials (RCTs) investigating the comparative effects of exercise and usual care among individuals with diagnosed CKD stages G1 to pre-dialysis G5, is an updated analysis including studies published up to 2023. We believe our meta-analysis expands the scope of prior reviews by encompassing outcomes that have not been previously documented.

## Methods

### Search strategy

We conducted systematic searches of MEDLINE, EMBASE, the Cochrane Library of Controlled Trials, CINAHL, and SPORTDiscus extending up to August 31, 2023. The search criteria encompassed a combination of controlled vocabulary MeSH and free text terms related to CKD, kidney or renal disease, exercise or physical activity, fitness, and randomised controlled trials.

The search strategy was developed in accordance with the PICO framework [29], utilising the following elements: P (Population)**—**individuals with diagnosed CKD stage G1 or greater, excluding those on dialysis, allocated to the exercise intervention group within RCTs; I (Intervention)—exercise training programs spanning a minimum duration of 2 weeks; C (Comparator)—individuals with diagnosed CKD stage G1 or higher, not on dialysis, assigned to a sedentary control group in RCTs focusing on exercise training intervention; and O (Outcomes)—encompassing parameters related to aerobic capacity, functional ability, quality of life, renal function, cardiovascular risk factors and inflammatory markers. The search was limited to peer-reviewed published randomized controlled trials.

A detailed description of the search strategy is provided in Online Resource (OR) 1, Supplemental Table (ST) 1. One reviewer (AT) conducted the search and full article eligibility was assessed by two reviewers (AT and GD/MP). In the event of disagreement, a third reviewer (NAS) was consulted.

### Study selection

The following criteria were applied for study identification and selection (1) randomized controlled (or prospective) clinical trials in pre-dialysis CKD; (2) human studies; (3) studies must have included a non-exercise or usual care control group; (4) the exercise intervention period was greater than or equal to 2 weeks. Studies of dialysis patients or those including participants with no diagnosis of CKD or < 18 years of age were excluded. The protocol for this analysis was registered with PROSPERO (CRD42023443902).

### Intervention

Exercise was defined as any method or combination of methods of structured physical activity such as aerobic exercise, resistance training, combined training, and mind–body disciplines such as Tai Chi.

### Outcomes

Studies were eligible to be included in the review if they reported on one or more of the following outcome categories:

1. Aerobic capacity assessed by volume of oxygen uptake during peak exercise (peak VO_2_) and peak respiratory rate.
2. Functional ability assessed by the six-minute walk test (6MWT), the two-minute step test, the sit to stand test (number of repetitions in 30 s), the timed up and go test, and handgrip strength.
3. Quality of life (QOL) assessed using the short form 36 (SF-36) health related quality of life (HRQOL) questionnaire.
4. Renal parameters including eGFR, serum creatinine, serum albumin, serum cystatin, urine albumin-to-creatinine ratio (UACR), urine protein-to-creatinine ratio (UPCR), 24-h urine protein, and blood urea nitrogen (BUN).
5. Cardiovascular risk factors including resting systolic blood pressure (SBP) and diastolic blood pressure (DBP), ambulatory 24-h SBP and DBP, resting heart rate, pulse wave velocity, augmentation index, asymmetric dimethylarginine, lipid studies, blood parameters, and body composition parameters.
6. Inflammatory markers including interleukin 6 (IL-6) and C-reactive protein (CRP) levels.

For the purpose of meta-analyses, outcomes were pooled only when data from three or more intervention groups were available.

### Data extraction

Two reviewers (AT and GD) conducted data extraction. For each study, the following information was collected: author; year of publication; study characteristics (country, study design, sample size); participant characteristics (age, sex, CKD stage); intervention characteristics (modality and delivery, intensity, duration, frequency); and outcomes. A standardised data extraction form was employed for this purpose. In cases where additional data or clarification was necessary, we reached out to the study authors.

### Heterogeneity and publication bias

Heterogeneity among the included studies was assessed using RevMan V5.4. The *I*^2^ test was employed to evaluate consistency across studies. *I*^2^ values less than 25% indicate a low risk, between 25 and 75% a moderate risk, and values exceeding 75% a high risk of heterogeneity [30].

We considered these *I*^2^ values along with an assessment of Egger funnel plots to evaluate overall heterogeneity and to assess the risk of publication bias [31].

### Study quality

We assessed the quality of included studies using two evaluation tools. First, we employed the Cochrane Risk of Bias tool [32] which evaluates various domains as having ‘low risk of bias’, ‘some concerns’, or ‘high risk’ of bias. Studies falling in to the ‘high risk’ category were considered to be of lower quality. The assessment was conducted independently by two reviewers, AT and GD.

Additionally, both reviewers conducted a secondary assessment of study quality using TESTEX (‘Tool for the assEssment of Study qualiTy and reporting in EXercise’) [33]. TESTEX is specifically designed for evaluating exercise training in trials focusing on participants with known chronic disease states. TESTEX employs a 15-point scale to assess both study quality (maximum 5 points) and reporting (maximum 10 points). Studies with a quality score below 10 were considered to be of lower quality [33].

### Data synthesis

We conducted statistical analyses using RevMan V5.4 [34]. Individual meta-analyses were completed for continuous data by using the mean baseline follow-up change and standard deviation (SD). In cases where the mean change was not reported, we calculated it by subtracting the baseline mean from the final mean at the end of the intervention. When the change SDs were not reported, but exact p values or 95% confidence intervals (CIs) were provided for within group changes, we used RevMan to calculate the change SDs. If exact p values or 95% CIs were not available, we imputed SDs using the Cochrane formula for standard deviation SD = square root [(SD_pre-treatment_)^2^ + (SD_post-treatment_)^2^ − (2rSD_pre-treatment_ × SD_post-treatment_) [35], with a correlation coefficient of 0.5, which is considered a conservative value. When standard error of the mean (SEM) was provided instead of the SD, we converted it to SD [30]. Median and interquartile range (IQR 1st and 3rd quarter) data were converted to mean and SD following the method described by Wan [36].

A random effects inverse variance model using the mean difference (MD) as the effect measure was employed. This model allows for heterogeneity and unobserved or unmeasured factors that may influence the data such as varying age and health of the participants and intensity of the intervention within the included studies [37]. The random effects model makes less stringent assumptions about the consistency of effects and imposes less restrictions than a fixed effect model, and is preferable over the fixed model in meta-analyses, as it generally produces a more realistic estimate of the uncertainty in the overall treatment effect [37, 38].

Where outcomes were measured using differing protocols, we used the standard mean difference (SMD). Data from studies that reported outcomes in different units were converted to the same units before pooling using appropriate conversion factors. Sensitivity analysis through a leave-one-out approach was performed to identify studies with a larger impact on results.

Sub-analyses were conducted for exercise modality, intervention duration, intervention frequency, the level of supervision, and CKD stage where applicable. Exercise modalities analysed included aerobic training, resistance training, combined aerobic and resistance training, and mind–body exercise training, (e.g., Tai Chi). Intervention durations analysed were categorised into sub-groups of less than or equal to 12 weeks, between 12 weeks and six months, and greater than six months. Levels of supervision were categorised into sub-groups of supervised, (with each session supervised throughout the trial), unsupervised (no session supervision throughout the trial), initially supervised (with a set number of supervised sessions followed by unsupervised sessions), or a combination of supervised and unsupervised sessions each week throughout the trial. We categorised pre-dialysis CKD stage sub-groups as follows: CKD 1–5 = studies reporting on stages G1 to pre-dialysis G5; CKD 2, 1–3 = studies reporting on stage G2 alone and those encompassing stages G1 to G3a and b; CKD 2–4 = studies reporting on stages G2 to G4; CKD 3–4 = studies reporting on stages G3a and b to G4; and CKD 3, 4, 3–5 = studies reporting on stages G3a and b, G4, and those encompassing stages G3a and b to pre-dialysis G5.

We considered statistical significance at the 5% level and reported pooled mean results with 95% CIs. In studies with multiple intervention groups and a control group, each intervention group was considered separately, and the control group’s sample size was divided by the number of intervention groups to prevent sample size inflation. When multiple time points during the intervention were reported, we only extracted data comparing baseline and the end of the intervention. In cases where two publications referred to the same study population outcomes, we used the publication with the largest participant count for that specific outcome.

## Results

**Fig. 1:**
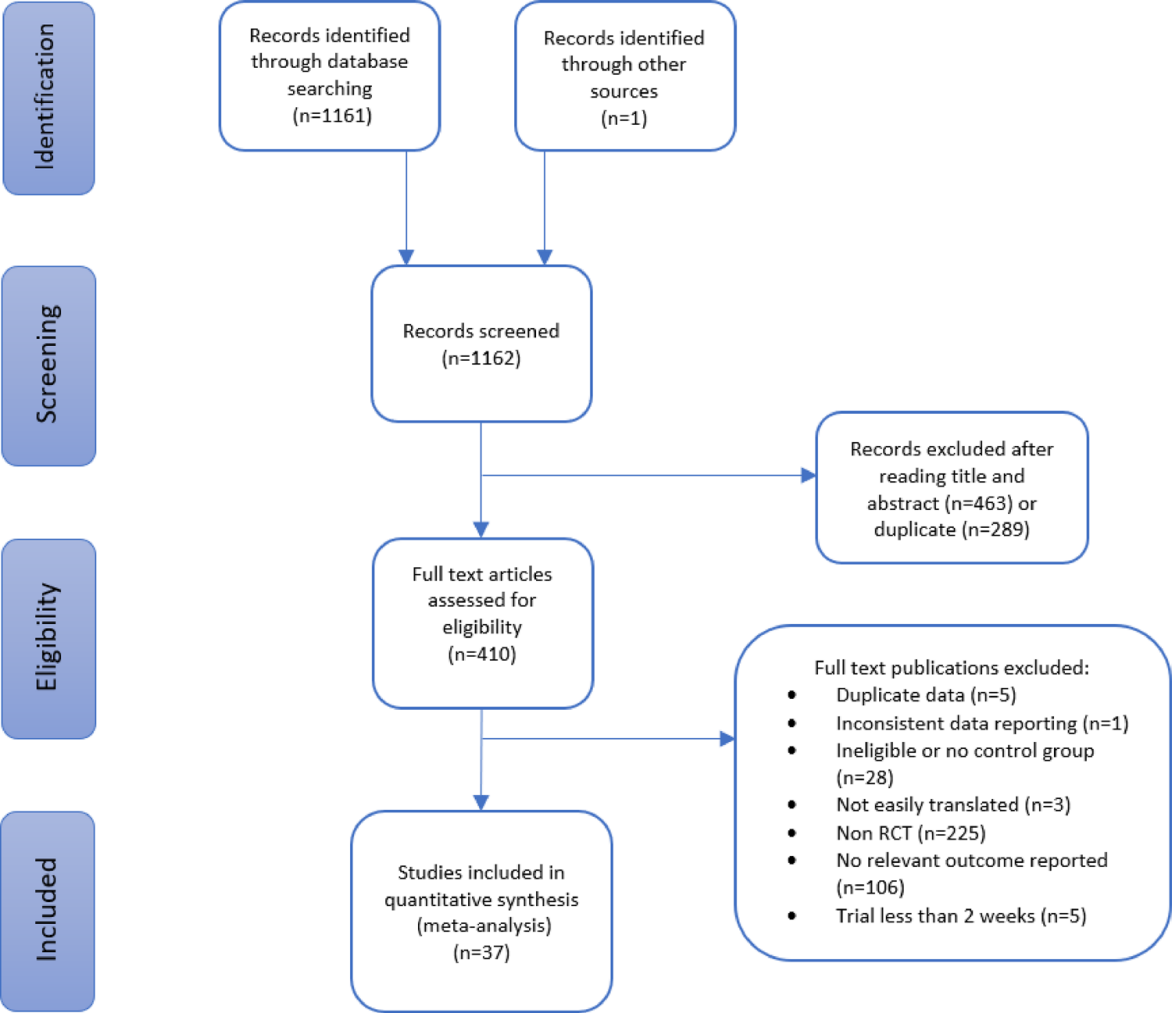
PRISMA flow diagram of the study selection

We retrieved 1162 published articles using the defined search criteria and nominated databases. One additional study was identified from reference lists. After removal of duplicates, eligibility screening excluded articles based on title and/or abstract. The remaining 410 articles were reviewed based on eligibility criteria of which 37 were included for analysis. The Preferred Reporting Items for Systematic Reviews and Meta-Analyses (PRISMA) flow diagram (Fig. 1) details the selection process. Details of excluded studies are supplied in Online Resource 1, Supplemental Table 2.

### Characteristics

Table 1 provides detailed characteristics of the 26 RCTs comprising 37 published articles, with 1248 participants in total (635 assigned to exercise groups and 613 to control groups). The mean age of the participants was 61 yrs ranging from 44.5 to 78.3 years, with 68.6% being male. Thirteen of these studies were conducted in the United States of America, eight in Brazil, four in Australia, three each in Japan and the Republic of China, two each in Canada and the United Kingdom, and one each in Belgium and Denmark. Most of the studies included a single exercise intervention group and a control group. However, two studies [36, 37] included both home and centre-based exercise groups in addition to the control group, resulting in 39 intervention groups.

**Table 1:** Characteristics of included studies

Author, date. country	Date of trial	Study population	No analysed exercise (left) control (right)		% Male	Mean age (years)	Training modality, frequency, duration, intensity	Data collection (weeks)	Drop out ex/C	Data extracted
Aoike, 2015 [58] Brazil	NS	CDK 3–4	29	14	65	55.1	AT, 3/week, 12 weeks, mild to moderate intensity	0, 12	0/0	ALB, BF, RHR
Aoike, 2018^*1^ [39] Centre based Brazil	NS	CDK 3–4	13	8	67.5	55.8	AT, 3/week, 24 weeks, moderate intensity	0, 12, 24	3/ 0	BMI, DBP, eGFR, HbA1c, peak VO_2_, SBP, STST, TUG, 2MST, 6MWT, 24HrUP
Aoike, 2018^*1^ [39] Home based Brazil	NS	CDK 3–4	12	7	67.5	55.8	AT, 3/week, 24 weeks, moderate intensity	0, 12, 24	2/0	BMI, DBP, eGFR, HbA1c, SBP, peak VO_2_, STST, TUG, 2MST, 6MWT, 24HrUP
Barcellos, 2018 [47] Brazil	NS	CDK 2–4	58	51	33	65	CT, 3/week, 16 weeks, moderate intensity	0, 8, 16	24/23	BS, BW, CRP, DBP, eGFR, HDL-C, LDL-C, SBP, STST, TC, TUG, 2MST
Baria, 2014^*1^ [67] Centre based Brazil	NS	CDK 3–4	10	5	100	52.1	AT, 3/week, 12 weeks, moderate intensity	0, 12	0/0	Hb, LBM, WC
Baria, 2014 ^*1^ [67] Home based Brazil	NS	CDK 3–4	8	4	100	52.1	AT, 3/week, 12 weeks, moderate intensity	0, 12	1/1	Hb, LBM, WC,
Beetham, 2018 [49] Australia Landmark Trial 111	2008–2011	CDK 3–4	74	68	42.5	62	CT, 2–3/week, 52 weeks, moderate intensity	0, 26, 52	8/13	BMI, BW, sCys-C, HF, peak VO_2_, sCr, TUG, UACR, UPCR, 6MWT,
Castaneda, 2004 [64] USA	NS	CDK 3–4	14	12	65.4	65	RT, 3/week, 12 weeks, moderate intensity	0, 12	0/0	ALB, BMI, BW
Chen, 2010 [66] ROC	NS	CDK 3–4	45	49	78.7	73.2	AT, 3–5/week, 12 weeks, moderate intensity	0, 12	5/1	Hb, TC
Correa, 2021^*2^ [26] Brazil	NS	CKD 2	30	30	61.6	57.5	RT, 3/week, 24 weeks, moderate intensity	0, 24	0/0	DBP, HF, RHR, SBP
Deus, 2022 [19] Brazil	NS	CKD 2	35	35	67.1	57.8	RT, 3/week, 24 weeks, moderate intensity	0, 24	0/0	ALB, BF, BMI, BS, BW, eGFR, HbA1c, HDL-C, LDL-C, TC, TG
de Araujo, 2015 [18] Brazil	NS	CKD 2	16	15	61.3	58	RT, 3–4/week, 22 weeks, moderate intensity	0, 22	3/0	ALB, DBP, eGFR, HbA1c, HDL-C, HF, IL-6, LBM, LDL-C, sCr, sCys-C, SBP, TC, TG, TUG, 6MWT
Eidemak, 1997 [40] Denmark	1991—1994	CDK 3–4	12	11	60	44.5	AT, daily, 78 weeks, moderate intensity	0, 78	3/4	HDL-C, LDL-C, peak VO_2_, TC, TG
Gomes, 2017^*1^ [59] Brazil	NS	CDK 3–4	24	15	68.75	55.5	AT, 3/week, 24 weeks, moderate intensity	0, 24	6/0	BW, CRP, HF, IL-6, sCr
Greenwood, 2015 [41] UK	NS	CKD 3–4	8	10	83.3	53.5	CT, 3/week, 52 weeks, moderate intensity	0, 26, 52	2/0	BMI, BW, CRP, sCys-C, DBP, eGFR, HDL-C, LDL-C, peak VO_2_, PWV, RHR, SBP, sCr, TC, WC
Gregory, 2011*^3^ [63] USA	NS	CKD 2–4	10	10	NS	55	CT, 3/week, 48 weeks, moderate intensity	0, 24, 48	4/0	HbA1c, sCr
Headley, 2012 [42] USA	NS	CKD 2–4	10	11	NS	55	CT, 3/week, 48 weeks, moderate intensity	0, 24, 48	4/0	BMI, BW, CRP, DBP, eGFR, HDL-C, IL-6, LDL-C, peak VO_2_, SBP, TC, TG, WC, 24HrUP
Headley, 2014 [50] USA	NS	CKD 3	25	21	65.2	57.6	AT, 3/week, 16 weeks, moderate intensity	0, 16	3/2	BF, BMI, CRP, DBP, EI, peak VO_2_, PWV, SBP, WC
Headley, 2017^*4^ [50] USA	NS	CKD 3	25	21	65.2	57.6	AT, 3/week, 16 weeks, moderate intensity	0, 16	3/2	RER, RHR
Hiraki, 2017 [43] Japan	2011–2014	CKD 3–4	14	14	100	68.7	CT, 7/week, 52 weeks, moderate intensity	0, 52	4/4	eGFR, HF, UPCR
Howden, 2013^*5^ [56] Australia Landmark Trial 111	2008–2011	CKD 3–4	36	36	62.5	61.1	CT, 2–3/week, 52 weeks, moderate intensity	0, 52	5/6	AIx, ALB, eGFR, HbA1c, HDL-C, LDL-C, PWV, RER, TC, TG
Howden, 2015^*5^ [68] Australia Landmark Trial 111	2008–2011	CKD 3–4	32	35	62.5	61.1	CT, 2–3/week, 52 weeks, moderate intensity	0, 26, 52	9/7	CRP, Hb
Huppertz, 2020^*5^ [65] Australia Landmark Trial 111	2008–2011	CKD 3–4	56	57	48	60.4	CT, 2–3/week, 52 weeks, moderate intensity	0, 52	8/13	DBP, RHR, SBP
Ikizler, 2018 [48] USA	2010–2014	CKD 3–4	22	21	58	60	AT, 3/week, 12 weeks, moderate to hard intensity	0, 16	5/5	ALB, BG, BF, BMI, BUN, DBP, eGFR, HbA1c Il-6, peak VO_2_, SBP
Kirkman, 2019 [45] USA	2013–2017	CKD 3–5	15	16	71	58	AT, 3/week, 12 weeks, moderate intensity	0, 12	4/1	AIx, DBP, eGFR, peak VO_2_, PWV, RHR, SBP
Kirkman, 2021^*6^ [20] USA	2013–2017	CKD 3–5	12	14	76.9	59	AT, 3/week, 12 weeks, moderate intensity	0, 12	7/3	HF, RER, STS
Leehey, 2009 [54] USA	NS	CKD 2–4	7	4	100	66	AT, 3/week, 24 weeks, moderate intensity	0, 6, 24	0/2	BUN, BW, CRP, DBP, eGFR, EI, Hb, HbA1c, HDL-C, LDL-C, peak VO_2_, RHR, SBP, sCr, TC, TG, UACR, UPCR, 24HrUP
Leehey, 2016 [51] USA	NS	CKD 2–4	14	18	100	66	CT, 3/week, 52 weeks, moderate intensity	0, 12, 52	4/0	BF, BMI, CRP, eGFR, HbA1c, HDL-C, LBM, LDL-C, peak VO_2_, SBP, SF-36, TC, TG, TUG, UACR, UPCR, 6MWT
Miele, 2017^*^^4^ [62] USA	NS	CKD 3	25	21	65.2	57.6	AT, 3/week, 16 weeks, moderate intensity	0, 16	3/2	eGFR, HDL-C, LDL-C, TC, TG
Mustata, 2011 [52] Canada	NS	CKD 3–5	10	10	65	68.3	AT, 2–5/week, 52 weeks, moderate intensity	0, 52	2/0	AIx, peak VO_2_, SF-36
Nixon, 2021 [21] UK	2018—2019	CKD 3–5	12	9	55.2	77.9	CT, 3/week, 12 weeks, moderate intensity	0, 12	2/6	MCS, PCS
Otobe, 2021 [22] Japan	2019	CKD 3–4	23	21	69.85	78.3	CT, 3/week, 24 weeks, moderate intensity	0, 24	4/5	ALB, BUN, CRP, sCys-C, eGFR, Hb, HbA1c, HDL, LDL, sCr, UPCR
Rossi, 2014 [46] USA	NS	CKD 3–4	48	46	52.3	68.4	CT, 2/week, 12 weeks, moderate intensity	0, 12	11/2	SF-36, 6MWT
Shi, 2014 [44] ROC	NS	CKD 1–5	11	10	71.4	69	Tai Chi, 3–5/week, 12 weeks, moderate intensity	0, 12	0/0	BUN, DBP, eGFR, HDL-C, LDL-C, RHR, SBP, sCr, TC, TG
Tang, 2017 [57] ROC	2015 2016	CKD 1–3	42	42	60.7	45.1	AT, ≥ 3/week, 12 weeks, moderate intensity	0, 12	3/3	SF-36, 6MWT
Thompson, 2022 [23] Canada	2018–2020	CKD 3–5	19	23	63.6	69	CT, 3/week, 24 weeks, moderate intensity	0, 24	2/0	AIx, BMI, BW, CRP, DBP, eGFR, HbA1c, HDL, LDL, MCS, peak VO_2,_ PCS, RHR, SBP, sCr, TC, TG
Uchiyama, 2021 [24] Japan	2018–2020	CKD 4	23	23	72	73	CT, 3/week, 24 weeks, moderate intensity	0, 12	0/0	ALB, BMI, CRP, HbA1c, HF, IL-6, PWV, WC
Van Craenenbroeck, 2015 [53] Belgium	2012–2014	CKD 3–4	19	21	55	53.2	AT, 4/day, 12 weeks, moderate to high intensity	0, 12	6/2	AIx, BMI, BW, CRP, eGFR, HDL-C, LDL-C, peak VO_2_, PWV, RER, RHR, SF-36, TC, WC
Weiner, 2022 [25] USA	NS	CKD 3–4	30	38	75	68	AT, 3/week, 52 weeks, moderate intensity	0, 52	19/12	BMI, eGFR, HbA1c, peak VO_2_, SBP, TUG, UACR, 6MWT

^*^^1^Same study as Aoike 2015

^*^^2^Same study as Deus 2022

^*^^3^Same study as Headley 2012

^*^^4^Same study as Headley 2014

^*^^5^Same study as Beetham 2018

^*^^6^Same study as Kirkman 2019

*Landmark 111 Trial* Longitudinal Assessment of Numerous Discrete Modifications of Atherosclerotic Risk in Kidney Disease, *ALB* albumin, *Aix* Augmentation Index %, *AT* aerobic training, *BF* body fat %, *BG* blood glucose, *BMI* Body Mass Index, *BUN* blood urea nitrogen, *BW* body weight, *C* control group, *CKD* chronic kidney disease, *CRP* C-reactive protein, *CT* combined training, *DBP* diastolic blood pressure, *eGFR* estimated glomerular filtration rate, *Ex* exercise group, *Hb* haemoglobin, *HbA1c* glycosylated haemoglobin, *HDL-C* high density lipoprotein cholesterol, *HF* handgrip force, *IL-6* interleukin-6, *LBM* lean body mass, *LDL-C* low density lipoprotein cholesterol, *NS* not stated, *peak VO*_*2*_ oxygen uptake during peak exercise, *PWV* aortic pulse wave velocity, *RER* peak respiratory exchange ratio,* RHR* resting heart rate, *ROC* Republic of China, *sCr* serum creatinine, *sCyc-C* serum cystatin-C, *SBP* systolic blood pressure, *SF-36* Short Form 36 Health Questionnaire, *STST* sit to stand test, *TC* total cholesterol, *TG* triglyceride, *TUG* timed up and go test, *UACR* urine albumin to creatinine ratio, *UPCR* urine protein to creatinine ratio, *UK* United Kingdom, *USA* United States of America, *WC* waist circumference, *2MST* two-minute step test, *24HrUP* 24 hour urine protein, *6MWT* six-minute walk test

The primary reported main causes of CKD were hypertensive nephropathy, diabetic nephropathy, polycystic kidney disease and glomerulonephritis [39–43]. Within the pool of studies, 18 specifically enrolled participants in CKD stages G3a and b to G4. An additional five studies exclusively targeted participants in stages G2 to G4 of CKD. Furthermore, five studies concentrated on stages G3a and b to pre-dialysis G5, and eight studies encompassed participants across stages G3a and b, G4 or within the G3a and b to pre-dialysis G5 range. One study [44] initially omitted specific CKD stage information; however, subsequent communication with the authors clarified that only individuals with pre-dialysis were included. As a result, this study has been analysed to encompass stages G1 to pre-dialysis G5 of CKD.

### Type of intervention

For detailed intervention characteristics, please refer to Supplemental Table 3 in Online Resource 2. Among the intervention groups, aerobic training was employed in 19, combined training in 15, resistance training in four and mind–body exercise in one. The duration of interventions varied, lasting anywhere from 12 to 78 weeks. Exercise session frequency ranged from twice to seven times weekly, with the majority (25 studies) being three. Session durations ranged from 10 to 90 min.

Supervision of the exercise intervention varied across studies, with 14 intervention groups having supervised sessions, ten starting as supervised and transitioning to unsupervised, seven being entirely unsupervised, and six employing a combination of both supervised and unsupervised sessions. Exercise intensity also varied, ranging from 50 to 80% of peak VO_2_ for aerobic and up to 80% of 1 repetition maximum for resistance training. Of the studies, 22 indicated a progressive exercise program, while 11 mentioned that the exercise intervention was individualised.

### Heterogeneity and publication bias

Upon examination of the *I*^2^ values, we observed predominately low to moderate levels of heterogeneity across most outcomes. However, certain outcomes displayed higher heterogeneity, suggesting some degree of bias. These outcomes encompassed the sit to stand and two-minute step test, serum albumin and blood glucose levels, blood pressure outcomes, and body weight.

Our assessment using Egger funnel plots indicated minimal evidence of publication bias (Online Resource 3, Supplemental Figures [SF] 1 to 9). Nevertheless, we did detect some potential bias in the reporting of lipid parameters, asymmetric dimethylarginine, body fat percentage and lean body mass.

### Study quality

When applying the Cochrane Risk of Bias assessment tool, the majority of the included studies raised ‘some concern’, primarily due to the absence of intention-to-treat analysis (Online Resource 4, Supplemental Table 4). It is noteworthy that we categorised none of the studies as having low-quality using this assessment tool. These findings contrast with those of Nakamura [13]. Of the same included studies, Nakamura reported a high risk in the ‘missing outcome data’ domain for three papers [42, 45, 46], one paper in the ‘measurement of outcome data’ domain [40], and in the ‘selection of reported results’ domain for four papers [41, 43, 45, 47]. Consequently, Nakamura assigned an overall high risk for these papers, ultimately leading to the conclusion of low-quality. The authors of Nakamura [13] decisions regarding the level of risk using the Cochrane Risk of Bias tool are unknown, however, we are confident our thorough assessment is appropriate for the outcomes we have included in this review.

The median TESTEX score, which evaluates both study quality and reporting, found 12 of the 37 studies to be of low-quality with a score of less than 10 out of a possible 15. Specifically, aspects such as blinding of assessors (observed in 8 out of 37 studies), intention to treat (found in 4 of 37 studies) and monitoring activity of the control group (evident in only 3 of 37 studies studies) were observed (Online Resource 4, Supplemental Table 5). We used the TESTEX data for our sub-analyses via removing low-quality studies.

### Outcomes

A summary of all meta-analyses is provided in Table 2. Further results, including sub-analyses, are available in Online Resource 6, Supplemental Table 6.

**Table 2:** Summary of meta-analyses: aerobic capacity, functional ability, quality of life, renal parameters, cardiovascular risk factors, and inflammatory markers

Outcome	Number of studies (intervention groups)	Participants exercise/control	Result: MD/SMD (95% CI), *p*, *I*^2^
**Aerobic capacity**			
Peak VO_2_ [mL/kg/min]			
	14 (15)	276/271	MD 2.66 (1.48, 3.83), * p* < 0.00001, *I*^2^ = 59%*
Peak respiratory exchange ratio			
	4	71/77	MD 0.02 (− 0.02, 0.06), * p* = 0.44, * I*^2^ = 0%
**Functional ability**			
Six-minute walk test (6MWT) [metres]			
	7 (8)	248/241	MD 58.83 (35.26, 82.41), * p* < 0.00001, * I*^2^ = 59%*
Timed up and go (TUG) [minutes]			
	6 (7)	23/195	SMD − 0.35 (− 0.54, − 0.15), * p* = 0.0006, * I*^2^ = 0%*
Two-minute step test (2MST) [steps]			
	2 (3)	80/59	MD 57.48 (27.80, 87.16), * p* = 0.0001, * I*^2^ = 76%*
Sit to stand (STS) [repetitions]			
	3 (4)	92/73	MD 4.55 (1.49, 7.60), * p* = 0.004, * I*^2^ = 86%*
Handgrip strength [kg]			
	7	193/179	MD 2.29 (− 0.67, 5.26), * p* = 0.13, * I*^2^ = 69%
**Quality of life (QoL) short form 36**			
General health (GH)			
	5	122/117	MD 4.26 (0.04, 8.47), * p* = 0.05, * I*^2^ = 14%*
Mental component summary (MCS)			
	6	115/127	MD 1.84 (0.18, 3.51), * p* = 0.03, * I*^2^ = 0%*
Physical component summary (PCS)			
	6	115/127	MD 1.16 (− 0.70, 3.02), * p* = 0.22, * I*^2^ = 14%
**Renal parameters**			
Estimated glomerular filtration rate serum creatinine (eGFR sCr) [mL/min/1.73 m^2^]			
	17 (16)	390/380	MD 2.19 (0.87, 3.50), * p* = 0.001, * I*^2^ = 17%*
	17 (16)	390/380	SMD 0.23 (0.01, 0.44), * p* = 0.04, * I*^2^ = 48%*
Estimated glomerular filtration rate serum cystatin-C (eGFR sCys-C) [mL/min/1.73 m^2^]			
	4	129/117	MD 1.49 (− 0.56, 3.55), * p* = 0.15, * I*^2^ = 0%
Serum creatinine (sCr) [mg/dL]			
	9	186/173	MD − 0.04 (− 0.12, 0.05), * p* = 0.39, * I*^2^ = 0%
Serum albumin (ALB) [g/dL]			
	8	185/175	MD 0.05 (− 0.02, 0.12), * p* = 0.16, * I*^2^ = 83%
Serum cystatin-C (sCys-C) [mg/L]			
	4	121/1149	MD − 0.06 (− 0.11, − 0.02), * p* = 0.004, * I*^2^ = 0%*
Urine albumin-to-creatinine ratio (UACR) [g/gCr]			
	4	125/127	MD − 0.00 (− 0.01, 0.01), * p* = 0.90, * I*^2^ = 0%
Urine protein-to-creatinine ratio (UPCR) [g/gCr]			
	5	132/125	MD − 0.00 (− 0.01, 0.01), * p* = 0.92, * I*^2^ = 0%
24-h urine protein [g/24h]			
	3 (4)	42/30	MD 0.09 (− 0.55, 0.73), * p* = 0.79, * I*^2^ = 0%
Blood urea nitrogen (BUN) [mg/dL]			
	4	65/53	MD − 0.55 (− 5.20, 4.10), * p* = 0.82, * I*^2^ = 0%
**Cardiovascular risk factors**			
Resting heat rate (RHR) [beats/min]			
	10	196/197	MD − 1.97 (− 3.84, − 0.11), * p* = 0.04, * I*^2^ = 0%*
**Blood pressure**			
Systolic blood pressure (SBP) [mmHg]			
	15 (16)	341/324	MD − 2.38 (− 7.32, 2.56), * p* = 0.35, * I*^2^ = 79%
Diastolic blood pressure (DBP) [mmHg]			
	14 (15)	327/306	MD − 2.28 (− 6.76, 2.21), * p* = 0.32, * I*^2^ = 87%
Ambulatory 24-h systolic blood pressure [mmHg]			
	4	80/84	MD − 0.22 (− 10.87, 10.43), * p* = 0.97, * I*^2^ = 84%
Ambulatory 24-h diastolic blood pressure [mmHg]			
	4	80/84	MD − 0.93 (− 9.55, 7.69), * p* = 0.83, * I*^2^ = 84%
**Endothelial function**			
Pulse wave velocity (PWV) aortic [m/s]			
	6	126/127	MD 0.09 (− 0.62, 0.80), * p* = 0.80, * I*^2^ = 46%
Augmentation Index (AIx) central arterial [%]			
	5	91/94	MD 1.80 (− 1.27, 4.86), * p* = 0.25, * I*^2^ = 0%
Asymmetric dimethylarginine (AMDA) [µmol/L]			
	3	49/47	MD − 0.30 (− 0.63, 0.02), * p* = 0.07, * I*^2^ = 90%
**Lipids and blood parameters**			
Triglyceride (TG) [mg/dL]			
	10	179/179	MD − 12.97 (− 17.30, − 8.63), * p* < 0.00001, * I*^2^ = 0%*
Total cholesterol (TC) [mg/dL]			
	14	309/308	MD 5.16 (− 2.79, 13.12), * p* = 0.20, * I*^2^ = 56%
Low density lipoprotein (LDL-C) [mg/dL]			
	13	273/265	MD 6.64 (− 0.39, 13.68), * p* = 0.06, * I*^2^ = 58%
High density lipoprotein (HDL-C) [mg/dL]			
	13	275/268	MD 1.32 (− 0.32, 2.97), * p* = 0.11, * I*^2^ = 0%
Glycosylated haemoglobin (HbA1c) [%]			
	12 (13)	240/230	MD − 0.25 (− 0.50, − 0.01), * p* = 0.04, * I*^2^ = 66%*
Blood glucose (BG) [mg/dL]			
	4	162/151	MD − 6.51 (− 18.52, 5.50), * p* = 0.29, * I*^2^ = 82%
Haemoglobin (Hb) [g/dL]			
	5 (6)	129/119	MD 0.23 (− 0.03, 0.50), * p* = 0.08, * I*^2^ = 0%
**Body composition parameters**			
Waist circumference [cm]			
	5 (6)	95/89	MD − 3.12 (− 4.37, − 1.86), * p* < 0.00001, * I*^2^ = 0%*
Body weight [kg]			
	9	240/218	MD − 0.85 (− 4.18, 2.49), * p* = 0.62, * I*^2^ = 81%
Body Mass Index (BMI) [kg/m^2^]			
	13 (14)	312/302	MD − 0.61 (− 1.50, 0.29), * p* = 0.18, * I*^2^ = 70%
Body fat [%]			
	5	107/104	MD − 1.94 (− 4.17, 0.30), * p* = 0.09, * I*^2^ = 28%
Lean body mass (LBM) [kg]			
	6 (7)	118/111	MD 0.89 (− 0.03, 1.81), * p* = 0.06, * I*^2^ = 58%
**Inflammatory markers**			
Interleukin-6 (IL-6) [pg/mL]			
	5	97/82	MD − 2.24 (− 3.87, − 0.61), * p* = 0.007, * I*^2^ = 69%*
C-reactive protein (CRP) [mg/L] {hs-CRP and non hs-CRP combined			
	12	255/244	MD 0.00 (− 0.01, 0.01), * p* = 0.92, * I*^2^ = 10%
	12	255/244	SMD 0.03 (− 0.23, 0.17), * p* = 0.75, * I*^2^ = 15%

*CI* confidence interval, *I*^*2*^ percentage of variation across studies due to heterogeneity, *MD* mean difference, *SMD* standard mean difference

*Significant *p* value of 0.5 or less

#### Aerobic capacity and functional ability

**Fig. 2:**
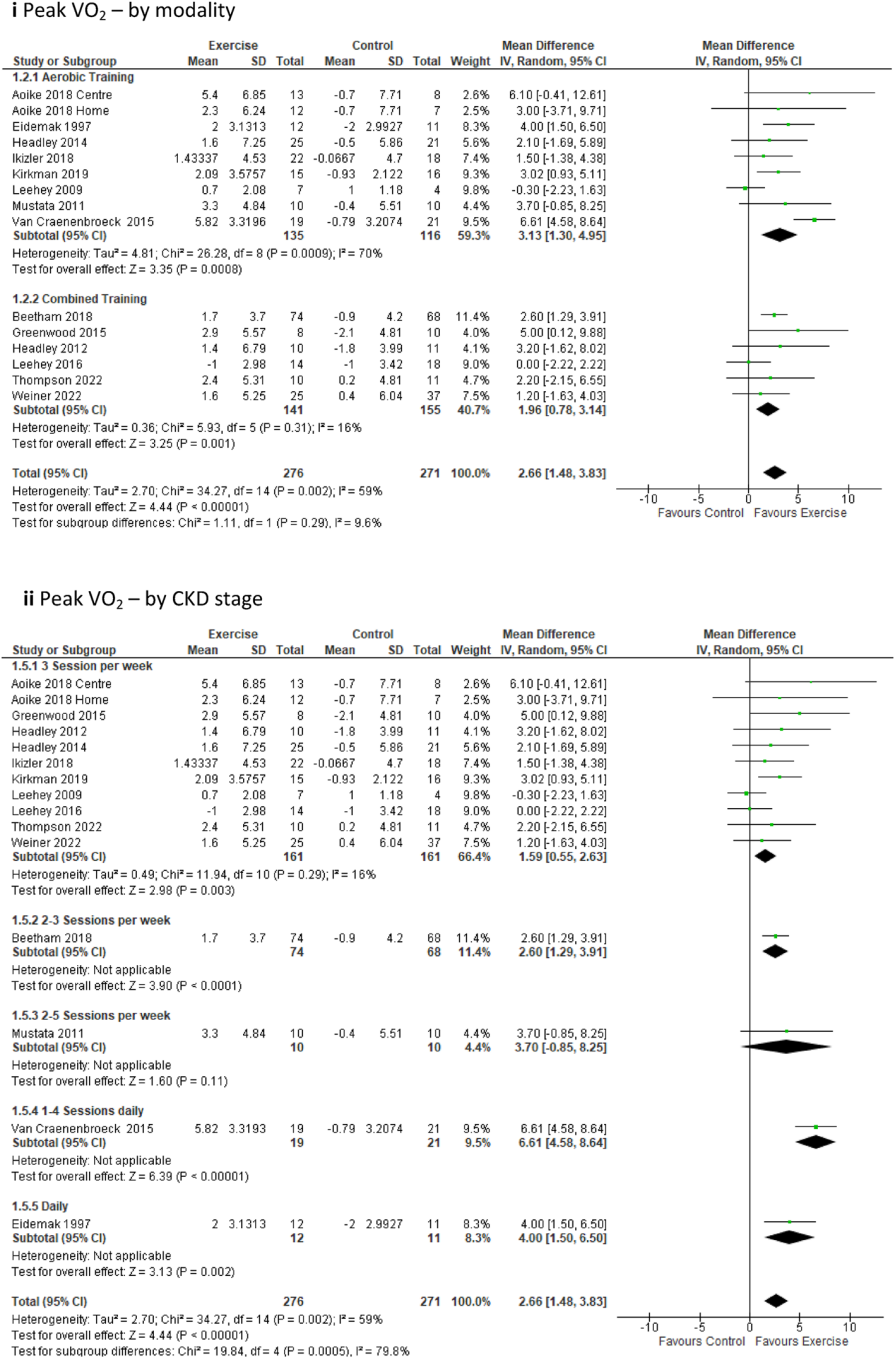
Change in aerobic capacity via Peak VO_2_ [mL/kg/min] in people with pre-dialysis CKD by modality i) and by CKD stage ii)

***Peak VO***_***2***_ data were available in 14 studies comprising 15 intervention groups and 547 participants [23, 25, 39–42, 45, 48–54]. Our pooled analysis demonstrated a significant improvement in peak VO_2_ (and hence aerobic capacity) for the exercise group when compared to the control group (MD 2.66 mL/kg/min; 95% CI 1.48 to 3.83; *p* < 0.00001) (Fig. 2). Sub-analysis removing low-quality studies from pooled analyses continued to display a significant improvement in peak VO_2_ in favour of the exercise group (Online Resource 5, Supplemental Fig. 10a).

Sub-analysis based on exercise modality demonstrated a significant improvement in peak VO_2_ for the exercise group when compared to the control group for both the aerobic and combined training interventions (OR 6, ST 6). Sub-analysis based on intervention duration revealed significant improvement in peak VO_2_ for the exercise group when compared to the control group when the exercise intervention lasted less than 12 weeks or extended beyond 6 months, with no statistically significant difference observed in either group for interventions spanning between 12 weeks and 6 months (Online Resource 6, Supplemental Table 6). The sub-analysis considering supervision of intervention showed a significant improvement of peak VO_2_ for the exercise group compared to the control group within the studies employing supervised sessions, the one study using unsupervised sessions, and the studies incorporating a combination of regular supervised and unsupervised sessions (Online Resource 6, Supplemental Table 6). In our sub-analysis based on frequency of intervention, a significant improvement in peak VO_2_ was noted for the exercise groups undertaking 3 sessions per week, as well as those undertaking 2–3 and 1–4 sessions per week, and daily sessions when compared to the control group (Online Resource 6, Supplemental Table 6). It is worth noting that these last three categories each included one study only. The sub-analysis examining the impact of exercise on peak VO_2_ within the sub-groups of CKD revealed significant improvements for the exercise group in the sub-groups CKD 3–4 and CKD 3, 4, 3–5 when compared to the control group (Online Resouorce 6, Supplemental Table 6).

***Peak respiratory rate*** data were available in 4 studies [20, 53, 55, 56] involving 148 participants (Online Resource 7 Supplemental Fig. 15a and b). Our pooled analysis did not reveal any significant differences between the intervention and control groups (Table 2). Sub analyses based on intervention modality, duration, supervision, and CKD stage also failed to demonstrate statistical significance for either group (Online Resource 6, Supplemental Table 6).

**Fig. 3:**
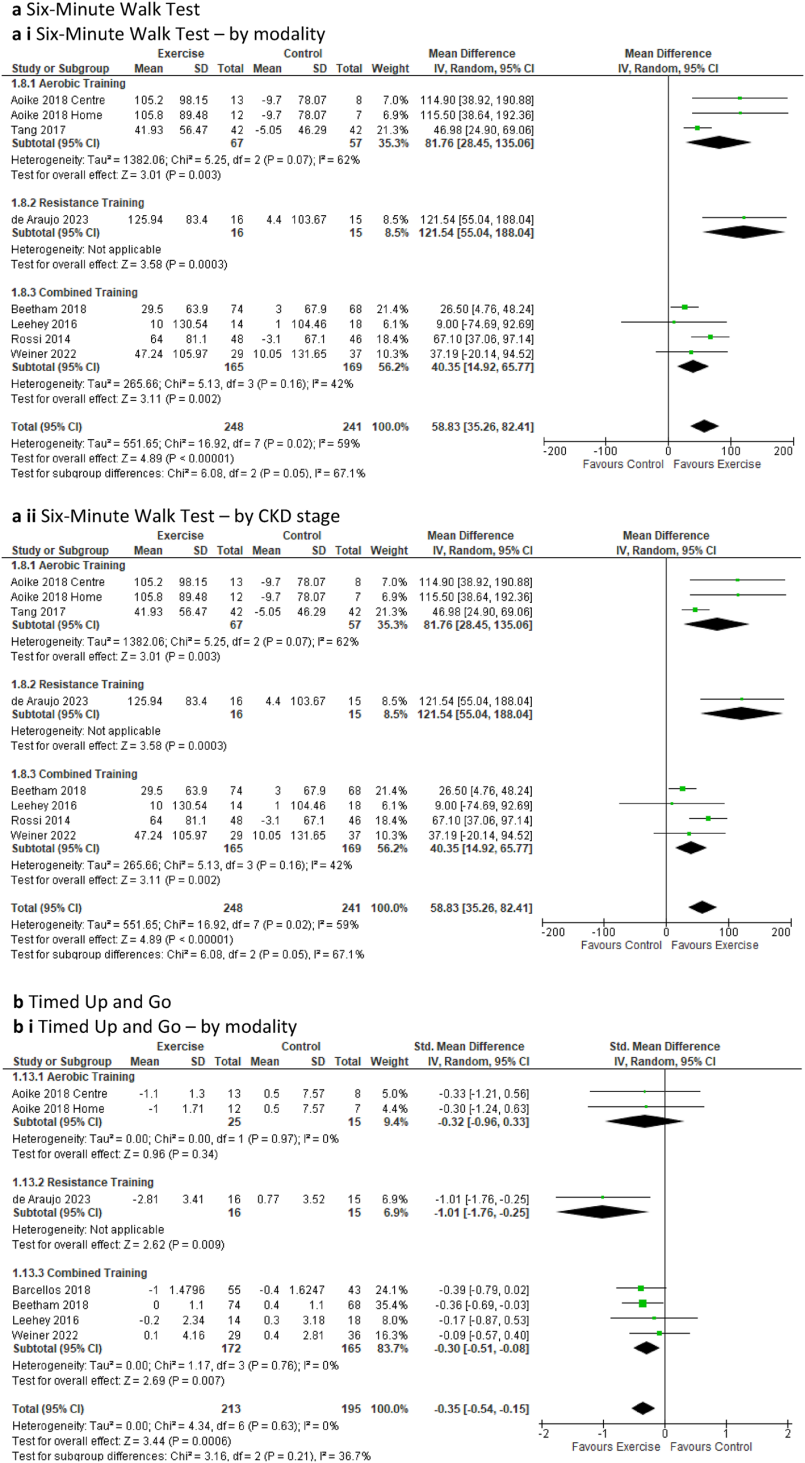

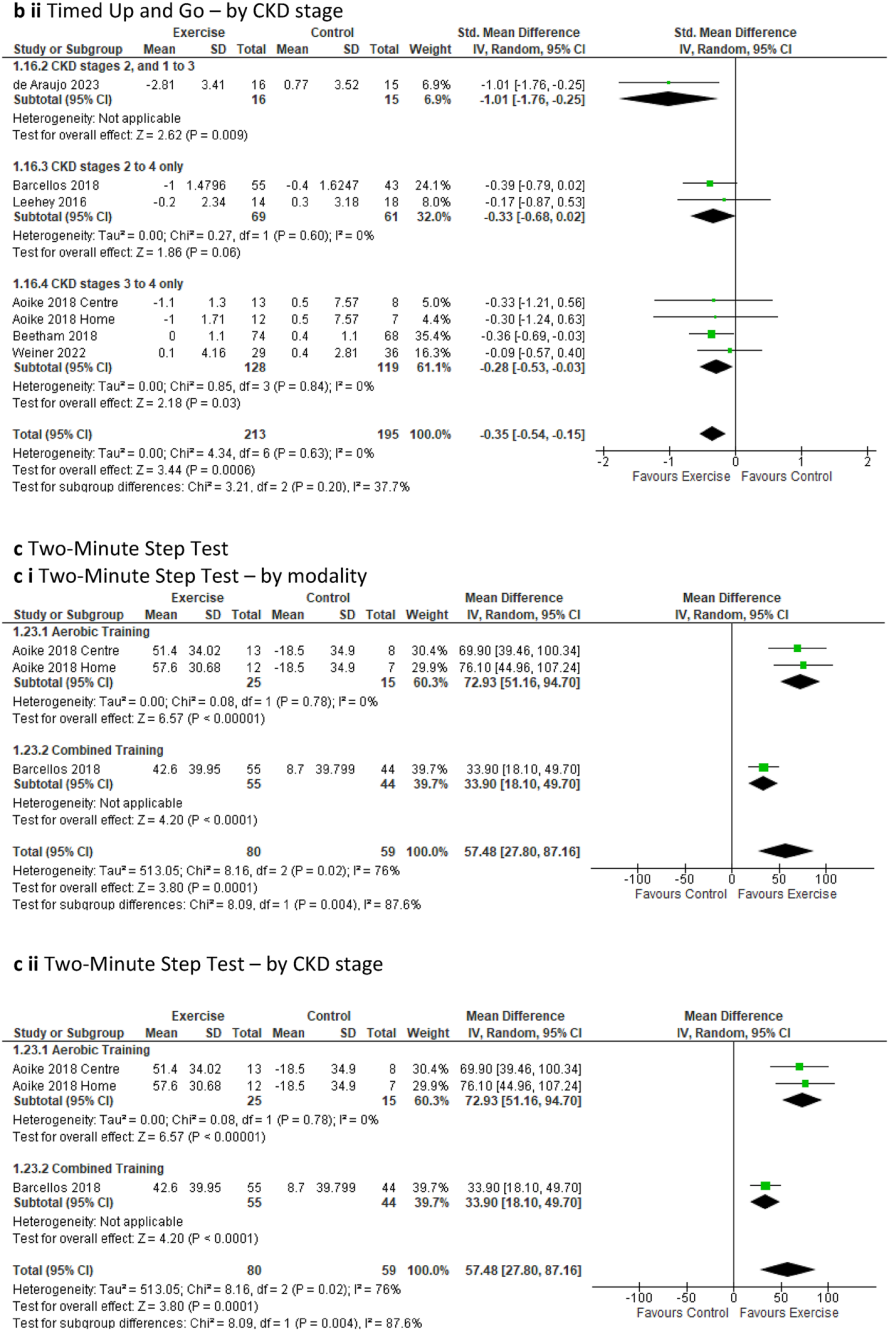

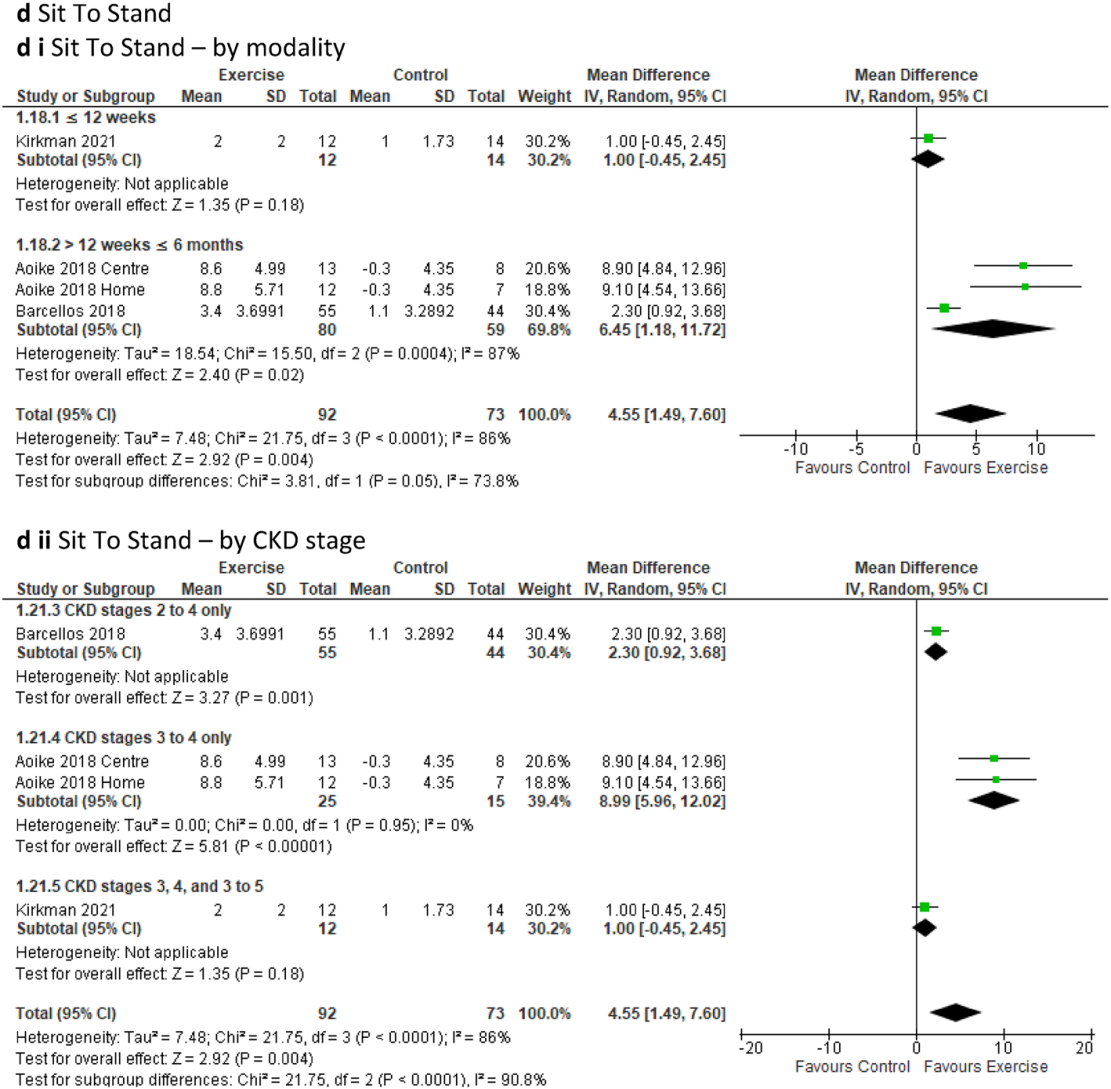
Change in functional ability in people with pre-dialysis CKD by modality i) and CKD stage ii)—exercise versus control: **a** six-minute walk test (6MWT) [meters]; **b** timed up and go (TUG) [minutes]; **c** two-minute step test (2MST) [number of steps achieved in two minutes]; **d** sit to stand (STS) [number of repetitions in 30 s]

***The six-minute walk test*** was reported in seven studies involving 8 intervention groups and 489 participants [18, 25, 39, 46, 49, 51, 57]. Our analysis revealed a statistically significant increase in the distance walked in six minutes for the exercise group compared to the control group (MD 58.83 m; 95% CI 35.26 to 82.41; *p* < 0.00001) (Fig. 3a). Sub-analyses removing low-quality studies from pooled analyses continued to show an improvement in the 6MWT for those participants in the intervention group compared to the control group (Online Resource 5, Supplemental Ffigure 10b).

Further sub-analyses of the aerobic, resistance which included one study only and combined training, all durations, and both the supervised and unsupervised (one study only) intervention sub-groups demonstrated significant improvements in the distance walked by the exercise intervention group compared to the control group (Online Resource 6, Supplemental Table 6). The sub-analysis of CKD stages indicated a significant improvement in the distance covered in the 6MWT for the exercise group compared to the control group for the sub-groups CKD 2, 1–3, and CKD 3–4. These findings mirror the results observed for peak VO_2_ (OR 6, ST 6).

Pooled analyses of the ***timed up and go test*** involving six studies, 7 intervention groups, and 408 participants [18, 25, 39, 47, 49, 51] indicated a significant decrease in the time required to perform the test for the exercise group compared to the control group (SMD − 0.35; 95% CI − 0.54 to − 0.15; *p* = 0.0006) (Fig. 3b). Typically, the timed up and go test test involves a three-meter distance. However, the study by Barcellos [47] measured timed up and go test over a distance of 8 feet (2.44m). Consequently, we analysed this particular outcome using the standard mean difference (SMD) due to the variation in measurement units between studies. Our sensitivity analysis revealed a loss of significant benefit when the study by Barcellos was removed, with *p* = 0.07 (Online Resource 8, Supplemental Fig. 20a).

The change in time taken to perform the timed up and go test remained significantly decreased in the exercise group compared to the control group with low-quality studies removed (Online Resource 5, Supplemental Fig. 10c). Further sub-analyses of timed up and go test also revealed a significant decrease in the time required to perform the test for the exercise group in both resistance (one study only) and combined training, as well as training durations spanning more than 12 weeks to less than 6 months, and over 6 months (Online Resource 6, Supplemental Table 6). These improvements in timed up and go test were consistent for both supervised and initially supervised exercise interventions versus the control group (Online Resource 6, Supplemental Table 6). Sub-analysis of the sub-groups involving CKD stages revealed a significant decrease in time taken to perform the test by the exercise group compared to the control group for sub-groups CKD 2, 1–3 (one study only), CKD 2–4, and those in CDK 3–4 (Online Resource 6, Supplemental Table 6).

The ***two-minute step test*** comprising two studies, 3 intervention groups, and 139 participants [47, 58] (MD 57.48 steps; 95% CI 27.80 to 87.16; *p* = 0.0001) (Fig. 3c) and the ***30-s sit to stand test*** involving three studies, 4 intervention groups, and 165 participants [20, 47, 58] (MD 4.55 repetitions; 95% CI 1.49 to 7.60; *p* = 0.004) (Fig. 3d), both showed significant improvements in the performance of the tests by the exercise groups compared to the control group. The sensitivity analysis for the sit to stand test failed to continue this improvement when the study by Barcellos [47] was removed, leading to *p* = 0.06 (Online Resource 8, Supplemental Fig. 20b).

Sub-analyses of the two-minute step test and sit to stand test tests revealed significantly improved changes in performance by the intervention group for the aerobic and combined (one study only) training sub-groups as well as for training duration of more than 12 weeks but less than or equal to 6 months when compared to the control group (Online Resource 6, Supplemental Table 6). Significant improvements in two-minute step test and sit to stand test were also observed in the exercise sub-groups that were supervised and initially supervised (one study only) compared to the control group (Online Resource 6, Supplemental Table 6). Additionally, participants in CKD sub-groups of stages CKD 2–4 and CKD 3–4 (one study only for each) displayed significant improvements in the two-minute step test and sit to stand test compared to the control group (Online Resource 6, Supplemental Table 6).

***Handgrip strength*** was assessed in seven studies [18, 20, 24, 26, 43, 49, 59] with 372 participants. The analysis did not reveal any significant gain in strength or differences between intervention and control groups (Online Resource 7, Supplemental Fig. 15c and d). The removal of one study [25] deemed low-quality, however, resulted in attainment of a statistically significant gain in strength for the exercise intervention group (*p* = 0.0003) (Online Resource 5, Supplemental Fig. 10d).

Sub-analyses of handgrip strength based on exercise modality, revealed a significant improvement in strength in favour of resistance training. Similarly, significant results for handgrip strength emerged in favour of the exercise intervention group for duration spanning more than 12 weeks to less than 6 months, and also for the sub-groups undergoing supervised sessions, when compared to the control group (Online Resource 6, Supplementary Table 6). However, the sub-analysis of exercise versus control based on CKD stage sub-groups failed to demonstrate statistical significance for either sub-group (Online Resource 6, Supplemental Table 6). Handgrip strength also displayed a significant improvement in favour of the exercise group (*p* = 0.04) on sensitivity analysis when the study by Beetham [49] was removed (Online Resource 8, Supplemental Fig. 21a).

#### Quality of life

The included studies employed comparable versions of the short form (SF)-36 questionnaire [60], with lower scores indicating poorer quality of life [61]. Three studies used the Kidney Disease Quality of Life SF-36 [24, 53, 57], two the Health Related Quality of Life SF-36 [50, 52], two the Kidney Disease Quality of Life SF-12 [21, 23], one the Medical Outcomes Study SF-36 Health Survey [51], and one the RAND 36-Item SF Health Survey [46].

**Fig. 4:**
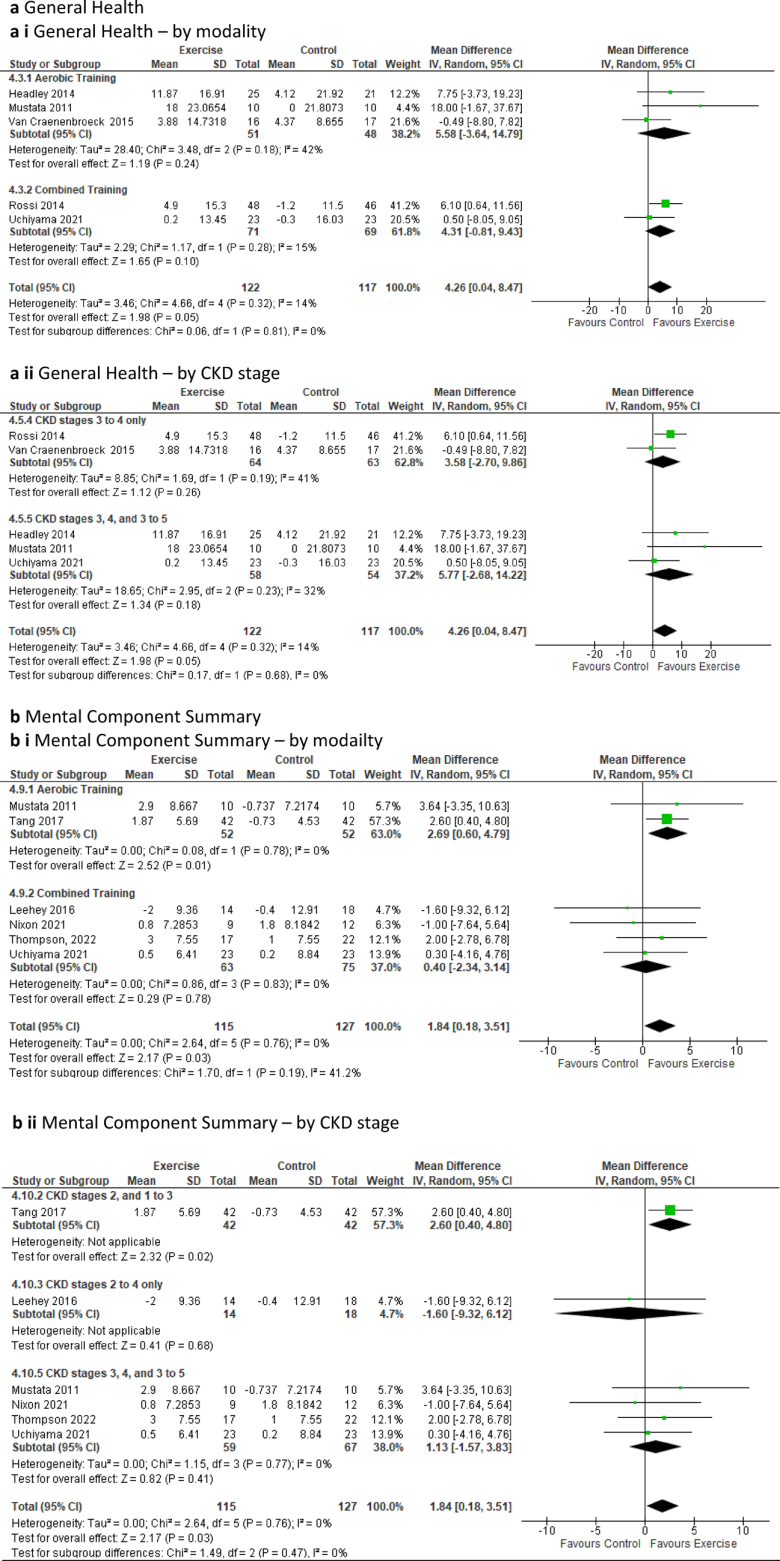
Change in quality of life (QoL) in people with pre-dialysis CKD by modality i) and ii) by CKD stage—exercise versus control: **a** Short Form (SF)-36 General Health (GH); **b** SF-36 Mental Component Summary (MCS)

For ***SF-36 general health***, data from five studies involving 239 participants [24, 46, 50, 52, 53] indicated that exercise had a positive change of effect on general health for the intervention group compared to the control group (MD 4.26; 95% CI 0.04 to 8.47; *p* = 0.05) (Fig. 4a). Six studies with 242 participants [21, 23, 24, 51, 52, 57] were included for both ***SF-36 physical component summary*** and ***SF-36 mental component summary*** (Table 2). Mental component study analysis revealed a significant positive change for the exercise group compared to the control group (MD 1.84; 95% CI 0.18 to 3.51; *p* = 0.03) (Fig. 4b), however, no significant improvement for either group was observed in physical component study (Online Resource 7, Supplemental Fig. 16a and b).

Our sub-analyses of general health, mental component study and physical component study excluding low-quality studies from pooled analyses, signalled a loss of significant effect between the exercise and control group for general health and mental component study, and did not yield a change for physical component study (Online Resource 5, Supplementary Fig. 11a to c). Sub-analyses indicated a significant positive change in general health for the exercise group compared to the control group within the supervised sub-group, and physical component study displayed a significantly positive change for the exercise group undertaking aerobic training when compared to the control group (Online Resource 6, ST 6). Mental component study exhibited a significant improvement for the aerobic training, duration of less than 12 weeks, and the unsupervised sub-groups when compared to the control group (Online Resource 6, Supplemental Table 6). Sub-analysis of the CKD stage sub-groups revealed a significant positive change over time for the exercise group when compared to the control group for the sub-group CKD 2, 1–3 for both physical component study and mental component study, however, only one study was involved in this range (Online Resource 6, Supplemental Table 6). Sensitivity analysis showed a significant improvement for physical component study (*p* = 0.02) in the exercise group upon removal of the study by Nixon [21] (Online Resource 8, Supplementary Fig. 21b). Sensitivity analysis revealed a loss of significant improvement for general health in the exercise group compared to the control group when the studies by Headley [50] and Rossi [46] were removed (Online Resource 8, Supplelemental Fig. 20c and d). Sensitivity analysis also revealed a loss of significant improvement for mental component study for the exercise group compared to control upon removal of Tang [57] (Online Resource 8, Supplementary Fig. 20e).

#### Renal parameters

**Fig. 5:**
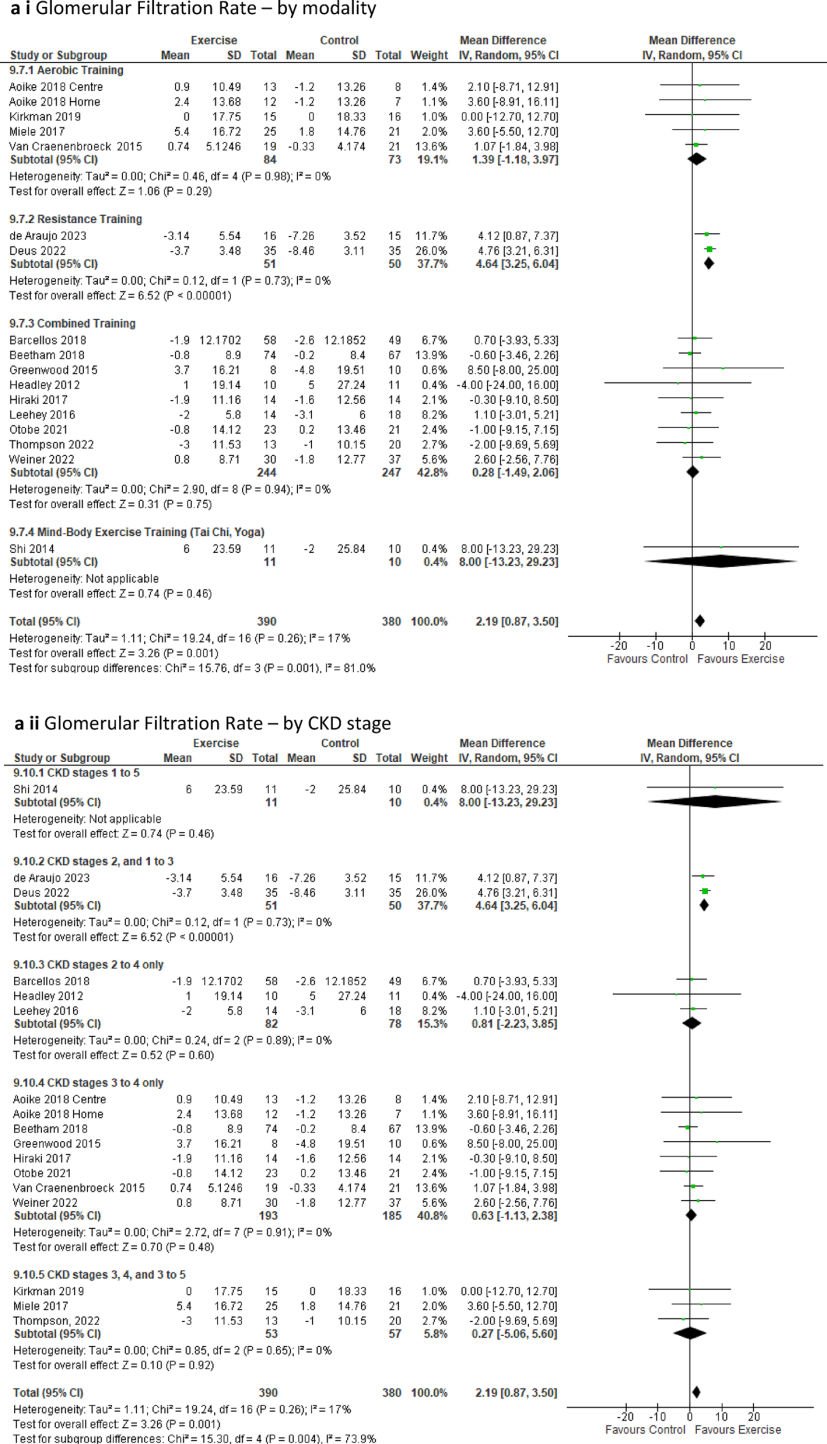

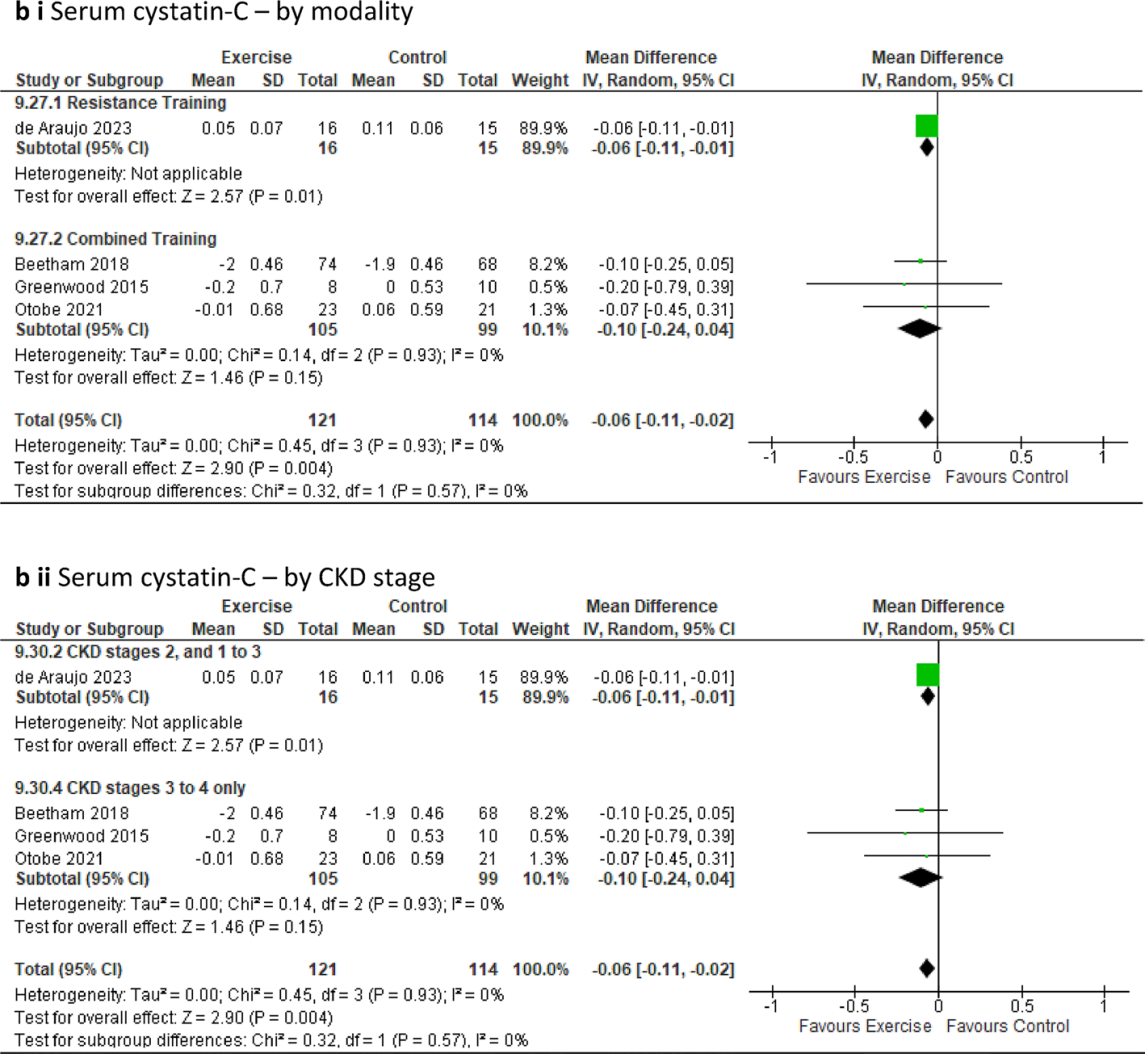
Change in Renal parameters in people with pre-dialysis CKD by modality i) and CKD stage ii)—exercise versus control: **a** Glomerular Filtration Rate (eGFR_Cr_) [ml/min/1.73 m^2^]; **b** Serum cystatin-C (mg/L)

The ***estimated glomerular filtration rate (eGFR***_***Cr***_***)****,* calculated using creatinine clearance rate, was evaluated in 16 studies comprising 17 intervention groups and a total of 770 participants [18, 19, 22, 23, 25, 39, 41–45, 47, 49, 51, 53, 62]. The analysis revealed a significant positive impact (via a slower decline in eGFR over time of trial) for the exercise group compared to the control group (MD 2.19 mL/min/1.73 m^2^; 95% CI 0.87 to 3.50; *p* = 0.001) (Fig. 5a). In our analysis, we excluded two studies: one [40] because the calculation was based on EDTA, and the other [54] because the reported units differed from the standard measurement. This statistically significant improvement of eGFR remained in favour of the intervention group upon removal of low-quality studies *p* = 0.02 (Online Resource 5, Supplemental Fig. 12a).

Sub-analyses of eGFR for resistance training, intervention durations exceeding 12 weeks but less than or equal to 6 months, supervised resistance training, and the CKD stage sub-group CKD 2, 1–3, all indicated a significantly slower decline of eGFR over time for the exercise group compared to the control (Online Resource 6, Supplemental Table 6). Our sensitivity analysis revealed the removal of the study by Deus [19] resulted in a loss of statistical significance for the intervention group to *p* = 0.06 (Online Resource 8, Supplemental Fig. 20f).

The ***estimated glomerular filtration rate (eGFR***_***Cys***_***)***, calculated using cystatin-C, was assessed in 4 studies involving 246 participants [22, 41, 48, 49]. Our analysis did not reveal statistically significant effects of exercise over time for either the exercise or control groups (OR 7, SF 17a and b). Sub-analyses which considered modality, duration, supervision, and CKD stage also failed to detect any notable change in eGFR in either group (Online Resource 6, Supplemental Table 6).

***Serum cystatin-C*** was analysed in four studies with 235 participants [18, 22, 41, 49] revealing a significant improvement of levels in favour of the intervention group compared to the control group (MD − 0.06mg/L, 95% CI − 0.11 to − 0.02; *p* = 0.004) (Fig. 5b). However, upon sensitivity analysis, this significant effect was lost when the study by de Araujo [18] was excluded (Online Resource 8, Supplemental Fig. 20g).

Several other renal parameters, including ***serum creatinine*** (in nine studies with 359 participants) [18, 22–24, 41, 44, 49, 54, 59, 63], **s*erum albumin*** (in eight studies with 360 participants) [18, 19, 22, 24, 48, 56, 58, 64], ***urine albumin-to-creatinine (UACR)*** (in four studies with 252 participants) [50, 53, 57, 74], ***urine protein-to-creatinine ratio (UPCR)*** (in five studies with 257 participants) [22, 43, 49, 51, 54], ***24-h urine protein*** (in three studies comprising four intervention groups with 72 participants) [39, 42, 54], and ***blood urea nitrogen (BUN)*** (in four studies with 118 participants) [22, 44, 48, 54] did not exhibit significant changes over time for either the intervention or control group (OR 6, ST 6; OR 7, SF 17c to n). Sub-analyses that excluded low-quality studies from pooled analyses failed to alter the results of these renal parameters (Online Resource 5, Supplemental Fig. 12b to f). Sub-analyses of serum creatinine (one study only), albumin and serum cystatin-C (1SO) levels showed a positive significant change over time in favour of the intervention group for resistance training, intervention duration greater than 12 weeks but less than or equal to 6 months, supervised exercise sessions, and the sub-group CKD 2, 1–3, when compared to the control group (Online Resource 6, Supplemental Table 6). Sensitivity analysis revealed a gain in significant effect for the exercise group compared to the control group for serum creatinine (*p* = 0.04) upon the removal of the study by Beetham [49], and for albumin (*p* = 0.05) upon exclusion of the study by Aoike [58] (Online Resource 8, Supplemental Fig. 21c and d).

#### Cardiovascular risk factors

**Fig. 6:**
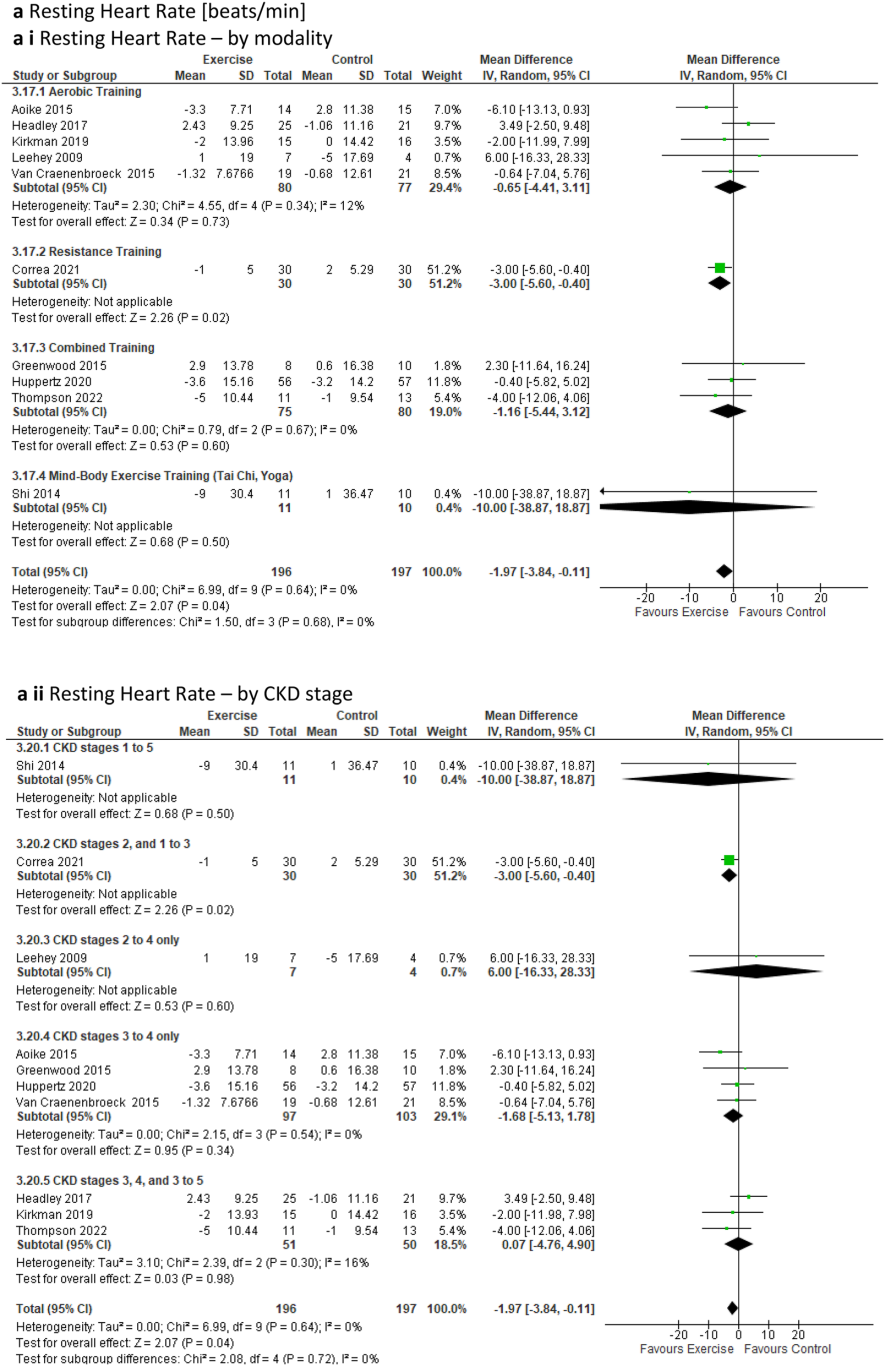

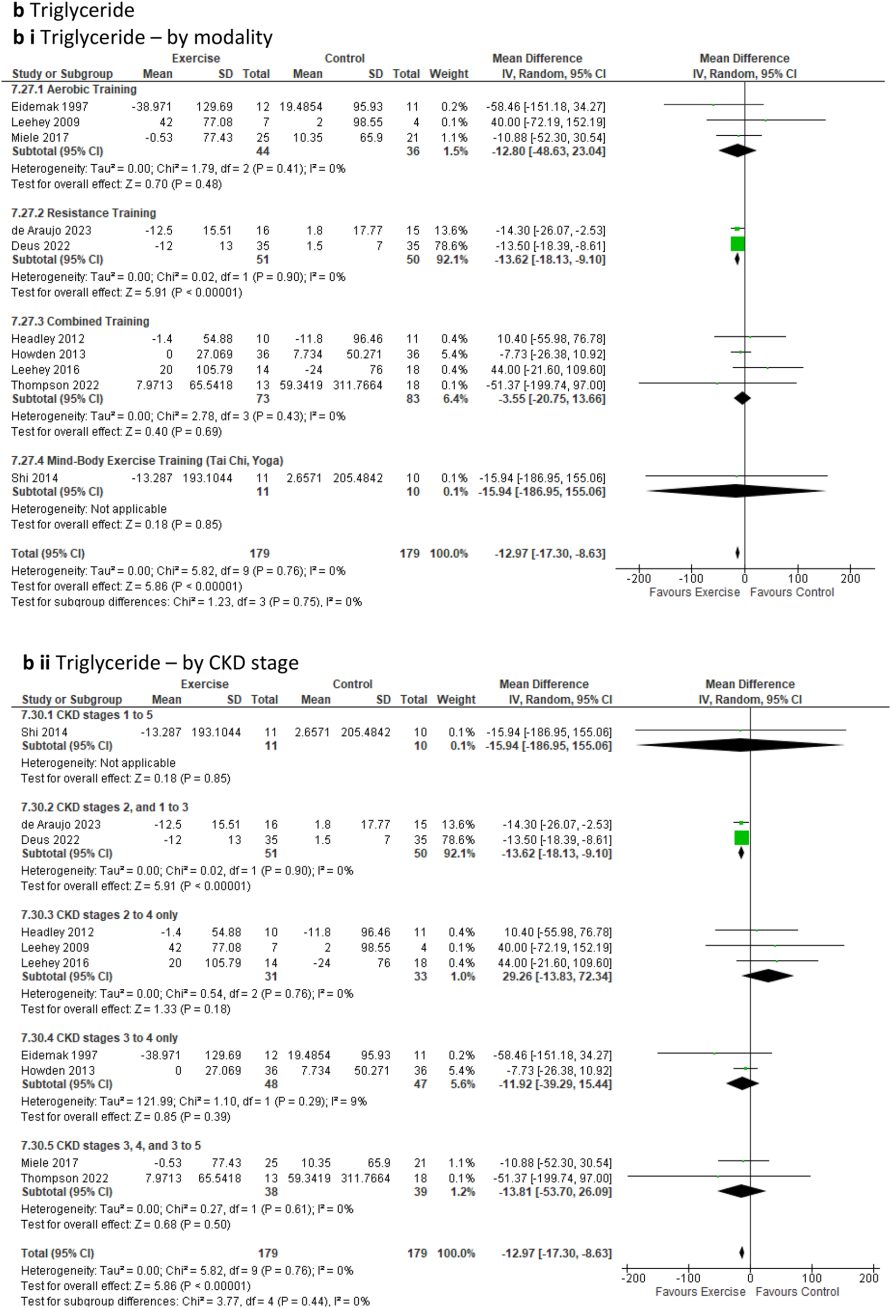
Change in Cardiovascular Risk Factors in people with pre-dialysis CKD– exercise versus control: **6a** Resting Heart Rate [beats/min] by modality i) and CKD stage ii); **6b** Triglyceride [mg/dL] by modality i) and CKD stage ii); **6c** Glycosylated haemoglobin [%] by modality i) and CKD stage ii); **6d** Waist circumference [cm] by modality i) and CKD stage ii)


Several cardiovascular risk-related outcomes were assessed. ***Resting heart rate*** analysis, which included 10 studies involving 393 participants [23, 26, 41, 44, 45, 53–55, 58, 65] revealed a statistically significant improvement over time (via a reduction of beats per minute) for the exercise group when compared to the control group (MD − 1.97beats/min; 95% CI − 3.84 to − 0.11; *p* = 0.04) (Fig. 6a). Sub-analysis with low-quality studies removed continued to show the significant impact of exercise on resting heart rate in favour of the intervention group (Online Resource 5, Supplemental Fig. 13a). Furthermore, our sub-analysis based on exercise modality indicated a significant improvement for resistance training (one study only) and for the CKD2, 1–3 sub-group (one study only) in favour of the intervention group (Online Resource 6, Supplemental Table 6). However, the leave-one-out sensitivity analysis revealed no difference between the exercise and control groups upon the removal of the Aoike study [58], as well as upon the exclusion of Correa [26], and Thompson [23] (Online Resource 8, Supplemental Fig. 20h to j).

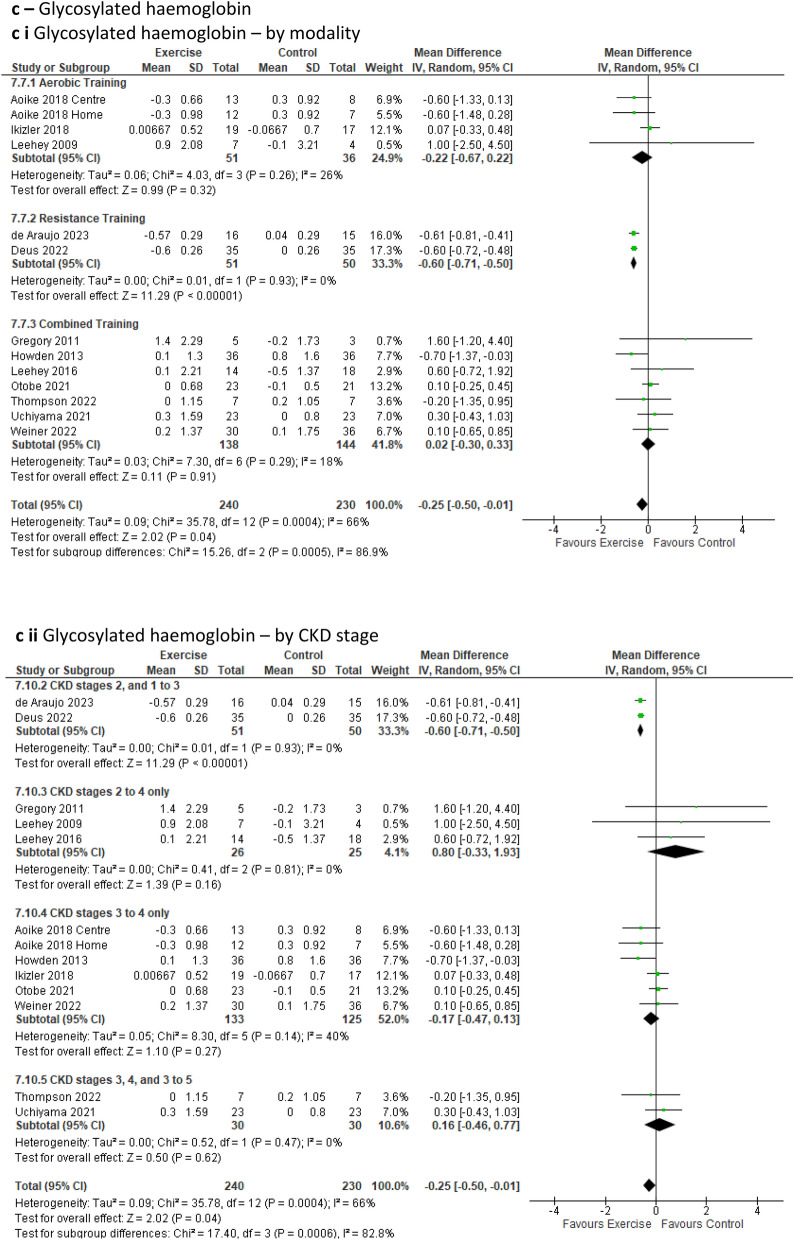

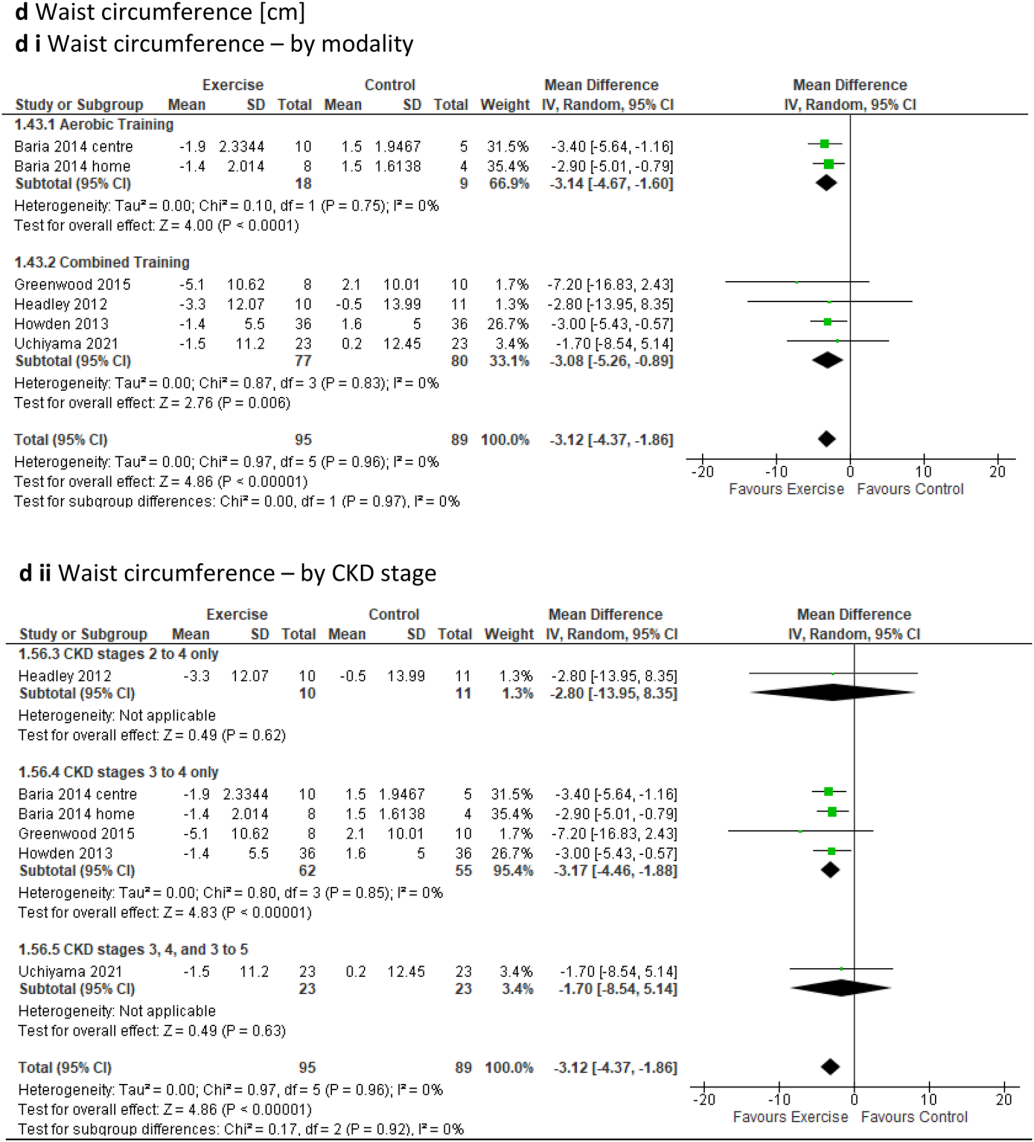


***Resting systolic blood pressure (SBP)***, evaluated in 15 studies encompassing 16 intervention groups and 665 participants [18, 23, 25, 26, 39, 41, 42, 44, 45, 47, 48, 50, 51, 54, 65], and ***resting diastolic blood pressure (DBP)***, investigated in 14 studies involving 15 intervention groups and, 633 participants [18, 23, 25, 26, 39, 41, 42, 44, 45, 47, 48, 50, 54, 65] showed no statistically significant decreases of either the systolic or diastolic figures over time in the exercise or control groups (OR 7, SF 17a to d). The sub-analyses, which excluded low-quality studies from pooled analyses, also revealed no difference between the two groups (OR 5, SF13b to c). ***Ambulatory 24-h SBP*** and ***ambulatory 24-h DBP*** were investigated in four studies involving 164 participants [23, 26, 42, 55], with analysis failing to yield statistically significant findings in either group (Online Resource 7, Supplemental Fig. 18e to h). The sub-analyses which excluded low-quality studies from pooled analyses, continued to show no difference between the two groups (Online Resource 5, Supplemental Fig. 13d and e).

The sub-analyses of these blood pressure outcomes indicated a significant improvement in favour of the intervention group for resistance training as well as the sub-group CKD2, 1–3 for SBP. Sub-analyses also showed a significant effect of resistance training on Ambulatory 24-h SBP and DBP within the intervention group, however it is essential to note that these findings are based on only one study (Online Resource 6, Supplemental Table 6).

**Endothelial function** assessment included ***pulse wave velocity*** in six studies comprising 253 participants [24, 41, 45, 50, 53], the aortic ***augmentation index*** in five studies involving 185 participants [23, 45, 52, 53, 56] and ***asymmetric dimethylarginine*** in three studies with a total of 96 participants [18, 22, 42]. None of the analyses for these outcomes yielded statistically significant results for either the intervention or control group (Online Resource 7, Supplemental Fig. 19i to n). A sub-analysis did reveal a significant improvement in favour of the intervention group for augmentation index in the sub-group undergoing a combination of supervised and unsupervised sessions, and in asymmetric dimethylarginine levels for resistance training (one study only) and the sub-groups CKD 2, 1–3 and CKD 2–4 (both one study only) compared to the control group (Online Resource 6, Supplemental Table 6). Sub-analysis by removing the low-quality study failed to demonstrate a significant difference for either group (Online Resource 5, Supplemental Fig. 13f).

***Lipid studies*** Exercise interventions yielded a significant beneficial reduction in ***triglyceride*** levels across ten studies involving 358 participants [18, 19, 23, 40, 42, 44, 51, 54, 56, 62] (MD − 12.97mg/dL; 95% CI − 17.30 to − 8.63; *p* < 0.00001) in favour of the exercise group (Fig. 6b). This statistical significance continued upon exclusion of low-quality studies (Online Resource 5, Supplemental Fig. 13g). Exercise did not produce statistically significant changes in favour of either group for ***total cholesterol*** levels across 14 studies involving 617 participants [18, 19, 23, 40–42, 44, 47, 51, 53, 54, 56, 62, 66], ***high density lipoprotein (HDL-C)*** across 13 studies involving 543 participants [18, 19, 22, 23, 41, 42, 44, 47, 51, 53, 54, 56, 62], and ***low density lipoprotein (LDL-C)*** across 13 studies involving 538 participants [18, 19, 22, 23, 41, 42, 44, 47, 51, 53, 54, 56, 62] (Online Resource 7, Supplemental Fig. 18o to t). Sub-analyses excluding low-quality studies from pooled analyses continued to demonstrate no difference in either group for these lipid outcomes (Online Resource 5, Supplemental Fig. 13h to j).

Further sub-analyses of the lipid studies revealed a significant reduction of triglycerides in favour of the intervention group in the categories of resistance training, duration of intervention greater than 12 weeks but less than six months, and supervised sessions, as well as within the sub-group CKD 2, 1–3, when compared to the control group (Online Resource 6, Supplemental Table 6). Total cholesterol displayed significant improvements in favour of the intervention group in sessions that were both supervised and unsupervised, and in the sub-group CKD 3, 4, 3–5 (Online Resource 6, Supplemental Table 6). HDL levels exhibited significant change in favour of the intervention group with resistance training and within the sub-group CKD 2, 1–3 when compared to the control group (Online Resistance 6, Supplemental Ttable 6). In contrast, LDL levels exhibited statistically significant changes in favour of the control group for aerobic training, combined training, in sessions that were both supervised and unsupervised, and in the sub-group CKD 2–4 when compared to the intervention group (Online Resource 6, Supplemental Table 6). Sensitivity analysis showed significant improvements in favour of the intervention group for total cholesterol (*p* = 0.03) upon removal of Chen [66]. LDL revealed a significant change in favour of the control group with removal of the studies by de Araujo [18] (*p* = 0.04), Headley [42] (*p* = 0.02), and Howden [56] (*p* = 0.03). HDL showed significant improvement in favour of the intervention upon group removal of Barcellos [47] (*p* = 0.05) (Online Resource 8, Supplemental Fig. 21e to i).

***Blood parameters*** were analysed revealing a significant positive impact of exercise on ***glycosylated haemoglobin (HbA1c)*** levels in favour of the exercise group via lower increases in HbA1c levels over time compared to the control group (Fig. 6c). This improvement was observed across 12 studies involving 13 intervention groups and 470 participants [18, 19, 22–25, 39, 48, 51, 54, 56, 63] (MD 0.25%; 95% CI –0.50 to − 0.01; *p* = 0.04). Upon removal of low-quality studies, HbA1c remained significant in favour of the intervention group at *p* = 0.02 (OR 5, SF 13k); however, sensitivity analysis of HbA1c showed no significant findings for either group upon individual removal of several studies [17, 19, 84, 85] (OR 8, SF 20k to o).

***Blood glucose,*** assessed in four studies with 313 participants [19, 47, 48, 66] and ***haemoglobin (Hb)***, analysed in five studies encompassing 6 intervention groups and 248 participants [22, 54, 66–68] did not reveal significant findings in either group (Online Resource 7, Supplemental Fig. 18u to x). This lack of significant findings did not change upon removal of low-quality studies for blood glucose and Hb for either group (OR 5, SF 13l to m). However, a significant improvement for Hb (*p* = 0.04) in favour of the intervention group was evident upon sensitivity analysis with the exclusion of Howden [68] (Online Resource 8, Supplemental Fig. 21j). Further sub-analyses revealed a significant reduction in blood glucose levels in favour of the intervention group in the resistance training category as well as for sub-group CKD 2, 1–3 when compared to the control group, although it is important to note that these findings were based on single-study results (Online Resource 6, Supplemental Table 6). Hb was seen to significantly improve in favour of the intervention group with aerobic training, intervention duration of less than 12 weeks as well as lasting over six months (1SO), in the supervised and initially supervised then unsupervised (one study only) group, as well as in sub-group CKD 3–4 group when compared to the control group (Online Resource 6, Supplemental Table 6).

***Body composition parameters*—*waist circumference*** was evaluated in five studies encompassing six intervention groups and 184 participants [24, 41, 42, 56, 67]. The analysis indicated a significant reduction in measurement in favour of the exercise group (MD − 3.12cm; 95% CI –4.37 to − 1.86; *p* < 0.00001) compared to the control group (Fig. 6d). This significant effect on waist circumference remained in favour of the exercise group after the exclusion of low-quality studies (OR 5, SF 13n). Significant results in favour of the exercise group were also observed during sub-analyses for both aerobic and combined training, interventions lasting less than 12 weeks and more than 6 months, in supervised settings as well as those initially supervised then unsupervised, and within the sub-group CKD 3–4 when compared to the control group (Online Resource 6, Supplemental Table 6).

The analysis of ***body weight*** (9 studies, 458 participants) [19, 23, 41, 42, 47, 49, 54, 59, 64], ***body mass index (BMI)*** (13 studies, 14 intervention groups, 614 participants) [19, 23–25, 41, 42, 48–51, 53, 58, 64], ***body fat*** (5 studies, 211 participants) [19, 48, 50, 51, 58], and ***lean body mass*** (6 studies, 7 intervention groups, 229 participants [18, 19, 23, 51, 62, 67] all indicated no significant change with the inclusion of exercise when comparing the two groups (Online Resource 7, Supplemental Fig. 18y to af). These findings remained consistent when low-quality studies were considered in sub-analyses (Online Resource 5, Supplemental Fig. 13o to q).

In further sub-analyses, body weight change was seen to significantly favour the exercise group with less weight gain over time compared to the control group within the combined training category, interventions lasting less than 12 weeks (one study only) and longer than 6 months categories, as well as for the sub-group CKD 2, 1–3 (one study only) (Online Resource 6, Supplementary Table 6). BMI change was found to significantly favour the exercise group with less increase in BMI over time compared to the control group with both aerobic and combined training, interventions lasting longer than 12 weeks but less than 6 months, and those lasting more than 6 months, across the initially supervised and then unsupervised and the combination of supervision and no supervision categories, and within the sub-group CKD 2, 1–3 (1SO) (Online Resource 6, Supplemental Table 6). In the case of body fat, significantly lower increases in body fat for the exercise group were observed with resistance training (one study only) and within the sub-group CKD 2, 1–3 (one study only) compared to the control group (Online Resource 6, Supplemental Table 6). Lastly, sub-analyses for lean body mass demonstrated significant improvements in favour of the exercise group compared to the control group with resistance training (one study only), interventions lasting longer than 12 weeks but less than 6 months, supervised sessions, and within the sub-group CKD 2, 1–3 (one study only) (Online Resource 6, Supplemental Table 6).

Upon conducting the sensitivity analysis for body composition parameters, significant improvements in favour of the exercise group compared to the control group were noted for body weight (*p* = 0.0003) and BMI (*p* < 0.0001) upon exclusion of the Castaneda study [64]. Furthermore, sensitivity analysis demonstrated significant improvements in favour of the exercise group compared to the control group for body fat (*p* = 0.0004) following the removal of Ikizler [48] and for lean body mass (*p* = 0.0001) after the exclusion of Baria [67] (Online Resource 8, Supplemental Fig. 21k to n).

#### Inflammatory markers

**Fig. 7:**
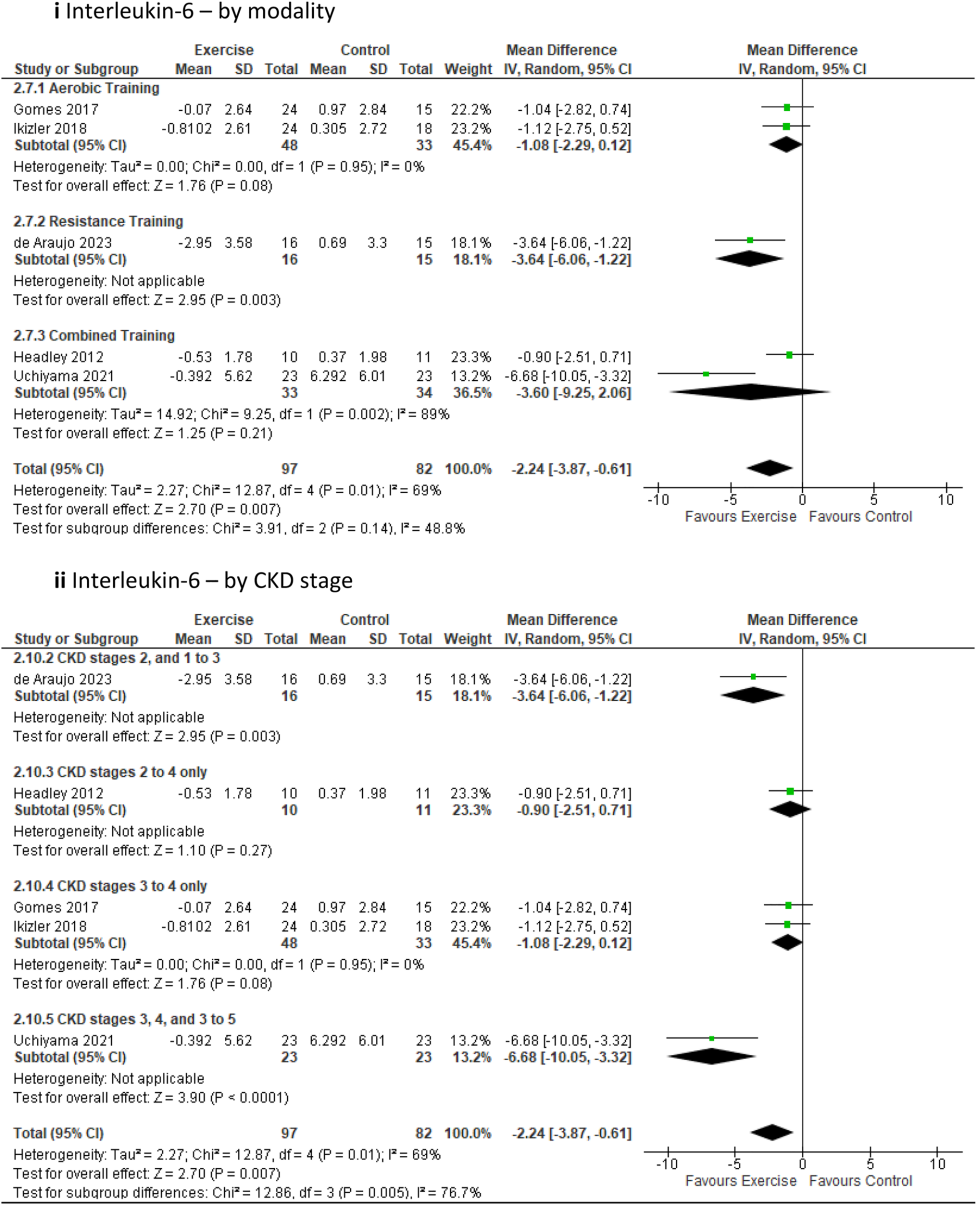
Change in inflammatory markers in people with pre-dialysis CKD by modality—exercise versus control via interleukin-6 [pg/mL]

***Interleukin-6 (IL-6)*** analysis, involving five studies with 179 participants [18, 24, 42, 48, 59] revealed a significant reduction in levels in favour of the exercise intervention (MD − 2.24pg/mL; 95% CI − 3.87 to − 0.61; *p* = 0.007) (Fig. 7). These significant results for IL-6 continued in favour of the exercise intervention group when low-quality studies were removed (Online Resource 5, Supplemental Fig. 14a). Further sub-analyses revealed a significant improvement in favour of the exercise group when compared to the control group for resistance training (one study only), interventions lasting longer than 12 weeks but less than six months, for supervised sessions, and within the sub-groups CKD 2, 1–3 and CKD 3, 4, 3 to 5 (Online Resource 6, Supplemental Table 6). On the other hand, ***C-reactive protein (CRP)*** evaluation encompassing 12 studies with 499 participants [22–24, 41, 42, 47, 50, 51, 53, 54, 59, 68] yielded no significant results for either group (Online Resoource 7, Supplemental Fig. 19a and b). The results for CRP remained consistent upon removal of low-quality studies (Online Resource 5 Supplemental Fig. 14b), and via sub-analysis of modality, duration, supervision, high sensitivity (hs)-CRP and non hs-CRP, and CKD stage (Online Resource 6, Supplemental Table 6).

## Discussion

This systematic review with meta-analyses demonstrates that exercise has the potential to improve numerous outcomes for people with pre-dialysis CKD including aerobic capacity and functional ability, quality of life, eGFR_Cr_, serum cystatin-C, resting heart rate, triglyceride, glycosylated haemoglobin and interleukin-6 levels, and waist circumference. This review offers the most wide-ranging meta-analyses and sub-analyses of CKD G1 to pre-dialysis G5 outcomes performed to date, with several previously unreported outcomes. These include the two-minute step test, body fat, lean body mass, asymmetric dimethylarginine and the physical and mental component summaries of quality of life questionnaires. We also understand our paper is novel in presenting statistically significant results for estimated glomerular filtration rate, glycosylated haemoglobin and serum cystatin-c levels. Furthermore, our comprehensive analysis includes a sub-analysis based on the CKD stages that each included study has reported on in their research.

Aerobic capacity and functional ability, commonly assessed through peak VO_2_ [69] and the 6MWT [70], generally exhibit a gradual decline with increasing severity of CKD [71]. These assessments also serve as valuable predictors of all-cause-mortality and endurance [72, 73]. Our analysis revealed a significant increase of peak VO_2_ in the exercise group compared to the control group aligning with findings from previous reviews [13, 14, 16, 74, 75]. Notably, the magnitude of change in peak VO_2_ approximates nearly one metabolic equivalent of task (MET) which could be considered the minimum clinically important difference.

In our sub-analysis focusing on exercise modality, we observed significant improvements in peak VO_2_ for both aerobic and combined training. However, the aerobic training group exhibited a slightly greater improvement in peak VO_2_ compared to the combined training group. Studies involving resistance training did not report peak VO_2_ outcomes. Sub-analysis of intervention duration indicated that exercising for less than 12 weeks yielded the greatest gain in peak VO_2_, while durations exceeding six months also significantly increased peak VO_2_. These results suggest that exercise’s beneficial effects on peak VO_2_ may plateau approximately 12 weeks after commencing exercise (possibly due to factors such as non-adherence or insufficient progression in exercise intensity and/or volume over time), however, also suggesting that long-term exercise routines continue to improve peak VO_2_.

Our sub-analysis targeting supervised intervention indicated that either fully supervised regimens or a combination of supervised and unsupervised exercise sessions yielded the most pronounced changes in peak VO_2_. In contrast, commencing with supervised training and then transitioning to unsupervised failed to yield significant changes by trial conclusion. These results underscore the positive role of ongoing supervision in achieving and maintaining improvements in peak VO_2_. In terms of CKD stage, our review indicated that participants in stages G3a and b, G4 and pre-dialysis G5 demonstrated significant improvements in peak VO_2_, suggesting that regular exercise is beneficial for improving peak VO_2_ in the later stages of pre-dialysis CKD.

Impaired mobility is known to be a significant contributor to the loss of functional independence [76] with levels often declining alongside a reduction in eGFR which may adversely affect CKD progression [75]. Functional ability represents an individual’s actual or potential capacity to perform everyday tasks and activities. A decline in functional ability correlates with decreased independence, diminished quality of life, and increased mortality risk [73]. In the context of chronic diseases, assessing functional ability serves to predict ongoing risk and guide treatment plans, utilising various assessment tools [75]. Exercise tests that simulate regular daily movements play a crucial role in evaluating functional ability. Our review focused on commonly reported assessment tools employed in RCTs investigating the effects of exercise on CKD over the course of a trial. For example, the 6MWT which measures the distance a person can walk in six minutes, and the timed up and go test, assessing the time it takes to rise from a chair, walk three meters and return to the chair, were among the tools analysed. Our findings consistently demonstrated that exercise has a beneficial effect on functional ability across all assessed tools, with the exception of handgrip strength.

Our analysis of the 6MWT revealed a significant increase in the distance walked within a 6-min timeframe, ranging from 28 to 135 m. Specifically, the mean difference of 82 m observed in the aerobic exercise studies (Online Resource 6, Supplemental Table 6) surpassed the minimum clinically important difference threshold of approximately 32 m. This minimum clinically important difference benchmark is recognized in the context of chronic heart failure patients, who often exhibit physical deconditioning levels comparable to those diagnosed with pre-dialysis CKD [77]. Furthermore, our sub-analyses of the 6MWT confirmed that aerobic, resistance and combined training approaches all led to significant improvements in the distance walked across the various durations (spanning less than 12 weeks, between 12 weeks and 6 months, and longer than 6 months), within supervised sessions, and for all pre-dialysis CKD stages, although only one [18] of the four studies based on resistance training reported on the 6MWT.

In addition to the significant result of the 6MWT, our analysis revealed noteworthy improvements in other functional assessments including timed up and go test, sit to stand test, and two-minute step test. These assessments have been sparsely reported in previous summary analyses [13, 15]. Sub-analyses indicated that resistance and combined training approaches may lead to greater improvements in timed up and go test performance compared to aerobic training, whereas analysis of sit to stand test and two-minute step test underscored the benefit of aerobic and combined training for improving functional ability. Timed up and go test, two-minute step test, and sit to stand test demonstrated significant improvements across all included CKD stages except for pre-dialysis G5—a reasonable outcome given the challenges faced by individuals at this stage of CKD. Although handgrip strength did not reach statistical significance it exhibited a trend toward improvement with exercise.

Considering that functional assessments such as the 6MWT are commonly impaired in individuals with pre-dialysis CKD [78], our results hold clinical significance. These results of improved aerobic capacity and functional ability suggest that integrating regular exercise into the routines of pre-dialysis CKD patients can potentially help them to maintain functional independence at home, contribute to disease mitigation, and reduce mortality risk [79]. With the SONG-CKD group identifying functional ability (life participation) as a high priority outcome for investigation in clinical trials [28], these results offer promise for those living with CKD, and opens new avenues for research.

Individuals with pre-dialysis CKD commonly experience poor HRQoL, which tends to deteriorate further as disease progresses [80]. QoL assessment frequently utilises the short form (SF)-36 health questionnaire, comprising multiple questions across eight domains [61]. In clinical trials, two key summary scores derived from the SF-36 are often reported: the mental component summary and the physical component summary. Additionally, the score general health is often reported) [81]. These measures provide valuable insights into the impact of CKD in patients’ overall well-being and guide intervention aimed at improving QoL.

Our analyses of QoL revealed a significant increase in the mental component study and general health scores for the intervention group compared to the control group. These findings indicate that the inclusion of exercise as a treatment leads to enhanced QoL. The improvement in general health met the minimum clinically important difference criteria ranging from 3 to 5 score units [82], however, the mental component study did not approach minimum clinically important difference. Regarding physical component study, we did not observe a meaningful change in either the exercise or control group. However, a sensitivity analysis excluding the study by Nixon [21], which focused on frail participants with a mean age of 78 years, demonstrated a statistically significant difference (*p* = 0.02) in favour of the exercise group.

We believe our review represents the first meta-analysis of RCT studies comparing an exercise intervention group to usual care in individuals with CKD stages G1 to pre-dialysis G5, and to report on physical component study and mental component study. Our results align with the established correlation between diminished functional ability and poor HRQoL in this population [83] and underscore the potential of regular exercise to enhance QoL for individuals living with pre-dialysis CKD.

While it is known that high levels of physical activity, encompassing both leisure and occupational activities, are linked to a slower decline of eGFR [84], exercise is not routinely recommended in the standard care of people with CKD stages G1 to pre-dialysis G5. This oversight likely stems from various factors, including a failure to fully recognise the potential benefits of exercise [85], and highlights the recommendation by the 2020 SONG-CKD group [28] that kidney function be a priority research topic within CKD research.

Our present review represents, to the best of our knowledge, the first meta-analysis consisting exclusively of RCTs comparing a usual care group with an exercise intervention group. The results demonstrate statistically significant beneficial effects on eGFR through the inclusion of regular exercise as a therapeutic intervention for pre-dialysis CKD. Our comprehensive review encompasses 16 studies with 17 intervention groups, an increase of at least seven studies compared to prior reviews. Previous reviews have yielded disparate findings regarding the impact of exercise on eGFR. While some reports have failed to identify discernible improvements [13, 16, 74, 86], others reported statistically significant enhancements [15, 79, 87, 88]. It is noted that certain prior reviews suffered from flaws such as duplicate data usage [16, 74, 79] and misinterpretation of inclusion criteria [12, 87, 88], which would have inaccurately influenced the results.

After excluding low-quality studies, our analysis consistently demonstrated a positive effect: a slower decline in eGFR for the exercise intervention group compared to the control group (*p* = 0.02). However, leave-one-out sensitivity analysis-removing the study by Deus [19] reduced this positive effect shifting our results from *p* = 0.001 to *p* = 0.06. The modality sub-analysis indicated that resistance training had a significant positive impact on eGFR, however, it should be noted that only two studies utilising resistance training reported eGFR, Deus [19] and de Araujo [18], both of which focused exclusively on stage G2 participants. Our results also revealed a significant impact on eGFR when the intervention was supervised and had a duration of between 12 weeks and 6 months (*p* < 0.001 for both sub-analyses) in favour of the exercise group. While our results indicated a slower decline in the exercise group compared to the control, it is important to consider that individuals with CKD stage G2 typically experience a slower decline in eGFR than those in grades G3 and higher.

Among the other evaluated renal parameters, our analysis highlighted a statistically significant and positive influence of exercise on cystatin-C levels. Although based on a limited inclusion of only four studies, cystatin-C, often favoured over creatinine levels in eGFR calculations [49], serves as a valuable indicator of renal function.

As the primary cause of death for individuals with CKD is more likely to be related to cardiovascular disease rather than renal failure [89], the reduction of cardiovascular risk factors becomes particularly beneficial. Our findings indicate a significant improvement from exercise in four cardiovascular risk outcomes: resting heart rate, triglyceride and glycosylated haemoglobin levels, as well as waist circumference.

Despite doubling the number of included trials compared to the study by Yamamoto [16], we did not observe any statistically significant benefit for blood pressure outcomes, which aligns with the findings of another study [79]. In contrast, Yamamoto reported a significant decrease in SBP. Upon closer examination, it becomes evident that this review [16] included participant numbers more than once for some outcomes, potentially leading to an overestimation of the training effect.

Our analysis revealed a favourable trend towards improvements in endothelial function and lipid and blood parameter outcomes among individuals with pre-dialysis CKD. It is important to consider the potential influence of both blood pressure and other cardiac medications (e.g., statins) since a substantial proportion of participants were reported to be medicated. This medication aspect may potentially mask the changes in blood pressure and heart rate that would typically be expected from the addition of an exercise regimen [90]. Available publication data did not allow for a more in-depth analysis accounting for these factors.

Our results also revealed a promising trend of improvement in BMI with regular exercise. Considering that a high BMI is associated with a worse prognosis in pre-dialysis CKD [91], a reduction in BMI at this pre-dialysis stage of disease may contribute to attenuating the progression of not only CKD but also of common co-morbid conditions such as hypertension and diabetes.

Our analysis demonstrated a significant reduction in IL-6 levels, favouring the exercise group. Additionally, we observed a trend toward reduced CRP levels favouring the exercise group when compared to the control group. As there is a well-established link between elevated CRP and IL-6 levels posing increased risks to both cardiovascular and renal systems [92, 93] these results are encouraging and align with previous reviews [15, 86]. As impaired renal function contributes to accumulation of IL-6, our observed improvement of eGFR may be linked to the improvement in IL-6 levels [94].

It is not currently feasible to prescribe a specific exercise modality, including recommendations for frequency, duration, and intensity of each session as a standardised treatment for CKD stages G1 to pre-dialysis G5. The individual nature of the disease, coupled with variables like socio-economic circumstances, geographical location, comorbidities, medications, and personal exercise preferences, necessitates a tailored approach. However, for individuals living with CKD stages G1 to pre-dialysis G5 and healthcare professionals seeking evidence-based guidance, our findings highlight the effectiveness of both aerobic and combined training in significantly improving various key outcomes including aerobic capacity, functional ability, renal parameters, and quality of life. Despite limited representation in our analysis with only four studies, our results also demonstrate the positive impact of resistance training. It is important to note, however, that ten out of 17 significant findings were based on only one intervention group using this modality within each outcome (online resource 9, Supplemental Notes). Similarly, mind/body training yielded no significant results, which may be attributed to the limited data from only one study using this type of intervention. These findings should not lead to the exclusion of resistance and mind/body training; instead, they underscore the need for further RCTs encompassing all types of training.

The majority of the included studies were based on three intervention sessions per week, although our findings did indicate benefits across all reported frequencies. Overall, intervention duration ranging between 12 weeks and 6 months demonstrated the highest percentage of significant outcomes; however, this varied per outcome.

Our sub-analyses demonstrate that fully supervised intervention sessions produced the most substantial improvements in outcomes. Furthermore, outcomes for participants engaged in initially supervised sessions or a combination of supervised and unsupervised sessions each week were significantly more positive than those in unsupervised sessions alone. This observation aligns with the well-known Hawthorne effect, which posits that individuals living with, for example, CKD, derive more substantial benefits from clinical trials when they are monitored, at least partially [95]. While our review did not undertake a comprehensive cost–benefit analysis of regularly supervised exercise sessions compared to the expense associated with CKD progression leading to dialysis and reduced community involvement, similar models exist for heart disease programs. These programs are available for cardiovascular disease sufferers and suggest a similar approach can be adopted for individuals living with CKD. Based on our results, it seems that one supervised session per week, complemented by continued unsupervised sessions, offer substantial benefits while also representing a cost-effective alternative to the conventional three-session per week approach.

Our sub-analyses based on removal of low-quality studies via TESTEX (our Risk of Bias test did not reveal any low-quality studies) removed the statistical level of significance for some outcomes. It should be noted when considering these changes that the nature of RCTs based on an exercise intervention often make areas included in TESTEX, such as blinding of assessors and monitoring activity of the control group, impractical.

We believe this systematic review to be the most comprehensive examination of exercise as a component of pre-dialysis CKD management to date, with minimal evidence of publication bias. Nevertheless, it is essential to acknowledge several limitations within our review. These limitations encompass the limited number of eligible studies for certain outcomes and the considerable variability in interventions and participant characteristics, and these factors have imposed limitation on our data analysis. Heterogeneity observed in our meta-analysis may be attributed to the limited number of studies, for example in the sit to stand test and two-minute step test, and to the diverse exercise-based programs’ features, such as exercise intensity, duration, frequency, supervision, and modality, as well as participant characteristics, particularly CKD stage. This diversity has contributed to significant heterogeneity in some outcomes, potentially affecting the reliability and interpretability of our findings. Furthermore, our inclusion criteria focused solely on published, peer reviewed RCTs, which inevitably excluded grey literature. This omission may have resulted in the exclusion of current data from unpublished or ongoing trials, potentially introducing bias.

The accuracy in recording participant exercise intensity per session and home exercise regularity remains unknown, and our inclusion criteria also prevented our review from exploring the effect of leisure time physical activity. As exercise volume has been shown to influence life expectancy across a range of activity levels [96], being unable to include these factors also limits our results. We must also recognise the potential for bias during statistical analysis when subtracting the baseline mean from the final mean value, as participants with lower baseline fitness levels may exhibit more pronounced improvements when compared to those who began with a reasonable to high fitness level. This pre-post design change may be susceptible to regression to the mean [97]. Additionally, the reporting of medication use and changes during or after trials was inadequate, potentially influencing our results. To gain further insights into the clinical significance of our statistically significant findings, it would be valuable to compare each outcome’s pooled data to previously established minimum clinically important difference [98]. Regrettably, a paucity of minimum clinically important difference values for analysed outcomes hampers such comparisons. Furthermore, the comparison of minimum clinically important difference would necessitate meta-regression, which was unfeasible due to the limited number of included studies. We believe that future research efforts aimed at establishing minimum clinically important difference values, alongside larger RCTs with extended duration and improved reporting precision, hold potential to provide additional evidence in this field.

## Conclusions

We believe our extensive review presents the most comprehensive meta-analysis of the effect of exercise on health-related outcomes conducted to date, revealing that regular exercise yields substantial improvements in various health parameters for individuals with diagnosed CKD stages G1 to pre-dialysis G5. Notably, exercise enhances aerobic capacity, functional ability, quality of life, and positively impacts estimated glomerular filtration rate, resting heart rate, triglyceride, interleukin-6, serum cystatin-C and glycosylated haemoglobin levels, and waist circumference. Given that these improvements have the potential to delay disease progression and alleviate the long-term burden, it is evident that regular exercise as a therapy holds benefits for individuals with pre-dialysis CKD.

## Supplementary Information

Below is the link to the electronic supplementary material.

Supplementary file1 (DOCX 810 KB)

Supplementary file2 (DOCX 778 KB)

Supplementary file3 (DOCX 1208 KB)

Supplementary file4 (DOCX 513 KB)

Supplementary file5 (DOCX 15 KB)

Supplementary file6 (DOCX 217 KB)

Supplementary file7 (DOCX 104 KB)

Supplementary file8 (DOCX 47 KB)

Supplementary file9 (DOCX 635 KB)

## Data Availability

Data for this analysis is available from the corresponding author.

## Abbreviations

OR: Online Resource
ST: Supplemental Table
SF: Supplemental Figure
