# Supplementary material for: The effect of exercise training in people with pre-dialysis chronic kidney disease: a systematic review with meta-analysis
Source: J Nephrol. 2024 Oct 17;37(8):2063–98. doi: 10.1007/s40620-024-02081-9 (PMC11649798; doi:10.1007/s40620-024-02081-9)
Supplement: Supplementary file 1 — Supplementary file1 (DOCX 810 KB) [file 40620_2024_2081_MOESM1_ESM.docx]

**The effect of exercise training in people with pre-dialysis chronic kidney disease. A systematic review with meta-analysis.**

Annette Traise*, Gudrun Dieberg, Melissa J Pearson, Neil A Smart

Clinical Exercise Physiology, School of Science and Technology, University of New England, NSW 2351, Australia

* Corresponding author

**Online Resource 3**

**Supplemental** **material: Figures 1 – 9: Egger Funnel Plot of Comparison (pseudo 95% confidence interval) of outcomes**

**Supplemental Figure 1** Aerobic capacity funnel plot of comparison

**Supplemental Figure 2** Functional ability funnel plot of comparison

**Supplemental Figure 3** Quality of Life

**Supplemental Figure 4** Renal parameters funnel plot of comparison

**Supplemental Figure 5** Cardiovascular Risk Factors: Blood Pressure

**Supplemental Figure 6** Cardiovascular Risk Factors: Endothelial factors

**Supplemental Figure 7** Cardiovascular Risk Factors: Lipids and blood parameters

**Supplemental Figure 8** Cardiovascular Risk Factors: Body composition parameters

**Supplemental Figure 9** Inflammatory markers

**Supplemental Figure 1** Aerobic capacity funnel plot of comparison

**SF1a**: Peak VO2 (mL/kg/min) (by intervention modality); **SF1b:** Peak VO2 (mL/kg/min) (by CKD stage); **SF1c**: Peak respiratory ratio (by intervention modality); **SF1d**: Peak respiratory ratio (by CKD stage)

| **SF1a:** Peak VO_2_ (mL/kg/min) p<0.00001**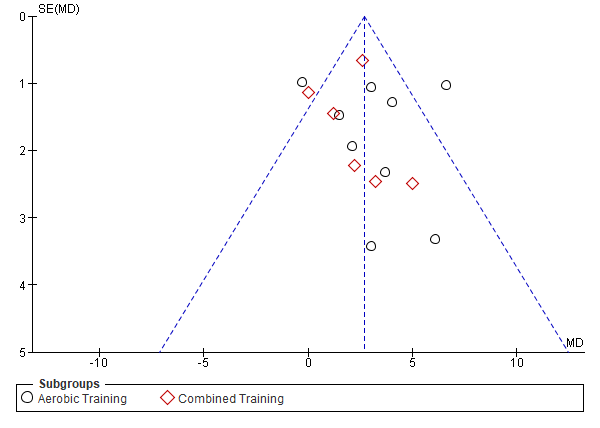** | **SF1b:** Peak VO_2_ (mL/kg/min) p<0.00001  **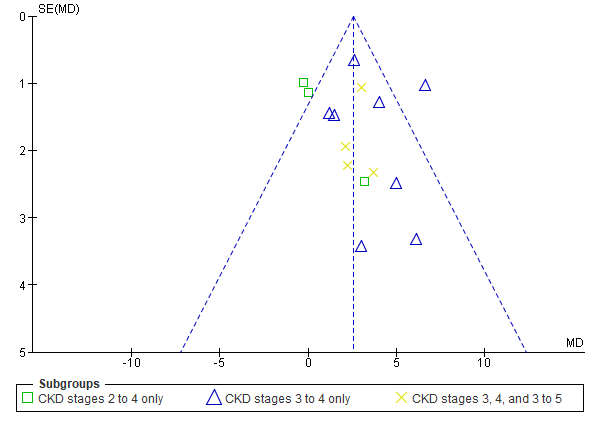** |
| --- | --- |
| **SF1c**: Peak respiratory ratio p=0.44  **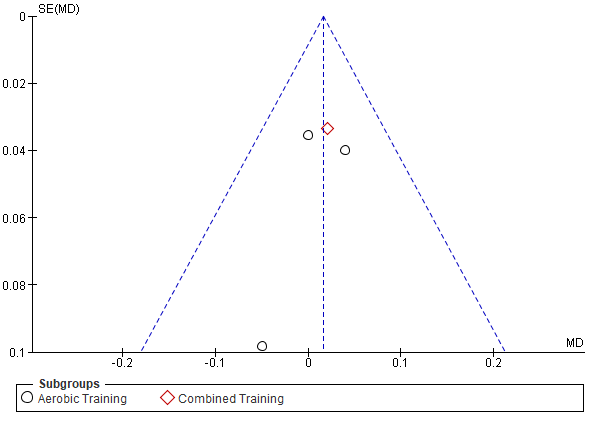** | **SF1d**: Peak respiratory ratio p=0.44  **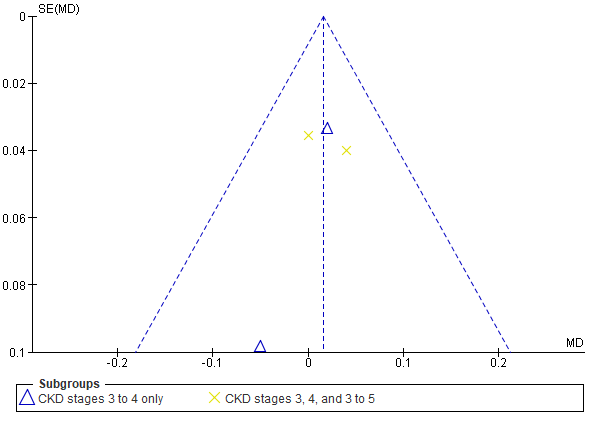** |

**Supplemental Figure 2** Functional ability funnel plot of comparison

**SF2a**: Six-minute walk distance test (m) (by intervention modality); **SF2b:** Six-minute walk distance test (m) (by CKD stage); **SF2c**: Timed up and go test (by intervention modality); **SF2d**: Timed up and go test (by CKD stage); **SF2e**: Two-minute step test (by intervention modality); **SF2f**: Two-minute step test (by CKD stage); **SF2g**: Sit to stand test (by intervention modality); **SF2h**: Sit to stand test (by CKD stage); **SF2i**: Handgrip strength (kg) (by intervention modality); **SF2j**: Handgrip strength (kg) (by CKD stage)

| **SF2a:** 6MWT (m) p<0.00001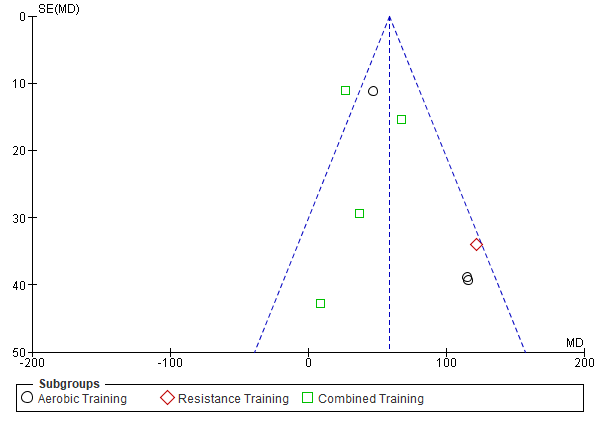 | **SF2b:** 6MWT (m) p<0.00001  **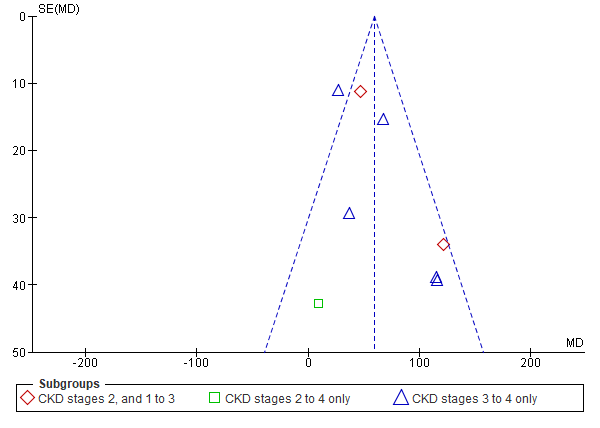** |
| --- | --- |
| **SF2c**: Timed up and go test (3m and return) p=0.0006  **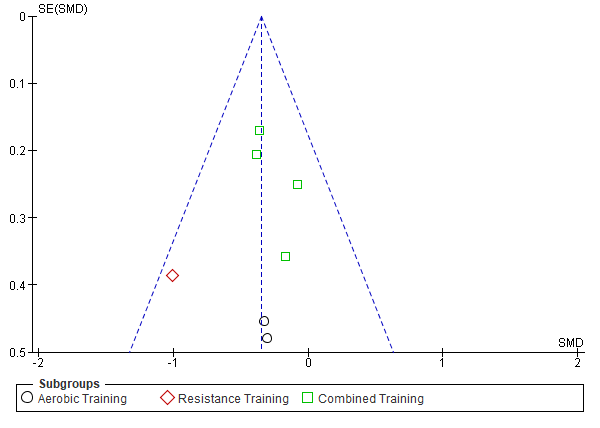** | **SF2d**: Timed up and go test (3m and return) p=0.0006  **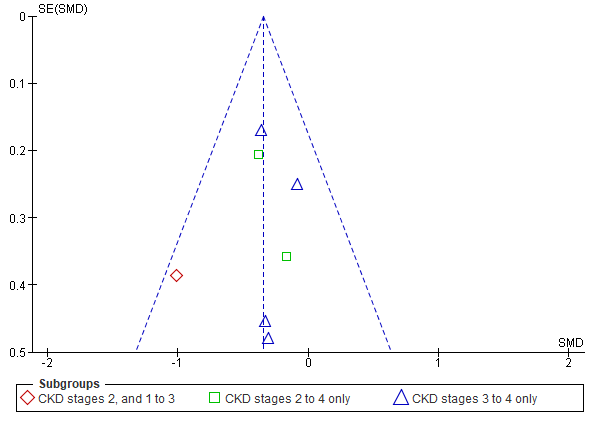** |
| **SF2e**: Two-minute step test (no of steps) p=0.0001**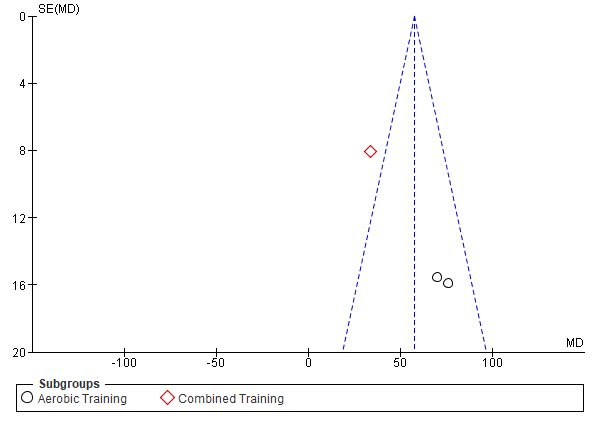** | **SF2f**: Two-minute step test (no of steps) p=0.0001  **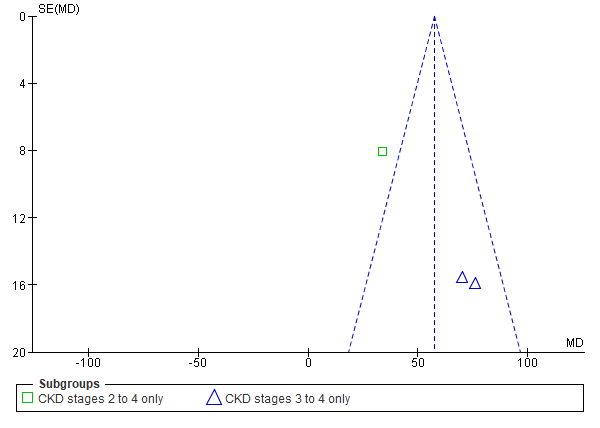** |
| **SF2g**: Sit to stand test (reps in 30sec) p=0.004**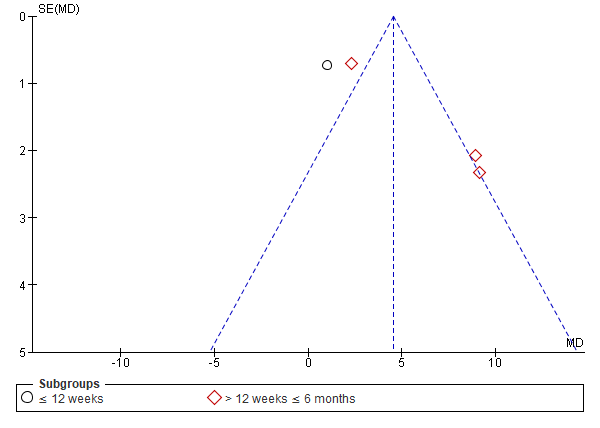** | **SF2h**: Sit to stand test (reps in 30sec) p=0.004  **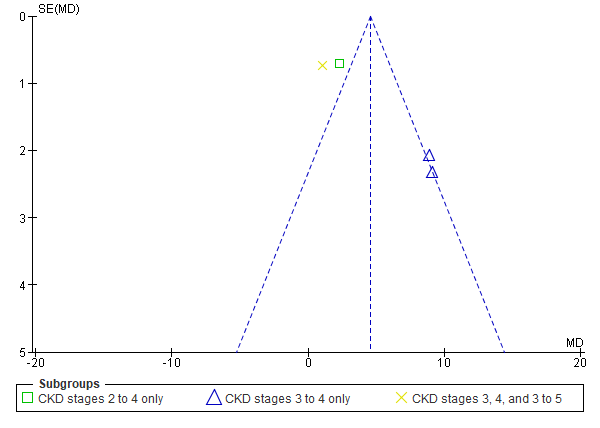** |
| **SF2i**: Handgrip strength (kg) p=0.13  **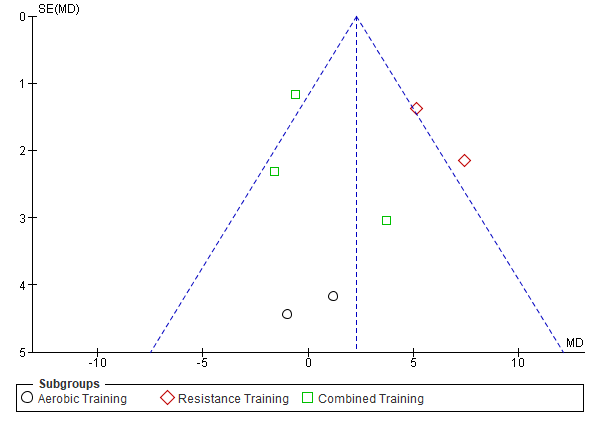** | **SF2j**: Handgrip strength (kg) p=0.13  **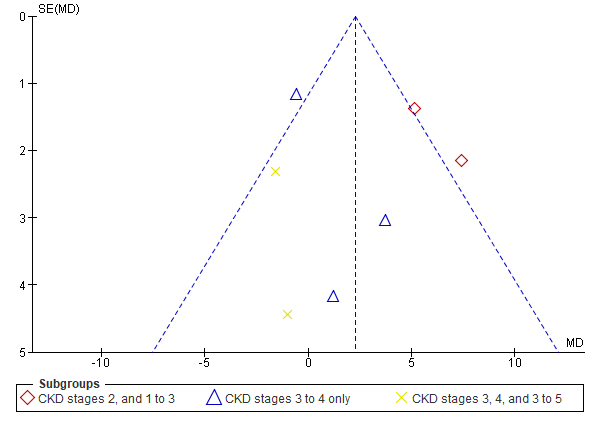** |

**Supplemental Figure 3** Quality of Life

**SF3a:** General Health (by intervention modality); **SF3b:** General Health (by CKD stage); **SF3c:** Mental Component Summary (by intervention modality); **SF3d:** Mental Component Summary (by CKD stage); **SF3e:** Physical Component Summary (by intervention modality); **SF3f:** Physical Component Summary (by CKD stage).

| **SF3a:** General Health p=0.05  **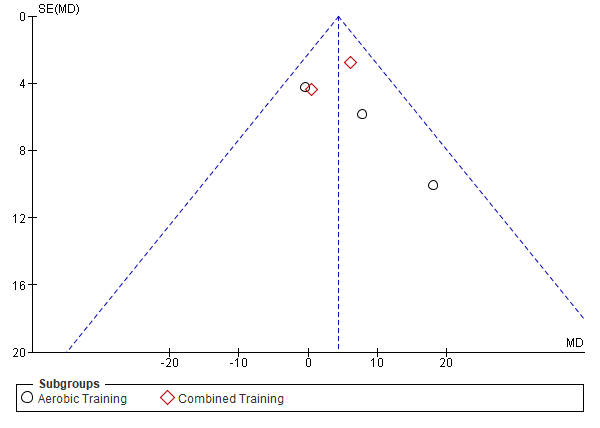** | **SF3b:** General Health p=0.05  **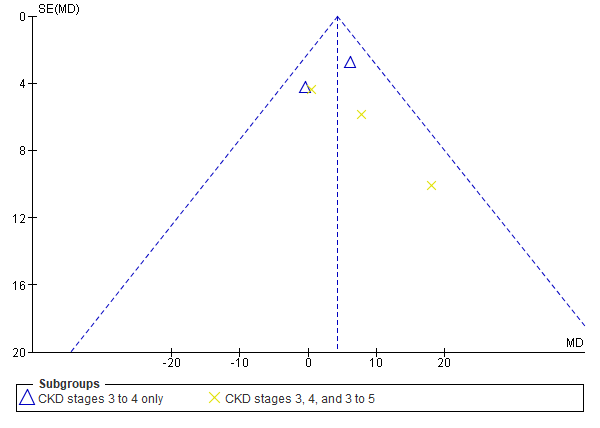** |
| --- | --- |
| **SF3c:** Mental Component Summary p=0.03  **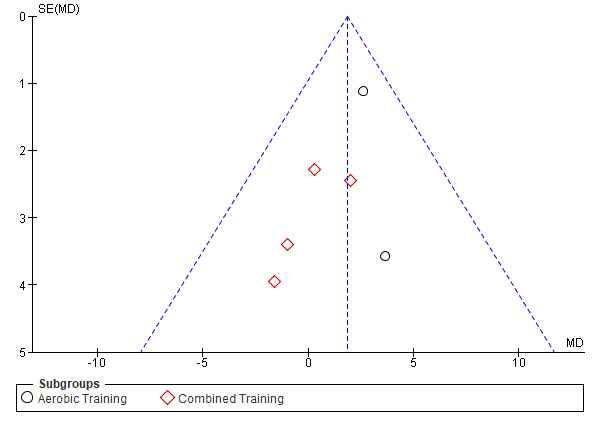** | **SF3d:** Mental Component Summary  **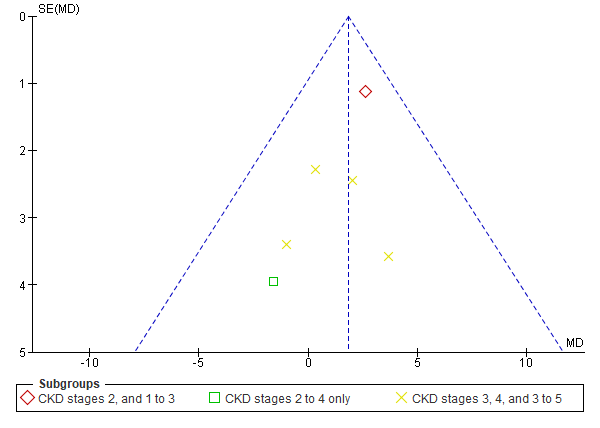** |
| **SF3e:** Physical Component Summary p=0.22  **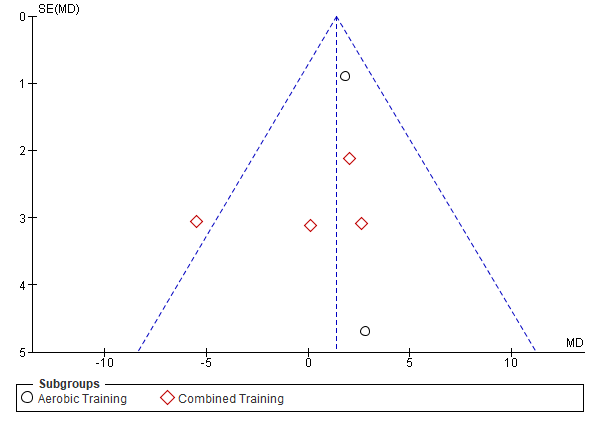** | **SF3f:** Physical Component Summary p=0.22  **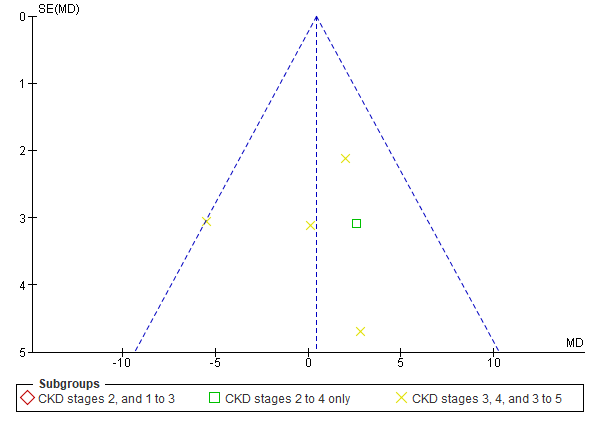** |

**Supplemental Figure 4** Renal parameters funnel plot of comparison

**SF4a**: Estimated glomerular filtration rate (mL/min/1.73m2) (by intervention modality); **SF4b:** Estimated glomerular filtration rate (mL/min/1.73m2) (by CKD stage); **SF4c:** Estimated glomerular filtration rate cystatin-C (mL/min/1.73m2) (by intervention modality); **SF4d:** Estimated glomerular filtration rate cystatin-C (mL/min/1.73m2) (by CKD stage); **SF4e:** Serum creatinine (mg/dL) (by intervention modality); **SF4f:** Serum creatinine (mg/dL) (by CKD stage); **SF4g:** Serum albumin (g/dL) (by intervention modality); **SF4h:** Serum albumin (g/dL) (by CKD stage); **SF4i:** Serum cystatin-C (mg/L) (by intervention modality); **SF4j:** Serum cystatin-C (mg/L) (by CKD stage); **SF4k:** Urine albumin-creatinine ratio (g/gCr) (by intervention modality); **SF4l:** Urine albumin-creatinine ratio (g/gCr) (by CKD stage); **SF4m:** Urine protein-creatinine ratio (g/gCr) (by intervention modality); **SF4n:** Urine protein-creatinine ratio (g/gCr) (by CKD stage); **SF4o:** 24 hour Urine Protein (g/24hr) (by intervention modality); **SF4p:** 24 hour Urine Protein (g/24hr) (by CKD stage); **SF4q:** Blood urea nitrogen (mg/dL) (by intervention modality); **SF4r:** Blood urea nitrogen (mg/dL) (by CKD stage)

| **SF4a:** Estimated glomerular filtration rate (mL/min/1.73m^2^) p=0.001  **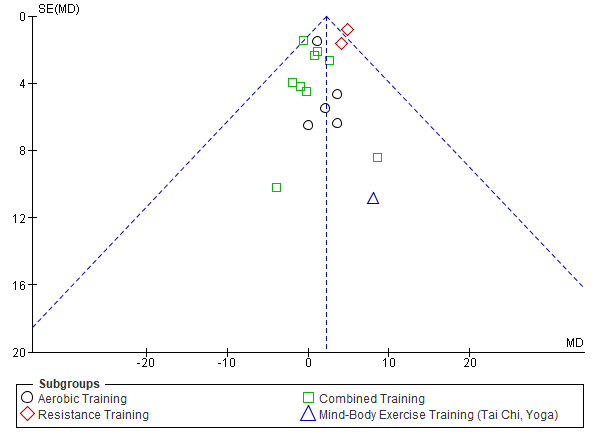** | **SF4b:** Estimated glomerular filtration rate (mL/min/1.73m^2^) p=0.001  **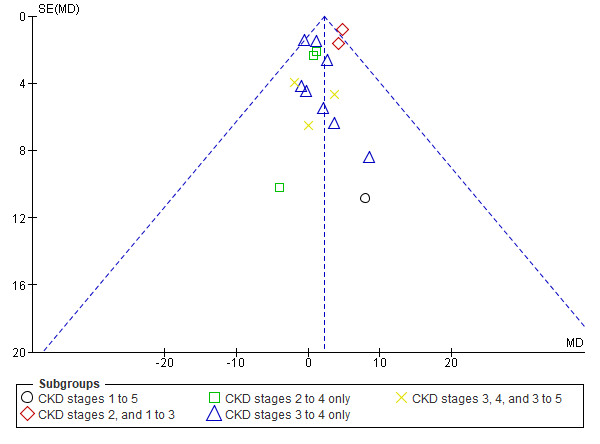** |
| --- | --- |
| **SF4c:** Estimated glomerular filtration rate cystatin-C (mL/min/1.73m^2^) p=0.15**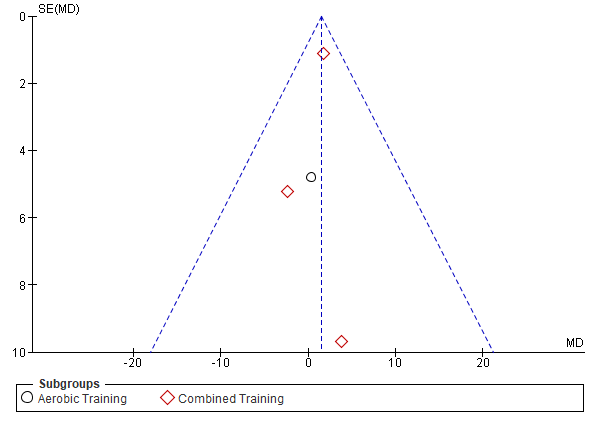** | **SF4d:** Estimated glomerular filtration rate cystatin-C (mL/min/1.73m^2^) p=0.15  **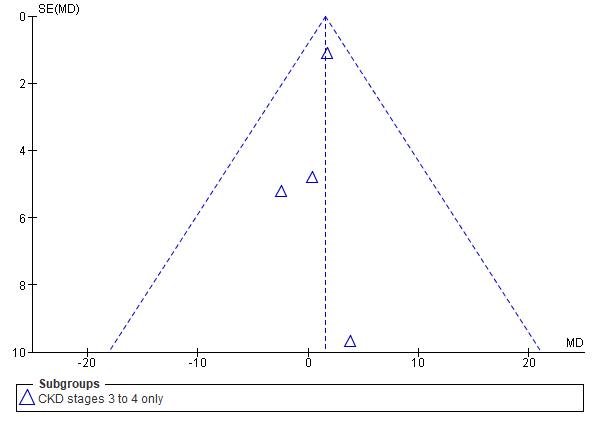** |
| **SF4e:** Serum creatinine (mg/dL) p=0.39**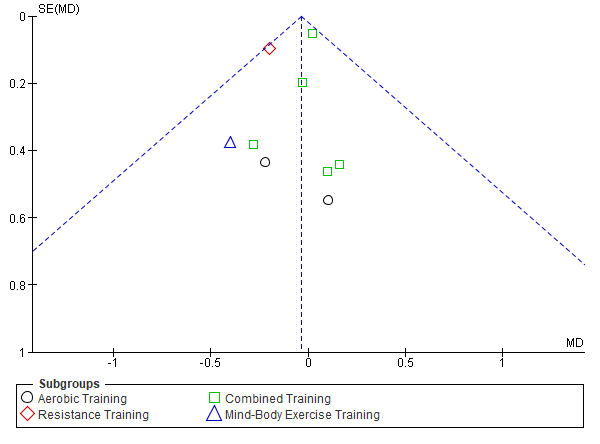** | **SF4f:** Serum creatinine (mg/dL) p=0.39  **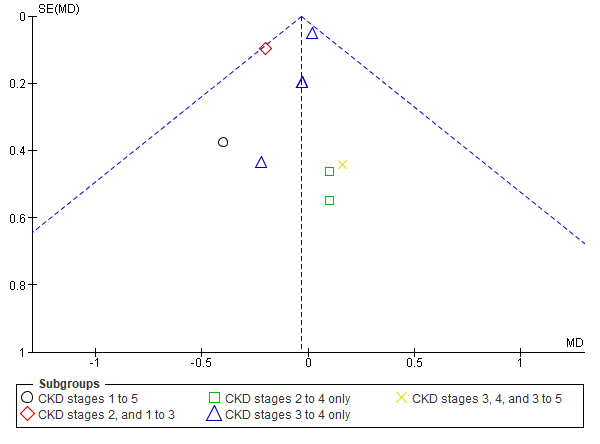** |
| **SF4g:** Serum Albumin (g/dL) p=0.16  **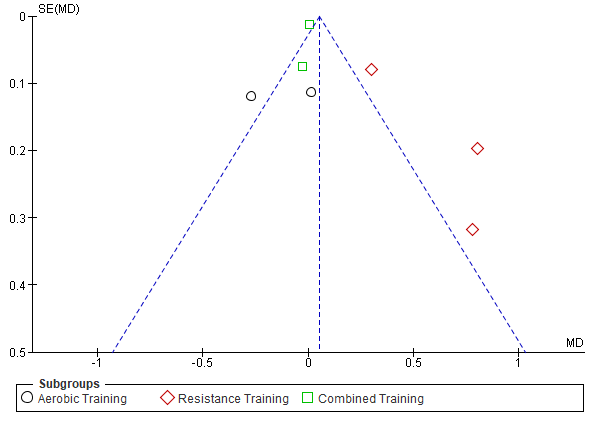** | **SF4h:** Serum Albumin (g/dL) p=0.16  **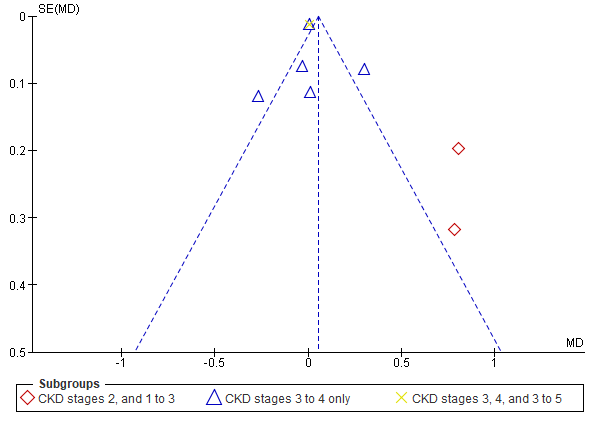** |
| **SF4i:** Serum cystatin-C (mg/L) p=0.004  **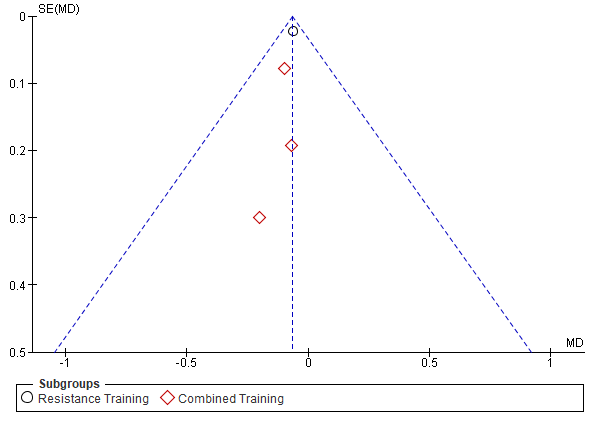** | **SF4j:** Serum cystatin-C (mg/L) p=0.004  **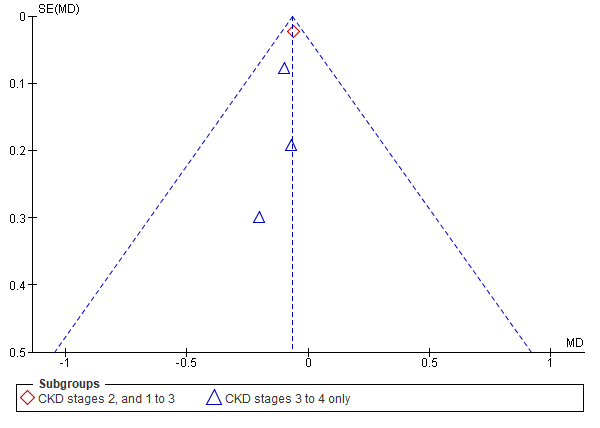** |
| **SF4k:** Urine albumin-creatinine ratio (g/gCr) p=0.90  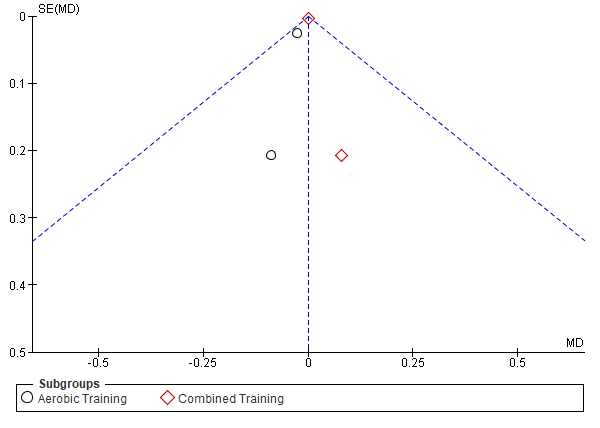 | **SF4l:** Urine albumin-creatinine ratio (g/gCr) p=0.90  **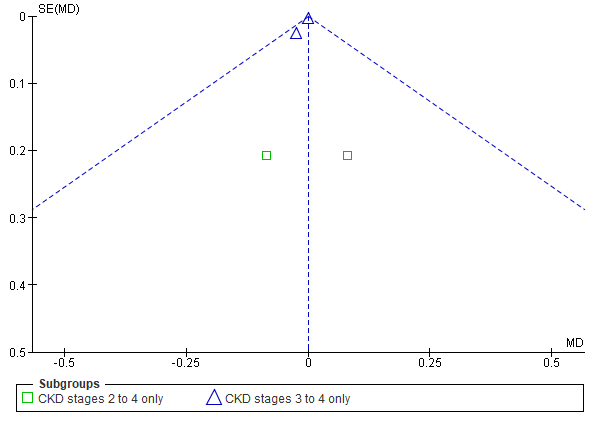** |
| **SF4m:** Urine protein-creatinine ratio (g/gCr) p=0.92**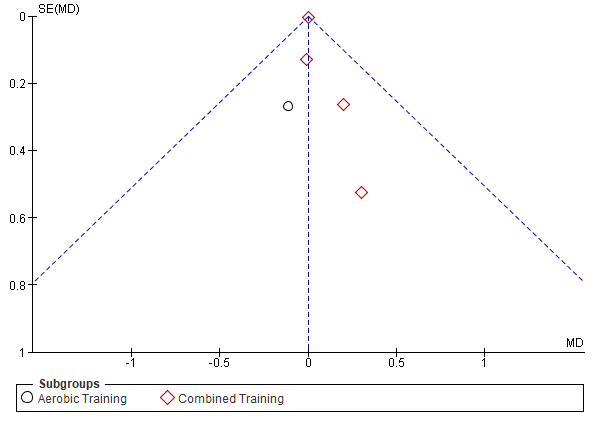** | **SF4n:** Urine protein-creatinine ratio (g/gCr) p=0.92  **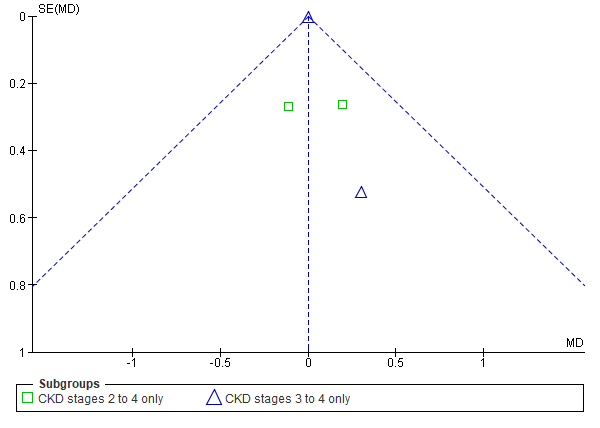** |
| **SF4o:** 24 hour Urine Protein (g/24hr) p=0.79  **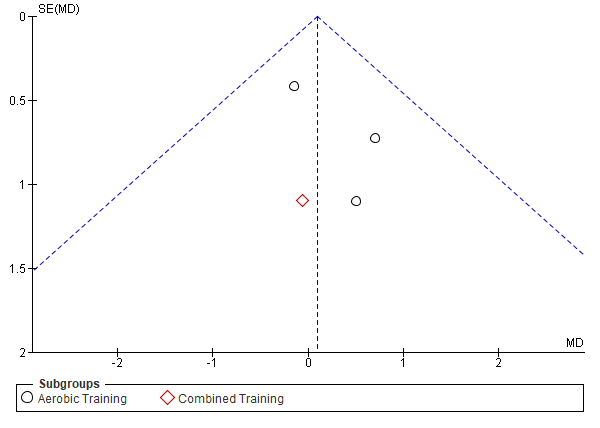** | **SF4p:** 24 hour Urine Protein (g/24hr) p=0.79  **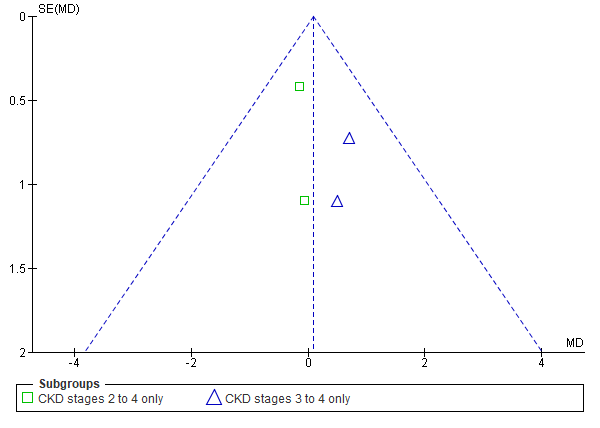** |
| **SF4q:** Blood urea nitrogen (mg/dL) p=0.82  **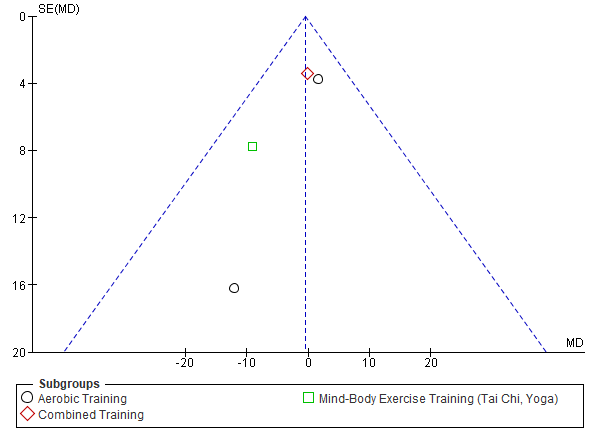** | **SF4r:** Blood urea nitrogen (mg/dL) p=0.82  **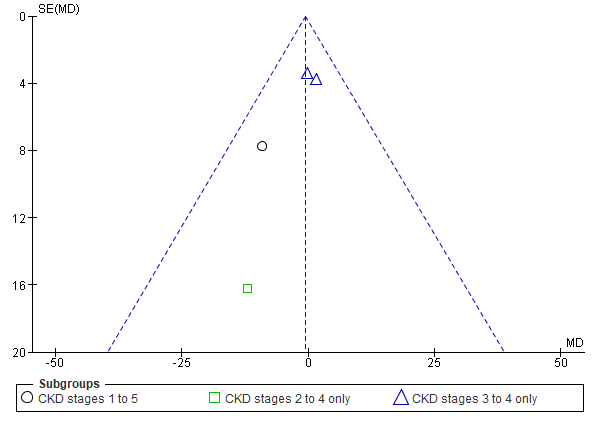** |

**Supplemental Figure 5** Cardiovascular Risk Factors: Blood Pressure

**SF5a:** Resting Heart Rate (by intervention modality); **SF5b:** Resting Heart Rate (by CKD stage); **SF5c:** Systolic Blood Pressure (by intervention modality); **SF5d:** Systolic Blood Pressure (by CKD stage); **SF5e:** Diastolic Blood Pressure (by intervention modality); **SF5f:** Diastolic Blood Pressure (by CKD stage); **SF5g:** Ambulatory 24 hour systolic blood pressure (mmHg) (by intervention modality); **SF5h:** Ambulatory 24 hour systolic blood pressure (mmHg) (by CKD stage); **SF5i:** Ambulatory 24 hour diastolic blood pressure (mmHg) (by intervention modality); **SF5j:** Ambulatory 24 hour diastolic blood pressure (mmHg) (by CKD stage)

| **SF5a:** Resting Heart Rate (bpm) p=0.04  **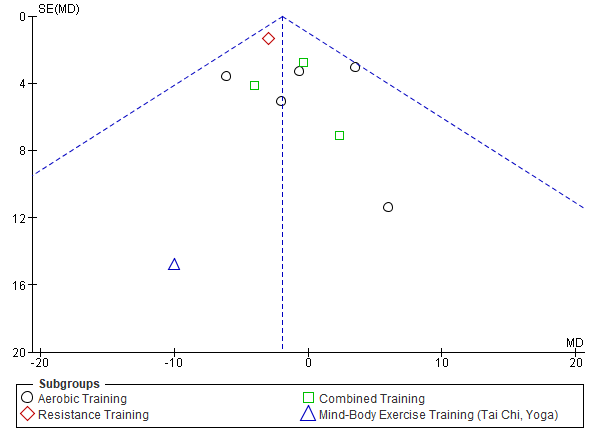** | **SF5b:** Resting Heart Rate (by CKD stage) (bpm) p=0.04  **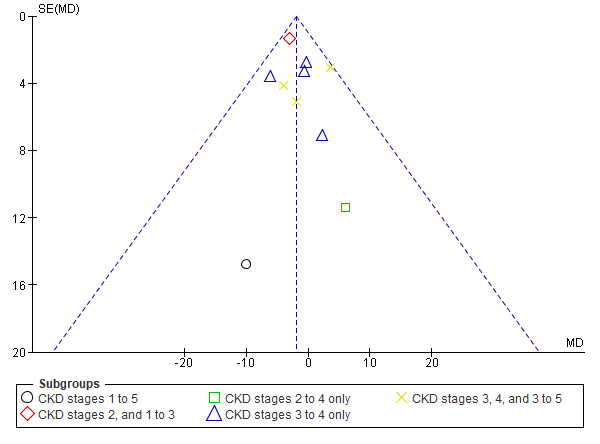** |
| --- | --- |
| **SF5d:** Systolic Blood Pressure (mmHg) p=0.35  **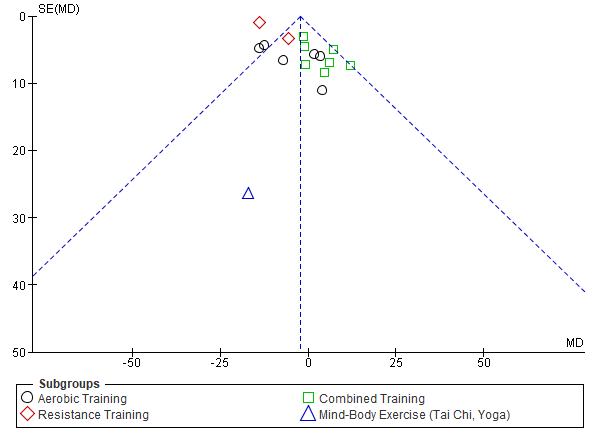** | **SF5d:** Systolic Blood Pressure (mmHg) p=0.35  **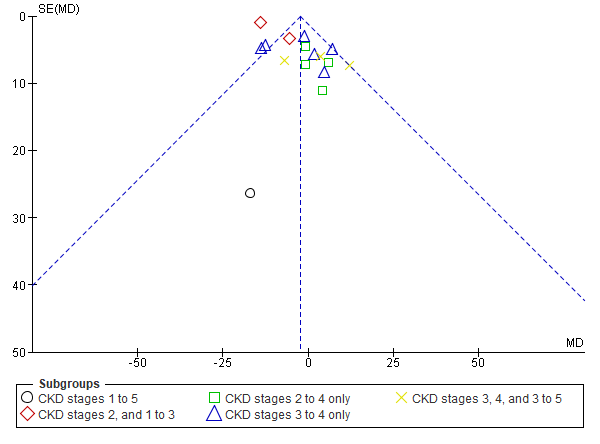** |
| **SF5e:** Diastolic Blood Pressure (mmHg) p=0.32  **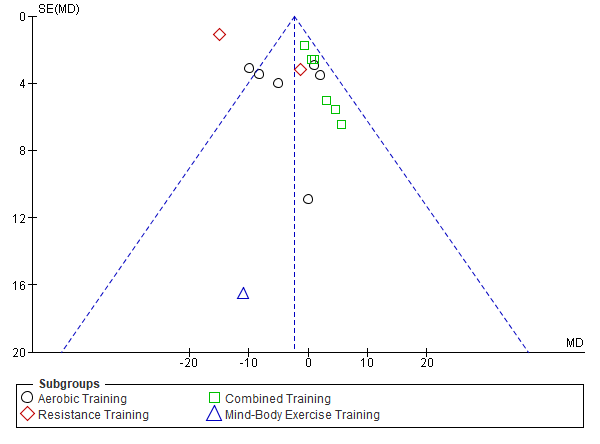** | **SF5f:** Diastolic Blood Pressure (mmHg) p=0.32  **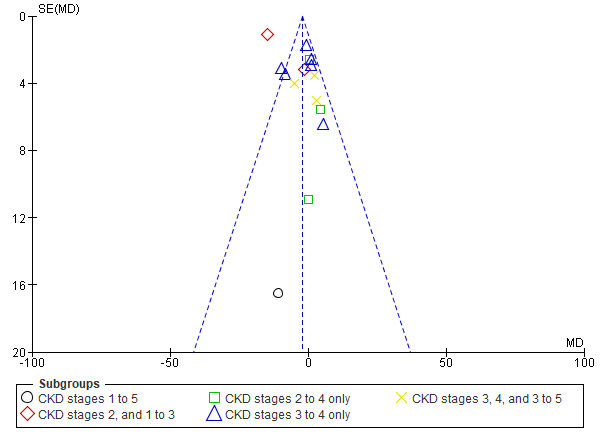** |
| **SF5g:** Ambulatory 24 hour systolic blood pressure (mmHg) p=0.97  **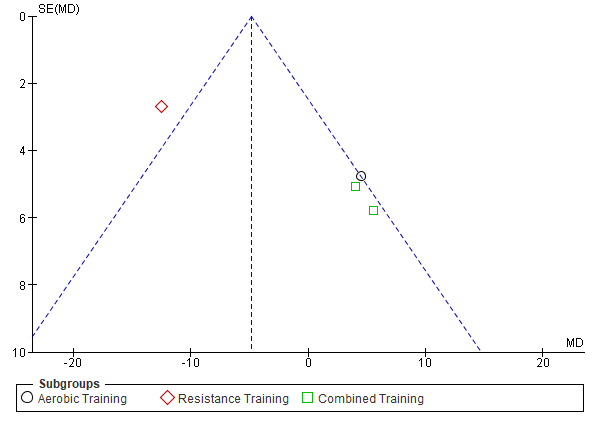** | **SF5h:** Ambulatory 24 hour systolic blood pressure  (mmHg) p=0.97  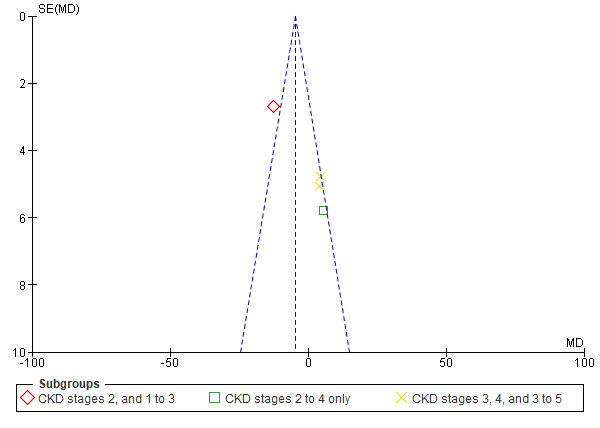 |
| **SF5i:** Ambulatory 24 hour diastolic blood pressure (mmHg) p=0.83  **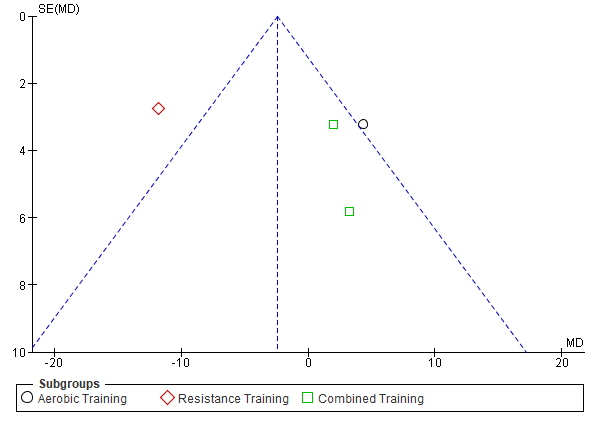** | **SF5j:** Ambulatory 24 hour diastolic blood pressure  (mmHg) p=0.83  **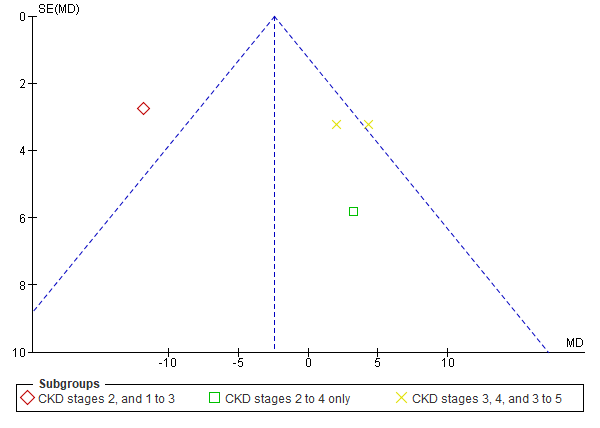** |

**Supplemental Figure 6** Cardiovascular Risk Factors: Endothelial factors

**SF6a:** Pulse Wave Velocity (m/s) (by intervention modality); **SF6b:** Pulse Wave Velocity (m/s) (by CKD stage); **SF6c:** Augmentation Index central arterial (%) (by intervention modality); **SF6d:** Augmentation Index central arterial (%) (by CKD stage); **SF6e:** Asymmetric dimethylarginine (*u*mol/L) (by intervention modality); **SF6f:** Asymmetric dimethylarginine (*u*mol/L) (by CKD stage)

| **SF6a:** Pulse Wave Velocity (m/s) p=0.80  **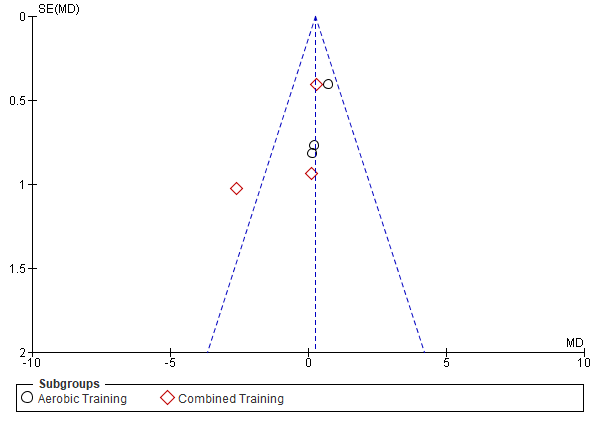** | **SF6b:** Pulse Wave Velocity (m/s) p=0.80  **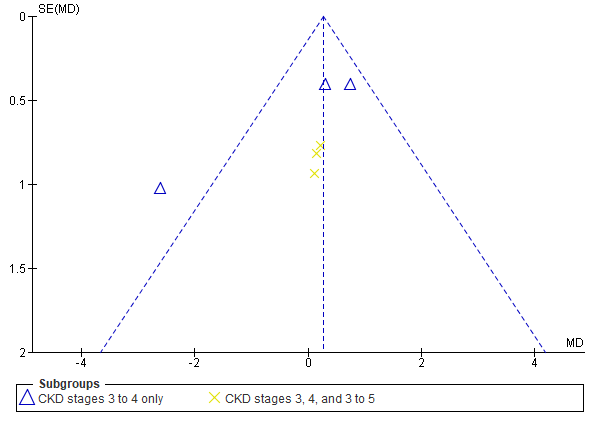** |
| --- | --- |
| **SF6c:** Augmentation Index central arterial (%) p=0.25  **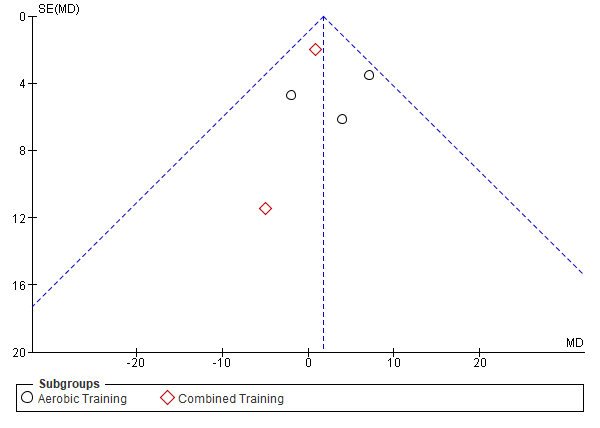** | **SF6d:** Augmentation Index central arterial (%) p=0.25  **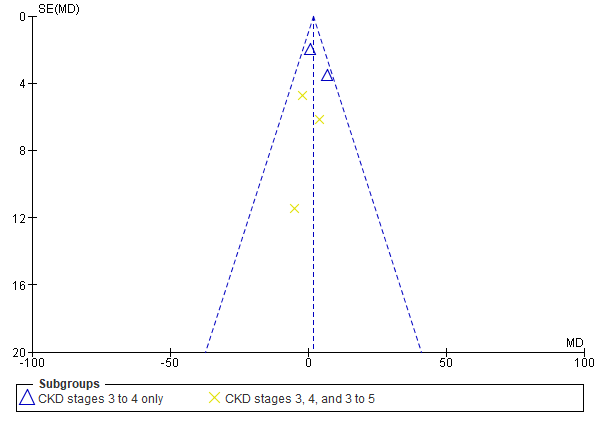** |
| **SF6e:** Asymmetric dimethylarginine (*u*mol/L) p=0.07  **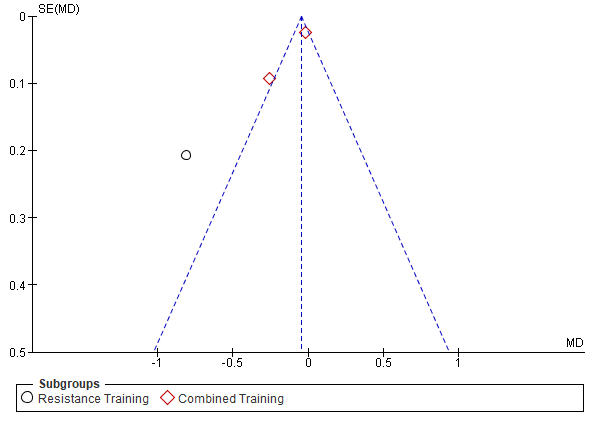** | **SF6f:** Asymmetric dimethylarginine (*u*mol/L) p=0.07  **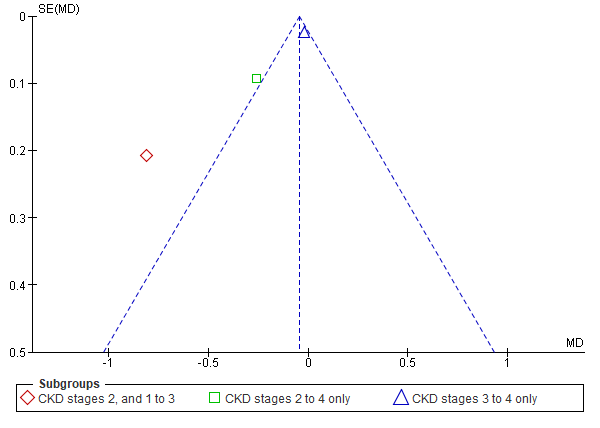** |

**Supplemental Figure 7** Cardiovascular Risk Factors: Lipids and blood parameters

**SF7a:** Triglyceride (by intervention modality); **SF7b:** Triglyceride (by CKD stage); **SF7c:** Total Cholesterol (by intervention modality); **SF7d:** Total Cholesterol (by CKD stage); **SF7e:** Low Density Lipoprotein (by intervention modality); **SF7f:** Low Density Lipoprotein (by CKD stage); **SF7g:** High Density Lipoprotein (by intervention modality); **SF7h:** High Density Lipoprotein (by CKD stage); **SF7i:** Glycosylated Haemoglobin (by intervention modality); **SF7j:** Glycosylated Haemoglobin (by CKD stage); **SF7k:** Blood Glucose (mg/dL) (by intervention modality); **SF7l:** Blood Glucose (mg/dL) (by CKD stage); **SF7m:** Haemoglobin (g/dL) (by intervention modality); **SF7n:** Haemoglobin (g/dL) (by CKD stage)

| **SF7a:** Triglyceride (mg/dL) p<0.00001  **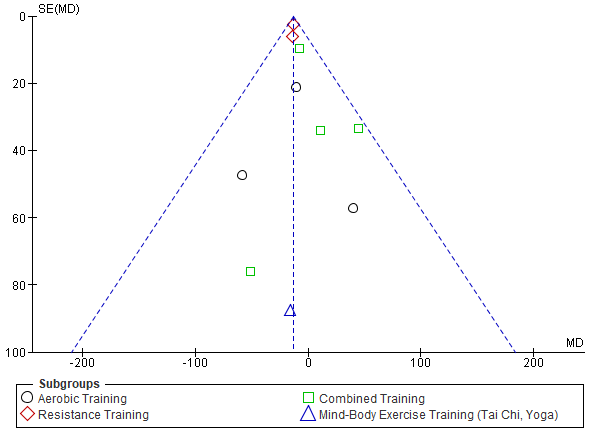** | **SF7b:** Triglyceride (mg/dL) p<0.00001  **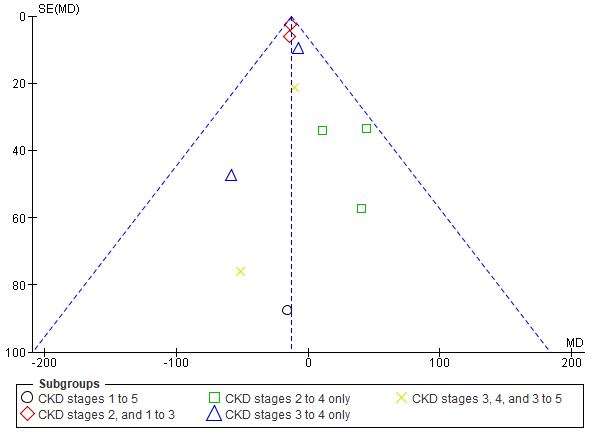** |
| --- | --- |
| **SF7c:** Total Cholesterol (mg/dL) p=0.20  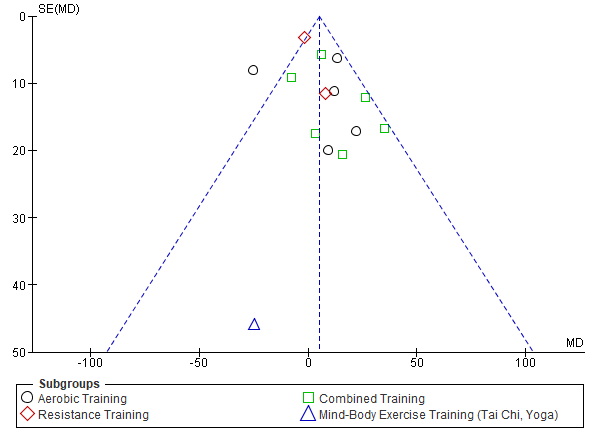 | **SR6d:** Total Cholesterol (mg/dL) p=0.20  **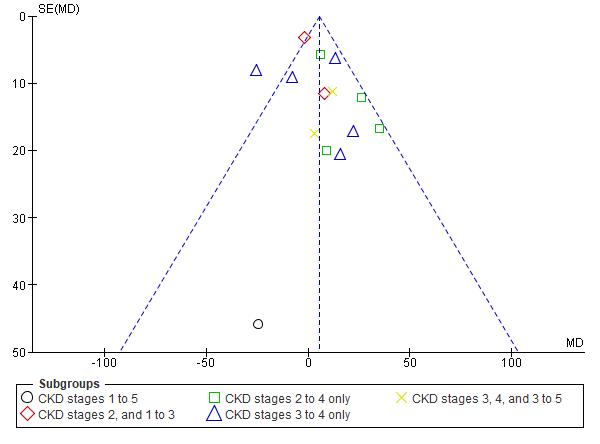** |
| **SF7e:** Low Density Lipoprotein(mg/dL) p=0.06  **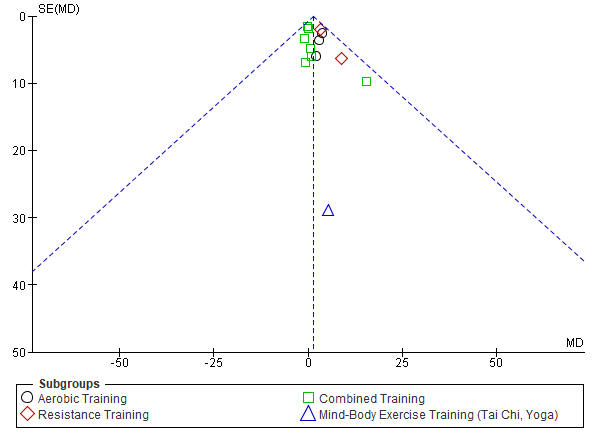** | **SF7f:** Low Density Lipoprotein (mg/dL) p=0.06  **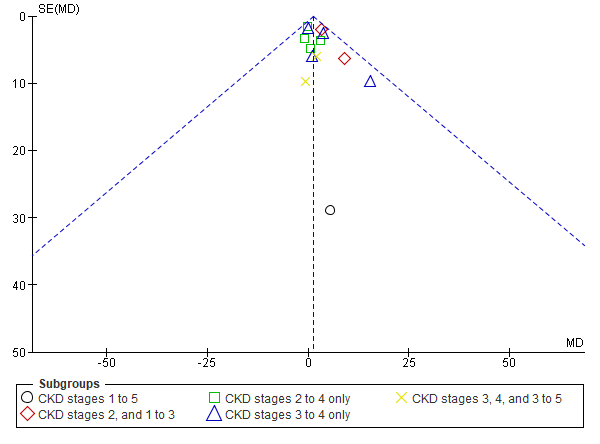** |
| **SF7g:** High Density Lipoprotein (mg/dL) p=0.11  **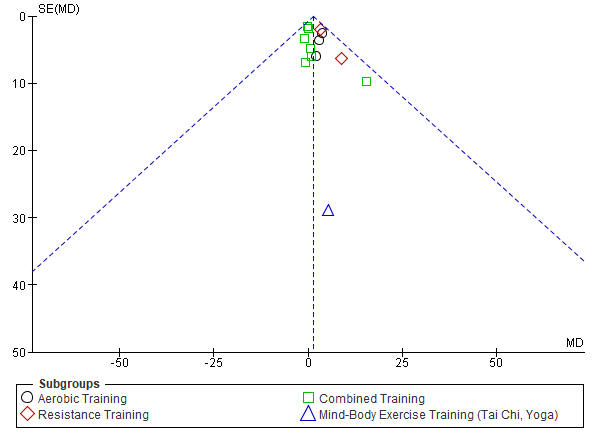** | **SF7h:** High Density Lipoprotein (mg/dL) p=0.11  **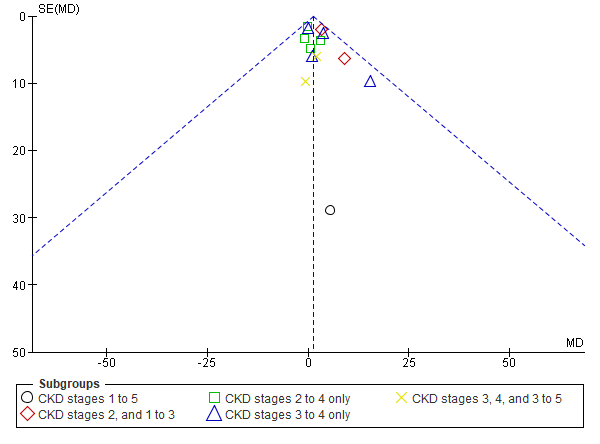** |
| **SF7i:** Glycosylated Haemoglobin (%) p=0.04  **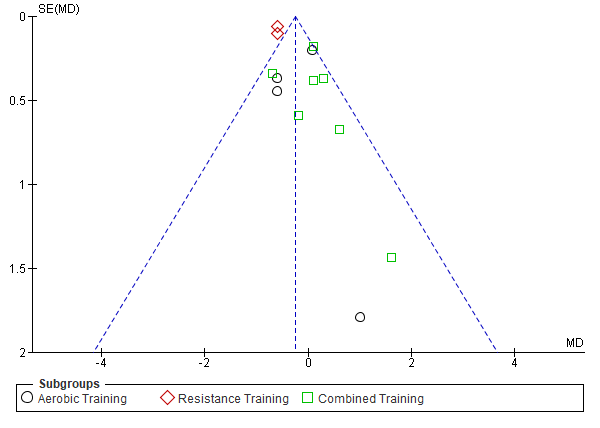** | **SF7j:** Glycosylated Haemoglobin (%) p=0.04  **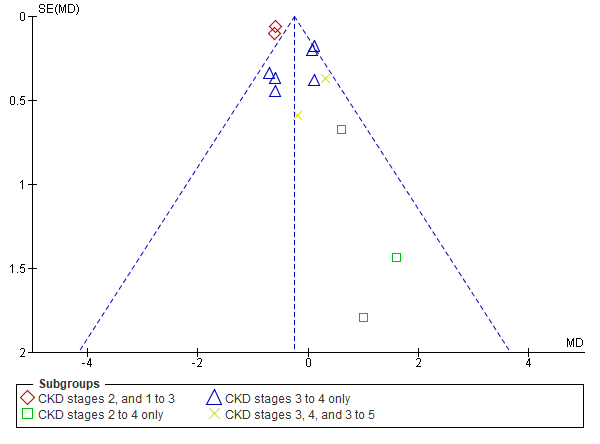** |
| **SF7k:** Blood Glucose (mg/dL) p=0.29  **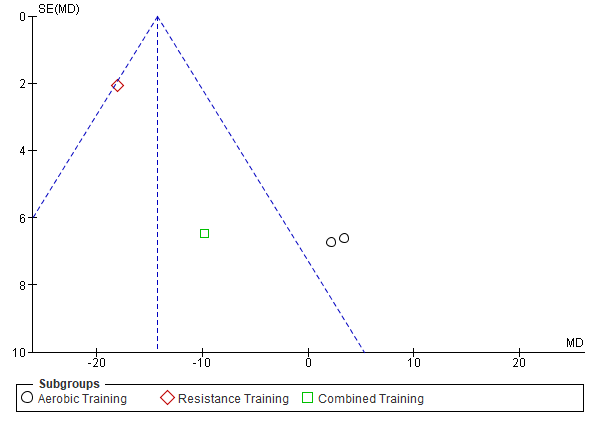** | **SF7l:** Blood Glucose (mg/dL) p=0.29  **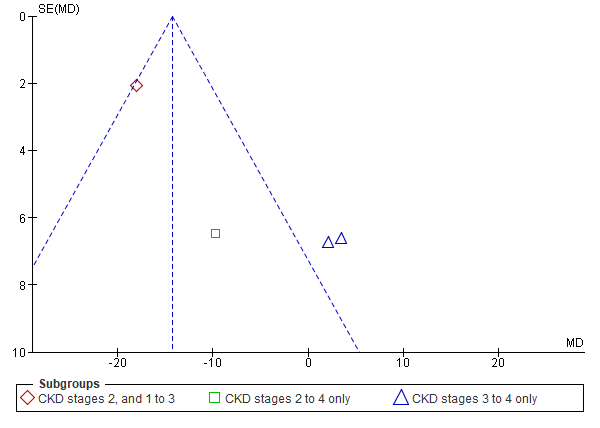** |
| **SF7m:** Haemoglobin (g/dL) p=0.08  **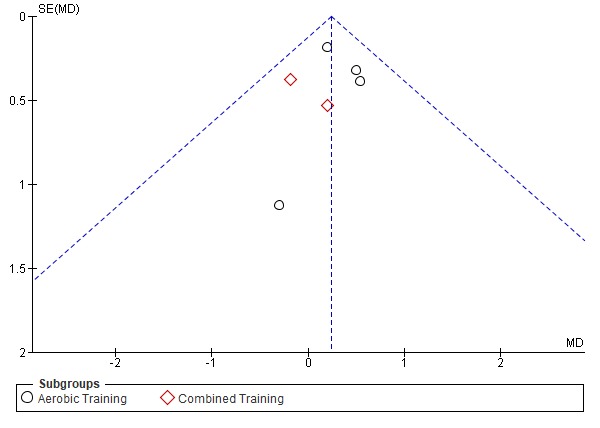** | **SF7n:** Haemoglobin (g/dL) p=0.08  **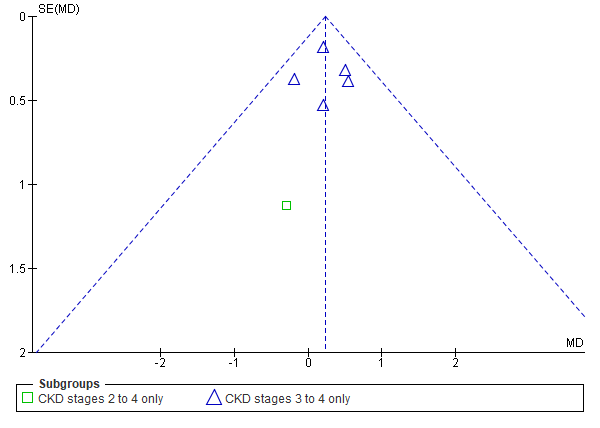** |

**Supplemental Figure 8** Cardiovascular Risk Factors: Body composition parameters

**SF8a:** Waist circumference (cm) (by intervention modality); **SF8b:** Waist circumference (cm) (by CKD stage); **SF8c:** Body Weight (by intervention modality); **SF8d:** Body Weight (by CKD stage); **SF8e:** Body Mass Index (by intervention modality); **SF8f:** Body Mass Index (by CKD stage); **SF8g:** Body Fat (%) (by intervention modality); **SF8h:** Body Fat (%)(by CKD stage);**SF8i:** Lean body mass (kg) (by intervention modality); **SF8l:** Lean body mass (kg) (by CKD stage)

| **SF8a:** Waist circumference (cm) p<0.00001  **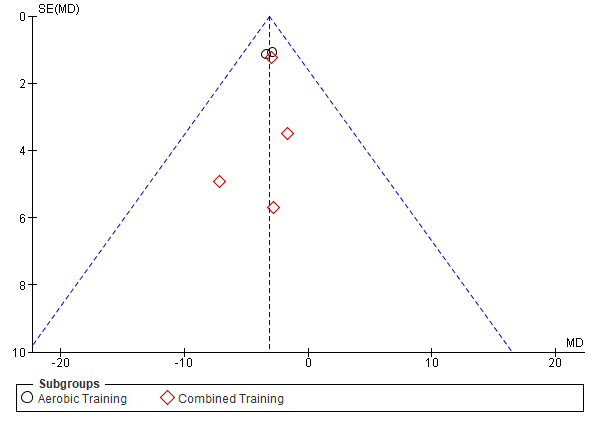** | **SF8b:** Waist circumference (cm) p<0.00001**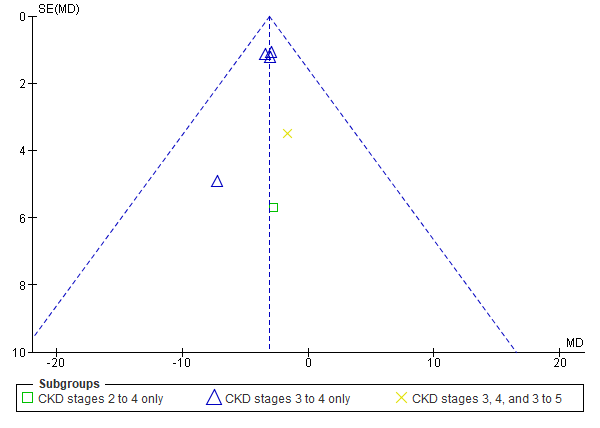** |
| --- | --- |
| **SF8c:** Body Weight (kg) p=0.62  **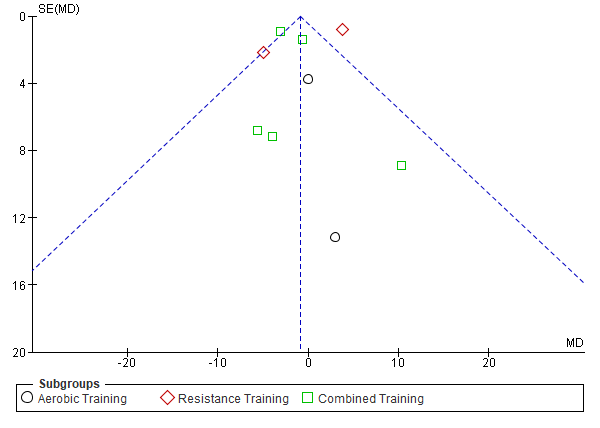** | **SF8d:** Body Weight (kg) p=0.62  **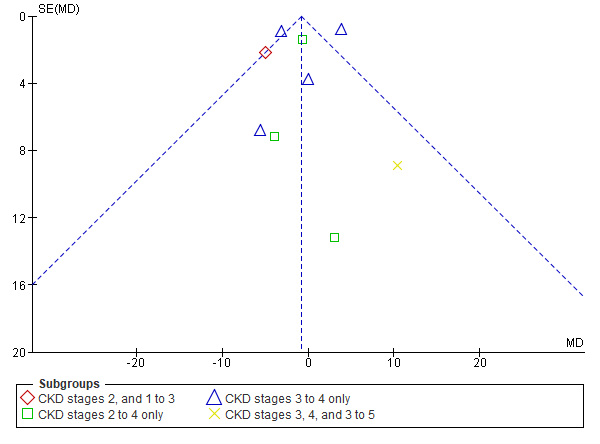** |
| **SF8d:** Body Mass Index (kg/m^2^) p=0.18  **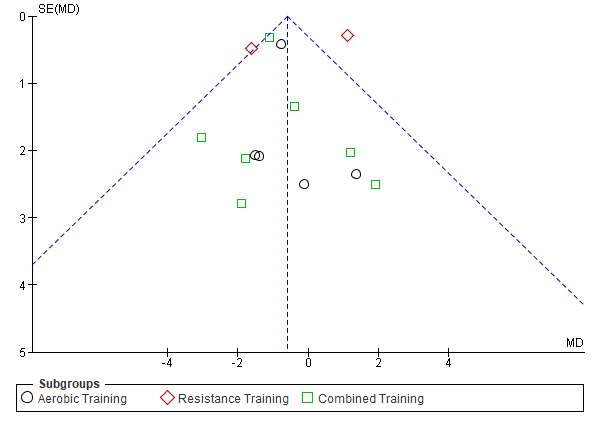** | **SF8f:** Body Mass Index (kg/m^2^) p=0.18  **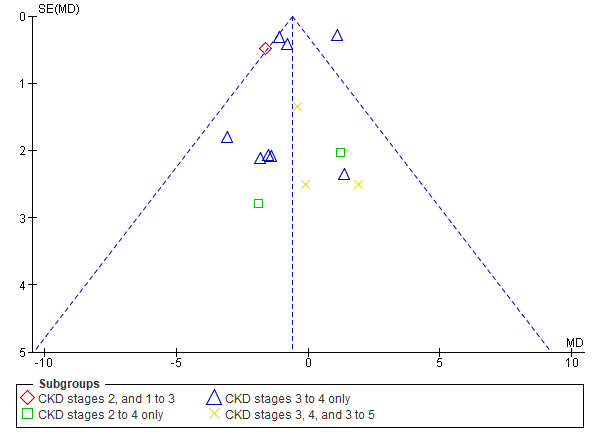** |
| **SF8g:** Body Fat (%) p=0.09  **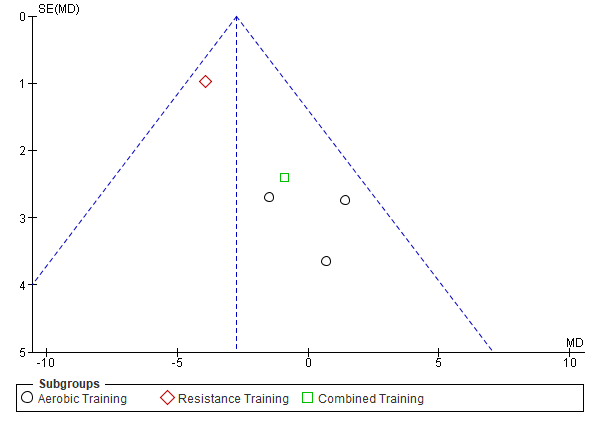** | **SF8h:** Body Fat (%) p=0.09  **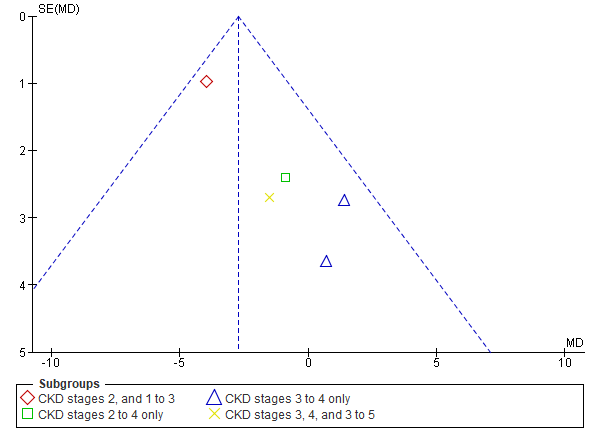** |
| **SF8i:** Lean body mass (kg) p=0.06  **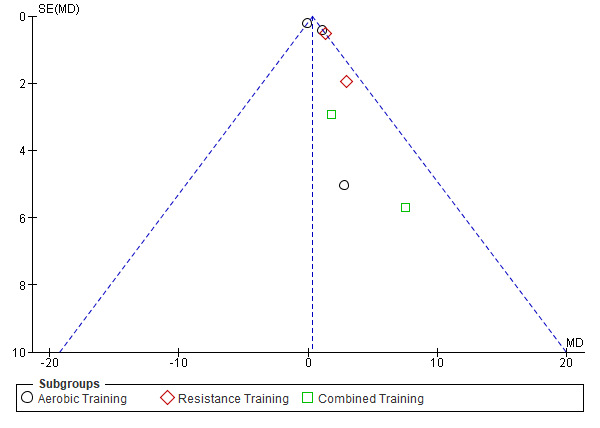** | **SF8j:** Lean body mass (kg) p=0.06  **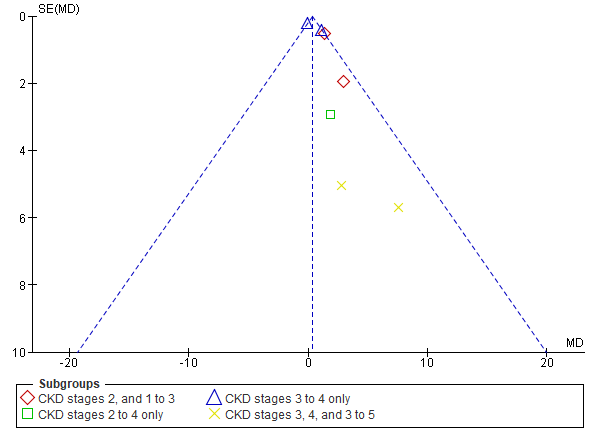** |

**Supplemental Figure 9** Inflammatory markers

**SF9a:** Interleukin-6 (by intervention modality); **SF9b:** Interleukin-6 (by CKD stage); **SF9c:** C-reactive protein (by intervention modality); **SF9d:** C-reactive protein (by CKD stage).

| **SF9a:** Interleukin-6 (pg/mL) p=0.007  **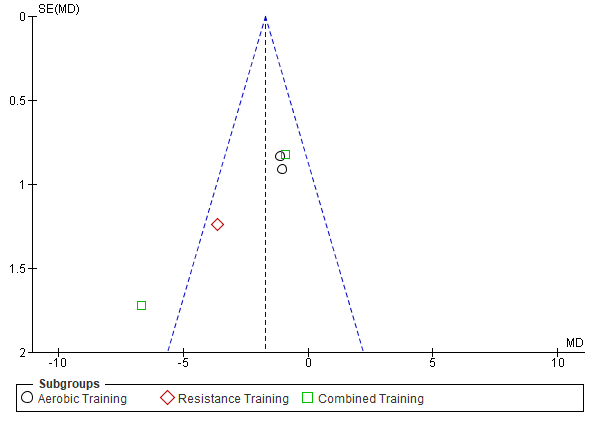** | **SF9b:** Interleukin-6 (pg/mL) p=0.007  **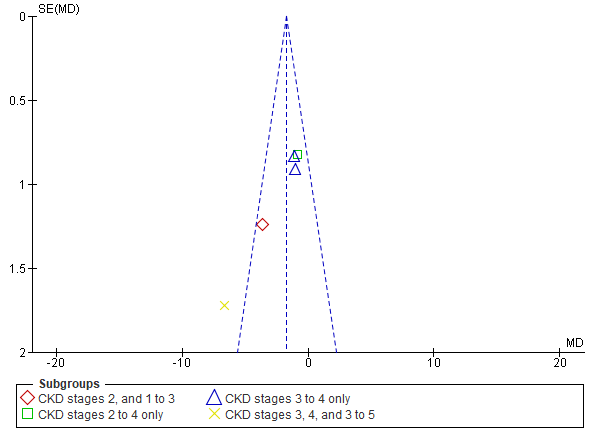** |
| --- | --- |
| **SF9c:** C-reactive protein (mg/L) p=0.92  **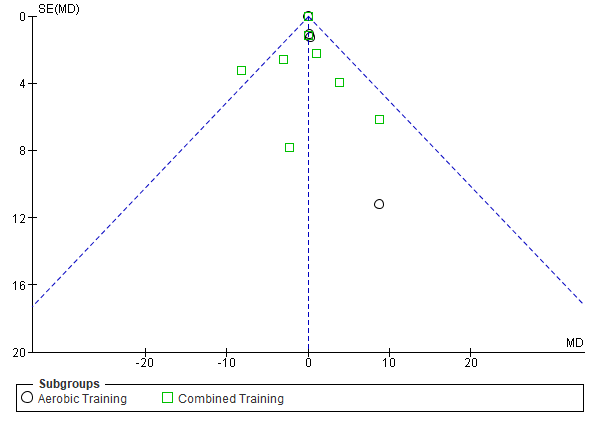** | **SF9c:** C-reactive protein (mg/L) p=0.92  **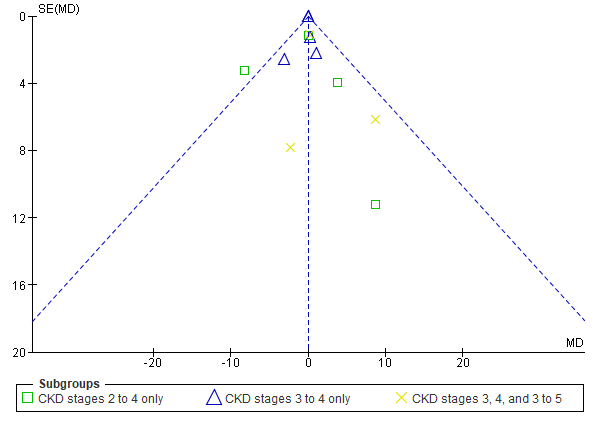** |
