# Supplementary material for: The effect of exercise training in people with pre-dialysis chronic kidney disease: a systematic review with meta-analysis
Source: J Nephrol. 2024 Oct 17;37(8):2063–98. doi: 10.1007/s40620-024-02081-9 (PMC11649798; doi:10.1007/s40620-024-02081-9)

**The effect of exercise training in people with pre-dialysis chronic kidney disease. A systematic review with meta-analysis.**

Annette Traise*, Gudrun Dieberg, Melissa J Pearson, Neil A Smart

Clinical Exercise Physiology, School of Science and Technology, University of New England, NSW 2351, Australia

* Corresponding author

**Online Resource 5**

**Supplemental material: Figures 10-14** Sub-analyses – Forest plots for outcomes with low-quality studies removed – exercise vs usual care

**Supplemental Figure 10** Sub-analyses with low-quality studies removed: Aerobic capacity and Functional ability

**Supplemental Figure 11** Sub-analyses with low-quality studies removed: Quality of Life (QoL)

**Supplemental Figure 12** Sub-analyses with low-quality studies removed: Renal Parameters

**Supplemental Figure 13** Sub-analyses Forest plot with low-quality studies removed: Cardiovascular Risk Factors

**Supplemental Figure 14** Sub-analyses Forest plot with low-quality studies removed: Inflammatory Markers

**Supplemental Figure 10** Sub-analyses with low-quality studies removed: Aerobic capacity and Functional ability – exercise vs usual care

**SF10a:** Peak VO_2_; **SF10b:** 6-Minute Walk Test (6MWT); **SF10c:** Timed Up and Go Test (TUG); **SF10d:** Handgrip Strength

**SF10a** Peak VO_2_ [mL/kg/min] sub-analysis by CKD stage with low-quality studies removed

(p<0.00001 when all studies included)


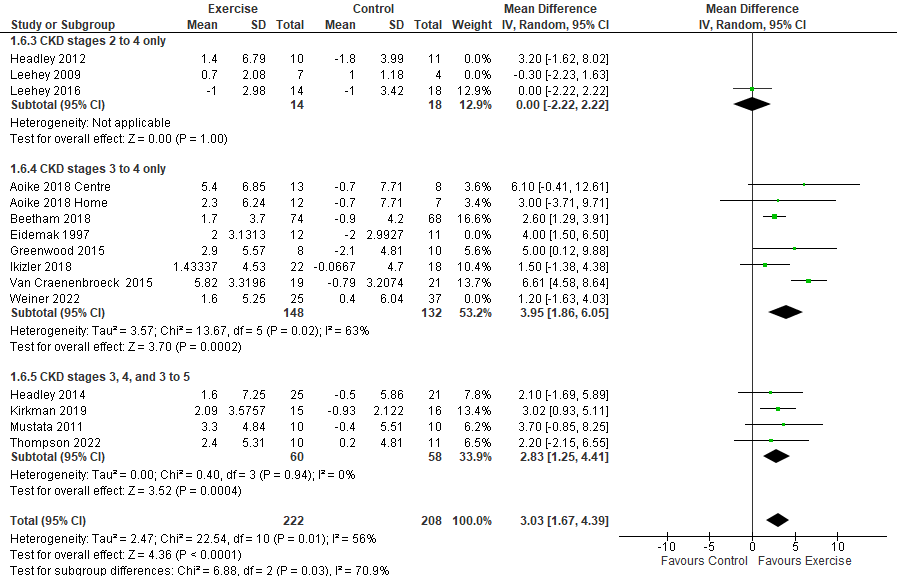


**SF10b** 6-Minute Walk Test (6MWT) [meters] sub-analysis by CKD stage with low-quality studies removed

(p<0.00001 when all studies included)


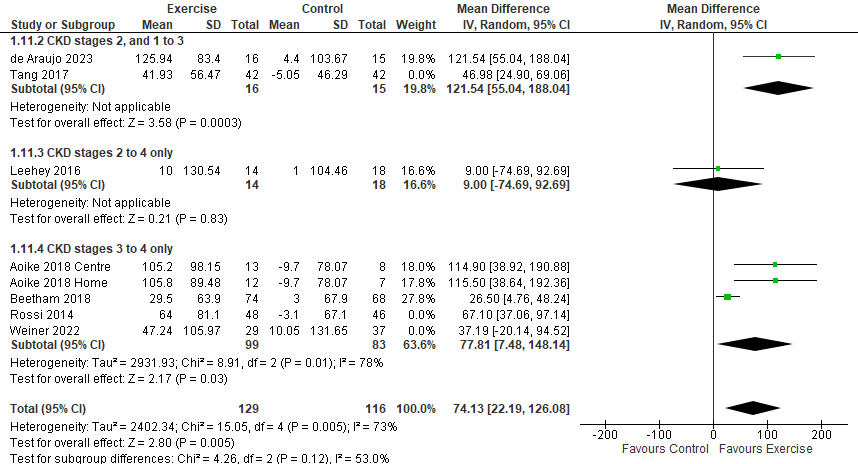


**SF10c** Timed Up and Go Test (TUG) {SMD} sub-analysis by CKD stage with low-quality studies removed

(p=0.0006 when all studies included)

**
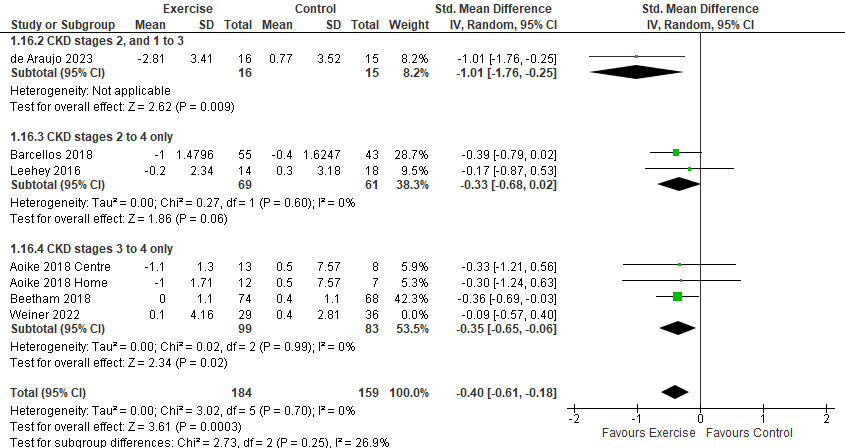
**

**SF10d** Handgrip Strength [kg] sub-analysis by CKD stage with low-quality studies removed

(p=0.13 when all studies included)

**
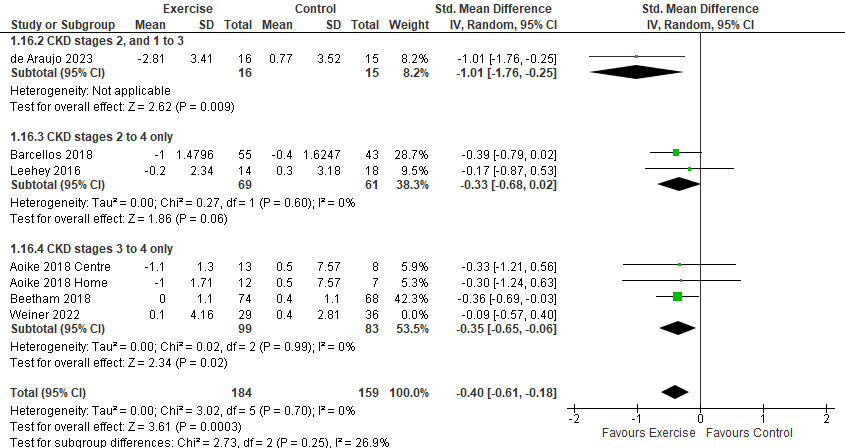
**

**Supplemental Figure 11** Sub-analyses with low-quality studies removed: Quality of Life (QoL) – exercise vs usual care

**SF11a:** Short Form 36 General Health (GH); **SF11b:** Short Form 36 Mental Component Summary (MCS); **SF11c:** Short Form 36 Physical Component Summary (PCS)

**SF11a** Short Form 36 General Health (GH) sub-analysis by CKD stage with low-quality studies removed

(p=0.05 when all studies included)


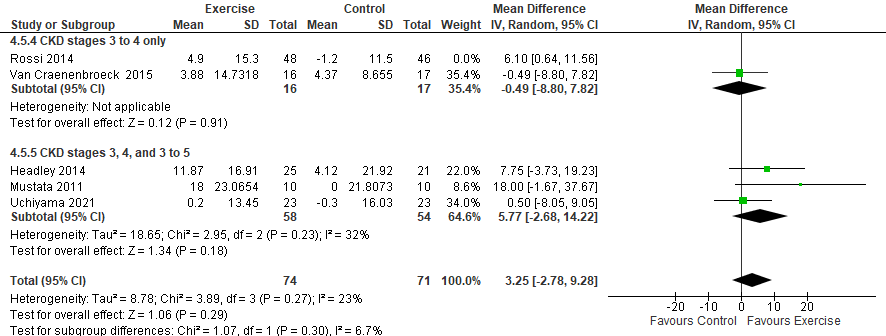


**SF11b** Short Form 36 Mental Component Summary (MCS) sub-analysis by CKD stage with low-quality studies removed

(p=0.03 when all studies included)
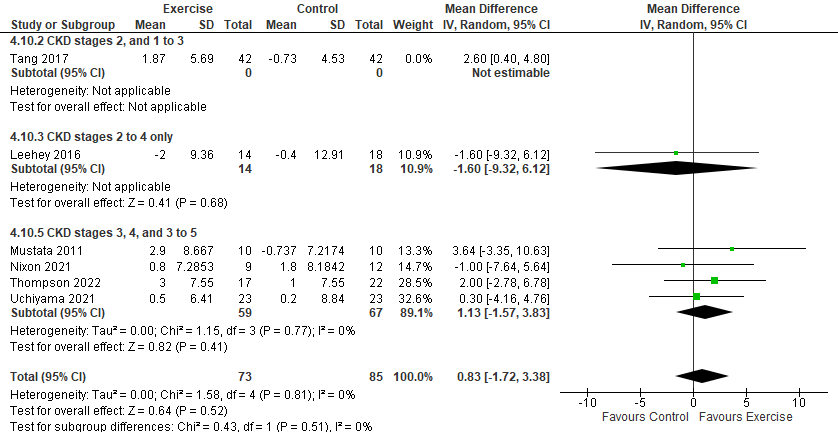


**SF11c** Short Form 36 Physical Component Summary (PCS) sub-analysis by CKD stage with low-quality studies removed

(p=0.22 when all studies included)


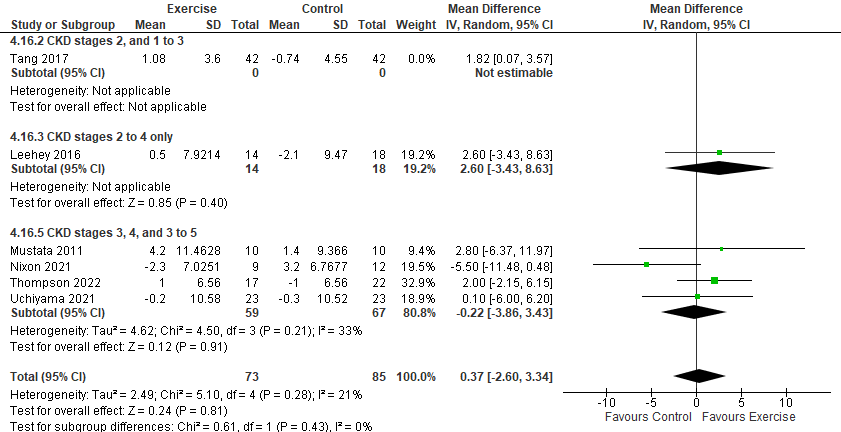


**Supplemental Figure 12** Sub-analyses with low-quality studies removed: Renal Parameters – exercise vs usual care

**SF12a:** Estimated Glomerular Filtration Rate (eGFR_Cr_); **SF12b:** Serum Creatinine (sCr); **SF12c:** Urine Albumin-to-Creatinine Ratio (UACR); **SF12d:** Urine Protein-to-Creatinine Ratio (UPCR); **SF12e:** 24-hour Urine Protein; **SF12f:** Blood Urea Nitrogen (BUN)

**SF12a** Estimated Glomerular Filtration Rate (eGFR_Cr_) [mL/min/1.73m^2^] sub-analysis by CKD stage with low-quality studies removed

(p=0.001 when all studies included)


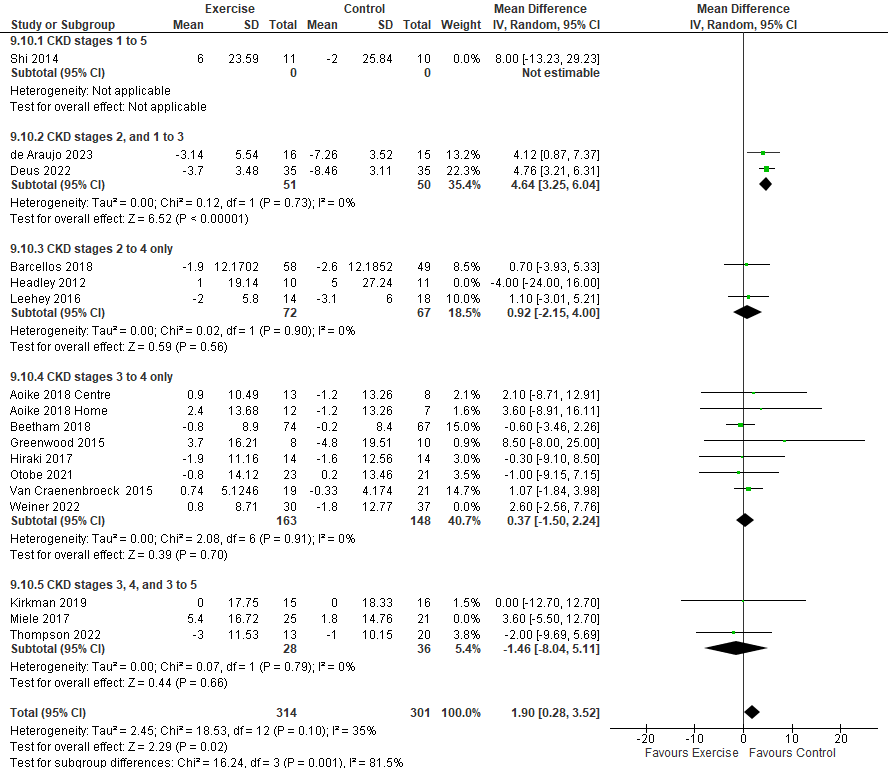


**SF12b** Serum Creatinine (sCr) [mg/dL] sub-analysis by CKD stage with low-quality studies removed

(p=0.39 when all studies included)


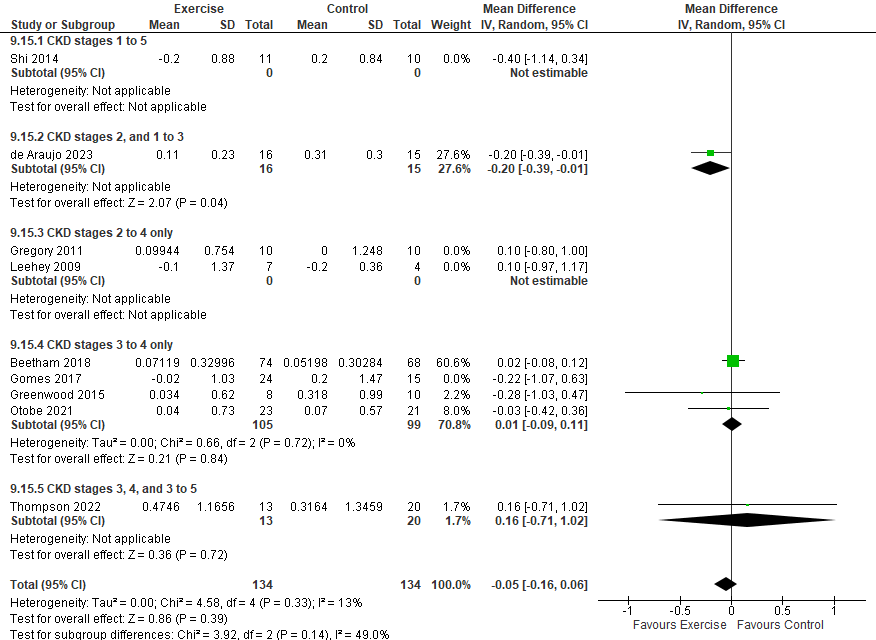


**SF12c** Urine Albumin-to-Creatinine Ratio (UACR) [mg/g] sub-analysis by CKD stage with low-quality studies removed

(p=0.90 when all studies included)

**
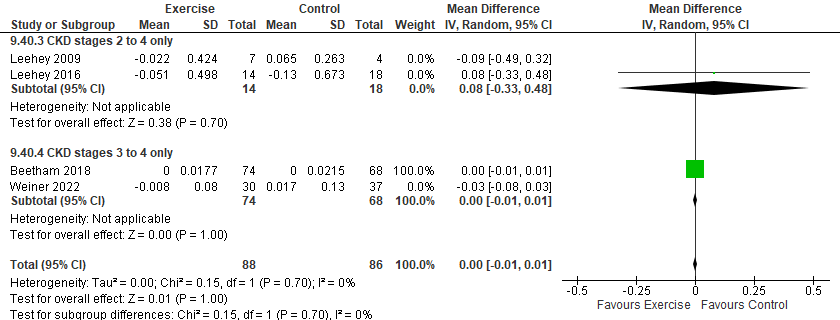
**

**SF12d** Urine Protein-to-Creatinine Ratio (UPCR) [mg/g] sub-analysis by CKD stage with low-quality studies removed

(p=0. when all studies included)

**
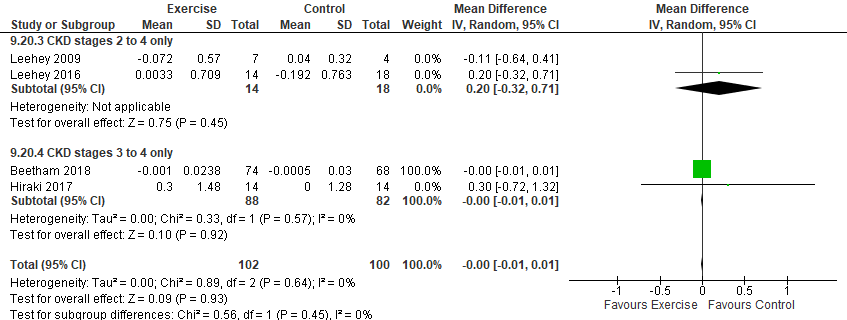
**

**SF12e** 24-hour Urine Protein [g/24hr] sub-analysis by CKD stage with low-quality studies removed

(p=0.79 when all studies included)

**
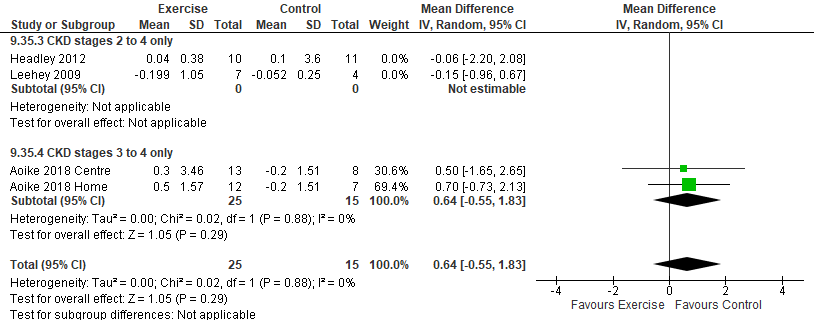
**

**SF12f** Blood Urea Nitrogen (BUN) [mg/dL] with by CKD stage low-quality studies removed

(p=0.82 when all studies included)

**
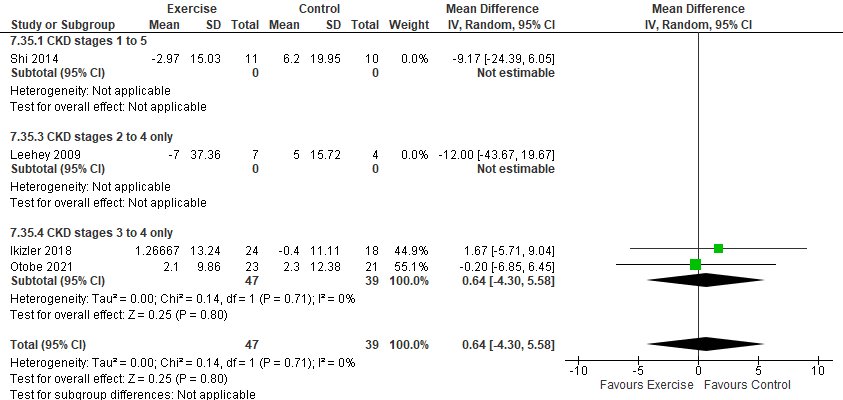
**

**Supplemental Figure 13** Sub-analyses Forest plot with low-quality studies removed: Cardiovascular Risk Factors – exercise vs usual care

**SF13a:** Resting Heart Rate (RHR); **SF13b:** Systolic Blood Pressure (SBP); **SF13c:** Diastolic Blood Pressure (DBP); **SF13d:** Ambulatory 24-hour Systolic Blood Pressure; **SF13e:** Ambulatory 24-hour Diastolic Blood Pressure; **SF13f:** Asymmetric dimethylarginine; **SF13g:** Triglyceride (TG); **SF13h:** Total Cholesterol (TC); **SF13i:** Low Density Lipoprotein (LDL-C); **SF13j:** High Density Lipoprotein (HDL-C); **SF13k:** Glycosylated Haemoglobin (HbA1c); **SF13l:** Blood Glucose (BG); **SF13m:** Haemoglobin (Hb); **SF13n:** Waist Circumference; **SF13o:** Body Weight [kg]; **SF13p:** Body Mass Index (BMI); **SF13q:** Lean Body Mass (LBM)

**SF13a** Resting Heart Rate (RHR) [beats/min] sub-analysis by CKD stage with low-quality studies removed

(p=0.04 when all studies included)


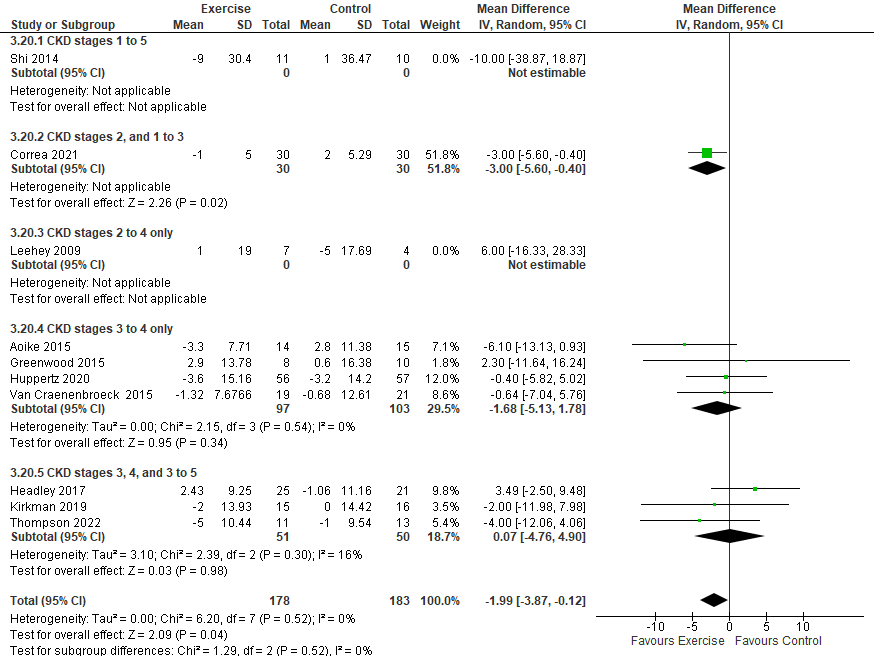


**SF13b** Systolic Blood Pressure (SBP) [mmHg] sub-analysis by CKD stage with low-quality studies removed

(p=0.35 when all studies included)


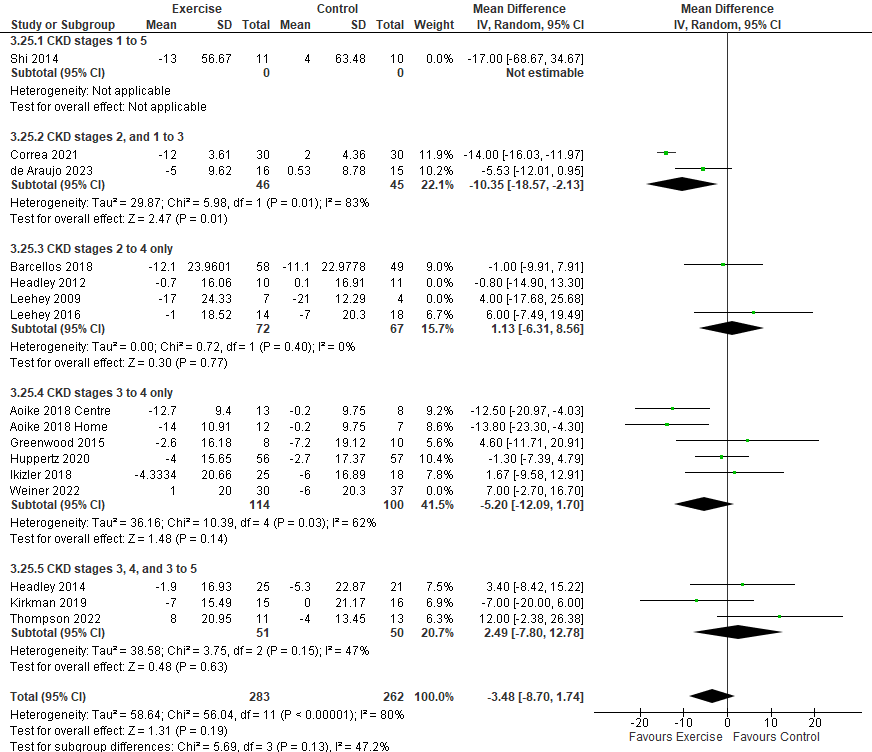


**SF13c** Diastolic Blood Pressure (DBP) [mmHg] sub-analysis by CKD stage with low-quality studies removed

(p=0.32 when all studies included)


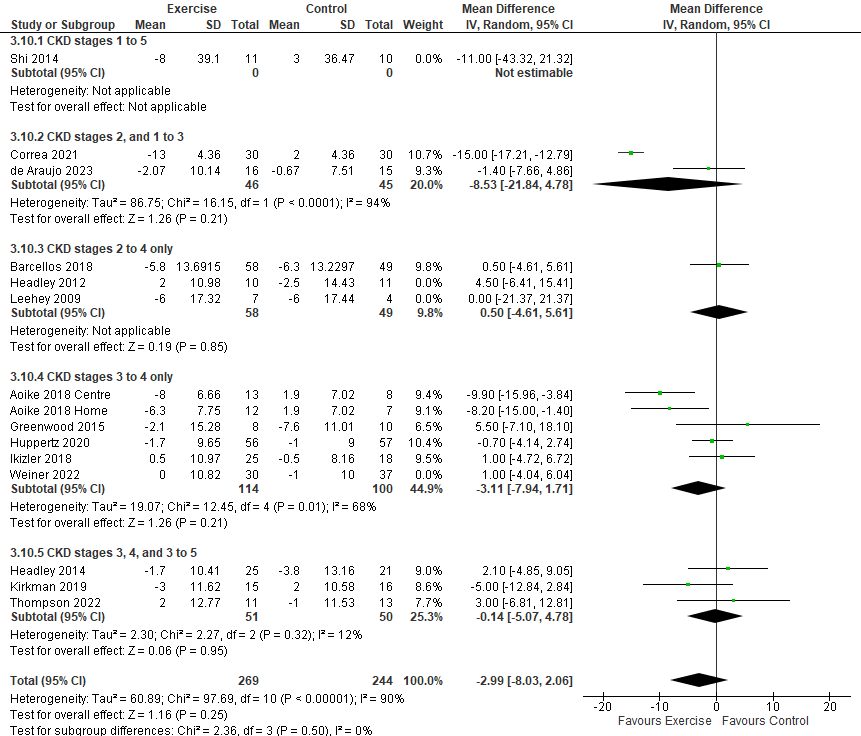


**SF13d** Ambulatory 24-hour Systolic Blood Pressure [mmHg] sub-analysis by CKD stage with low-quality studies removed

(p=0.97 when all studies included)

**
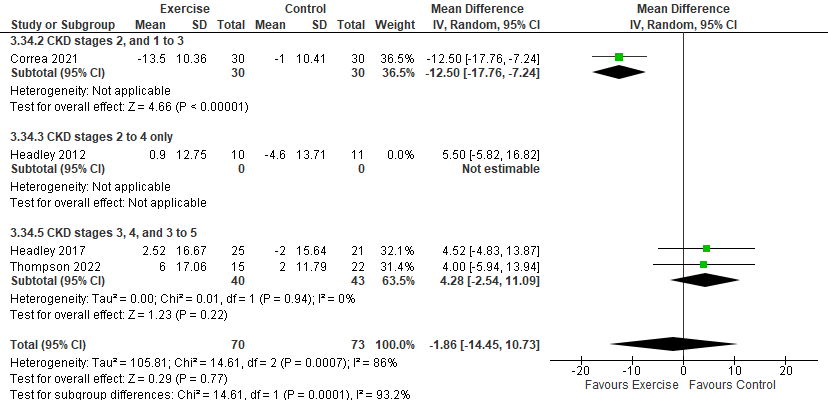
**

**SF13e** Ambulatory 24-hour Diastolic Blood Pressure [mmHg] sub-analysis by CKD stage with low-quality studies removed

(p=0.83 when all studies included)


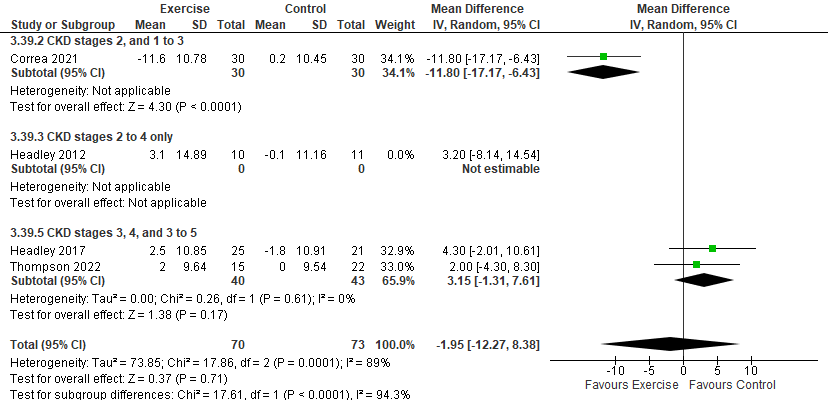


**SF13f** Asymmetric dimethylarginine (umol/L) sub-analysis by CKD stage with low-quality studies removed

(p=0.07 when all studies included)


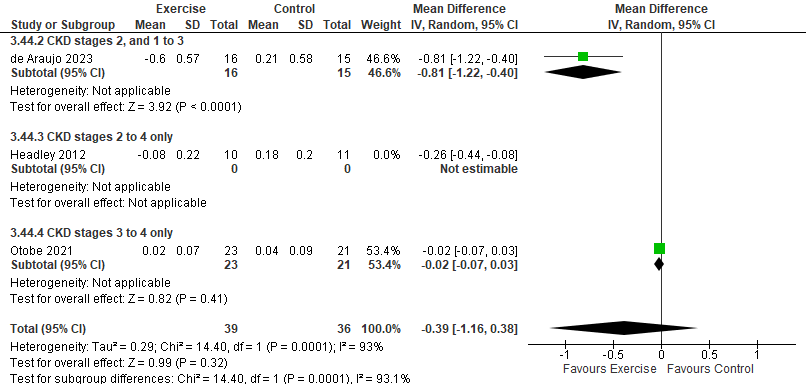


**SF13g** Triglyceride (TG) [mg/dL] sub-analysis by CKD stage with low-quality studies removed

(p<0.00001 when all studies included)


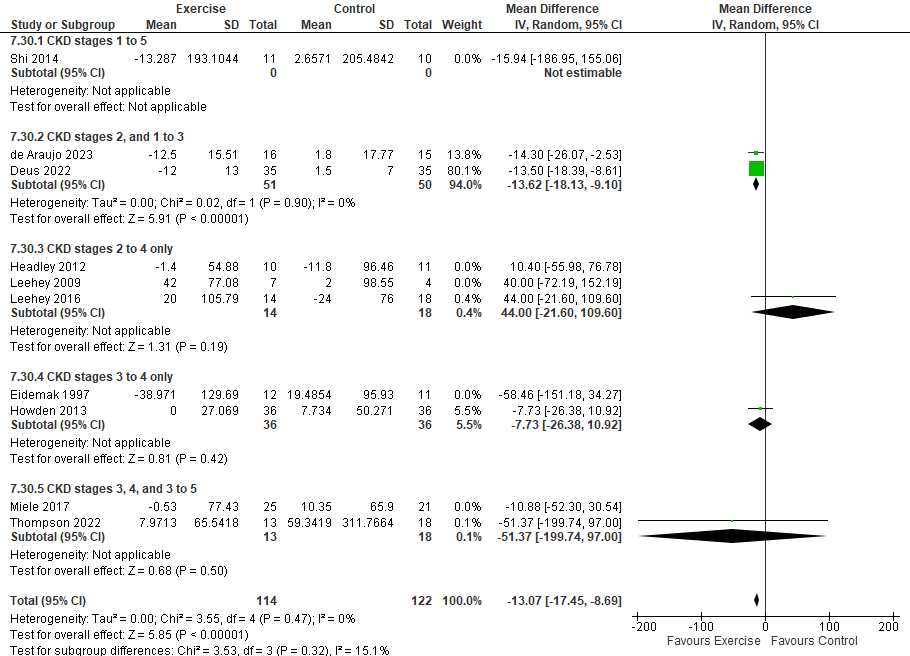


**SF13h** Total Cholesterol (TC) [mg/dL] sub-analysis by CKD stage with low-quality studies removed

(p=0.20 when all studies included)


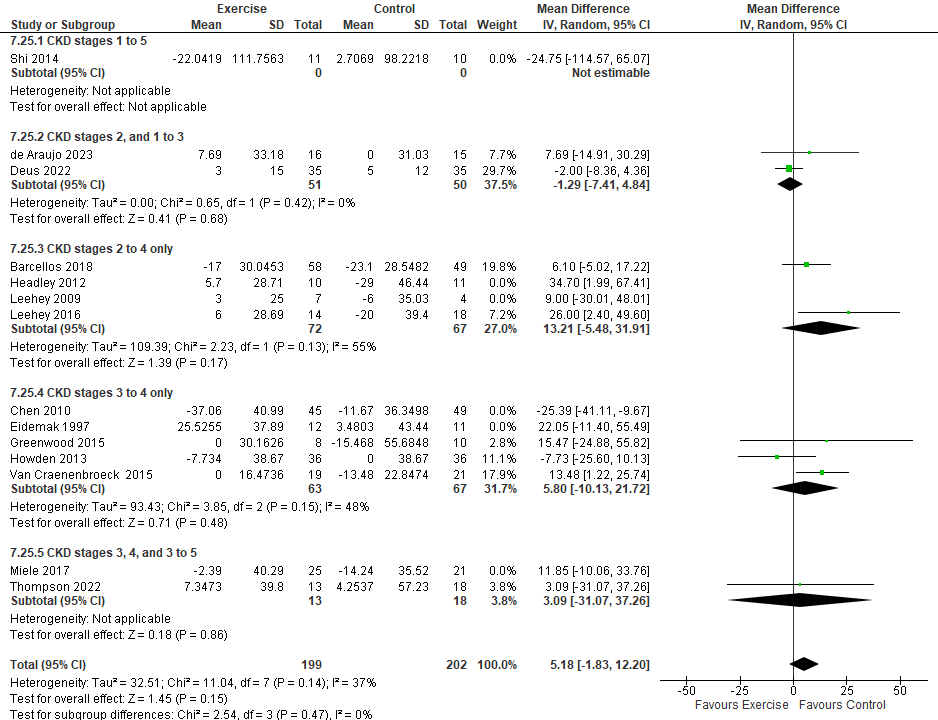


**SF13i** Low Density Lipoprotein (LDL-C) [mg/dL] sub-analysis by CKD stage with low-quality studies removed

(p=0.06 when all studies included)


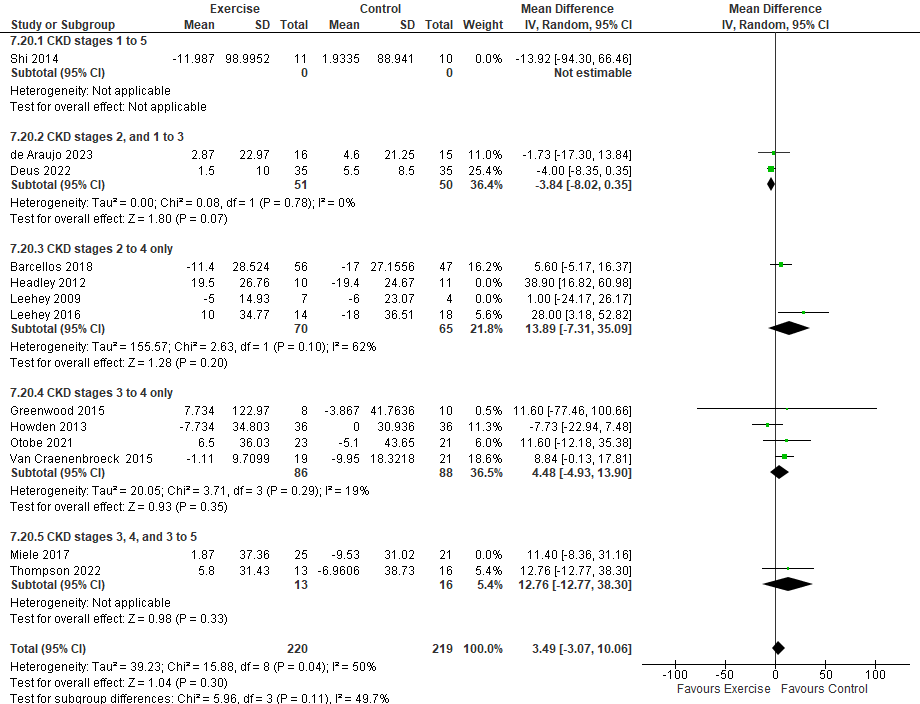


**SF13j** High Density Lipoprotein (HDL-C) [mg/dL] sub-analysis by CKD stage with low-quality studies removed

(p=0.11 when all studies included)


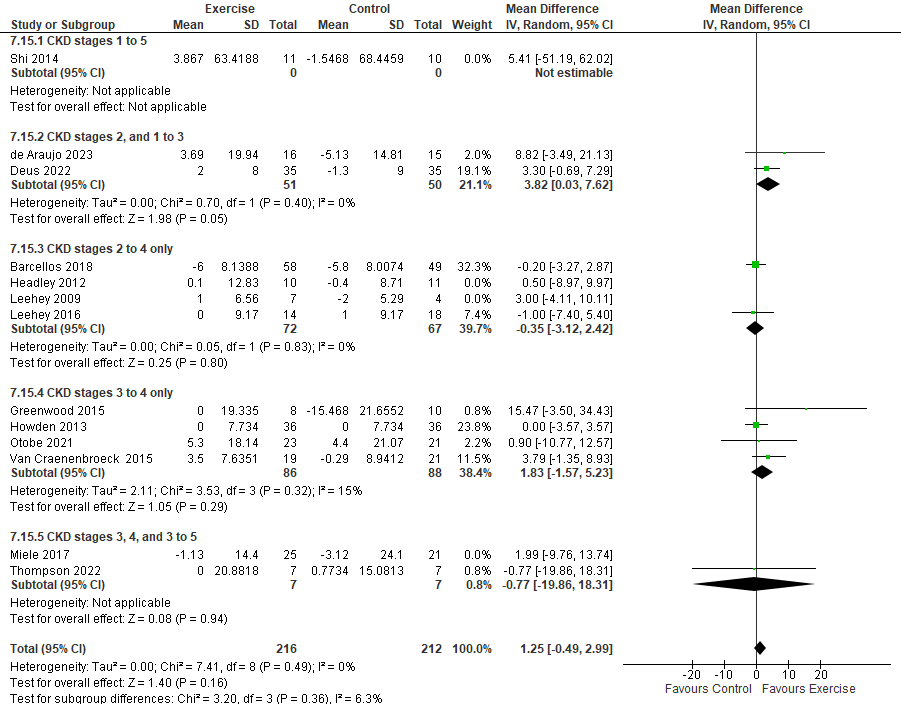


**SF13k** Glycosylated Haemoglobin (HbA1c) [%] sub-analysis by CKD stage with low-quality studies removed

(p=0.04 when all studies included)


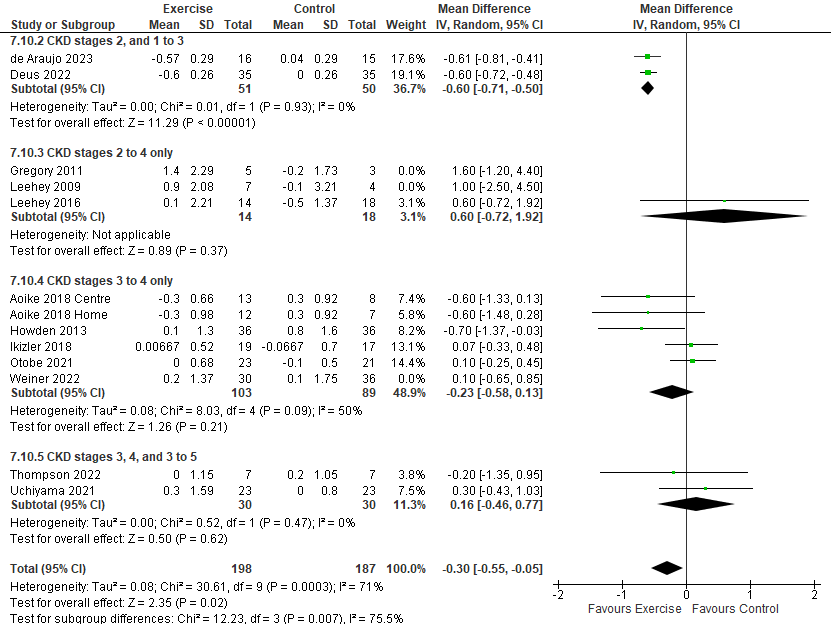


**SF13l** Blood Glucose (BG) [mg/dL] sub-analysis by CKD stage with low-quality studies removed

(p=0.29 when all studies included)


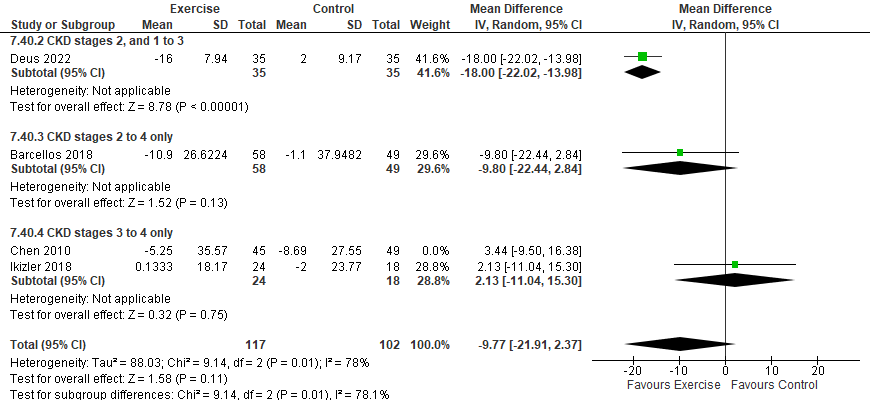


**SF13m** Haemoglobin (Hb) [g/dL] sub-analysis by CKD stage with low-quality studies removed

(p=0.08 when all studies included)

**
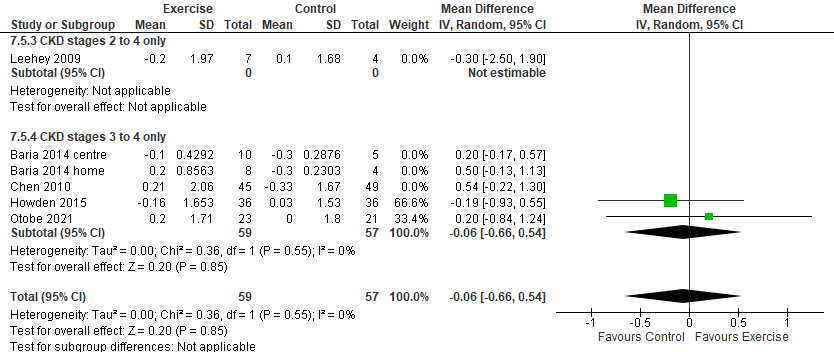
**

**SF13n** Waist Circumference [cm] sub-analysis by CKD stage with low-quality studies removed

(p<0.00001 when all studies included)


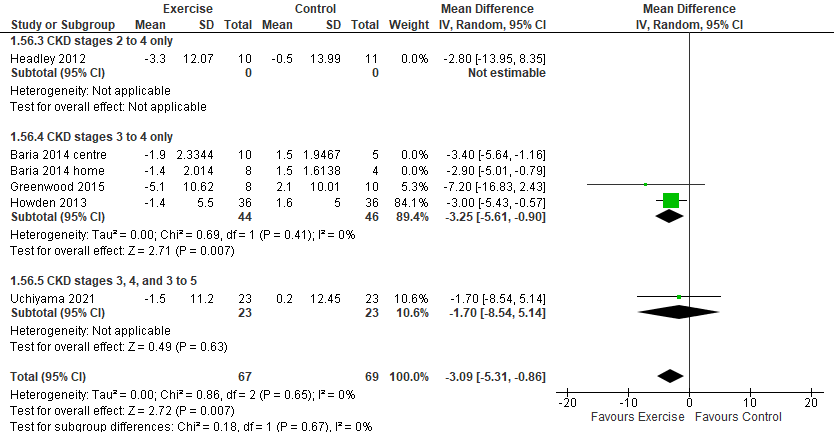


**SF13o** Body Weight [kg] sub-analysis by CKD stage with low-quality studies removed

(p=0.62 when all studies included)


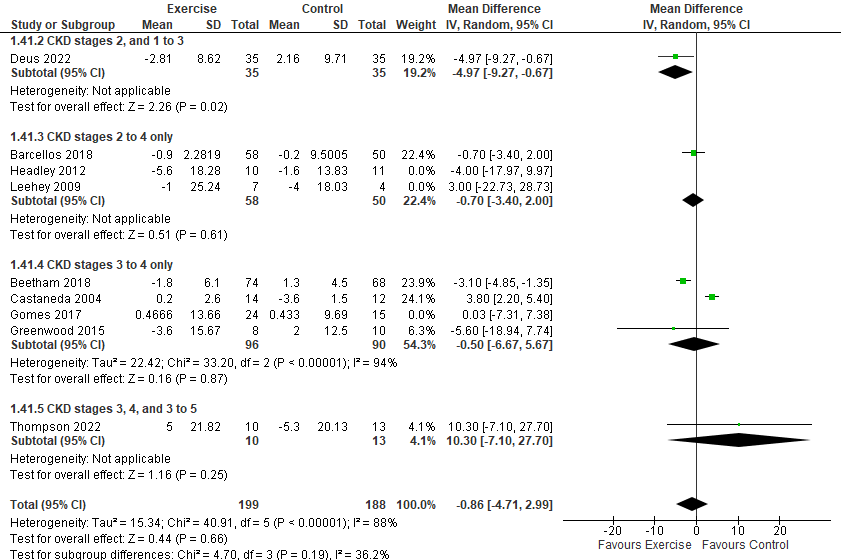


**SF13p** Body Mass Index (BMI) [kg/m^2^] sub-analysis by CKD stage with low-quality studies removed

(p=0.18 when all studies included)


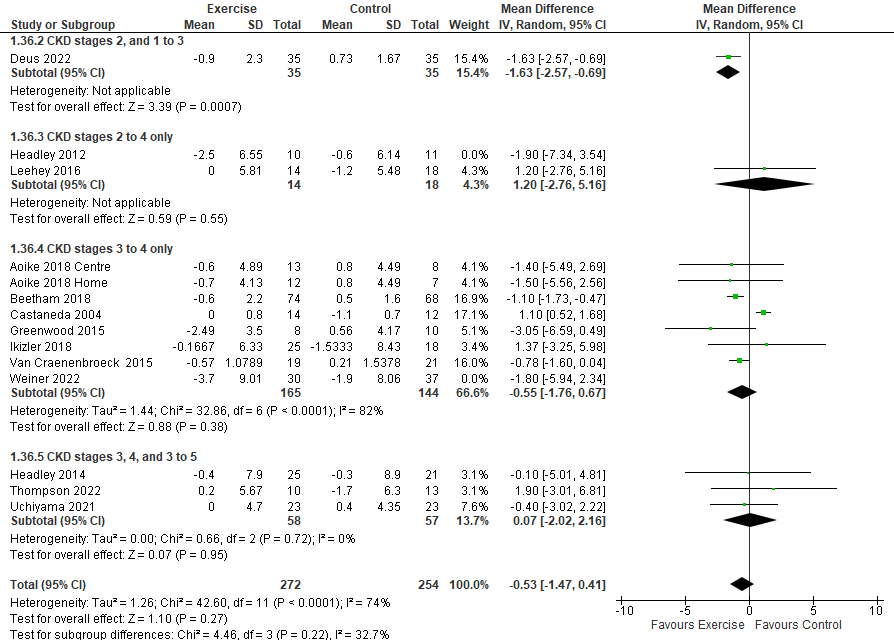


**SF13q** Lean Body Mass (LBM) [kg] sub-analysis by CKD stage with low-quality studies removed

(p=0.06 when all studies included)


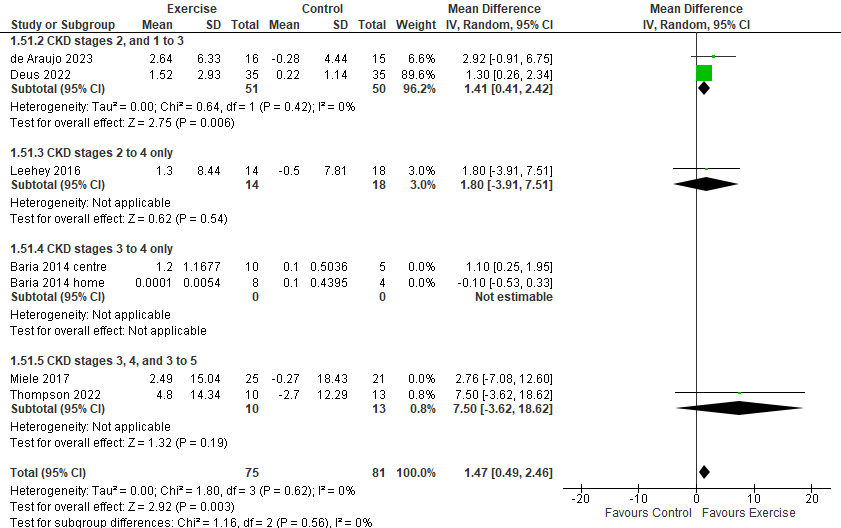


**Supplemental Figure 14** Sub-analyses Forest plot with low-quality studies removed: Inflammatory Markers – exercise vs usual care

**S14a:** Interleukin-6 (Il-6); **SF14b:** C-Reactive Protein (CRP)

**S14a** Interleukin-6 (Il-6) [pg/mL] sub-analysis by CKD stage with low-quality studies removed

(p=0.007 when all studies included)


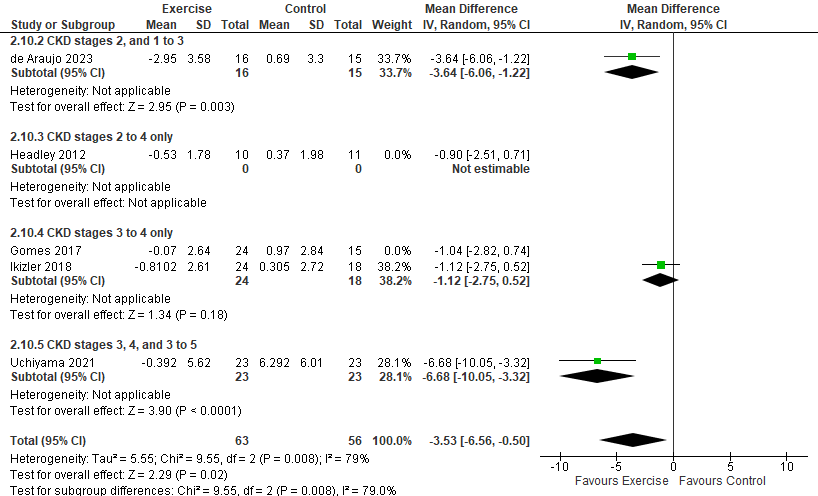


**SF14b** C-Reactive Protein (CRP) [mg/L] {hs-CRP and non hs-CRP combined} sub-analysis by CKD stage with low-quality studies removed

(p=0.92 when all studies included)


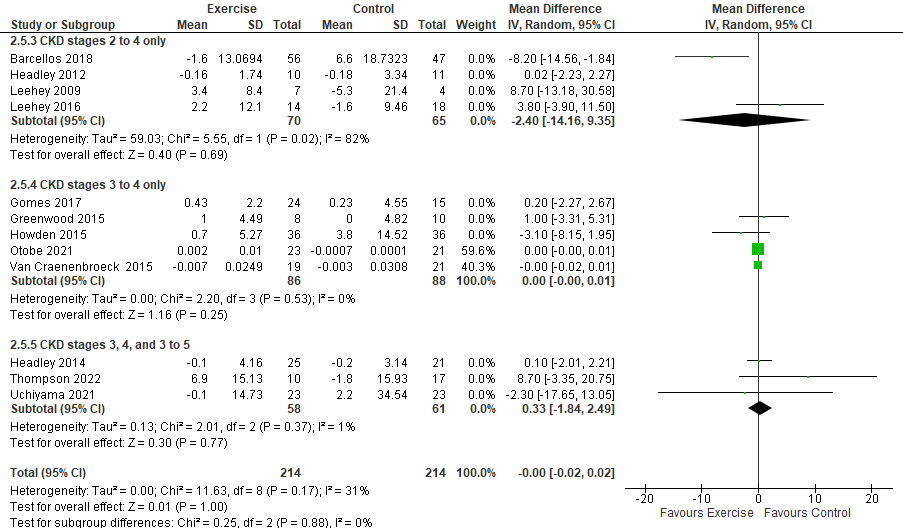

Supplement: Supplementary file 2 — Supplementary file2 (DOCX 778 KB) [file 40620_2024_2081_MOESM2_ESM.docx]
