# Supplementary material for: The effect of exercise training in people with pre-dialysis chronic kidney disease: a systematic review with meta-analysis
Source: J Nephrol. 2024 Oct 17;37(8):2063–98. doi: 10.1007/s40620-024-02081-9 (PMC11649798; doi:10.1007/s40620-024-02081-9)

**The effect of exercise training in people with pre-dialysis chronic kidney disease. A systematic review with meta-analysis.**

Annette Traise*, Gudrun Dieberg, Melissa J Pearson, Neil A Smart

Clinical Exercise Physiology, School of Science and Technology, University of New England, NSW 2351, Australia

* Corresponding author

**Online Resource 7**

**Supplemental material: Figures 15 to 19 Non-significant meta-analyses**

**Supplemental Figure SF15** Forest plots for non-significant Aerobic Capacity and Functional Ability

**Supplemental Figure SF16** Forest plots for non-significant Quality of Life

**Supplemental Figure SF17** Forest plots for non-significant Renal Parameters

**Supplemental Figure SF18** Forest plots for non-significant Cardiovascular Risk Factors

**Supplemental Figure SF19** Forest plot for non-significant Inflammatory Markers

**Supplemental Figure SF15** Forest plots for non-significant Aerobic Capacity and Functional Ability – exercise versus usual care

**SF15a:** Peak expiratory exchange ratio by modality; **SF15b:** Peak expiratory exchange ratio by CKD stage; **SF15c:**  Handgrip Strength by modality; **SF15d:** Handgrip Strength by CKD stage;

**SF15a** Peak expiratory exchange ratio in people with pre-dialysis CKD by modality p=0.44:

**
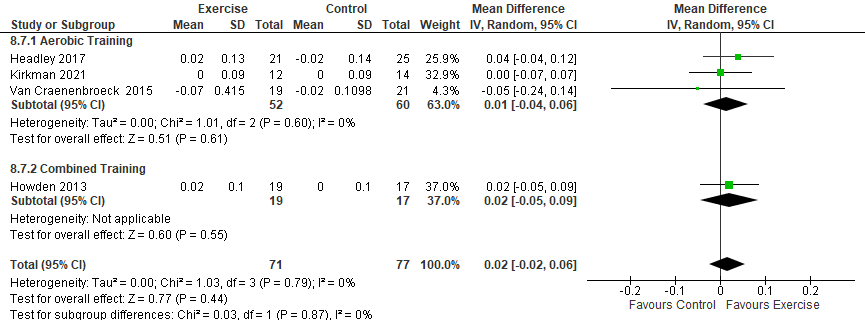
**

**SF15b** Peak expiratory exchange ratio in people with pre-dialysis CKD by CKD stage p=0.44:

**
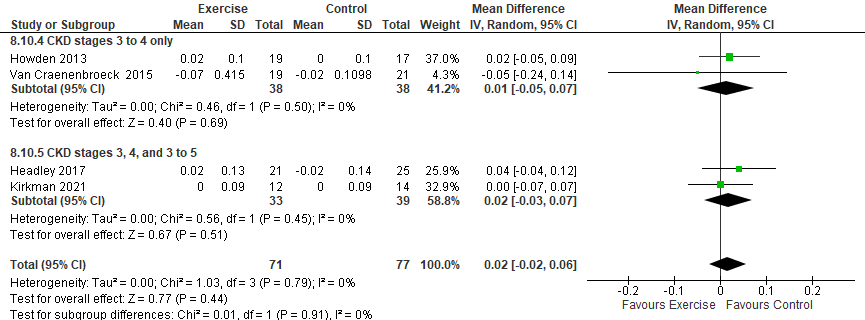
**

**SF15c** Handgrip Strength [kg] in people with pre-dialysis CKD by modality p=0.13:
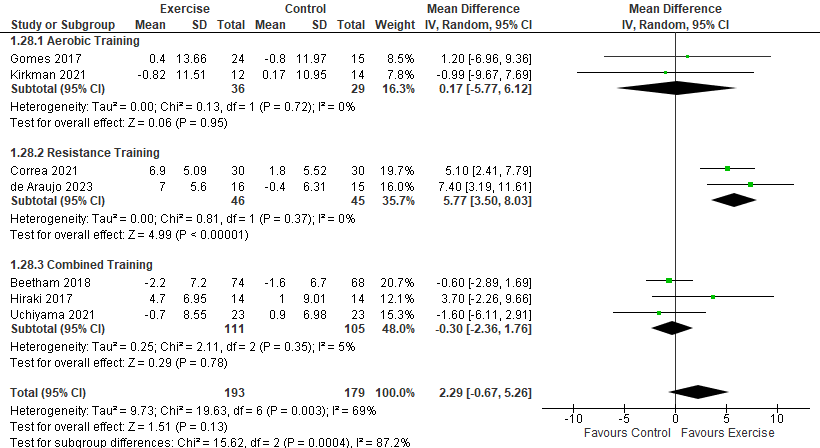


**SF15d** Handgrip Strength [kg] in people with pre-dialysis CKD by CKD stage p=0.13:

**
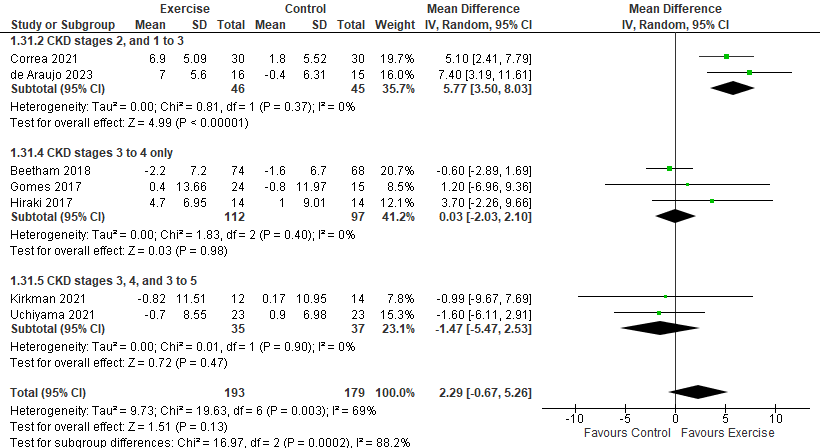
**

**Supplemental Figure SF16** Forest plots for non-significant Quality of Life – exercise versus usual care

**SF16a:** Physical Component Summary by modality; **SF16b:** Physical Component Summary by CKD stage

**SF16a** Physical Component Summary in people with pre-dialysis CKD by modality p=0.22:

**
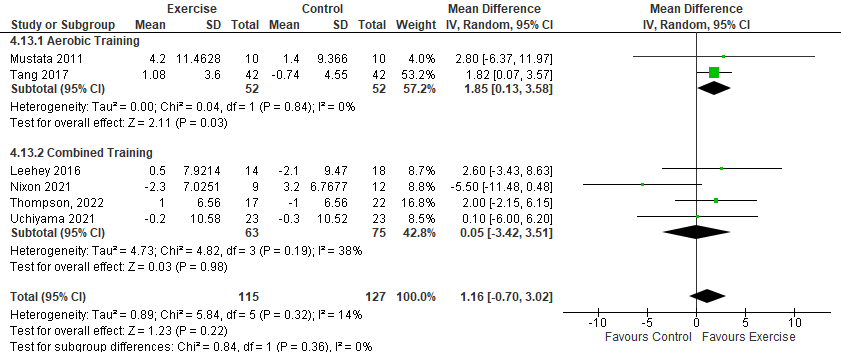
**

**SF16b** Physical Component Summary in people with pre-dialysis CKD by CKD stage p=0.22:**
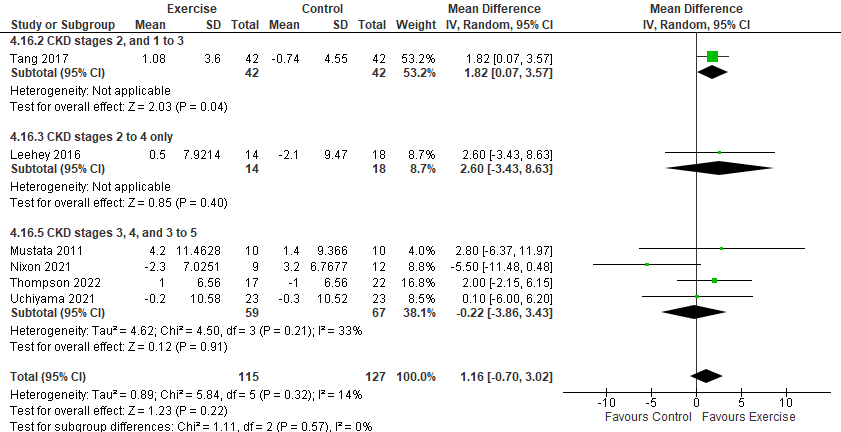
**

**Supplemental Figure SF17** Forest plots for non-significant Renal Parameters – exercise versus usual care

**SF17a:** Glomerular Filtration Rate (eGFR_Cys_) by modality; **SF17b** Glomerular Filtration Rate (eGFR_Cys_) by CKD stage; **SF17c:** Serum Creatinine (sCr) by modality; **SF17d:** Serum Creatinine (sCr) by CKD stage; **SF17e:** Serum Albumin (ALB) by modality; **SF17f:** Serum Albumin (ALB) by CKD stage; **SF17:g** Urine Albumin-to-Creatinine Ratio (UACR) by modality; **SF17h:** Urine Albumin-to-Creatinine Ratio (UACR) by CKD stage; **SF17i:** Urine Protein-to-Creatinine Ratio (UPCR) by modality; **SF17j:** Urine Protein-to-Creatinine Ratio (UPCR) by CKD stage; **SF17k:** 24-hour Urine Protein by modality; **SF17l:** 24-hour Urine Protein by CKD stage; **SF17m:** Blood Urea Nitrogen (BUN) by modality; **SF17n:** Blood Urea Nitrogen (BUN) by CKD stage

**SF17a** Glomerular Filtration Rate (eGFR_Cys_) [ml/min/1.73m^2^] in people with pre-dialysis CKD by modality p=0.15:

**
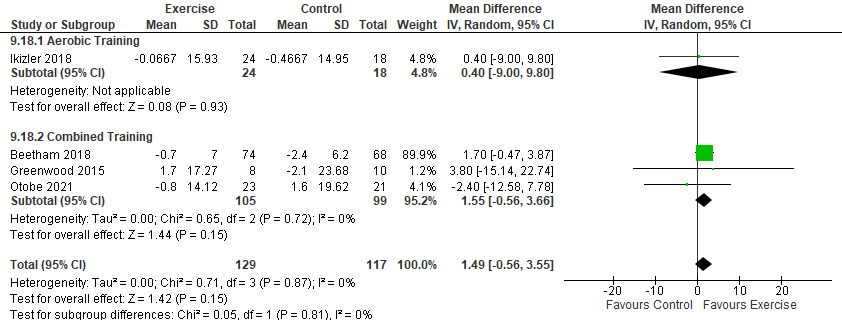
**

**SF17b** Glomerular Filtration Rate (eGFR_Cys_) [ml/min/1.73m^2^] in people with pre-dialysis CKD by CKD stage p=0.15:

**
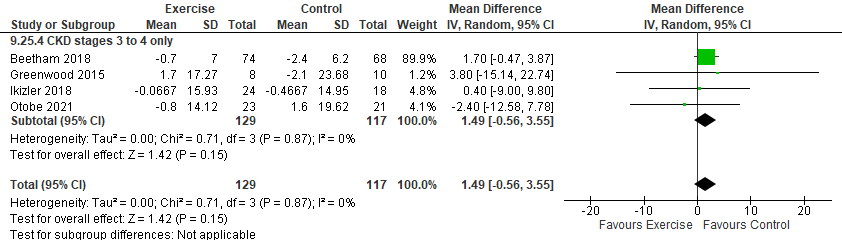
**

**SF17c** Serum Creatinine (sCr) [mg/dL] in people with pre-dialysis CKD by modality p=0.39:
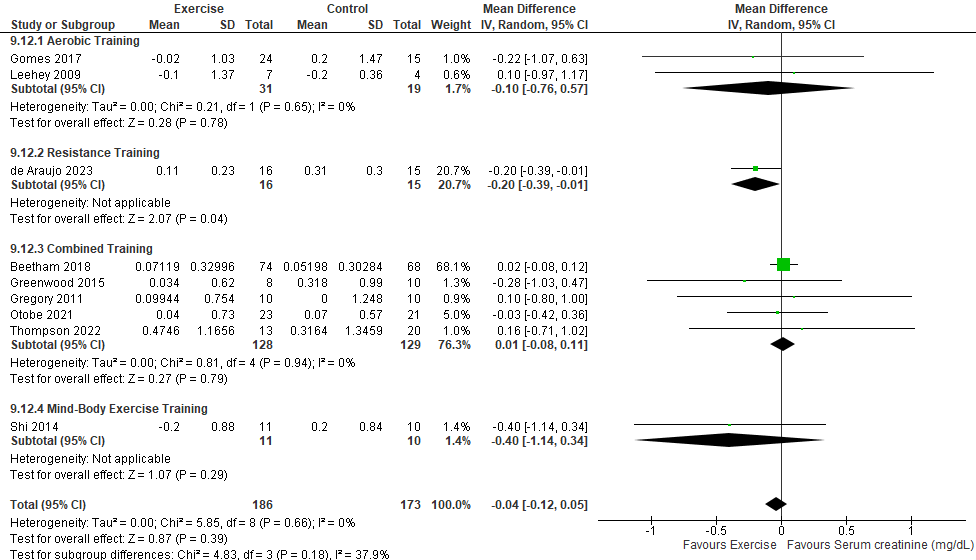


**SF17d** Serum Creatinine (sCr) [mg/dL] in people with pre-dialysis CKD by CKD stage p=0.39:

**
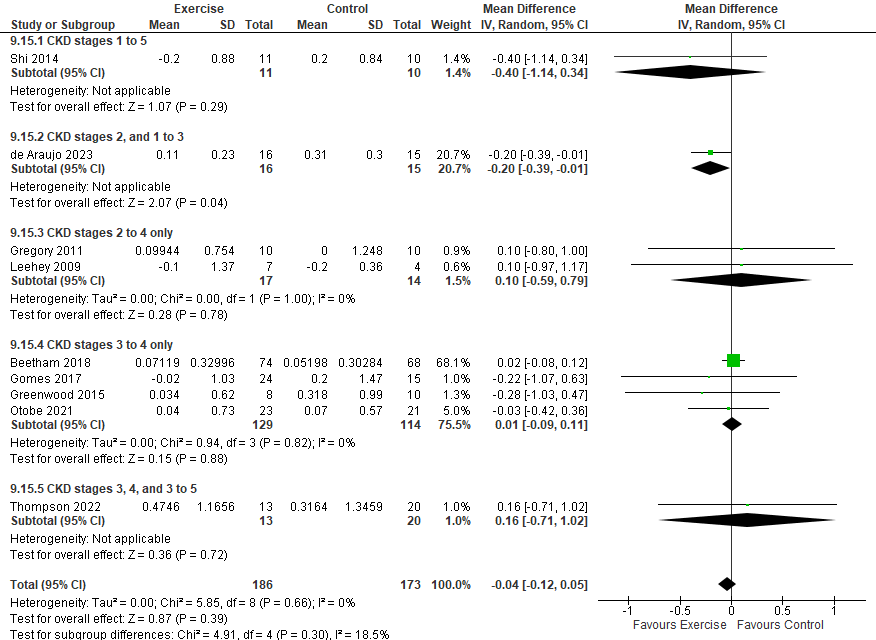
**

**SF17e** Serum Albumin (ALB) [g/dL] in people with pre-dialysis CKD by modality p=0.16:
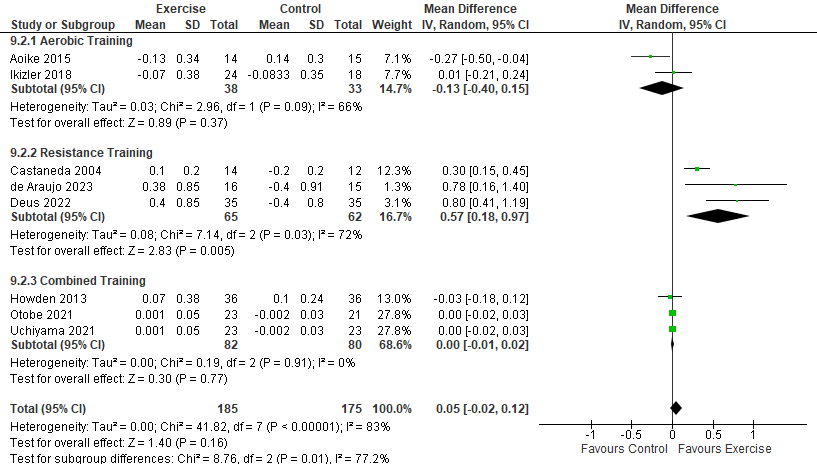


**SF17f** Serum Albumin (ALB) [g/dL] in people with pre-dialysis CKD by CKD stage p=0.16:


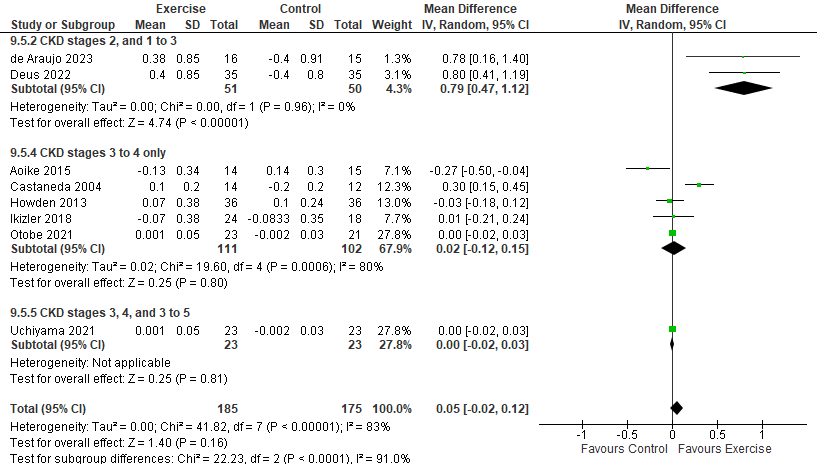


**SF17g** Urine Albumin-to-Creatinine Ratio (UACR) [mg/g] in people with pre-dialysis CKD by modality p=0.90:


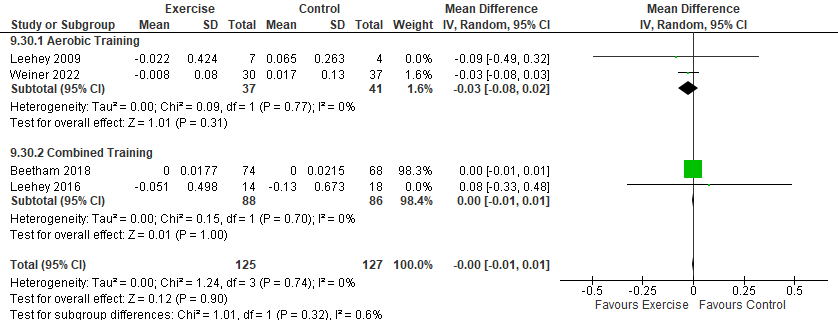


**SF17h** Urine Albumin-to-Creatinine Ratio (UACR) [mg/g] in people with pre-dialysis CKD by CKD stage p=0.90: **
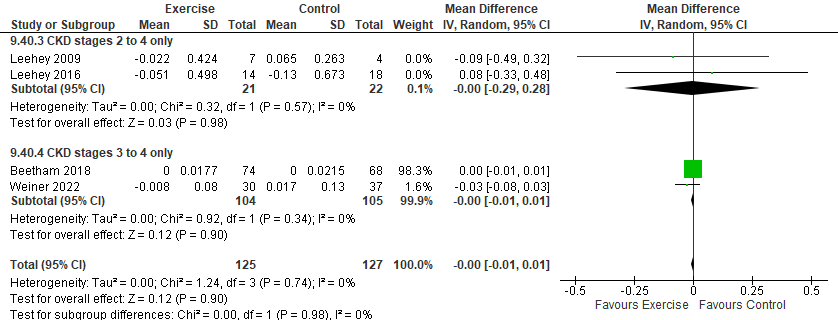
**

**SF17i** Urine Protein-to-Creatinine Ratio (UPCR) [mg/g] in people with pre-dialysis CKD by modality p=0.92:


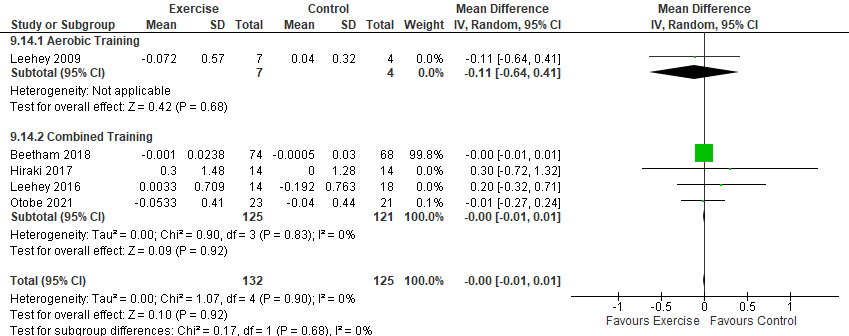


**SF17j** Urine Protein-to-Creatinine Ratio (UPCR) [mg/g] in people with pre-dialysis CKD by CKD stage p=0.92:

**
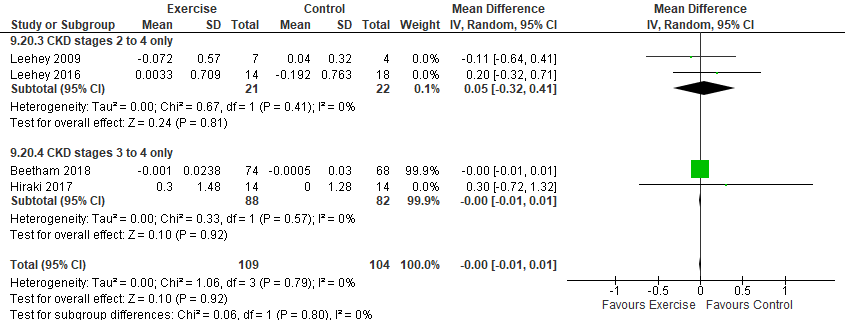
**

**SF17k** 24-hour Urine Protein [g/24hr] in people with pre-dialysis CKD by modality p=0.79:


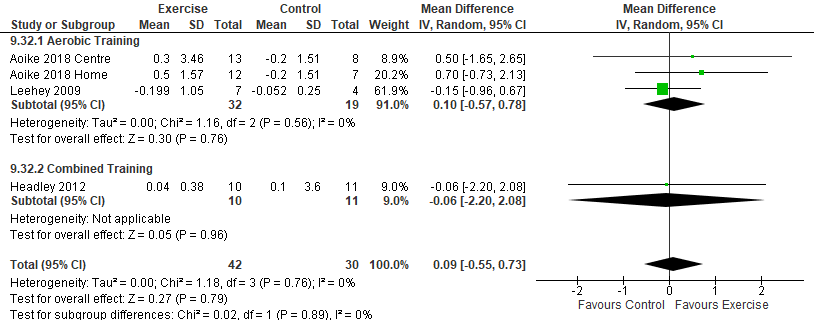


**SF17l** 24-hour Urine Protein [g/24hr] in people with pre-dialysis CKD by CKD stage p=0.79:
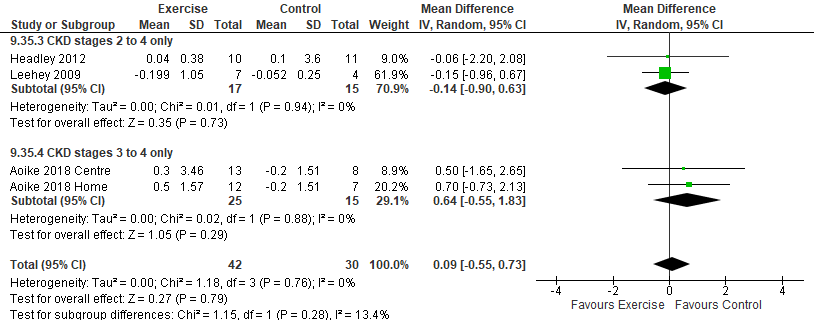


**SF17m** Blood Urea Nitrogen (BUN) [mg/dL] in people with pre-dialysis CKD by modality p=0.82:


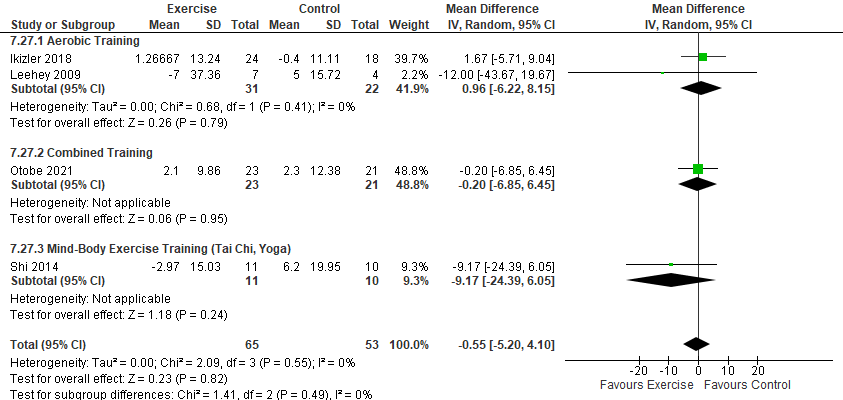


**SF17n** Blood Urea Nitrogen (BUN) [mg/dL] in people with pre-dialysis CKD by CKD stage p=0.82:

**
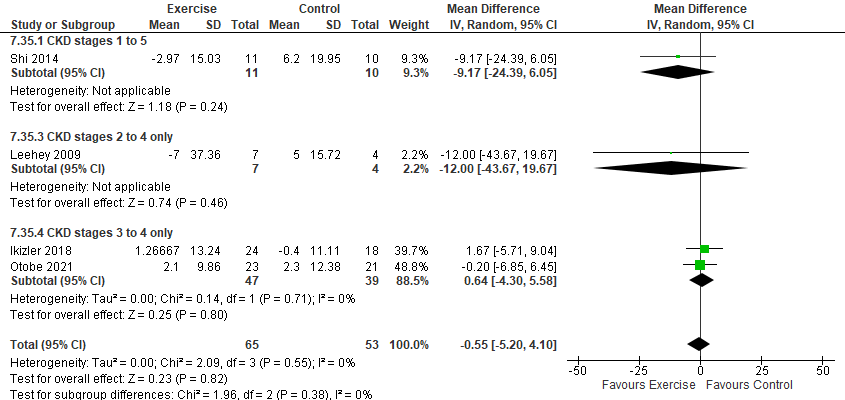
**

**Supplemental Figure SF18** Forest plots for non-significant Cardiovascular Risk Factors – exercise versus usual care

**SF18a:** Systolic Blood Pressure (SBP) by modality; **SF18b:** Systolic Blood Pressure (SBP) by CKD stage; **SF18c:** Diastolic Blood Pressure (DBP) by modality; **SF18d:** Diastolic Blood Pressure (DBP) by CKD stage; **SF18e:** Ambulatory 24-hour Systolic Blood Pressure by modality; **SF18f:** Ambulatory 24-hour Systolic Blood Pressure by CKD stage; **SF18g:** Ambulatory 24-hour Diastolic Blood Pressure by modality; **SF18h:** Ambulatory 24-hour Diastolic Blood Pressure by CKD stage; **SF18i:** Pulse Wave Velocity (PWV) by modality; **SF18j:** Pulse Wave Velocity (PWV) by CKD stage; **SF18k:** Augmentation Index (AIx) by modality; **SF18l:** Augmentation Index (AIx) by CKD stage; **SF18m:** Asymmetric dimethylarginine (ADMA) by modality; **SF18n:** Asymmetric dimethylarginine (ADMA) by CKD stage; **SF18o:** Total Cholesterol (TC) by modality; **SF18p:** Total Cholesterol (TC) by CKD stage; **SF18q:** Low Density Lipoprotein (LDL-C) by modality; **SF18r:** Low Density Lipoprotein (LDL-C) by CKD stage; **SF18s:** High Density Lipoprotein (HDL-C) by modality; **SF18t:** High Density Lipoprotein (HDL-C) by CKD stage; **SF18u:** Blood Glucose (BG) by modality; **SF18v:** Blood Glucose (BG) by CKD stage; **SF18w:** Haemoglobin (Hb) by modality; **SF18x:** Haemoglobin (Hb) by CKD stage; **SF18y:** Body Weight by modality; **SF18z:** Body Weight by CKD stage; **SF18aa:** Body Mass Index (BMI) by modality; **SF18ab:** Body Mass Index (BMI) by CKD stage; **SF18ac:** Body Fat by modality; **F18ad:** Body Fat by CKD stage; **SF18ae:** Lean Body Mass by modality; **SF18af:** Lean Body Mass by CKD stage

**SF18a** Systolic Blood Pressure (SBP) [mmHg] in people with pre-dialysis CKD by modality p=0.35:


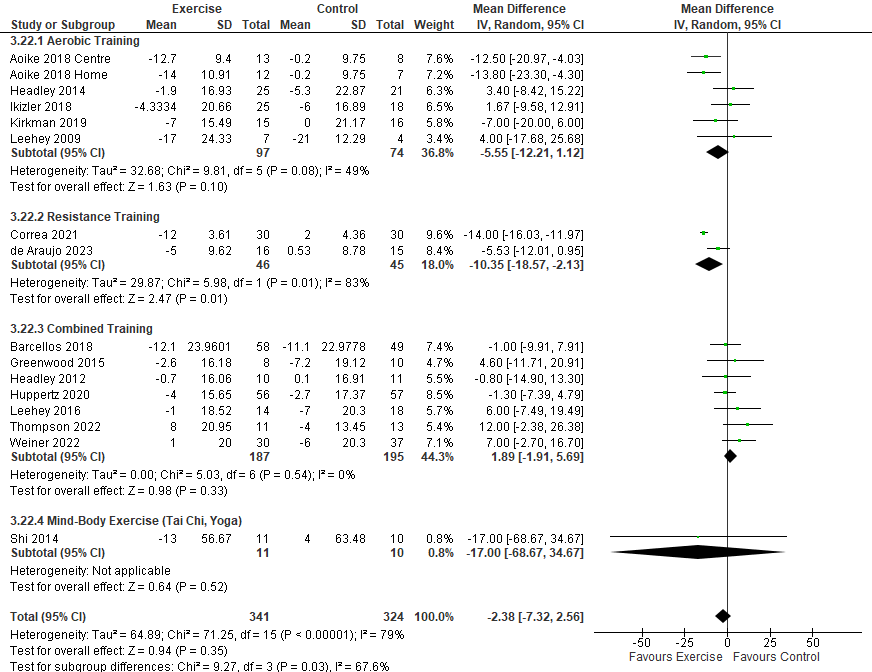


**SF18b** Systolic Blood Pressure (SBP) [mmHg] in people with pre-dialysis CKD by CKD stage p=0.35:

**
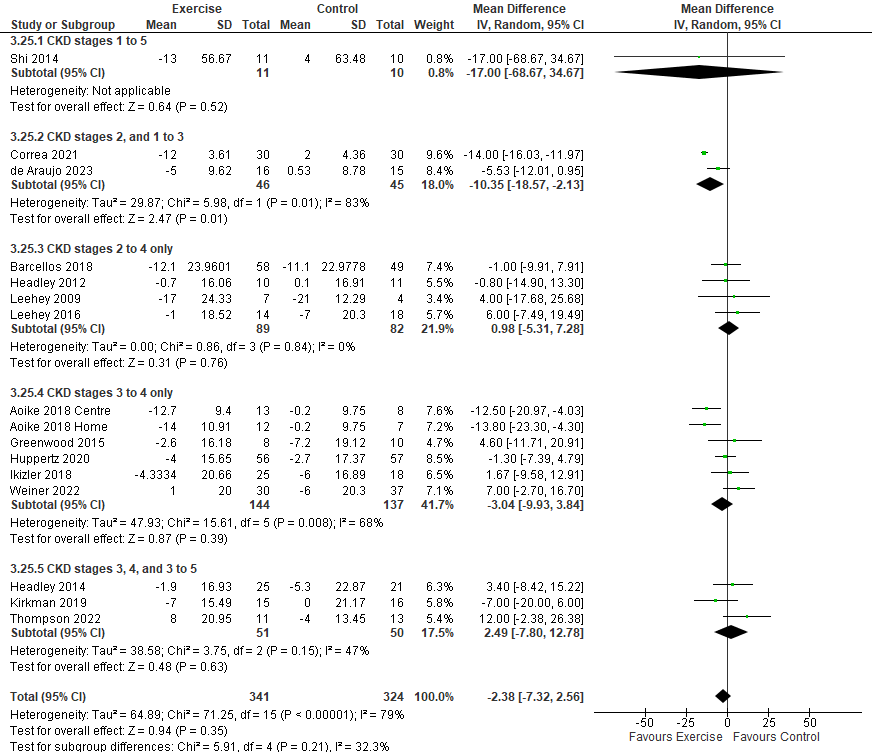
**

**SF18c** Diastolic Blood Pressure (DBP) [mmHg] in people with pre-dialysis CKD by modality p=0.32:


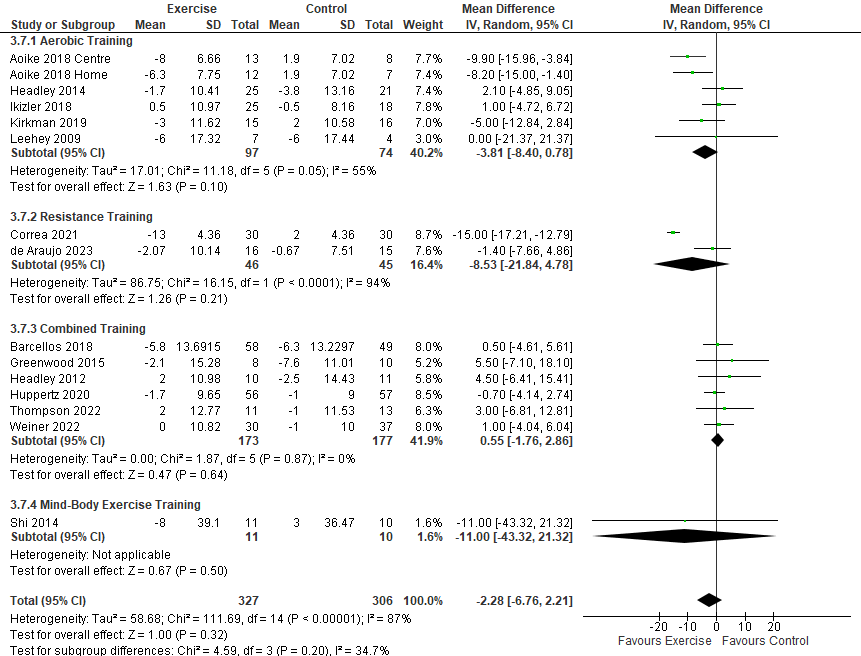


**SF18d** Diastolic Blood Pressure (DBP) [mmHg] in people with pre-dialysis CKD by CKD stage p=0.32:

**
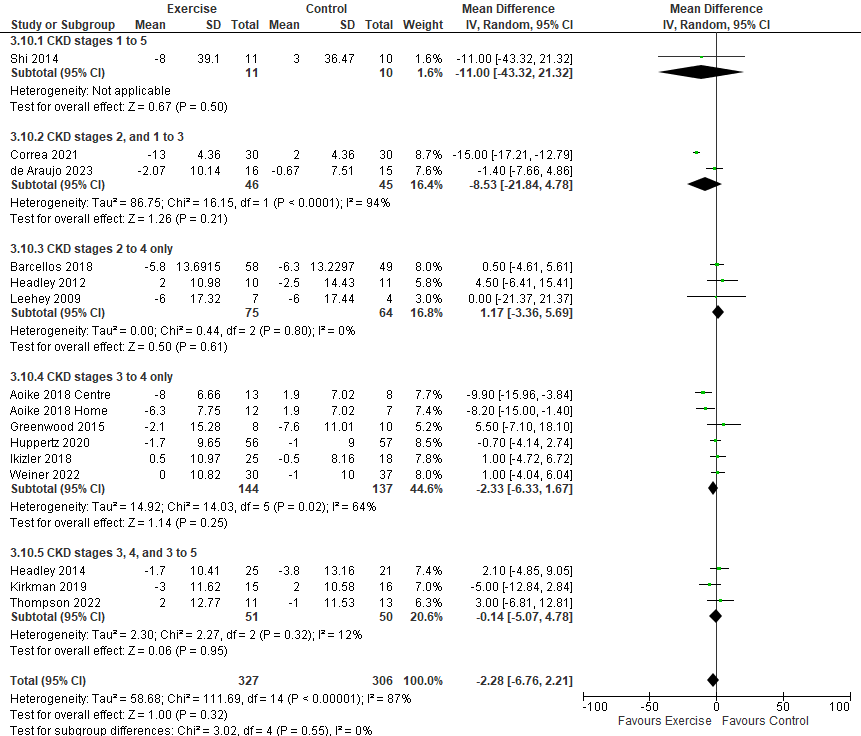
**

**SF18e** Ambulatory 24-hour Systolic Blood Pressure [mmHg] in people with pre-dialysis CKD by modality p=0.97:
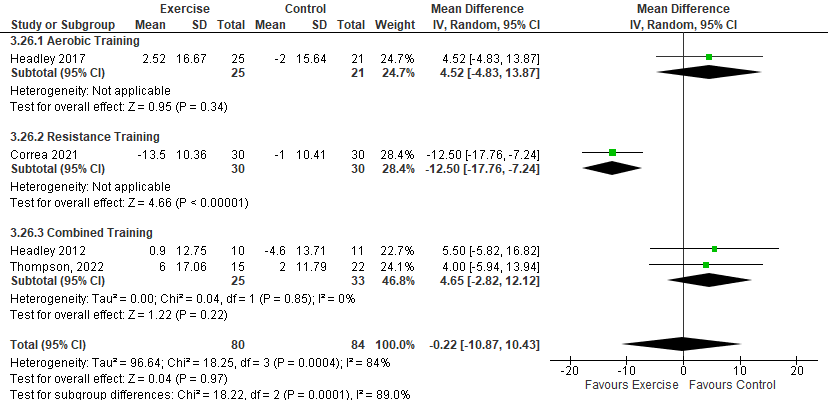


**SF18f** Ambulatory 24-hour Systolic Blood Pressure [mmHg] in people with pre-dialysis CKD by CKD stage p=0.97:

**
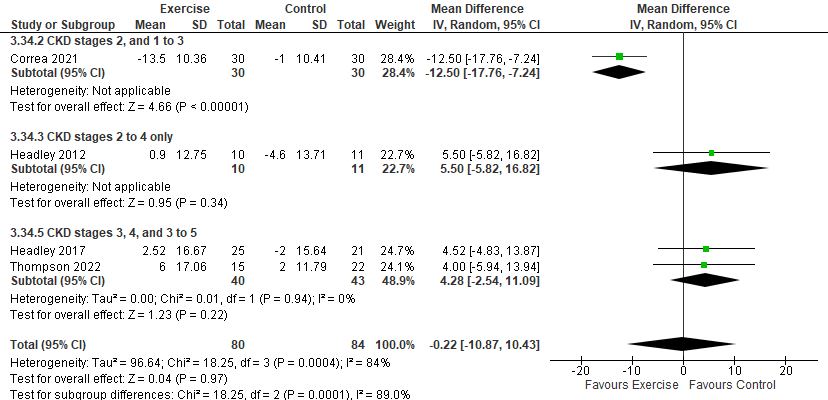
**

**SF18g** Ambulatory 24-hour Diastolic Blood Pressure [mmHg] in people with pre-dialysis CKD by modality p=0.83:

**
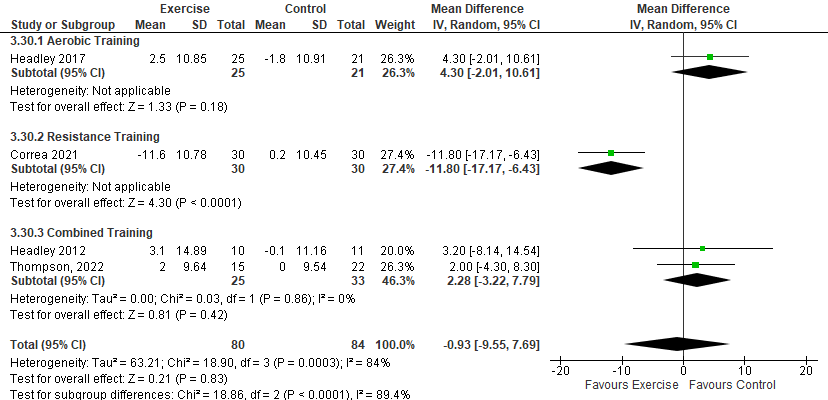
**

**SF18h** Ambulatory 24-hour Diastolic Blood Pressure [mmHg] in people with pre-dialysis CKD by CKD stage p=0.83:**
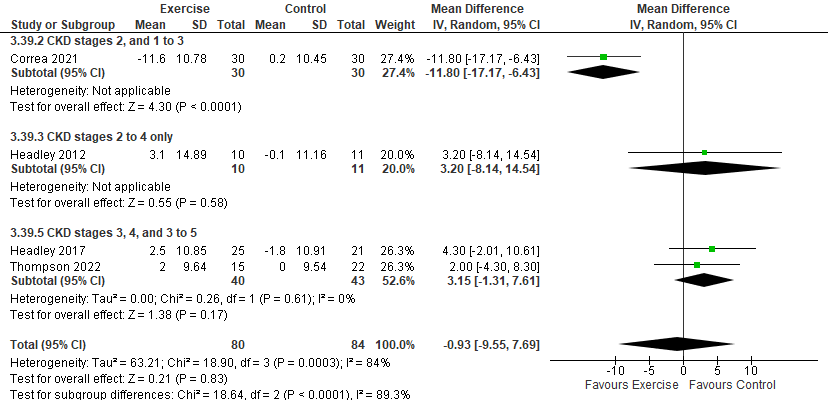
**

**S19i** Pulse Wave Velocity (PWV) aortic [m/s] in people with pre-dialysis CKD by modality p=0.80:


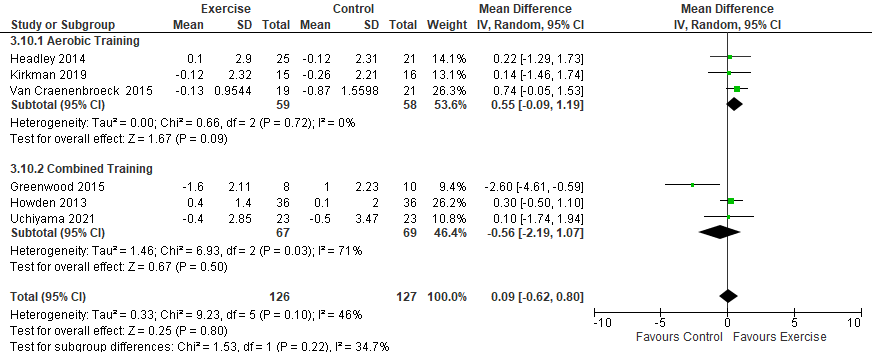


**SF18j** Pulse Wave Velocity (PWV) aortic [m/s] in people with pre-dialysis CKD by CKD stage p=0.80:

**
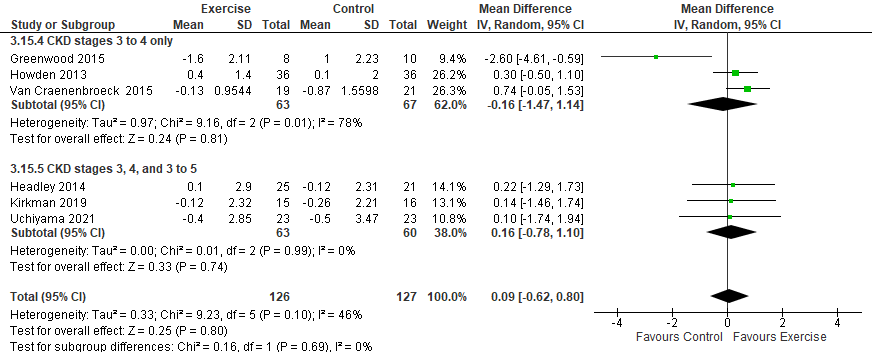
**

**SF18k** Augmentation Index (AIx) central arterial [(%] in people with pre-dialysis CKD by modality p=0.25:
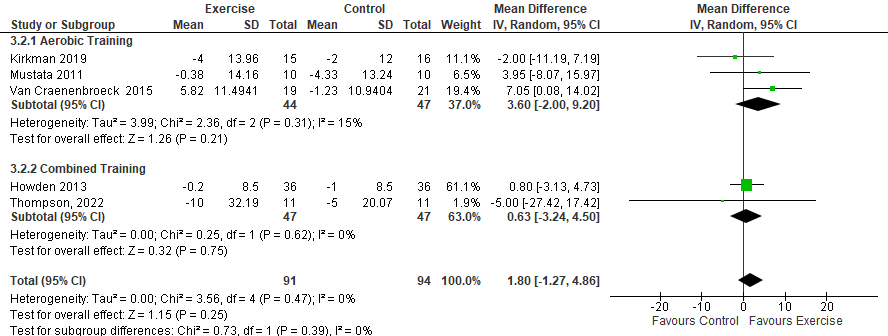


**SF18l** Augmentation Index (AIx) central arterial [(%] in people with pre-dialysis CKD by CKD stage p=0.25:

**
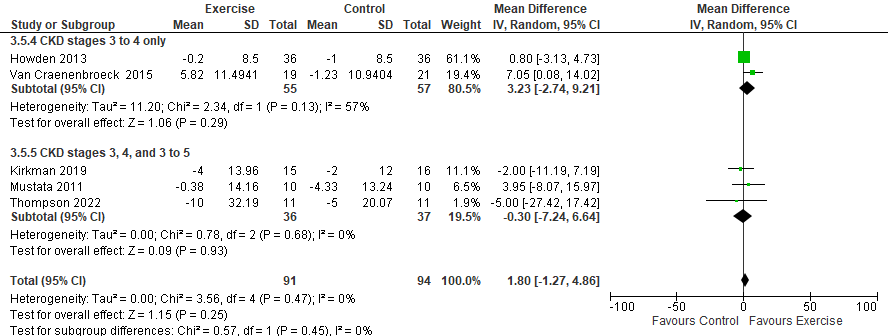
**

**SF18m** Asymmetric dimethylarginine (ADMA) [*u*mol/L] in people with pre-dialysis CKD by modality p=0.07:

**
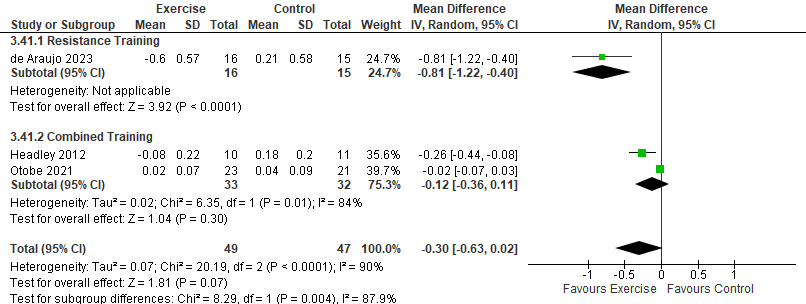
**

**SF18n** Asymmetric dimethylarginine (ADMA) [*u*mol/L] in people with pre-dialysis CKD by CKD stage p=0.07:**
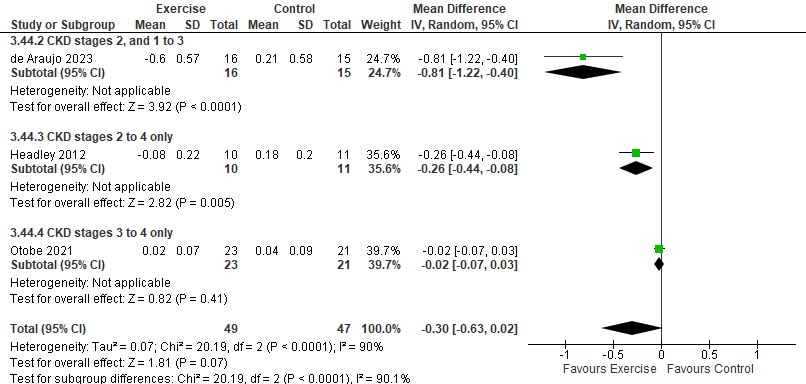
**

**SF18o** Total Cholesterol (TC) [mg/dL] in people with pre-dialysis CKD by modality p=0.34:

**
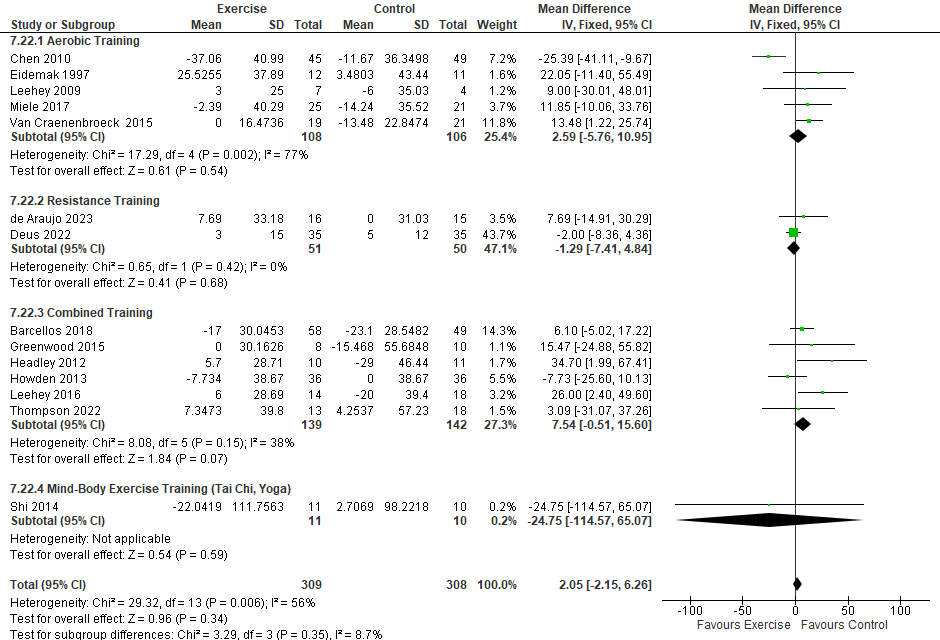
**

**SF18p** Total Cholesterol (TC) [mg/dL] in people with pre-dialysis CKD by CKD stage p=0.34:

**
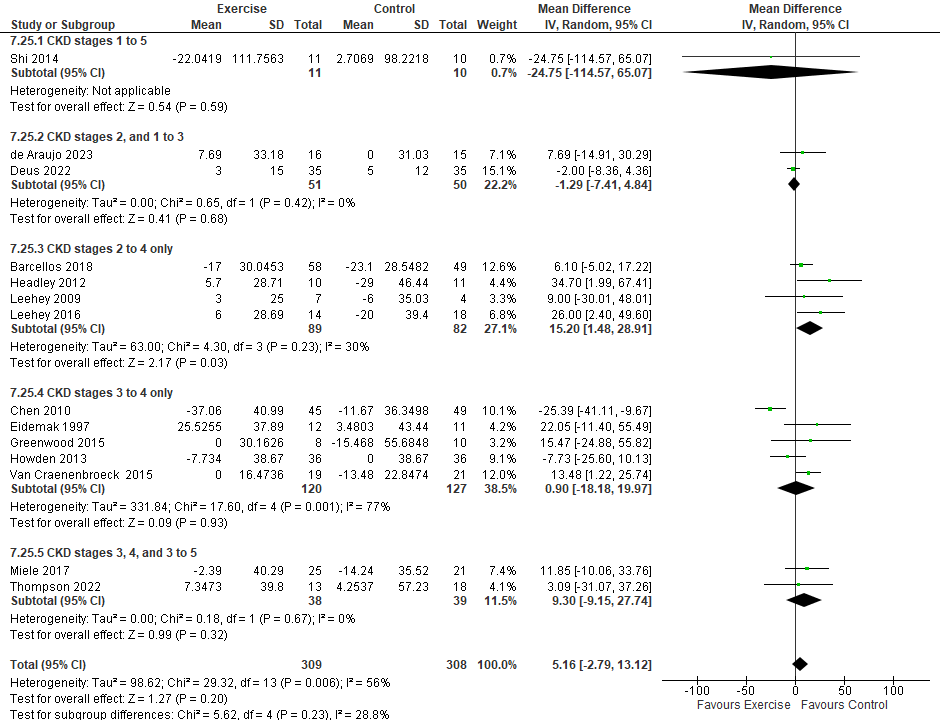
**

**SF18q** Low Density Lipoprotein (LDL-C) [mg/dL] in people with pre-dialysis CKD by modality p=0.06:**
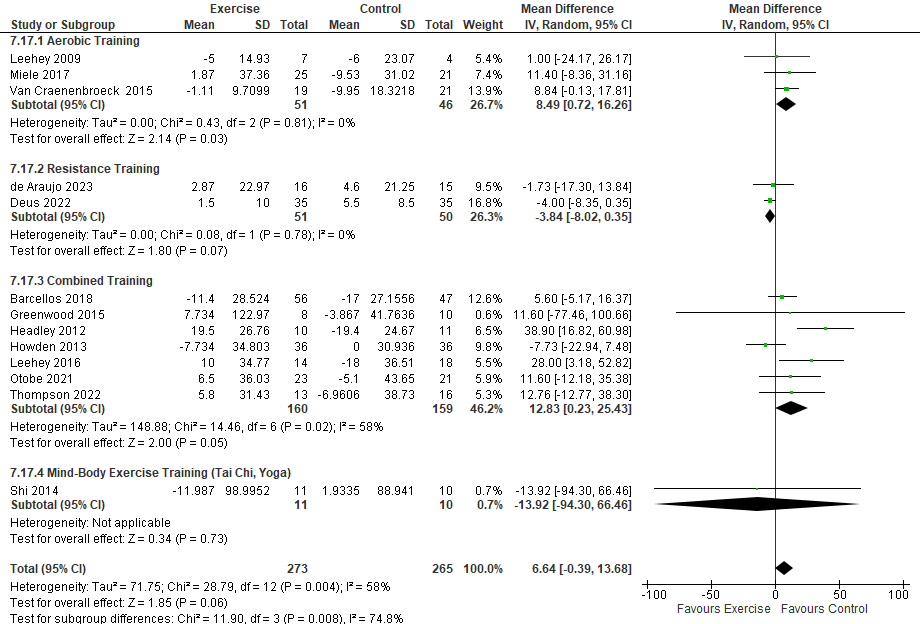
**

**SF18r** Low Density Lipoprotein (LDL-C) [mg/dL] in people with pre-dialysis CKD by CKD stage p=0.06:

**
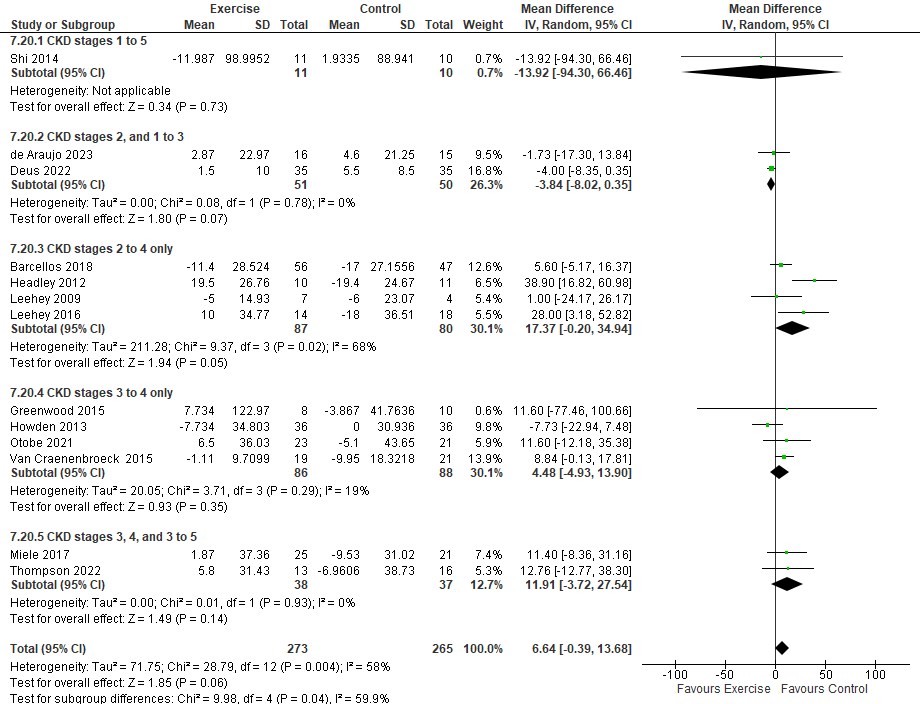
**

**SF18s** High Density Lipoprotein (HDL-C) [mg/dL] in people with pre-dialysis CKD by modality p=0.11:

**
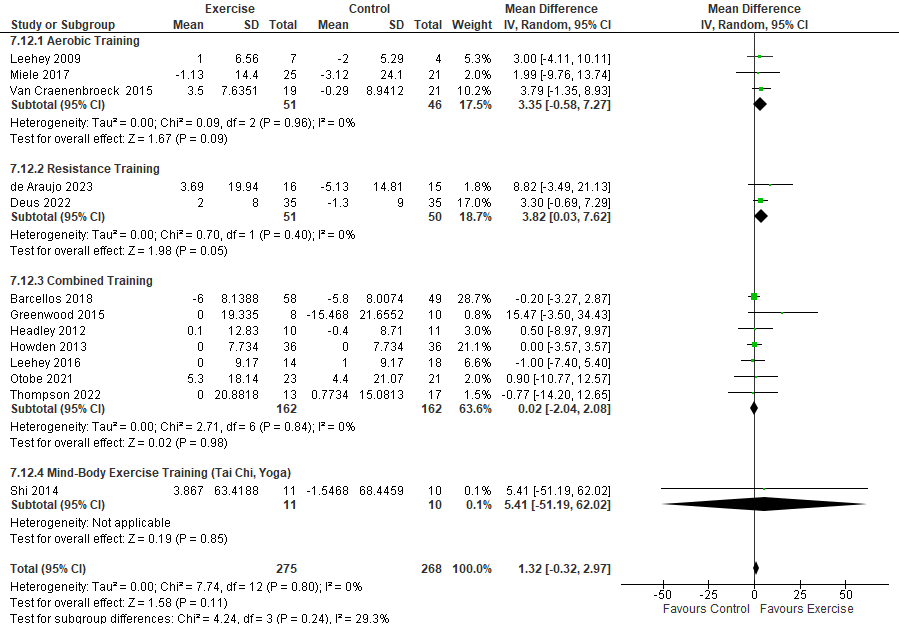
**

**SF18t** High Density Lipoprotein (HDL-C) [mg/dL] in people with pre-dialysis CKD by CKD stage p=0.11:

**
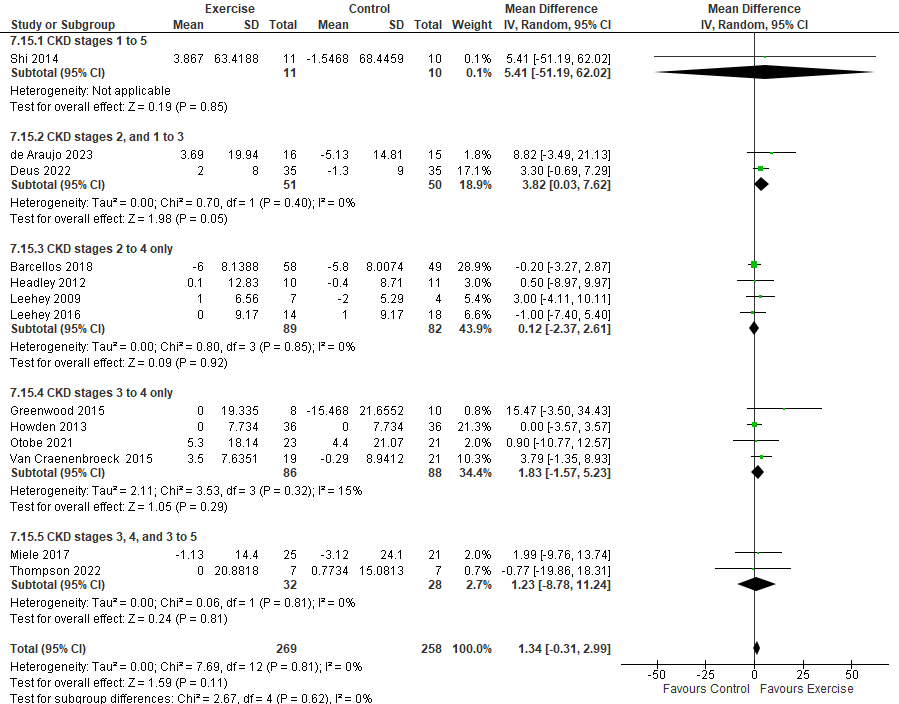
**

**SF18u** Blood Glucose (BG) [mg/dL] in people with pre-dialysis CKD by modality p=0.29:


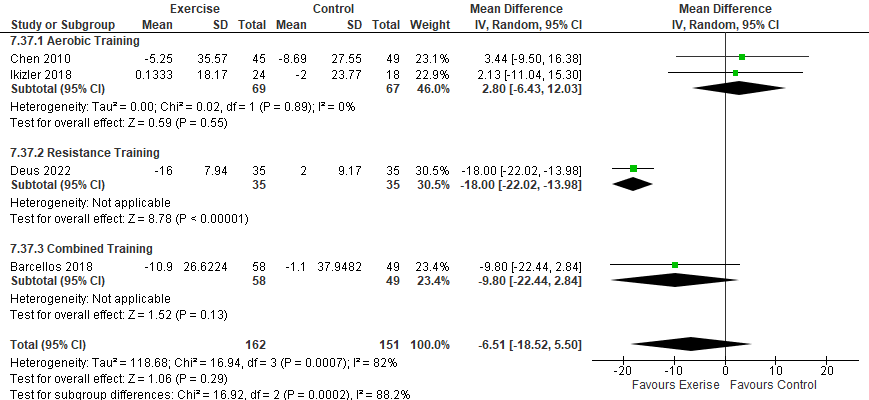


**SF18v** Blood Glucose (BG) [mg/dL] in people with pre-dialysis CKD by CKD stage p=0.29:**
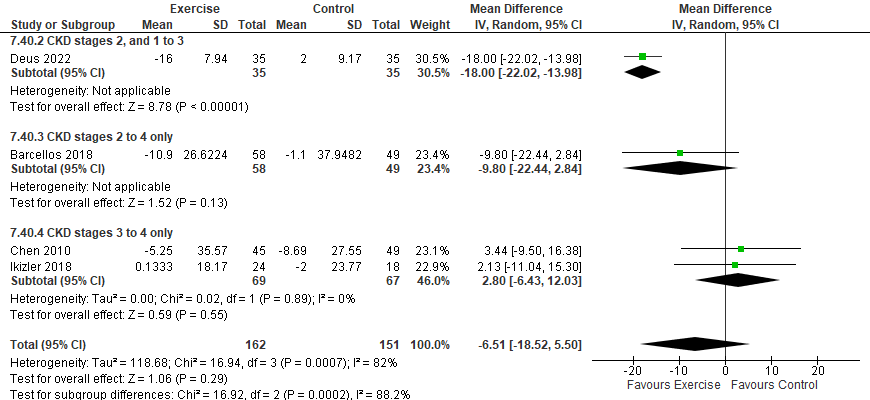
**

**SF18w** Haemoglobin (Hb) [g/dL] in people with pre-dialysis CKD by modality p=0.08:

**
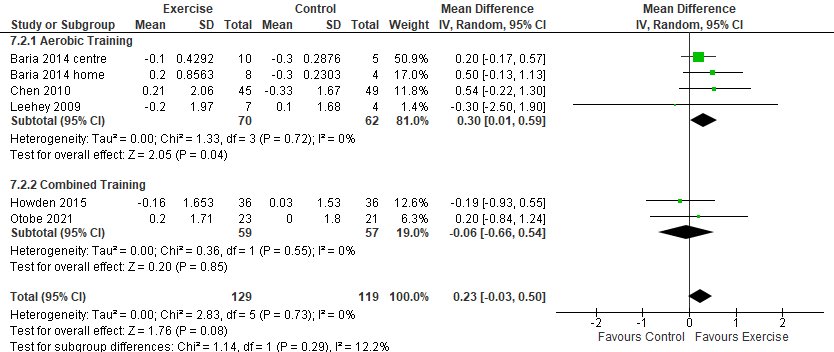
**

**SF18x** Haemoglobin (Hb) [g/dL] in people with pre-dialysis CKD by CKD stage p=0.08:**
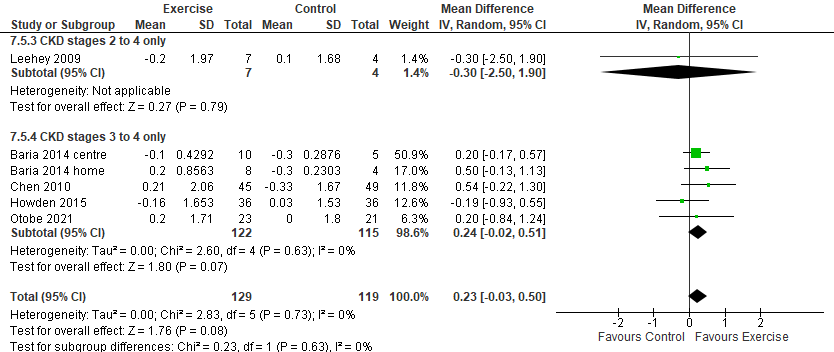
**

**SF18y** Body Weight [kg] in people with pre-dialysis CKD by modality p=0.62:
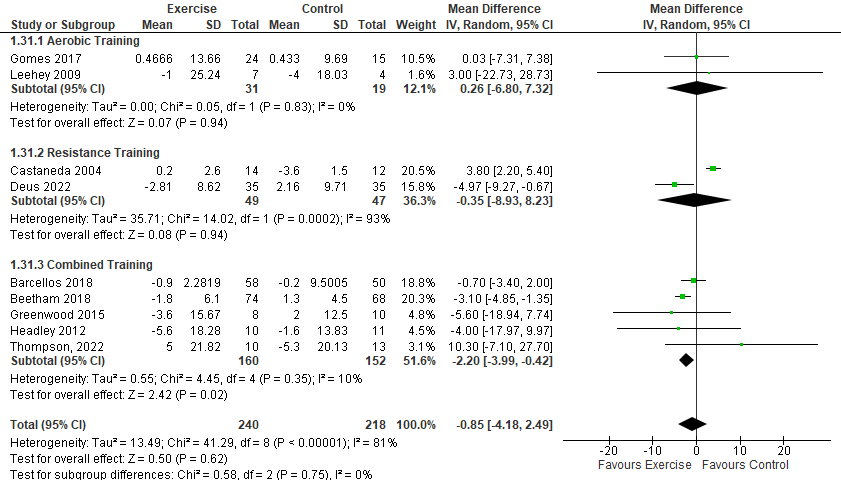


**SF18z** Body Weight [kg] in people with pre-dialysis CKD by CKD stage p=0.62:

**
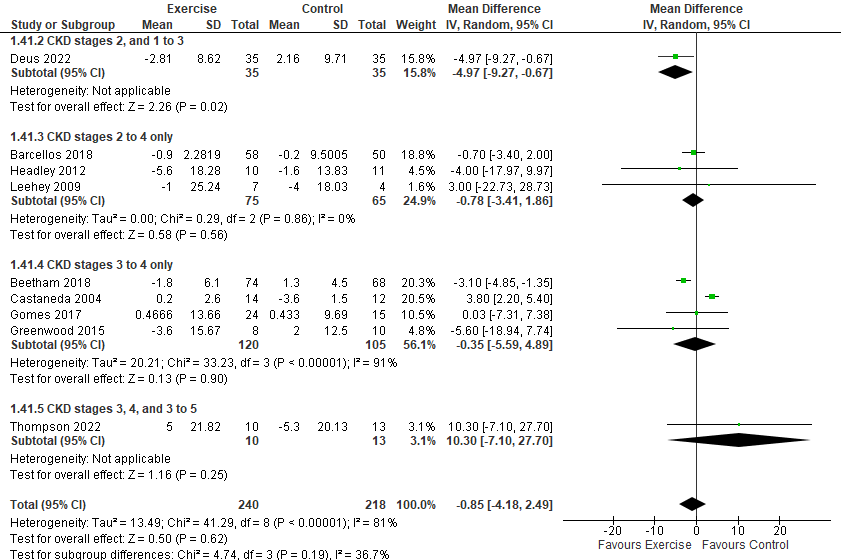
**

**SF18aa** Body Mass Index (BMI) [kg/m^2^] in people with pre-dialysis CKD by modality p=0.18:
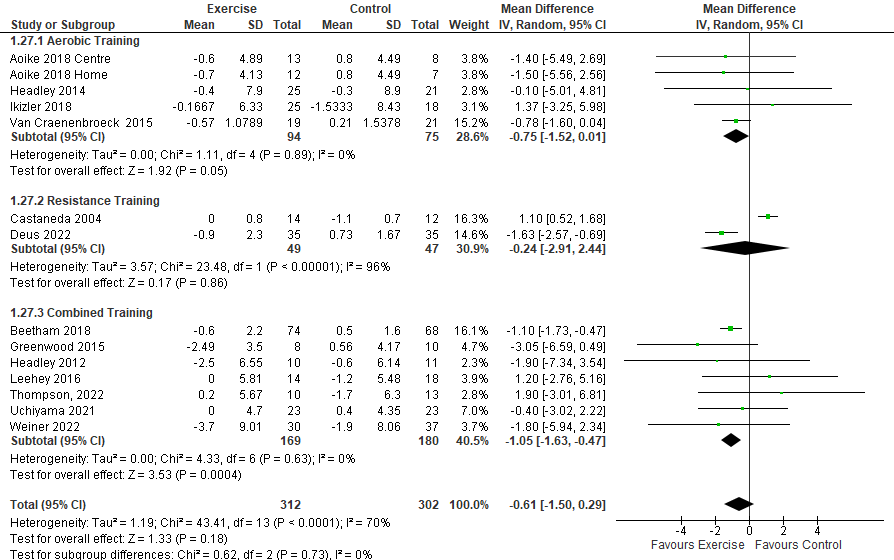


**SF18ab** Body Mass Index (BMI) [kg/m^2^] in people with pre-dialysis CKD by CKD stage p=0.18:

**
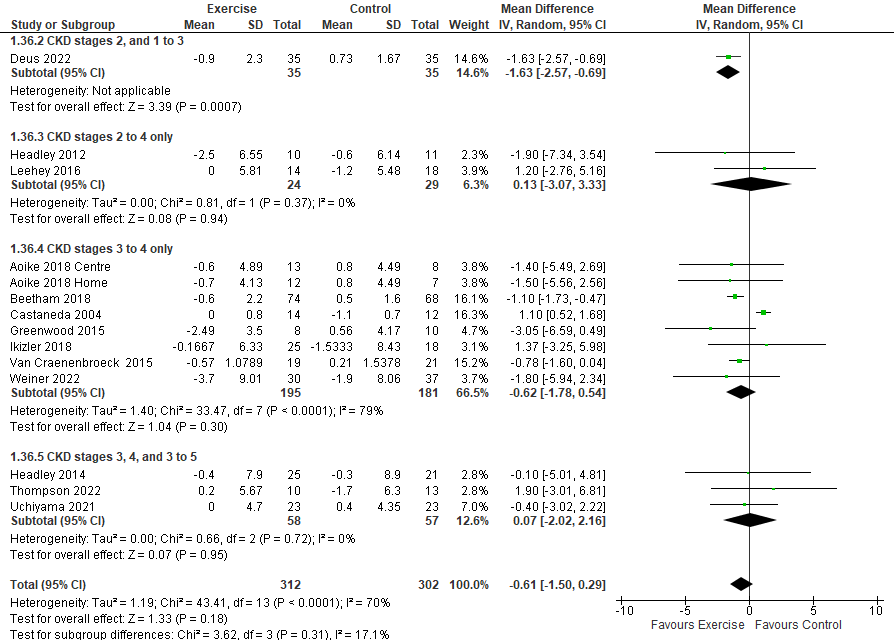
**

**SF18ac** Body Fat [%] in people with pre-dialysis CKD by modality p=0.09:
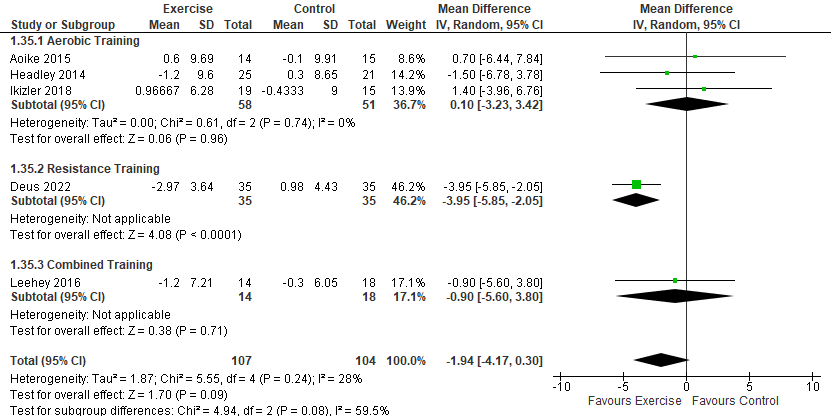


**SF18ad** Body Fat [%] in people with pre-dialysis CKD by CKD stage p=0.09:

**
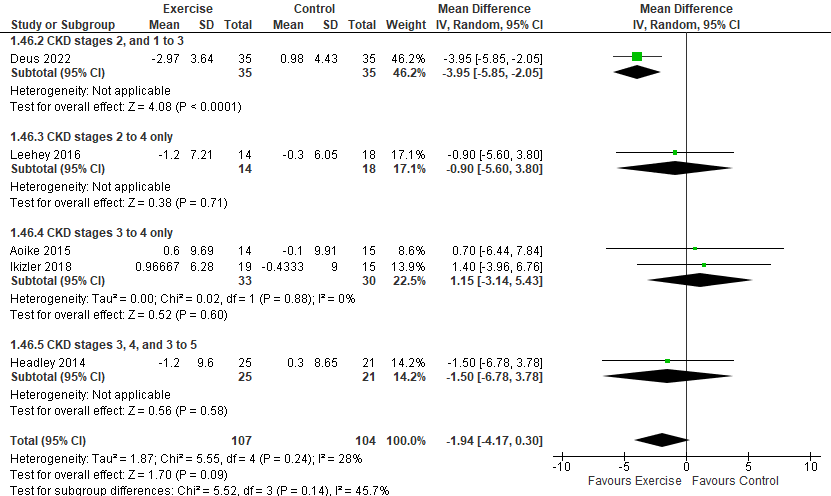
**

**SF18ae** Lean Body Mass [kg] in people with pre-dialysis CKD by modality p=0.06:


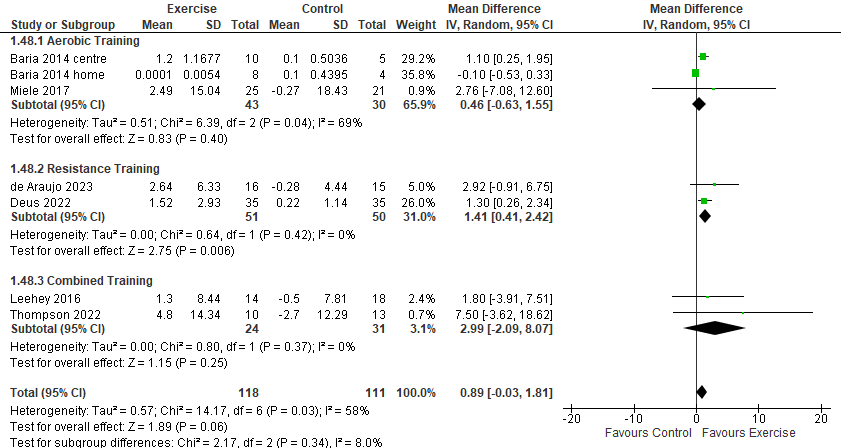


**SF18af** Lean Body Mass [kg] in people with pre-dialysis CKD by CKD stage p=0.06:
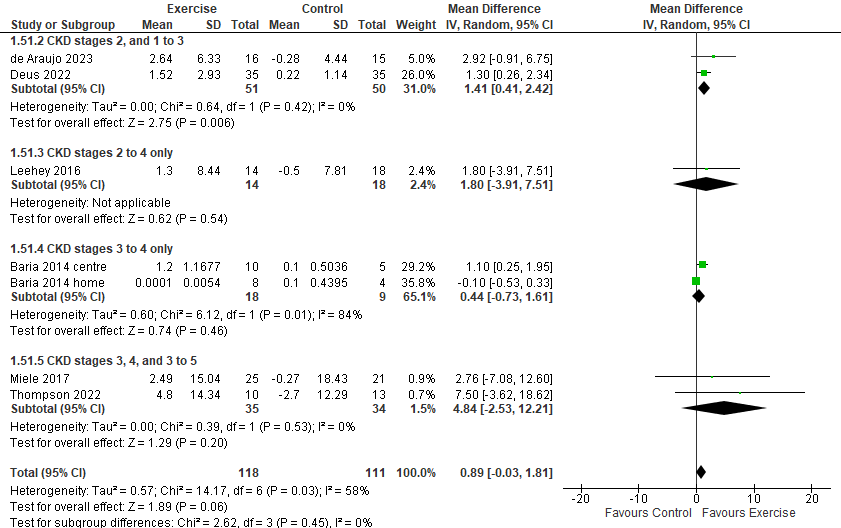


**Supplemental Figure SF19** Forest plot for non-significant Inflammatory Markers – exercise versus usual care**:**

**SF19a:** C-Reactive Protein (CRP) by modality; **SF19:** C-Reactive Protein (CRP) by CKD stage

**SF19a** C-Reactive Protein (CRP) [mg/L] {hs-CRP and non hs-CRP combined} in people with pre-dialysis CKD by modality p=0.92:

**
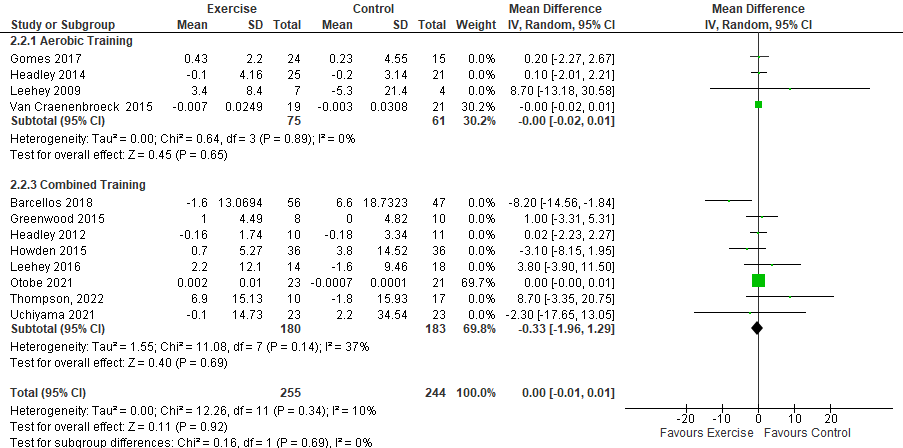
**

**SF19b** C-Reactive Protein (CRP) [mg/L] {hs-CRP and non hs-CRP combined} in people with pre-dialysis CKD by CKD stage p=0.92:


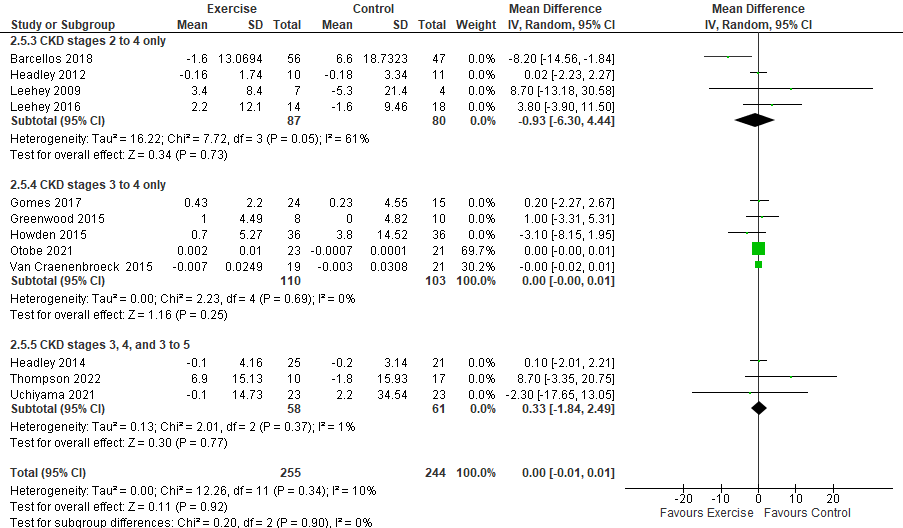

Supplement: Supplementary file 3 — Supplementary file3 (DOCX 1208 KB) [file 40620_2024_2081_MOESM3_ESM.docx]
