# Supplementary material for: The effect of exercise training in people with pre-dialysis chronic kidney disease: a systematic review with meta-analysis
Source: J Nephrol. 2024 Oct 17;37(8):2063–98. doi: 10.1007/s40620-024-02081-9 (PMC11649798; doi:10.1007/s40620-024-02081-9)

**The effect of exercise training in people with pre-dialysis chronic kidney disease. A systematic review with meta-analysis.**

Annette Traise*, Gudrun Dieberg, Melissa J Pearson, Neil A Smart

Clinical Exercise Physiology, School of Science and Technology, University of New England, NSW 2351, Australia

* Corresponding author

**Online Resource 8**

**Supplemental material: Figures 20 – 21** Sensitivity analyses upon removal of one study

**Supplemental Figure 20** Loss of significance upon removal of one study

**Supplemental Figure 21** Gain of significance upon removal of one study

**Figure SF20** Forest plot for sensitivity analyses showing *loss of significance* upon removal of one study (exercise versus usual care)

**SF20a:** Timed up and go test; **SF20b:** Sit to stand test; **SF20c:** Quality of life SF-36 General health; **SF20d:** Quality of life SF-36 General health; **SF20e:** Quality of life SF-36 Mental component summary; **SF20f:** Estimated glomerular filtration rate; **SF20g:** Serum cystatin-C; **SF20h:** Resting heart rate; **SF20i:** Resting heart rate; **SF20j:** Resting heart rate; **SF20k:** Glycosylated haemoglobin; **SF20l:** Glycosylated haemoglobin; **SF20m:** Glycosylated haemoglobin; **SF20n:** Glycosylated haemoglobin; **SF20o:** Glycosylated haemoglobin

**SF20a** Timed up and go test (rise from a chair, walk three meters, and sit back) [p=0.007] – with Barcellos 2018 [1] removed p=0.07:

**
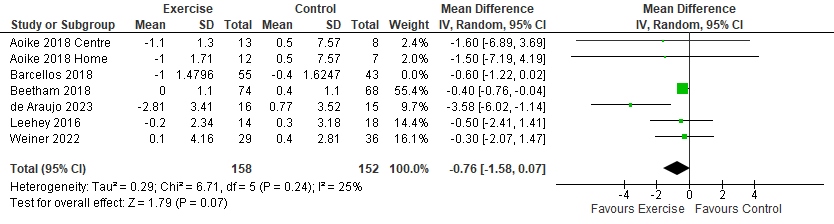
**

**SF20b** Sit to stand test [p=0.004] – with Barcellos 2018 [1] removed=0.06:

**
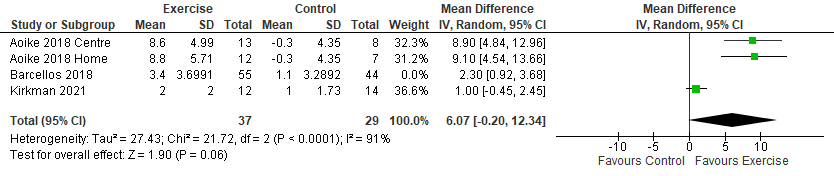
**

**SF20c** Quality of life SF-36 General health [p=0.05] with Headley 2014 [2] removed p=0.15:


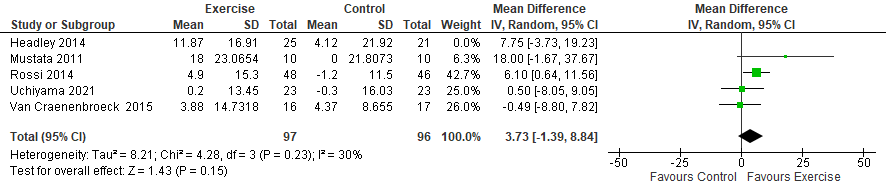


**SF20d** Quality of life SF-36 General health [p=0.05] with Rossi 2014 [3] removed p=0.29:

**
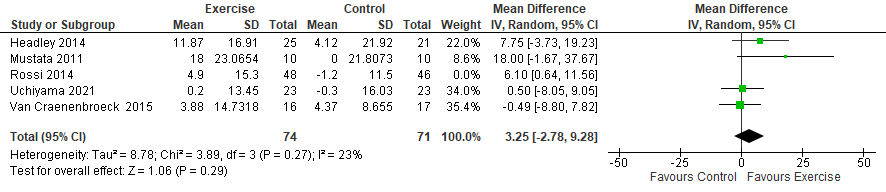
**

**SF20e** Quality of life SF-36 Mental component summary [p=0.03] – with Tang 2017 [4] removed p=0.52:

**
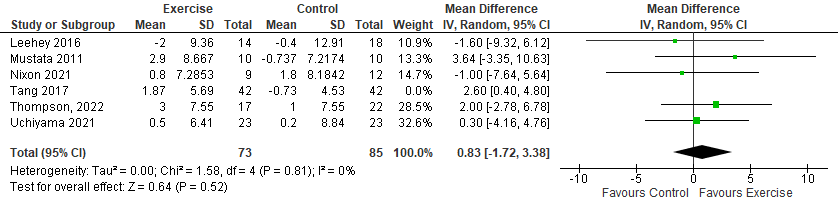
**

**SF20f** Estimated glomerular filtration rate [p=0.001] – with Deus 2022 [10] removed p=0.06:

**
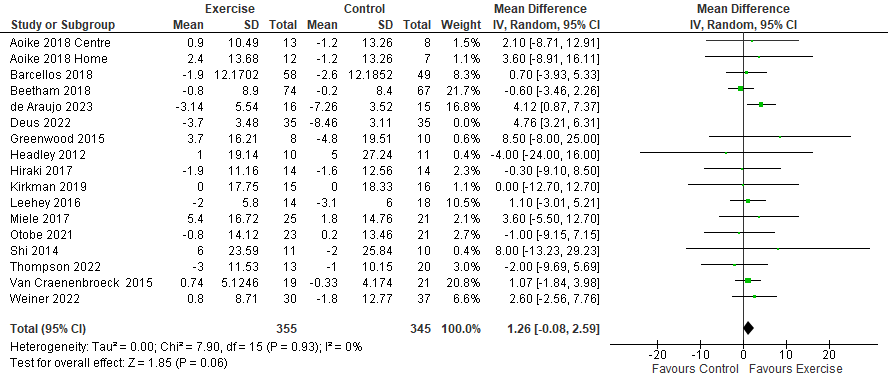
**

**SF20g** Serum cystatin-C [p=0.004] – with de Araujo 2023 [9] removed p=0.15:


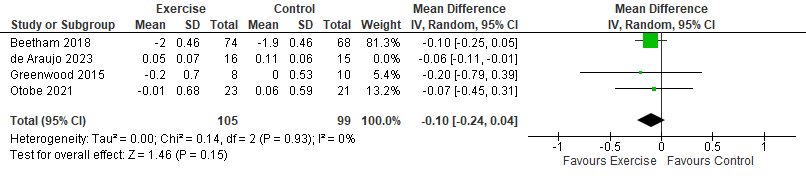


**SF20h** Resting heart rate (bpm) [p=0.04] – with Aoike 2015 [5] removed p=0.09:

**
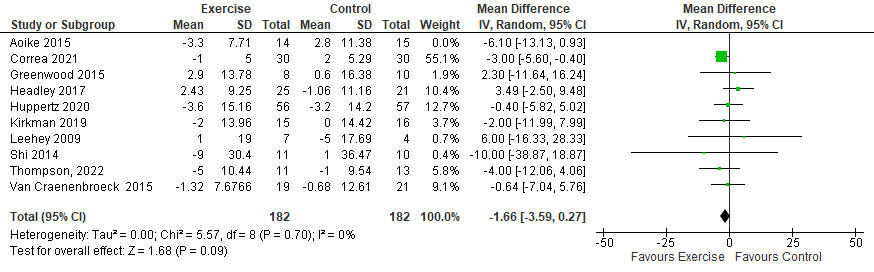
**

**SF20i** Resting heart rate (bpm) [p=0.04] – with Correa 2021 [6] removed p=0.51:

**
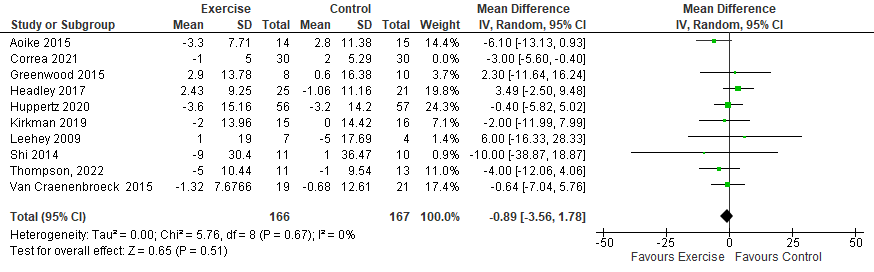
**

**SF20j** Resting heart rate (bpm) [p=0.04] – with Thompson 2022 [7] removed p=0.06:

**
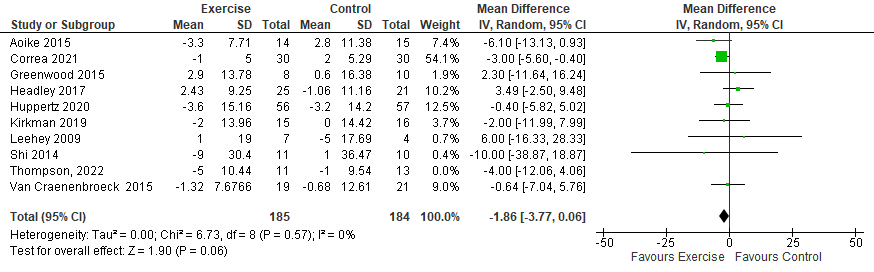
**

**SF20k** Glycosylated haemoglobin (%) [p=0.04] – with Aoike 2018 [8] Centre based intervention group removed p=0.09:

**
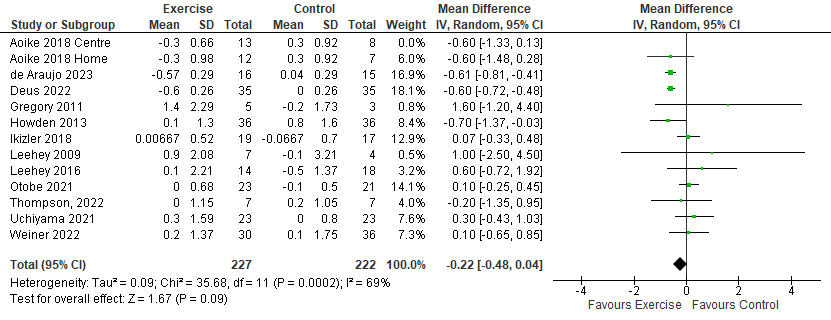
**

**SF20l** Glycosylated haemoglobin (%) [p=0.04] – with Aoike 2018 [8] Home based intervention group removed p=0.08:

**
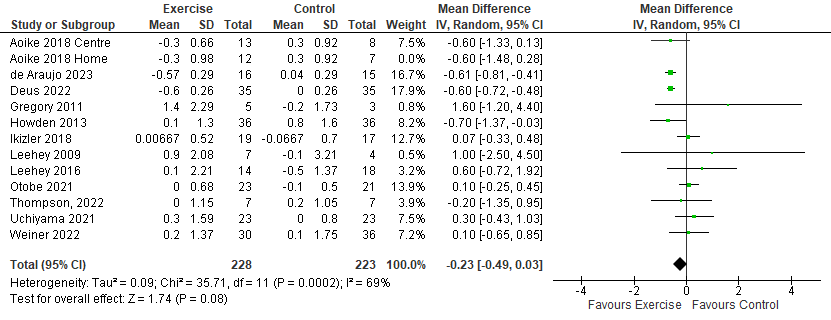
**

**SF20m** Glycosylated haemoglobin (%) [p=0.04] – with de Araujo 2023 [9] removed p=0.29:


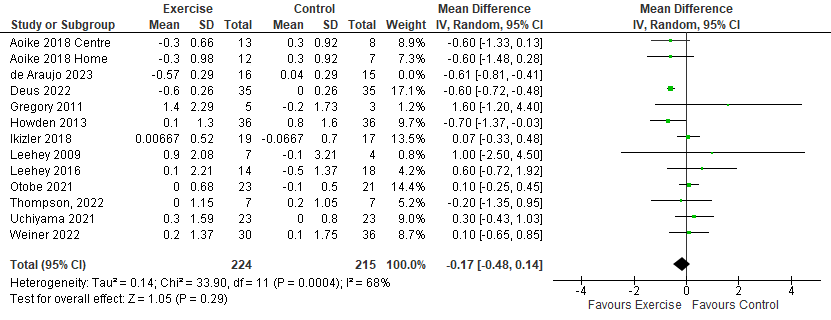


**SF20n** Glycosylated haemoglobin (%) [p=0.04] – with Deus 2022 [10] removed p=0.26:

**
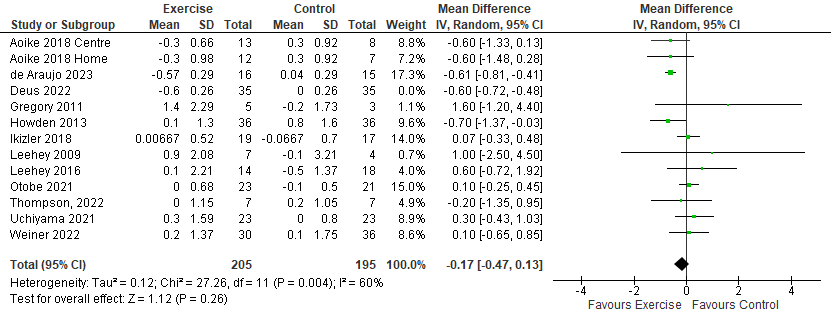
**

**SF20o** Glycosylated haemoglobin (%) [p=0.04] – with Howden 2013 [11] removed p=0.11:

**
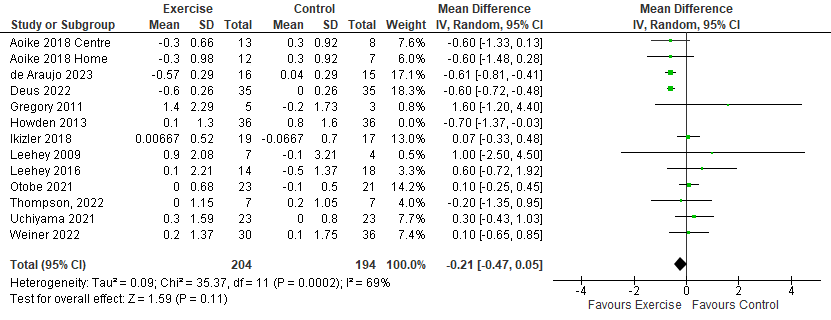
**

**Figure SF21** Sensitivity analyses showing *significance* upon removal of one study

**SF21a:** Handgrip force; **SF21b:** Quality of life SF-36 Physical component summary; **SF21c:** Serum creatinine; **SF21d:** Albumin; **SF21e:** Total cholesterol; **SF21f:** Low-density lipoprotein; **SF21g:** Low-density lipoprotein; **SF21h:** Low-density lipoprotein; **SF21i:** High-density lipoprotein; **SF21j:** Haemoglobin; **SF21k:** Body Weight; **SF21l:** Body Mass Index; **SF21m:** Body fat; **SF21n** Lean body mass

**SF21a** Handgrip force (kg) [p=0.13] – with Beetham 2018 [12] removed p=0.04:


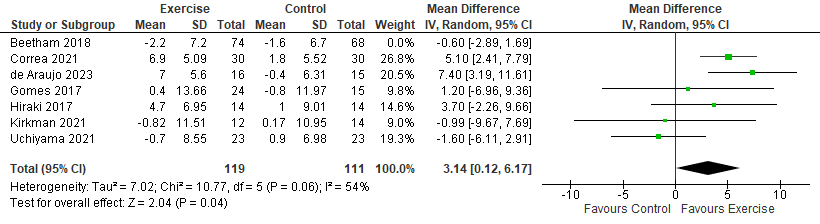


**SF21b** Quality of life SF-36 Physical component summary [p=0.22] – with Nixon 2021 [13] removed p=0.02:

**
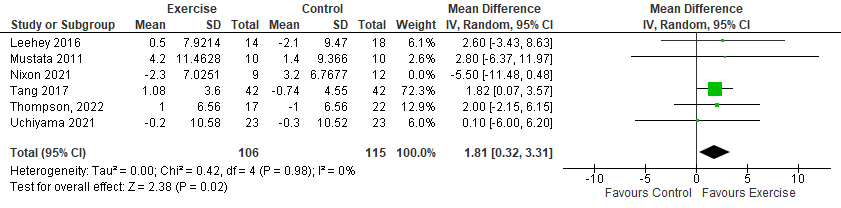
**

**SF21c** Serum creatinine [p=0.39] – with Beetham 2018 [12] removed p=0.04:


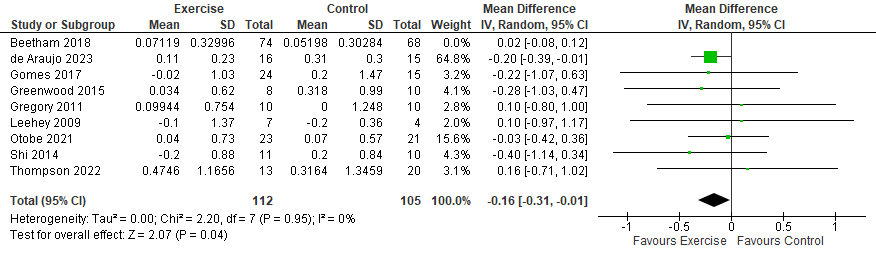


**SF21d** Albumin [p=0.16] – with Aoike 2015 [5] removed p=0.05:


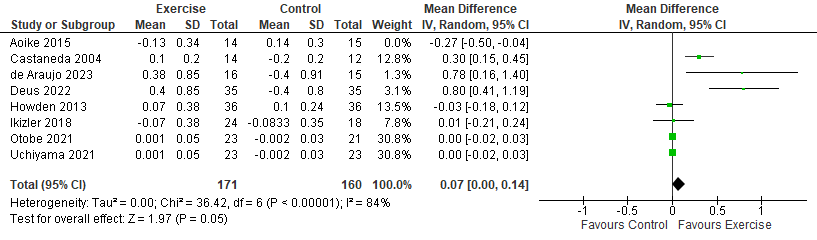


**SF21e** Total cholesterol [p=0.20] – with Chen 2010 [18] removed p=0.03:


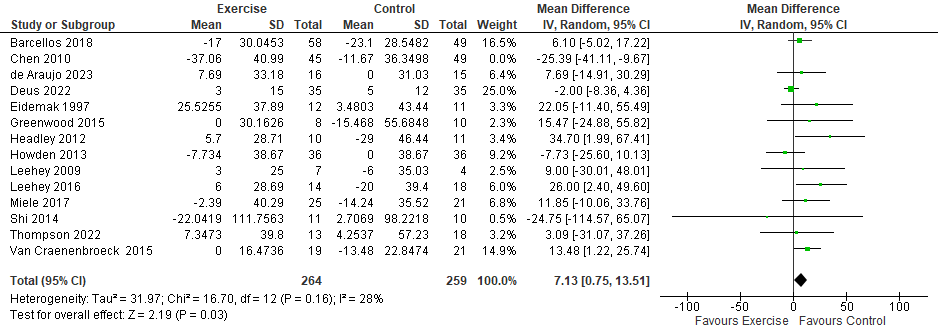


**SF21f** Low-density lipoprotein [p=0.06] – with de Araujo 2023 [9] removed p=0.05:


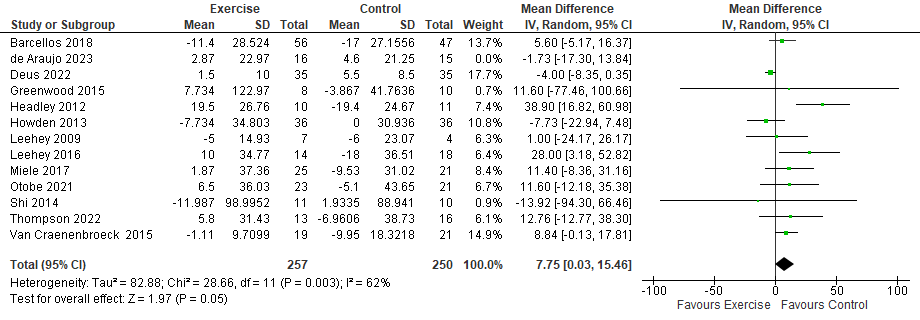


**SF21g** Low-density lipoprotein [p=0.06] – with Deus 2023 [10] removed p=0.02:
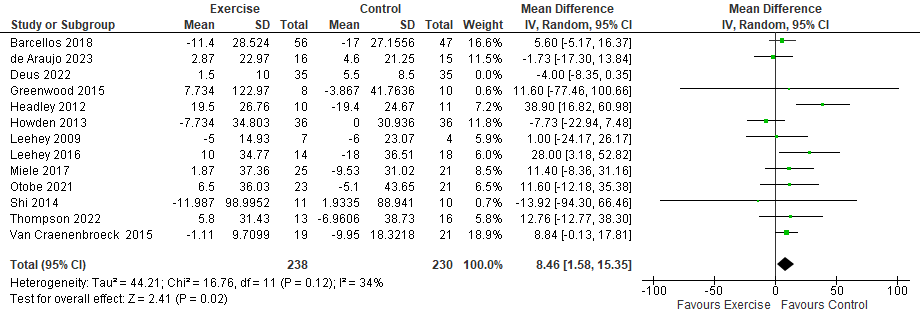


**SF21h** Low-density lipoprotein [p=0.06] – with Howden 2013 [11] removed p=0.03:


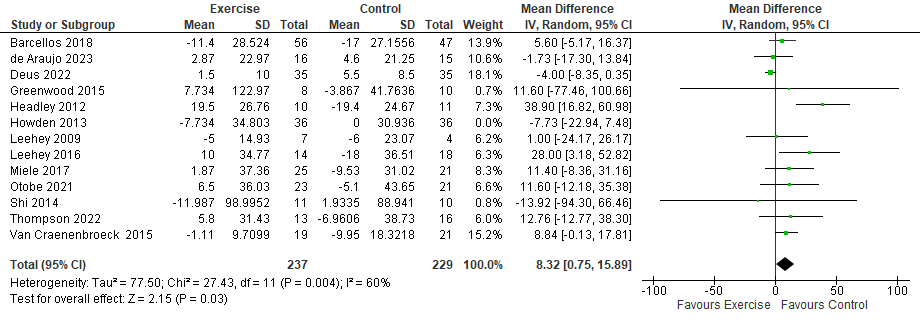


**SF21i** High-density lipoprotein [p=0.11] – with Barcellos 2018 [1] removed p=0.05:

**
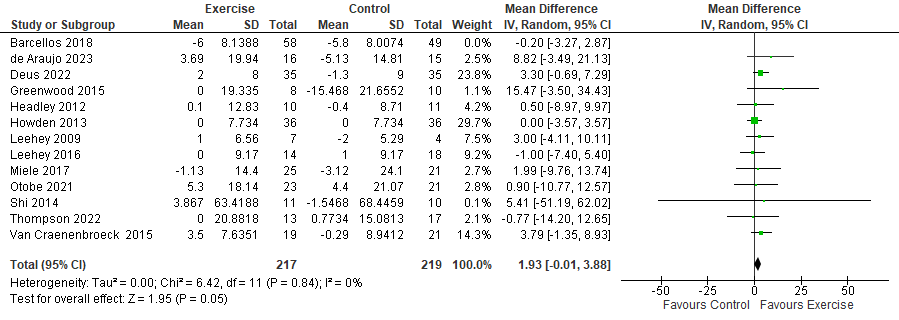
**

**SF21j** Haemoglobin (g/dL) [p=0.08] – with Howden 2015 [17] removed p=0.04:

**
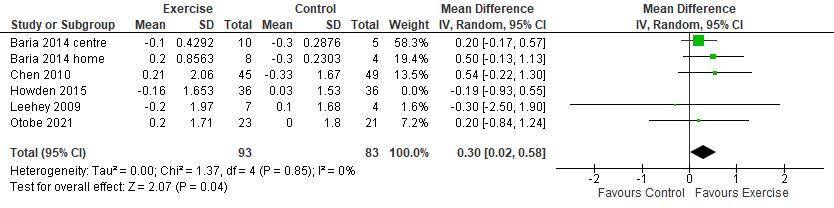
**

**SF21k** Body Weight (kg) [p=0.62] – with Castenada 2004 [14] removed p=0.0003:

**
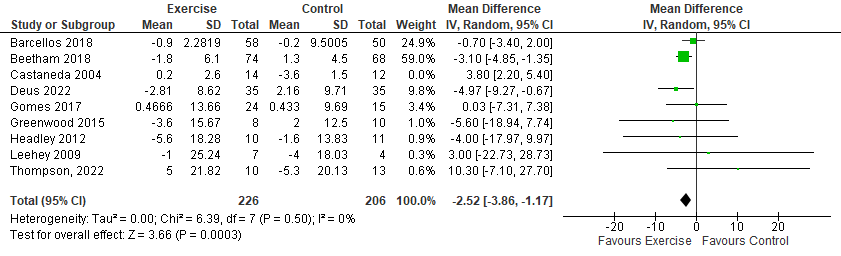
**

**SF21l** Body Mass Index (kg/m^2^) [p=0.18] – with Castenada 2004 [14] removed p<0.00001:

**
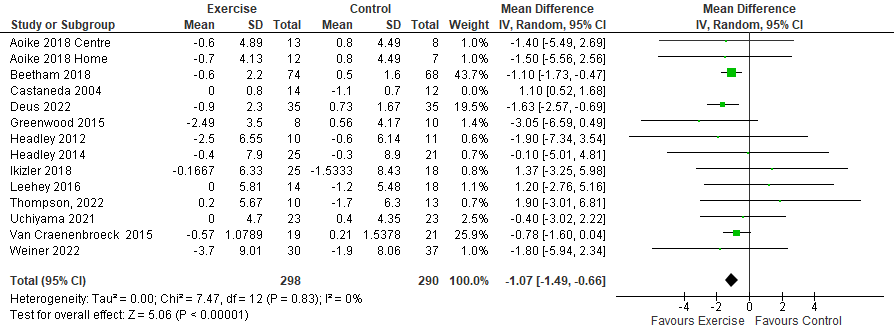
**

**SF21m** Body fat (%) [p=0.09] – with Ikizler 2018 [15] removed p=0.0004:


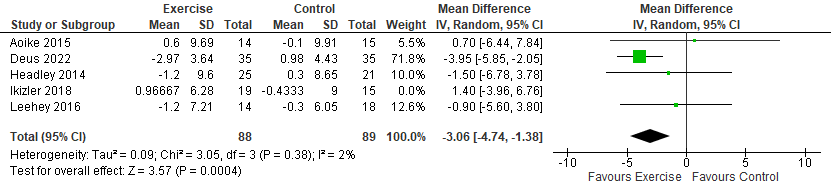


**SF21n** Lean body mass (kg) [p=0.06] – with Baria 2014 [16] home intervention group removed p=0.0001:


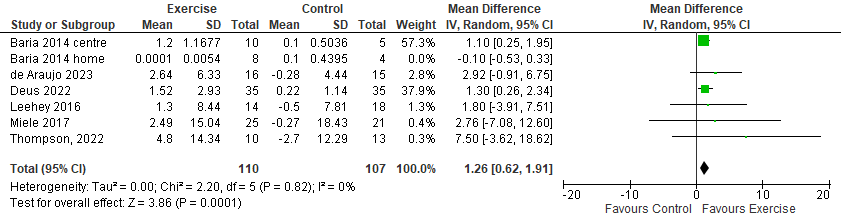

Supplement: Supplementary file 4 — Supplementary file4 (DOCX 513 KB) [file 40620_2024_2081_MOESM4_ESM.docx]
