# Supplementary material for: The effect of exercise training in people with pre-dialysis chronic kidney disease: a systematic review with meta-analysis
Source: J Nephrol. 2024 Oct 17;37(8):2063–98. doi: 10.1007/s40620-024-02081-9 (PMC11649798; doi:10.1007/s40620-024-02081-9)
Supplement: Supplementary file 5 — Supplementary file5 (DOCX 15 KB) [file 40620_2024_2081_MOESM5_ESM.docx]

**The effect of exercise training in people with pre-dialysis chronic kidney disease. A systematic review with meta-analysis.**

Annette Traise*, Gudrun Dieberg, Melissa J Pearson, Neil A Smart

Clinical Exercise Physiology, School of Science and Technology, University of New England, NSW 2351, Australia

* Corresponding author

**Online Resource 9**

**Supplemental notes:** Sub-analyses summary

**Sub-analyses summary**

***Modality*** Regarding the exercise modality sub-analyses investigated in our data extraction, a total of 39 outcomes were evaluated in the context of aerobic exercise as the intervention, with 10 of these (accounting for 26% of the outcomes) exhibiting statistically significant results. In the case of resistance training, 24 outcomes included this modality as the intervention, but it is worth noting that 11 of these were represented by one intervention study only (1SO). Although 17 of the total outcomes indicated significance (71%), ten of these were 1SO making up 58% of these significant findings. Turning to combined training, this intervention was involved in 40 outcomes including six representing 1SO, nine showed significance (23%) with two of these nine being 1SO (22%). Finally, ten outcomes were evaluated with mind/body exercise as the intervention, and none of them demonstrated significance, all stemming from a single study.

***Duration*** Our data included 33 outcomes with an intervention duration of less than 12 weeks, 12 of which included 1SO. Of these 33, eight showed significance (24%) with only one of these eight being 1SO (8%). For the outcomes with a duration of longer than 12 weeks but less than 6 months (41 in total, with four containing 1SO), 11 showed significance (27%), all of which included more than one intervention group. In the case of outcomes with a duration of longer than 6 months (36 in total, with 12 being 1SO), six showed significance (16%) and all of these six contained more than one intervention group.

***Supervision*** In our data, 38 outcomes were assessed with continued supervision, with five being of 1SO. Of these 38, 16 showed significance (42%), all of which included more than one study. There were 21 outcomes assessed as unsupervised exercise sessions, with 14 of them containing 1SO; however, only three of the 21 showed significance (14%), and 2 of these were 1SO (67%). In cases where the intervention was initially supervised and then unsupervised (34 outcomes, with three being 1SO), five showed significance (24%), and two of the five were 1SO (40%). For the outcomes with a combination of supervised and unsupervised interventions each week (30 outcomes, with 12 being 1SO), five showed significance (17%), with none of these being 1SO only.

***CKD stage*** We evaluated 10 outcomes that were assessed in studies with participants in CKD 1-5 stage, with none demonstrating significant change from baseline, and all of them being 1SO. For outcomes related to CKD 2, 1-3 (25 in total, with 16 being 1SO), 22 were significant (88%), however, 15 of these showing significance were 1SO (68%). In outcomes involving CKD 2-4 (34 outcomes, with 16 1SO), seven were significant (21%) and of these seven outcomes, three were 1SO (43%). There were 37 outcomes that included the CKD 3-4 stage, with only two being 1SO, and six showing significance, all of which included more than one intervention group, Finally, 29 outcomes involved participants with CKD 3,4, 3-5, including seven which were 1SO, and only two were significant (7%), with one of them being 1SO (50%).
