# Supplementary material for: The effect of exercise training in people with pre-dialysis chronic kidney disease: a systematic review with meta-analysis
Source: J Nephrol. 2024 Oct 17;37(8):2063–98. doi: 10.1007/s40620-024-02081-9 (PMC11649798; doi:10.1007/s40620-024-02081-9)
Supplement: Supplementary file 6 — Supplementary file6 (DOCX 217 KB) [file 40620_2024_2081_MOESM6_ESM.docx]

**The effect of exercise training in people with pre-dialysis chronic kidney disease. A systematic review with meta-analysis.**

Annette Traise*, Gudrun Dieberg, Melissa J Pearson, Neil A Smart

Clinical Exercise Physiology, School of Science and Technology, University of New England, NSW 2351, Australia

* Corresponding author

**Online Resource 2**

**Supplemental material: Table** 3 Detailed exercise intervention characteristics for included studies

**Supplemental Table 3** Detailed exercise intervention characteristics for included studies

| **Study** | **Modality** | **Duration**  **(weeks)** | **Frequency**  **(sessions/**  **week)** | **Total Time**  **per session** | **Intensity** |
| --- | --- | --- | --- | --- | --- |
| Aoike, 2015 [1] | Aerobic, walking, initially supervised then unsupervised | 12 | 3 (alternate days) | 30 min ↑ 10 min @ weeks 4 & 8 (includes 5 min WU & 5 min CD) | Mild to moderate, prescribed according to VT obtained during cardiopulmonary exercise test |
| Aoike, 2018 [2] Home | Aerobic, walking, initially 3 supervised sessions, then unsupervised | 24 | 3 (alternate days) | 30 min ↑ 10 min @ weeks 4 & 8 (includes 5 min WU & 5 min CD) | Moderate, prescribed according to VT obtained during cardiopulmonary exercise test |
| Aoike, 2018 [2] Centre | Aerobic, treadmill walking, supervised | 24 | 3 (alternate days) | 30 min ↑ 10 min @ weeks 4 & 8 (includes 5 min WU & 5 min CD) | Moderate, prescribed according to VT obtained during cardiopulmonary exercise test |
| Barcellos,  2018 [3] | Combined training. Aerobic and resistance interval training, including core and flexibility, supervised | 16 | 3 | 60 min (includes 10 WU) | Moderate, RPE, progression via ↑ effort and time, and ↓break time |
| Baria, 2014 [4]  Home | Aerobic, walking, initially 3 supervised sessions, then unsupervised | 12 | 3 | 30 min ↑ 10 min @ weeks 4 & 8 (includes 5 min WU & 5 min CD | Moderate, prescribed according to VT obtained during cardiopulmonary exercise test |
| Baria, 2014 [4]  Centre | Aerobic, treadmill walking, supervised | 12 | 3 | 30 min ↑ 10 min @ weeks 4 & 8 (includes 5 min WU & 5 min CD | Moderate, prescribed according to VT obtained during cardiopulmonary exercise test |
| Beetham, 2018 [5] | Combined training. Aerobic, treadmill, stationary bike or rowing ergometer. Resistance, machine and free weight. 8 weeks supervised at gym, then home based using TheraBand and Swiss ball, unsupervised | 52 | 2-3 (150 min) | 50 min (includes 5 min WU & 5 min CD) | Moderate with progression and RPE |
| Castaneda, 2004 [6] | Resistance, machine, supervised | 12 | 3 | 45 min (includes 5 min WU & 5 min CD) | 80% of 1 RM with progression (3 sets, 8 repetitions) |
| Chen, 2010 [7] | Aerobic, walking, unsupervised | 12 | 3-5 | 30 min | Moderate with progression guided by talk test |
| Correa, 2021 [8] | Resistance, 8 exercises on machine and free weight, supervised | 24 | 3 | 60 min | 50% of 1 RM (3 sets, 12 repetitions) x 8 weeks; 60% of 1 RM (3 sets, 10 repetitions) x 8 weeks; 70% of 1 RM (3 sets, 8 repetitions) x 8 weeks |
| de Araujo, 2023 [9] | Resistance, elastic based and body weight, supervised at home | 22 | 3 weeks 1- 7, 4 weeks 8-22 | N/S | Low ↑ Moderate with progression and RPE |
| Deus, 2022 [10] | Resistance, 8 exercises on machine and free weight, supervised | 24 | 3 | 60 min | 50% of 1 RM (3 sets, 12 repetitions) x 8 weeks; 60% of 1 RM (3 sets, 10 repetitions) x 8 weeks; 70% of 1 RM (3 sets, 8 repetitions) x 8 weeks |
| Eidemak, 1997 [11] | Aerobic, stationary bike, running, swimming, and walking, unsupervised | 78 | 7 | 30 min | Progressive to 60-75% MEC |
| Gomes, 2017 [12] | Aerobic, walking, unsupervised | 24 | 3 (alternate days) | 30 min ↑ 10 min @ weeks 4 & 8 (includes 5 min WU & 5 min CD | Moderate, prescribed according to VT obtained during cardiopulmonary exercise test |
| Greenwood, 2015 [13] | Combined training. Aerobic, stationary recumbent cycle. Resistance, TheraBand, free weights. Supervised 2 per week, unsupervised 1 per week | 52 | 3 | 2 x 20 min ↑ 1 x 40 min (includes 5 min WU & 5 min CD) | Aerobic: 80% HRR and RPE  ↑ in wattage upon a consistent reduction in RPE of 1 unit. Resistance: 80% of 1 RM (up to 3 sets, 8-10 repetitions) |
| Gregory, 2011 [14] | Combined training. Aerobic, cycling, treadmill, elliptical and/or Stairmaster, supervised. Resistance (added after week 24), machine weights | 48 | 3 | 10 min ↑ 45 min (includes 5 min WU & 5 min CD) | Aerobic: low intensity ↑ to moderate (50%–60% Peak VO_2_)  Resistance exercise: (twice per week in addition to aerobic) (2 sets, 10-15 repetitions) |
| Headley, 2012 [15] | Combined training. Aerobic, cycling, treadmill, elliptical and/or Stairmaster, supervised. Resistance (added after week 24), machine weights | 48 | 3 | 10 min ↑ 45 min (includes 5 min WU & 5 min CD) | Aerobic: low intensity ↑ to moderate (50%–60% Peak VO_2_)  Resistance exercise: (twice per week in addition to aerobic) (2 sets, 10-15 repetitions) |
| Headley, 2014 [16] | Aerobic, various apparati, supervised | 16 | 3 | 15-30 min ↑ 55 min (includes 5 min WU & 5 min CD) | Moderate (50%-60% Peak VO_2_) and RPE |
| Headley, 2017 [17] | Aerobic, various apparati, supervised | 16 | 3 | 15-30 min ↑ 55 min (includes 5 min WU & 5 min CD) | Moderate (50%-60% Peak VO_2_) and RPE |
| Hiraki, 2017 [18] | Combined training. Aerobic, walking, steps counted via pedometer worn majority of day, unsupervised. Resistance, handgrip device, squats, calf-raises. Control group also wore pedometer | 52 | Aerobic 7  Resistance ≥3 | Aerobic: 30 min each day or 8000-1000 steps. Resistance: not stated | Aerobic: brisk walking and RPE. Resistance: 20 repetitions per exercise at mid-level load and RPE |
| Howden, 2013 [19] | Combined training. Aerobic, treadmill, stationary bike, or rowing ergometer. Resistance, machine and free weight. 8 weeks supervised at gym, then home based using TheraBand and Swiss ball, unsupervised | 52 | 2-3 (150 min) | 50 min (includes 5 min WU & 5 min CD) | Moderate with progression and RPE |
| Howden, 2015 [20] | Combined training. Aerobic, treadmill, stationary bike, or rowing ergometer. Resistance, machine and free weight. 8 weeks supervised at gym, then home based using TheraBand and Swiss ball, unsupervised | 52 | 2-3 (150 min) | 50 min (includes 5 min WU & 5 min CD) | Moderate with progression and RPE |
| Huppertz, 2020 [21] | Combined training. Aerobic, treadmill, stationary bike, or rowing ergometer. Resistance, machine and free weight. 8 weeks supervised at gym, then home based using TheraBand and Swiss ball, unsupervised | 52 | 2-3 (150 min) | 50 min (includes 5 min WU & 5 min CD) | Moderate with progression and RPE |
| Ikizler, 2018 [22] | Aerobic, treadmill, elliptical cross-trainer, Nu-Step cross-trainer, recumbent stationary bicycle, supervised. | 16 | 3 | 40-45 min | Individualised, progressive intensity based on low impact, moderate pace (60-80% Peak VO_2_) and PRE |
| Kirkman, 2019 [23] | Aerobic, cycling, walking/jogging, elliptical, supervised | 12 | 3 | 45 min | Consistent modality of exercise with progressive duration and intensity up to moderate (60-85% HRR) and RPE |
| Kirkman, 2021 [24] | Aerobic, cycling, walking/jogging, elliptical, supervised | 12 | 3 | 45 min | Consistent modality of exercise with progressive duration and intensity up to moderate (60-85% HRR) and RPE |
| Leehey, 2009 [25] | Aerobic, interval, treadmill or outside. Supervised for 6 weeks, unsupervised thereafter | 24 | 3 | 30 min ↑ 5 min every 2 weeks to 40 min (includes 3-5 min WU, CD and stretches) | Individualised, progressive intensity (max of 60-84% Peak VO_2_) |
| Leehey, 2016 [26] | Combined training. Aerobic, interval, treadmill, elliptical trainer, cycle ergometer. Resistance, machine, elastic bands and free weight. 8 weeks supervised at gym, then home based, unsupervised | 52 | 3 | Supervised: 60 min aerobic, 30 min resistance  Unsupervised: 60 min 3/week or 30 min 6/week | Supervised: individualised, progressive intensity (max of 60-84% Peak VO_2_) Unsupervised: increase of step count by 10% weekly |
| Miele, 2017 [27] | Aerobic, various apparati, supervised | 16 | 3 | 15-30 min ↑ 55 min (includes 5 min WU & 5 min CD) | Moderate (50%-60% Peak VO_2_) and RPE |
| Mustata, 2011 [28] | Aerobic, treadmill, stationary cycle and elliptical trainer, supervised. Walking added, from second month, unsupervised | 52 | 2 supervised  1-3 unsupervised | 5-30 min ↑ 60 | Individualised, progressive. Moderate (40–60% of Peak VO_2_), heart rate monitor and RPE. |
| Nixon, 2021 [29] | Aerobic, walking, marching. Resistance, leg extension, calf raise, push up, sit to stand with hand weights  Unsupervised | 12 | 3 | 30-45 min | Individualised, progressive, resistance at 3 sets of 10 repetitions, RPE |
| Otobe, 2021 [30] | Combined training. Aerobic, cycle ergometer. Resistance, bodyweight, resistance band supervised once a week. Unsupervised aerobic and resistance at home twice weekly. | 24 | 1 supervised  2 unsupervised | 60 min (includes 10 min stretch, 20 min resistance, 5 min balance, 20 min aerobic & 5 min CD) | Moderate intensity with RPE, Resistance 1 set of 20 repetitions |
| Rossi, 2014 [31] | Combined training. Aerobic, treadmill, walking, stationary cycle. Resistance, free weights, supervised | 12 | 2 | 60 min supervised  (Also encouraged to perform 5,000-10,000 steps/day unsupervised) | Individualised, progressive, PLE. Aerobic, max of 60%–65% predicted maximal heart rate Resistance, 1 set of 10 repetition ↑ to 3 sets of 15 repetitions, ↑ weight |
| Shi, 2014 [32] | Tai Chi (10 movements), supervised 4 weeks, unsupervised thereafter | 12 | 3-5 | 45 min (includes 10 min WU & 5 min CD) | Moderate |
| Tang, 2017 [33] | Aerobic, walking, cycling, jogging, unsupervised | 12 | ≥3 | 30-40 min ↑ 55 min (includes 3-5 min WU & 3-5 min CD) | Individualised, progressive, low intensity ↑ moderate using RPE of 12-15 |
| Thompson, 2022 [34] | Combined training, Aerobic, treadmill, elliptical trainer or cycle ergometer Supervised once weekly for 8 weeks, unsupervised thereafter | 24 | 3 | 5 min WU, 10-20 min resistance, ↑ 30 min aerobic, 5 min CD | Moderate 40-49% HRR and RPE |
| Uchiyama, 2021 [35] | Combined training, Aerobic, walking. Resistance, TheraBand  Unsupervised | 24 | Aerobic 3, Resistance 2 | Aerobic, 20-30 per session. Resistance, not stated, | Aerobic, 40-60% HRR and RPE Resistance, progress to 70% 1RM, 1 set of 10 repetitions |
| Van Craenenbroeck, 2015 [36] | Intermittent aerobic, cycling, 3 sessions supervised during weeks 1-2, 1 session per week supervised thereafter | 12 | 28 (4 daily sessions) | 40 min (10 min x 4) | Moderate to 90% of heart rate achieved at the anaerobic threshold on baseline testing |
| Weiner, 2022 [37] | Combined training, Aerobic, treadmill, stationary bike. Resistance, free weights. Supervised 6 months at centre, then 1 session per week unsupervised at home, 2 supervised | 52 | Aerobic 3, Resistance 2 | Aerobic:20 min ↑ 40 min (includes 3-5 min WU & 3-5 min CD); Resistance: 10 min (includes brief WU & CD) | 50-60% HRR progress to 70-80% HRR and RPE |

**CD**=cool-down; **HRR**=heart rate reserve; **max**=maximum; **MEC**=maximal exercise capacity based on maximal O_2_ consumption and corresponding heart rate; **min**=minute; **Peak VO_2_**=oxygen uptake during peak exercise; **PLE**=perceived level of exertion; **RM**=repetition maximum; **RPE**=Borg Rating of Perceived Exertion scale; **VT**=ventilatory threshold; **WU**=warm-up; **↑**=increase; **↓**=decrease[40]

**References**

1. Aoike DT, Baria F, Kamimura MA, Ammirati A, de Mello MT, Cuppari L (2015). Impact of home-based aerobic exercise on the physical capacity of overweight patients with chronic kidney disease. Int Urol Nephrol 47:359-367. <https://doi.org/10.1007/s11255-014-0894-8>

2. Aoike DT, Baria F, Kamimura MA, Ammirati A, Cuppari L (2018). Home-based versus center-based aerobic exercise on cardiopulmonary performance, physical function, quality of life and quality of sleep of overweight patients with chronic kidney disease. Clin Exp Nephrol 22:87-98. <https://doi.org/10.1007/s10157-017-1429-2>

3. Barcellos FC, Del Vecchio FB, Reges A, Mielke G, Santos IS, Umpierre D, Bohlke M, Hallal PC (2018). Exercise in patients with hypertension and chronic kidney disease: a randomized controlled trial. J Hum Hypertens 32:397-407. <https://doi.org/10.1038/s41371-018-0055-0>

4. Baria F, Kamimura MA, Aoike DT, Ammirati A, Rocha ML, de Mello MT, Cuppari L (2014). Randomized controlled trial to evaluate the impact of aerobic exercise on visceral fat in overweight chronic kidney disease patients. Nephrol Dial Transplant 29:857-864. <https://doi.org/10.1093/ndt/gft529>

5. Beetham KS, Howden EJ, Isbel NM, Coombes JS (2018). Agreement between cystatin-C and creatinine based eGFR estimates after a 12-month exercise intervention in patients with chronic kidney disease. BMC Nephrol 19:366. <https://doi.org/10.1186/s12882-018-1146-4>

6. Castaneda C, Gordon PL, Parker RC, Uhlin KL, Roubenoff R, Levey AS (2004). Resistance training to reduce the malnutrition-inflammation complex syndrome of chronic kidney disease. Am J Kidney Dis 43:607-616. <https://doi.org/10.1053/j.ajkd.2003.12.025>

7. Chen PY, Huang YC, Kao YH, Chen JY (2010). Effects of an exercise program on blood biochemical values and exercise stage of chronic kidney disease patients. J Nurs Res 18:98-107. <https://doi.org/10.1097/JNR.0b013e3181dda726>

8. Correa HL, Neves RVP, Deus LA, Maia BCH, Maya AT, Tzanno-Martins C, Souza MK, Silva JAB, Haro AS, Costa F, Moraes MR, Simoes HG, Prestes J, Stone W, Rosa TS (2021). Low-load resistance training with blood flow restriction prevent renal function decline: The role of the redox balance, angiotensin 1-7 and vasopressin. Physiol Behav 230:113295. <https://doi.org/10.1016/j.physbeh.2020.113295>

9. de Araujo TB, de Luca Correa H, de Deus LA, Neves RVP, Reis AL, Honorato FS, da SBJM, Palmeira TRC, Aguiar SS, Sousa CV, Santos CAR, Neto LSS, Amorim CEN, Simoes HG, Prestes J, Rosa TS (2023). The effects of home-based progressive resistance training in chronic kidney disease patients. Exp Gerontol 171:112030. <https://doi.org/10.1016/j.exger.2022.112030>

10. Deus LA, Correa HL, Neves RVP, Reis AL, Honorato FS, Araujo TB, Souza MK, Haro AS, Silva VL, Barbosa J, Padula IA, Andrade RV, Simoes HG, Prestes J, Stone WJ, Melo GF, Rosa TS (2022). Metabolic and hormonal responses to chronic blood-flow restricted resistance training in chronic kidney disease: a randomized trial. Appl Physiol Nutr Metab 47:183-194. <https://doi.org/10.1139/apnm-2021-0409>

11. Eidemak I, Haaber AB, Feldt-Rasmussen B, Kanstrup IL, Strandgaard S (1997). Exercise training and the progression of chronic renal failure. Nephron 75:36-40. <https://doi.org/10.1159/000189497>

12. Gomes TS, Aoike DT, Baria F, Graciolli FG, Moyses RMA, Cuppari L (2017). Effect of Aerobic Exercise on Markers of Bone Metabolism of Overweight and Obese Patients With Chronic Kidney Disease. J Ren Nutr 27:364-371. <https://doi.org/10.1053/j.jrn.2017.04.009>

13. Greenwood SA, Koufaki P, Mercer TH, MacLaughlin HL, Rush R, Lindup H, O'Connor E, Jones C, Hendry BM, Macdougall IC, Cairns HS (2015). Effect of exercise training on estimated GFR, vascular health, and cardiorespiratory fitness in patients with CKD: a pilot randomized controlled trial. Am J Kidney Dis 65:425-434. <https://doi.org/10.1053/j.ajkd.2014.07.015>

14. Gregory SM, Headley SA, Germain M, Flyvbjerg A, Frystyk J, Coughlin MA, Milch CM, Sullivan S, Nindl BC (2011). Lack of circulating bioactive and immunoreactive IGF-I changes despite improved fitness in chronic kidney disease patients following 48 weeks of physical training. Growth Horm IGF Res 21:51-56. <https://doi.org/10.1016/j.ghir.2010.12.005>

15. Headley S, Germain M, Milch C, Pescatello L, Coughlin MA, Nindl BC, Cornelius A, Sullivan S, Gregory S, Wood R (2012). Exercise training improves HR responses and V O2peak in predialysis kidney patients. Med Sci Sports Exerc 44:2392-2399. <https://doi.org/10.1249/MSS.0b013e318268c70c>

16. Headley S, Germain M, Wood R, Joubert J, Milch C, Evans E, Poindexter A, Cornelius A, Brewer B, Pescatello LS, Parker B (2014). Short-term aerobic exercise and vascular function in CKD stage 3: a randomized controlled trial. Am J Kidney Dis 64:222-229. <https://doi.org/10.1053/j.ajkd.2014.02.022>

17. Headley S, Germain M, Wood R, Joubert J, Milch C, Evans E, Cornelius A, Brewer B, Taylor B, Pescatello LS (2017). Blood pressure response to acute and chronic exercise in chronic kidney disease. Nephrology (Carlton) 22:72-78. <https://doi.org/10.1111/nep.12730>

18. Hiraki K, Shibagaki Y, Izawa KP, Hotta C, Wakamiya A, Sakurada T, Yasuda T, Kimura K (2017). Effects of home-based exercise on pre-dialysis chronic kidney disease patients: a randomized pilot and feasibility trial. BMC Nephrol 18:198. <https://doi.org/10.1186/s12882-017-0613-7>

19. Howden EJ, Leano R, Petchey W, Coombes JS, Isbel NM, Marwick TH (2013). Effects of exercise and lifestyle intervention on cardiovascular function in CKD. Clin J Am Soc Nephrol 8:1494-1501. <https://doi.org/10.2215/CJN.10141012>

20. Howden EJ, Coombes JS, Strand H, Douglas B, Campbell KL, Isbel NM (2015). Exercise training in CKD: efficacy, adherence, and safety. Am J Kidney Dis 65:583-591. <https://doi.org/10.1053/j.ajkd.2014.09.017>

21. Huppertz N, Beetham KS, Howden EJ, Leicht AS, Isbel NM, Coombes JS (2020). A 12-month lifestyle intervention does not improve cardiac autonomic function in patients with chronic kidney disease. Auton Neurosci 224:102642. <https://doi.org/10.1016/j.autneu.2020.102642>

22. Ikizler TA, Robinson-Cohen C, Ellis C, Headley SAE, Tuttle K, Wood RJ, Evans EE, Milch CM, Moody KA, Germain M, Limkunakul C, Bian A, Stewart TG, Himmelfarb J (2018). Metabolic Effects of Diet and Exercise in Patients with Moderate to Severe CKD: A Randomized Clinical Trial. J Am Soc Nephrol 29:250-259. <https://doi.org/10.1681/ASN.2017010020>

23. Kirkman DL, Ramick MG, Muth BJ, Stock JM, Pohlig RT, Townsend RR, Edwards DG (2019). Effects of aerobic exercise on vascular function in nondialysis chronic kidney disease: a randomized controlled trial. Am J Physiol Renal Physiol 316:F898-F905. <https://doi.org/10.1152/ajprenal.00539.2018>

24. Kirkman DL, Ramick MG, Muth BJ, Stock JM, Townsend RR, Edwards DG (2021). A randomized trial of aerobic exercise in chronic kidney disease: Evidence for blunted cardiopulmonary adaptations. Ann Phys Rehabil Med 64:101469. <https://doi.org/10.1016/j.rehab.2020.101469>

25. Leehey DJ, Moinuddin I, Bast JP, Qureshi S, Jelinek CS, Cooper C, Edwards LC, Smith BM, Collins EG (2009). Aerobic exercise in obese diabetic patients with chronic kidney disease: a randomized and controlled pilot study. Cardiovasc Diabetol 8:62. <https://doi.org/10.1186/1475-2840-8-62>

26. Leehey DJ, Collins E, Kramer HJ, Cooper C, Butler J, McBurney C, Jelinek C, Reda D, Edwards L, Garabedian A, O''Connell S (2016). Structured Exercise in Obese Diabetic Patients with Chronic Kidney Disease: A Randomized Controlled Trial. Am J Nephrol 44:54-62. <https://doi.org/10.1159/000447703>

27. Miele EM, Headley SAE, Germain M, Joubert J, Herrick S, Milch C, Evans E, Cornelius A, Brewer B, Taylor B, Wood RJ (2017). High-density lipoprotein particle pattern and overall lipid responses to a short-term moderate-intensity aerobic exercise training intervention in patients with chronic kidney disease. Clin Kidney J 10:524-531. <https://doi.org/10.1093/ckj/sfx006>

28. Mustata S, Groeneveld S, Davidson W, Ford G, Kiland K, Manns B (2011). Effects of exercise training on physical impairment, arterial stiffness and health-related quality of life in patients with chronic kidney disease: a pilot study. Int Urol Nephrol 43:1133-1141. <https://doi.org/10.1007/s11255-010-9823-7>

29. Nixon AC, Bampouras TM, Gooch HJ, Young HML, Finlayson KW, Pendleton N, Mitra S, Brady ME, Dhaygude AP (2021). Home-based exercise for people living with frailty and chronic kidney disease: A mixed-methods pilot randomised controlled trial. PLoS One 16:e0251652. <https://doi.org/10.1371/journal.pone.0251652>

30. Otobe Y, Yamada M, Hiraki K, Onari S, Taki Y, Sumi H, Hachisuka R, Han W, Takahashi M, Suzuki M, Kimura Y, Koyama S, Masuda H, Shibagaki Y, Tominaga N (2021). Physical Exercise Improves Cognitive Function in Older Adults with Stage 3-4 Chronic Kidney Disease: A Randomized Controlled Trial. Am J Nephrol 52:929-939. <https://doi.org/10.1159/000520230>

31. Rossi AP, Burris DD, Lucas FL, Crocker GA, Wasserman JC (2014). Effects of a renal rehabilitation exercise program in patients with CKD: a randomized, controlled trial. Clin J Am Soc Nephrol 9:2052-2058. <https://doi.org/10.2215/CJN.11791113>

32. Shi ZM, Wen HP, Liu FR, Yao CX (2014). The effects of tai chi on the renal and cardiac functions of patients with chronic kidney and cardiovascular diseases. J Phys Ther Sci 26:1733-1736. <https://doi.org/10.1589/jpts.26.1733>

33. Tang Q, Yang B, Fan F, Li P, Yang L, Guo Y (2017). Effects of individualized exercise program on physical function, psychological dimensions, and health-related quality of life in patients with chronic kidney disease: A randomized controlled trial in China. Int J Nurs Pract 23:1-8. <https://doi.org/10.1111/ijn.12519>

34. Thompson S, Wiebe N, Stickland MK, Gyenes GT, Davies R, Vallance J, Graham M (2022). Physical Activity in Renal Disease and the Effect on Hypertension: A Randomized Controlled Trial. Kidney Blood Press Res 47:475-485. <https://doi.org/10.1159/000524518>

35. Uchiyama K, Adachi K, Muraoka K, Nakayama T, Oshida T, Yasuda M, Hishikawa A, Minakuchi H, Miyashita K, Tokuyama H, Wakino S, Itoh H (2021). Home-based aerobic exercise and resistance training for severe chronic kidney disease: a randomized controlled trial. J Cachexia Sarcopenia Muscle 12:1789-1802. <https://doi.org/10.1002/jcsm.12775>

36. Van Craenenbroeck AH, Van Craenenbroeck EM, Van Ackeren K, Vrints CJ, Conraads VM, Verpooten GA, Kouidi E, Couttenye MM (2015). Effect of Moderate Aerobic Exercise Training on Endothelial Function and Arterial Stiffness in CKD Stages 3-4: A Randomized Controlled Trial. Am J Kidney Dis 66:285-296. <https://doi.org/10.1053/j.ajkd.2015.03.015>

37. Weiner DE, Liu CK, Miao S, Fielding R, Katzel LI, Giffuni J, Well A, Seliger SL (2023). Effect of Long-term Exercise Training on Physical Performance and Cardiorespiratory Function in Adults With CKD: A Randomized Controlled Trial. Am J Kidney Dis 81:59-66. <https://doi.org/10.1053/j.ajkd.2022.06.008>
