# Supplementary material for: The effect of exercise training in people with pre-dialysis chronic kidney disease: a systematic review with meta-analysis
Source: J Nephrol. 2024 Oct 17;37(8):2063–98. doi: 10.1007/s40620-024-02081-9 (PMC11649798; doi:10.1007/s40620-024-02081-9)
Supplement: Supplementary file 7 — Supplementary file7 (DOCX 104 KB) [file 40620_2024_2081_MOESM7_ESM.docx]

**The effect of exercise training in people with pre-dialysis chronic kidney disease. A systematic review with meta-analysis.**

Annette Traise*, Gudrun Dieberg, Melissa J Pearson, Neil A Smart

Clinical Exercise Physiology, School of Science and Technology, University of New England, NSW 2351, Australia

* Corresponding author

**Online Resource** **6**

**Supplemental material: Table 6** Summary of sub-analyses

**Supplemental Table S6** Summary of sub-analyses (modality, duration, supervision, frequency, and CKD stage) for: Aerobic Capacity, Functional Ability, Quality of Life, Renal Parameters, Cardiovascular Risk Factors, and Inflammatory Markers

| **Outcome** | **Number of studies (intervention groups)** | **Participants**  **Exercise/Control** | **Result:**  **MD/SMD (95%CI), *p*, I^2^** |
| --- | --- | --- | --- |
| **Aerobic capacity** | | | |
| **Peak VO_2_ [mL/kg/min]** | | | |
| *Modality* |  |  |  |
| - Aerobic Training | 8 (9) | 135/116 | MD 3.13 (1.30, 4.95), p=0.0008, I^2^=70% ***** |
| - Combined Training | 6 | 141/155 | MD 1.96 (0.78, 3.14), p=0.001, I^2^=16% ***** |
| *Intervention Duration* |  |  |  |
| - ≤ 12 weeks | 2 | 34/37 | MD 4.82 (1.31, 8.34), p=0.007, I^2^=83% ***** |
| - > 12 weeks to ≤ 6 months | 5 (6) | 89/69 | MD 1.09 (-0.30, 2.47), p=0.13, I^2^=3% |
| - > 6 months | 7 | 153/165 | MD 2.37 (1.16, 3.59), p=0.0001, I^2^=28% ***** |
| *Supervision* |  |  |  |
| - Supervised | 5 | 85/74 | MD 2.68 (1.24, 4.11), p=0.0003, I^2^=0% ***** |
| - Unsupervised | 1 | 12/11 | MD 4.00 (1.50, 6.50), p=0.002, I^2^=NA ***** |
| - Initially supervised, then unsupervised | 5 | 117/108 | MD 1.18 (-0.40, 2.77), p=0.14, I^2^=51% |
| - Both supervised and unsupervised | 4 | 62/78 | MD 4.18 (1.23, 7.13), p=0.005, I^2^=68% ***** |
| *Frequency* |  |  |  |
| - 3 session/week | 10 (11) | 161/61 | MD 1.59 (0.55, 2.63), p=0.003, I^2^=16% ***** |
| - 2-3 session/week | 1 | 74/68 | MD 2.60 (1.29, 3.91), p<0.0001, I^2^=N/A ***** |
| - 2-5 session/week | 1 | 10/10 | MD 3.70 (-0.85, 8.25), p=0.11, I^2^=N/A |
| - 1-4 session/week | 1 | 19/21 | MD 6.61 (4.58, 8.64), p<0.00001, I^2^=N/A ***** |
| - Daily sessions | 1 | 12/11 | MD 4.00 (1.50, 6.50), p=0.002, I^2^=N/A ***** |
| *CKD stage* |  |  |  |
| - 2 to 4 only | 3 | 31/33 | MD 0.11 (-1,28, 1.50), p=0.88, I^2^=0% |
| - 3 to 4 only | 7 (8) | 185/180 | MD 3.56 (1.98, 5.13), p<0.00001, I^2^=57% ***** |
| - 3, 4, and 3 to 5 | 4 | 276/271 | MD 2.83 (1.25, 4.41), p=0.0004, I^2^=0% ***** |
| **Peak respiratory Exchange Ratio** | | | |
| *Modality* |  |  |  |
| - Aerobic Training | 3 | 52/60 | MD 0.01 (-0.04, 0.06), p=0.61, I^2^=0% |
| - Combined Training | 1 | 19/17 | MD 0.02 (-0.05, 0.09), p=0.55, I^2^=N/A |
| *Intervention Duration* |  |  |  |
| - ≤ 12 weeks | 2 | 31/35 | MD -0.01 (-0.07, 0.06), p=0.86, I^2^=0% |
| - > 12 weeks to ≤ 6 months | 1 | 21/25 | MD 0.04 (-0.04, 0.12), p=0.32, I^2^=N/A |
| - > 6 months | 1 | 19/17 | MD 0.02 (-0.05, 0.09), p=0.55, I^2^=N/A |
| *Supervision* |  |  |  |
| - Supervised | 2 | 33/39 | MD 0.02 (-0.03, 0.07), p=0.51, I^2^=0% |
| - Unsupervised | 1 | 19/17 | MD 0.02 (-0.05, 0.09), p=0.55, I^2^=N/A |
| - Both supervised and unsupervised | 1 | 19/21 | MD -0.05 (-0.24, 0.14), p=0.61, I^2^=N/A |
| *CKD stage* |  |  |  |
| - 3 to 4 only | 2 | 38/38 | MD 0.01 (-0.05, 0.07), p=0.69, I^2^=0% |
| - 3, 4, and 3 to 5 | 2 | 33/39 | MD 0.02 (-0.03, 0.07), p=0.51, I^2^=0% |
| **Functional Ability** | | | |
| **Six-Minute Walk Test (6MWT) [metres]** | | | |
| *Modality* |  |  |  |
| - Aerobic Training | 2 (3) | 67/57 | MD 81.76 (28.45, 135.06), p=0.003, I^2^=62% ***** |
| - Resistance Training | 1 | 16/15 | MD 121.54 (55.04, 188.04), p=0.0003, I^2^=N/A ***** |
| - Combined Training | 4 | 165/169 | MD 40.35 (14.92, 65.77), p=0.002, I^2^=42% ***** |
| *Intervention Duration* |  |  |  |
| - ≤ 12 weeks | 2 | 90/88 | MD 54.36 (35.35, 73.36), p<0.00001, I^2^=11% ***** |
| - > 12 weeks to ≤ 6 months | 2 (2) | 41/30 | MD 117.72 (75.78, 159.66), p<0.0001, I^2^=0% ***** |
| - > 6 months | 3 | 117/123 | MD 26.79 (7.04, 46.54), p=0.008, I^2^=0% ***** |
| *Supervision* |  |  |  |
| - Supervised | 3 | 77/69 | MD 89.19 (51.50, 126.89), p<0.00001, I^2^=34% ***** |
| - Unsupervised | 1 | 42/42 | MD 46.98 (24.90, 69.06), p<0.0001, I^2^=N/A ***** |
| - Initially supervised, then unsupervised | 3 | 100/93 | MD 45.57 (-8.87, 100.00), p=0.10, I^2^=61% |
| - Both supervised and unsupervised | 1 | 29/37 | MD 37.19 (-20.14, 94.52), p=0.20, I^2^=NA |
| *CKD stage* |  |  |  |
| - 2, and 1 to 3 | 2 | 58/57 | MD 77.39 (5.57, 149.21), p=0.03, I^2^=77% ***** |
| - 2 to 4 only | 1 | 14/18 | MD 9.00 {-74.69, 92.69), p=0.83, I^2^=N/A |
| - 3 to 4 only | 4 (5) | 176/166 | MD 61.33 (28.10, 94.57), p=0.0003, I^2^=65% ***** |
| **Timed Up and Go (TUG)** | | | |
| *Modality* |  |  |  |
| - Aerobic Training | 1 (2) | 25/15 | SMD -0.32 (-0.96, 0.33), p=0.34, I^2^=0% |
| - Resistance Training | 1 | 16/15 | SMD -1.01 (-1.76, -0.25), p=0.009, I^2^=N/A ***** |
| - Combined Training | 4 | 172/165 | SMD -0.30 (-0.51, -0.08), p=0.007, I^2^=0% ***** |
| *Intervention Duration* |  |  |  |
| - > 12 weeks to ≤ 6 months | 3 (4) | 96/73 | SMD -0.48 (-0.79, -0.16), p=0.003, I^2^=0% ***** |
| - > 6 months | 3 | 117/122 | SMD -0.26 (-0.52, - 0.01), p=0.05, I^2^=0% ***** |
| *Supervision* |  |  |  |
| - Supervised | 3 | 84/66 | SMD -0.51 (-0.87, -0.15), p=0.006, I^2^=9% ***** |
| - Initially supervised, then unsupervised | 3 | 100/93 | SMD -0.32 (-0.61, - 0.04), p=0.03, I^2^=0% ***** |
| - Both supervised and unsupervised | 1 | 29/36 | SMD -0.09 (-0.57, 0.40), p=0.73, I^2^=NA |
| *CKD stage* |  |  |  |
| - 2, and 1 to 3 | 1 | 16/15 | SMD -1.01 (-1.76, - 0.25), p=0.009, I^2^=N/A ***** |
| - 2 to 4 only | 2 | 69/61 | SMD -0.33 (-0.68, 0.02), p=0.06, I^2^=0% ***** |
| - 3 to 4 only | 3 (4) | 128/119 | SMD -0.28 (-0.53, - 0.03), p=0.03, I^2^=0% ***** |
| **Two-Minute Step Test (2MST) [steps]** | | | |
| *Modality* |  |  |  |
| - Aerobic Training | 1 (2) | 25/15 | MD 72.93 (51.16, 94.70), p<0.00001, I^2^=0% ***** |
| - Combined Training | 1 | 55/44 | MD 33.90 (18.10, 49.70), p<0.0001, I^2^=N/A ***** |
| *Intervention Duration* |  |  |  |
| - > 12 weeks to ≤ 6 months | 2 (3) | 80/59 | MD 57.48 (27.80, 87.16), p=0.0001, I^2^=76 ***** |
| *Supervision* |  |  |  |
| - Supervised | 2 | 68/52 | MD 49.45 (14.50, 84.40), p=0.006, I^2^=76% ***** |
| - Initially supervised, then unsupervised | 1 | 12/7 | MD 76.10 (44.96, 107.24), p<0.00001, I^2^=N/A ***** |
| *CKD stage* |  |  |  |
| - 2 to 4 only | 1 | 55/44 | MD 33.90 (18.10, 49.70), p<0.0001, I^2^=N/A ***** |
| - 3 to 4 only | 1 (2) | 25/15 | MD 72.93 (51.16, 94.70), p<0.00001, I^2^=0% ***** |
| **Sit To stand (STS) [repetitions in 30 seconds]** | | | |
| *Modality* |  |  |  |
| - Aerobic Training | 2 (3) | 37/29 | MD 6.07 (-0.20, 12.34), p=0.06, I^2^=91% ***** |
| - Combined Training | 1 | 55/44 | MD 2.30 (0.92, 3.68), p=0.001, I^2^=N/A ***** |
| *Intervention Duration* |  |  |  |
| - ≤ 12 weeks | 1 | 12/14 | MD 1.00 (-0.45, 2.45), p=0.18, I^2^=N/A |
| - > 12 weeks to ≤ 6 months | 2 (3) | 80/59 | MD 6.45 (1.18, 11.72), p=0.02, I^2^=87% ***** |
| *Supervision* |  |  |  |
| - Supervised | 3 | 80/66 | MD 3.31 (0.47, 6.15), p=0.02, I^2^=85% ***** |
| - Initially supervised, then unsupervised | 1 | 12/7 | MD 9.10 (4.54, 13.66), p<0.0001, I^2^=N/A ***** |
| *CKD stage* |  |  |  |
| - 2 to 4 only | 1 | 55/44 | MD 2.30 (0.92, 3.68), p=0.001, I^2^=N/A ***** |
| - 3 to 4 only | 1 (2) | 25/15 | MD 8.99 (5.96, 12.02), p<0.0001, I^2^=N/A ***** |
| - 3, 4, and 3 to 5 | 1 | 12/14 | MD 1.00 (-0.45, 2.45), p=0.18, I^2^=N/A |
| **Handgrip Strength [kg]** | | | |
| *Modality* |  |  |  |
| - Aerobic Training | 2 | 36/29 | MD 0.17 (-5.77, 6.12), p=0.95, I^2^=0% |
| - Resistance Training | 2 | 46/45 | MD 5.77 (3.50, 8.03), p<0.00001, I^2^=0% ***** |
| - Combined Training | 3 | 111/105 | MD -0.30 (-2.36, 1.76), p=0.78, I^2^=5% |
| *Intervention Duration* |  |  |  |
| - ≤ 12 weeks | 1 | 12/14 | MD -0.99 (-9.67, 7.69), p=0.82, I^2^=NA |
| - > 12 weeks to ≤ 6 months | 4 | 93/83 | MD 3.43 (-0.51, 7.37), p=0.09, I^2^=68% * |
| - > 6 months | 2 | 88/82 | MD 0.63 (-3.18, 4.44), p=0.74, I^2^=43% |
| *Supervision* |  |  |  |
| - Supervised | 3 | 58/59 | MD 5.21 (2.14, 8.28), p=0.0009, I^2^=33% * |
| - Unsupervised | 3 | 61/52 | MD 0.47 (-2.82, 3.76), p=0.78, I^2^=0% |
| - Initially supervised, then unsupervised | 1 | 74/68 | MD -0.60 (-2.89, 1.69), p=0.61, I^2^=NA |
| *CKD stage* |  |  |  |
| - 2 to 4 only | 2 | 46/45 | MD 5.77 (3.50, 8.03), p<0.00001, I^2^=0% * |
| - 3 to 4 only | 3 | 112/97 | MD 0.03 (-2.03, 2.10), p=0.98, I^2^=0% |
| - 3, 4, and 3 to 5 | 2 | 35/37 | MD -1.47 (-5.47, 2.53), p=0.47, I^2^=0% |
| **Quality of Life (QoL)** | | | |
| **Short Form 36 General Health (GH)** | | | |
| *Modality* |  |  |  |
| - Aerobic Training | 3 | 51/48 | MD 5.58 (-3.64, 14.79), p=0.24, I^2^=42% |
| - Combined Training | 2 | 71/69 | MD 4.31 (-0.81, 9.43), p=0.10 I^2^=15% |
| *Intervention Duration* |  |  |  |
| - ≤ 12 weeks | 2 | 64/63 | MD 3.58 (-2.70, 9.86), p=0.26, I^2^=41% |
| - > 12 weeks to ≤ 6 months | 2 | 48/44 | MD 3.09 (-3.77, 9.95), p=0.38, I^2^=0% |
| - > 6 months | 1 | 10/10 | MD 18 (-1.67, 37.67), p=0.07, I^2^=N/A |
| *Supervision* |  |  |  |
| - Supervised | 2 | 73/67 | MD 6.40 (1.48, 11.33), p=0.01, I^2^=0% ***** |
| - Unsupervised | 1 | 23/23 | MD 0.50 (-8.05, 9.05), p=0.91, I^2^=NA |
| - Both supervised and unsupervised | 2 | 26/27 | MD 6.52 (-11.06, 24.10), p=0.47, I^2^=65% |
| *CKD stage* |  |  |  |
| - 3 to 4 only | 2 | 64/63 | MD 3.58 (-2.70, 9.86), p=0.26, I^2^=41% |
| - 3, 4, and 3 to 5 | 3 | 58/54 | MD 5.77 (-2.68, 14.22), p=0.18, I^2^=32% |
| **Short Form 36 Mental Component Summary (MCS)** | | | |
| *Modality* |  |  |  |
| - Aerobic Training | 2 | 52/52 | MD 2.69 (0.60, 4.79), p=0.01, I^2^=0% ***** |
| - Combined Training | 4 | 63/75 | MD 0.40 (-2.34, 3.14), p=0.78, I^2^=0% |
| *Intervention Duration* |  |  |  |
| - ≤ 12 weeks | 2 | 51/54 | MD 2.22 (0.05, 4.39), p=0.04, I^2^=2% ***** |
| - > 12 weeks to ≤ 6 months | 2 | 40/45 | MD 1.09 (-2.17, 4.35), p=0.51, I^2^=0% |
| - > 6 months | 2 | 24/28 | MD 1.28 (-3.90, 6.46), p=0.63, I^2^=0% |
| *Supervision* |  |  |  |
| - Unsupervised | 3 | 74/77 | MD 1.89 (0.00, 3.79), p=0.05, I^2^=0% ***** |
| - Initially supervised, then unsupervised | 2 | 31/40 | MD 1.00 (-3.06, 5.07), p=0.63, I^2^=0% |
| - Both supervised and unsupervised | 1 | 10/10 | MD 3.64 (-3.35, 10.63), p=0.31, I^2^=N/A |
| *CKD stage* |  |  |  |
| - 2, and 1 to 3 | 1 | 42/42 | MD 2.60 (0.40, 4.80), p=0.02, I^2^=N/A * |
| - 2 to 4 only | 1 | 14/18 | MD -1.60 (-9.32, 6.12), p=0.68, I^2^=N/A |
| - 3, 4, and 3 to 5 | 4 | 59/67 | MD 1.13 (-1.57, 3.83), p=0.41, I^2^=0% |
| **Short Form 36 Physical Component Summary (PCS)** | | | |
| *Modality* |  |  |  |
| - Aerobic Training | 2 | 52/52 | MD 1.85 (0.13, 3.58), p=0.03, I^2^=0% ***** |
| - Combined Training | 4 | 63/75 | MD 0.05 (-3.42, 3.51), p=0.98, I^2^=38% |
| *Intervention Duration* |  |  |  |
| - ≤ 12 weeks | 2 | 51/54 | MD -1.26 (-8.34, 5.82), p=0.73, I^2^=81% |
| - > 12 weeks to ≤ 6 months | 2 | 40/45 | MD 1.40 (-2.03, 4.83), p=0.42, I^2^=0% |
| - > 6 months | 2 | 24/28 | MD 2.66 (-2.38, 7.70), p=0.30, I^2^=0% |
| *Supervision* |  |  |  |
| - Unsupervised | 3 | 74/77 | MD -0.53 (-4.82, 3.77), p=0.81, I^2^=63% |
| - Initially supervised, then unsupervised | 2 | 31/40 | MD 2.19 (-1.23, 5.61), p=0.21, I^2^=0% |
| - Both supervised and unsupervised | 1 | 10/10 | MD 2.80 (-6.37, 11.97), p=0.55, I^2^=N/A |
| *CKD stage* |  |  |  |
| - 2, and 1 to 3 | 1 | 42/42 | MD 1.25 (0.07, 3.57), p=0.04, I^2^=N/A ***** |
| - 2 to 4 only | 1 | 14/18 | MD 2.60 (-3.43, 8.63), p=0.40, I^2^=N/A |
| - 3, 4, and 3 to 5 | 4 | 59/67 | MD -0.22 (-3.86, 3.43), p=0.91, I^2^=33% |
| **Renal Parameters** | | | |
| **Estimated Glomerular Filtration Rate (eGFR sCr) [mL/min/1.73m^2^]** | | | |
| *Modality* |  |  |  |
| - Aerobic Training | 4(5) | 84/73 | MD 1.39 (-1.18, 3.97), p=0.29, I^2^=0% |
| - Resistance Training | 2 | 51/50 | MD 4.64 (3.25, 6.04), p<0.00001, I^2^=0% ***** |
| - Combined Training | 9 | 244/247 | MD 0.28 (-1.49, 2.06), p=0.75, I^2^=0% |
| - Mind-Body Exercise | 1 | 11/10 | MD 8.00 (-13.23, 29.23), p=0.46, I^2^=N/A |
| *Intervention Duration* |  |  |  |
| - ≤ 12 weeks | 3 | 45/47 | MD 1.14 (-1.68, 3.96), p=0.43, I^2^=0% |
| - > 12 weeks to ≤ 6 months | 7(8) | 195/176 | MD 3.96 (2.69, 5.23), p<0.00001, I^2^=0% ***** |
| - > 6 months | 6 | 150/157 | MD 0.45 (-1.60, 2.49), p=0.67, I^2^=0% |
| *Supervision* |  |  |  |
| - Supervised | 7 | 172/1550 | MD 4.19 (2.88, 5.49), p<0.00001, I^2^=0% ***** |
| - Unsupervised | 1 | 14/14 | MD -0.30 (-9.10, 8.50), p=0.95, I^2^=N/A |
| - Initially supervised, then unsupervised | 5 | 124/122 | MD -0.01 (-2.20, 2.19), p=0.99, I^2^=0% |
| - Both supervised and unsupervised | 4 | 80/89 | MD 1.38 (-1.02, 3.78), p=0.26, I^2^=0% |
| *CKD stage* |  |  |  |
| - 1 to 5 | 1 | 11/10 | MD 8.00 (-13.23, 29.23), p=0.46, I^2^=N/A |
| - 2, and 1 to 3 | 2 | 51/50 | MD 4.64 (3.25, 6.04), p<0.00001, I^2^=0% ***** |
| - 2 to 4 only | 3 | 82/78 | MD 0.81 (-2.23, 3.85), p=0.60, I^2^=0% |
| - 3 to 4 only | 7(8) | 193/185 | MD 0.63 (-1.13, 2.38), p=0.48, I^2^=0% |
| - 3, 4, and 3 to 5 | 3 | 53/57 | MD 0.27 (-5.06, 5.60), p=0.92, I^2^=0% |
| **Estimated Glomerular Filtration Rate serum cystatin-C (eGFR sCys-C) [mL/min/1.73m^2^]** | | | |
| *Modality* |  |  |  |
| - Aerobic Training | 1 | 24/18 | MD 0.40 (-9.00, 9.80), p=0.93, I^2^=N/A |
| - Combined Training | 3 | 105/99 | MD 1.55 (-0.56, 3.66), p=0.15, I^2^=0% |
| *Intervention Duration* |  |  |  |
| - ≤ 12 weeks | 1 | 74/68 | MD 1.70 (-0.47, 3.87), p=0.12, I^2^=N/A |
| - > 12 weeks to ≤ 6 months | 2 | 47/39 | MD -0.89 (-7.79, 6.02), p=0.80, I^2^=0% |
| - > 6 months | 1 | 8/10 | MD 3.80 (-15.14, 22.74), p=0.69, I^2^=N/A |
| *Supervision* |  |  |  |
| - Supervised | 2 | 98/86 | MD 1.63 (-0.48, 3.75), p=0.13, I^2^=0% |
| - Both supervised and unsupervised | 2 | 31/31 | MD -1.01 (-9.98, 7.96), p=0.83, I^2^=0% |
| *CKD stage* |  |  |  |
| - 3 to 4 only | 4 | 129/117 | MD 1.49 (-0.56, 3.55), p=0.15, I^2^=0% |
| **Serum Creatinine (sCr) [mg/dL]** | | | |
| *Modality* |  |  |  |
| - Aerobic Training | 2 | 31/19 | MD -0.10 (-0.76, 0.57), p=0.78, I^2^=0% |
| - Resistance Training | 1 | 16/15 | MD -0.20 (-0.39, -0.01), p=0.04, I^2^=N/A * |
| - Combined Training | 5 | 128/129 | MD 0.01 (-0.08, 0.11), p=0.79, I^2^=0% |
| - Mind-Body Exercise | 1 | 11/10 | MD -0.40 (-1.14, 0.34), p=0.29, I^2^=N/A |
| *Intervention Duration* |  |  |  |
| - ≤ 12 weeks | 1 | 11/10 | MD -0.40 (-1.14, 0.34), p=0.29, I^2^= N/A |
| - > 12 weeks to ≤ 6 months | 5 | 83/75 | MD -0.15 (-0.31, 0.01), p=0.07, I^2^=0% |
| - > 6 months | 3 | 92/88 | MD 0.01 (-0.09, 0.12), p=0.78, I^2^=0% |
| *Supervision* |  |  |  |
| - Supervised | 2 | 26/25 | MD- 0.19 (-0.37, -0.00), p=0.05, I^2^=o% * |
| - Unsupervised | 1 | 24/15 | MD -0.22 (-1.07, 0.63), p=0.61, I^2^=N/A |
| - Initially supervised, then unsupervised | 4 | 105/102 | MD 0.01 (-0.09, 0.12), p=0.79, I^2^=0% |
| - Both supervised and unsupervised | 2 | 31/31 | MD -0.08 (-0.43, 0.26), p=0.63, I^2^=0% |
| *CKD stage* |  |  |  |
| - 1 to 5 | 1 | 11/10 | MD -0.40 (-1.14, 0.34), p=0.29, I^2^= N/A |
| - 2, and 1 to 3 | 1 | 16/15 | MD -0.20 (-0.39, - 0.01), p=0.04, I^2^= N/A * |
| - 2 to 4 only | 2 | 17/14 | MD 0.10 (-0.59, 0.79), p=0.78, I^2^=0% |
| - 3 to 4 only | 4 | 129/114 | MD 0.01 (-0.09, 0.11), p=0.88, I^2^=0% |
| - 3, 4, and 3 to 5 | 1 | 13/20 | MD 0.16 (-0.71, 1.02), p=0.72, I^2^= N/A |
| **Serum Albumin (ALB) [g/dL]** | | | |
| *Modality* |  |  |  |
| - Aerobic Training | 2 | 38/33 | MD -0.13 (-0.40, 0.15), p=0.37, I^2^=66% |
| - Resistance Training | 3 | 65/62 | MD 0.57 (0.18, 0.97), p=0.005, I^2^=72% ***** |
| - Combined | 3 | 82/80 | MD 0.00 (-0.01, 0.02), p=0.77, I^2^=0% |
| *Intervention Duration* |  |  |  |
| - ≤ 12 weeks | 2 | 28/27 | MD 0.02 (-0.54, 0.58), p=0.94, I^2^= 94% |
| - > 12 weeks to ≤ 6 months | 4 | 121112 | MD 0.04 (-0.03, 0.11), p=0.29, I^2^=82% |
| - > 6 months | 1 | 36/36 | MD -0.03 (-0.18, 0.12), p=0.69, I^2^=N/A |
| *Supervision* |  |  |  |
| - Supervised | 4 | 89/80 | MD 0.40 (0.08, 0.72), p=0.01, I^2^=80% * |
| - Unsupervised | 1 | 23/23 | MD 0.00 (-0.02, 0.03), p=0.81, I^2^=N/A |
| - Initially supervised, then unsupervised | 2 | 50/51 | MD -0.13 (-0.36, 0.10), p=0.27, I^2^=66% |
| - Both supervised and unsupervised | 1 | 23/21 | MD 0.00 (-0.02, 0.03), p=0.81, I^2^=N/A |
| *CKD stage* |  |  |  |
| - 2, and 1 to 3 | 2 | 51/50 | MD 0.79 (0.47, 1.12), p<0.00001, I^2^=0% ***** |
| - 3 to 4 only | 5 | 111/102 | MD 0.02 (-0.12, 0.15), p=0.80, I^2^=80% |
| - 3, 4, and 3 to 5 | 1 | 23/23 | MD 0.00 (-0.02, 0.03), p=0.81, I^2^=N/A |
| **Serum Cystatin-C (sCys-C) [mg/L]** | | | |
| *Modality* |  |  |  |
| - Resistance Training | 1 | 16/15 | MD -0.06 (-0.11, - 0.01), p=0.01, I^2^=N/A* |
| - Combined Training | 3 | 105/99 | MD -0.10 (-0.24, 0.04), p=0.15, I^2^=0% |
| *Intervention Duration* |  |  |  |
| - ≤ 12 weeks | 1 | 74/68 | MD -0.10 (-0.25, 0.05), p=0.20, I^2^=N/A |
| - > 12 weeks to ≤ 6 months | 2 | 24/25 | MD -0.06 (-0.11, -0.02), p=0.009, I^2^=0%* |
| - > 6 months | 1 | 23/21 | MD -0.07 (-0.45, 0.31), p=0.71, I^2^=N/A |
| *Supervision* |  |  |  |
| - Supervised | 2 | 90/83 | MD -0.06 (-0.11, -0.02), p=0.005, I^2^=0%* |
| - Both supervised and unsupervised | 2 | 31/31 | MD -0.11 (-0.42, 0.21), p=0.50, I^2^=0% |
| *CKD stage* |  |  |  |
| - 2, and 1 to 3 | 1 | 16/15 | MD -0.06 (-0.11, -0.01), p=0.01, I^2^=N/A* |
| - 3 to 4 only | 3 | 105/99 | MD -0.10 (-0.24, 0.04), p=0.15, I^2^=0% |
| **Urine Albumin-to-Creatinine Ratio (UACR) [g/gCr]** | | | |
| *Modality* |  |  |  |
| - Aerobic Training | 2 | 37/41 | MD -0.03 (-0.08, 0.02), p=0.31, I^2^=0% |
| - Combined Training | 2 | 88/86 | MD 0.00 (-0.01, 0.01), p=1.00, I^2^=0% |
| *Intervention Duration* |  |  |  |
| - ≤ 12 weeks | 1 | 74/68 | MD 0.00 (-0.01, 0.01), p=1.00, I^2^=N/A |
| - > 12 weeks to ≤ 6 months | 1 | 7/4 | MD -0.09 (-0.49, 0.32), p=0.67, I^2^=N/A |
| - > 6 months | 2 | 44/55 | MD -0.02 (-0.07, 0.03), p=0.36, I^2^=0% |
| *Supervision* |  |  |  |
| - Supervised | 1 | 74/68 | MD 0.00 (-0.01, 0.01), p=1.00, I^2^=N/A |
| - Initially supervised, then unsupervised | 2 | 21/22 | MD -0.00 (-0.29, 0.28), p=0.98, I^2^=0% |
| - Both supervised and unsupervised | 1 | 30/37 | MD -0.03 (-0.08, 0.03), p=0.33, I^2^=N/A |
| *CKD stage* |  |  |  |
| - 2 to 4 only | 2 | 21/22 | MD -0.00 (-0.29, 0.28), p=0.98, I^2^=0% |
| - 3 to 4 only | 2 | 104/105 | MD -0.00 (-0.01, 0.01), p=0.90, I^2^=0% |
| **Urine Protein-to-Creatinine Ratio (UPCR) [g/gCr]** | | | |
| *Modality* |  |  |  |
| - Aerobic Training | 1 | 7/4 | MD -0.11 (-0.64, 0.41), p=0.68, I^2^=N/A |
| - Combined Training | 4 | 125/121 | MD -0.00 (-0.01, 0.01), p=0.92, I^2^=0% |
| *Intervention Duration* |  |  |  |
| - > 12 weeks to ≤ 6 months | 2 | 30/25 | MD -0.03 (-0.26, 0.20), p=0.78, I^2^=0% |
| - > 6 months | 3 | 102/100 | MD -0.00 (-0.01, 0.01), p=0.93, I^2^=0% |
| *Supervision* |  |  |  |
| - Supervised | 1 | 14/14 | MD 0.30 (-0.72, 1.32), p=0.57, I^2^=N/A |
| - Initially supervised, then unsupervised | 3 | 95/90 | MD -0.00 (-0.01, 0.01), p=0.92, I^2^=0% |
| - Both supervised and unsupervised | 1 | 23/21 | MD -0.01 (-0.27, 0.24), p=0.92, I^2^=N/A |
| *CKD stage* |  |  |  |
| - 2 to 4 only | 2 | 21/22 | MD 0.05 (-0.32, 0.41), p=0.81, I^2^=0% |
| - 3 to 4 only | 2 | 88/82 | MD -0.00 (-0.01, 0.01), p=0.92, I^2^=0% |
| **24-hour Urine Protein [g/24hr]** | | | |
| *Modality* |  |  |  |
| - Aerobic Training | 2(3) | 32/19 | MD 0.10 (-0.57, 0.78), p=0.76, I^2^=0% |
| - Combined Training | 1 | 10/11 | MD -0.06 (-2.20, 2.08), p=0.96, I^2^=N/A |
| *Intervention Duration* |  |  |  |
| - > 12 weeks to ≤ 6 months | 2(3) | 32/19 | MD 0.10 (-0.57, 0.78), p=0.76, I^2^=0% |
| - > 6 months | 1 | 10/11 | MD -0.06 (-2.20, 2.08), p=0.96, I^2^=N/A |
| *Supervision* |  |  |  |
| - Supervised | 2 | 23/19 | MD 0.22 (-1.30, 1.74), p=0.78, I^2^=0% |
| - Initially supervised, then unsupervised | 2 | 19/11 | MD 0.07 (-0.65, 0.78), p=0.86, I^2^=2% |
| *CKD stage* |  |  |  |
| - 2 to 4 only | 2 | 17/15 | MD -0.14 (-0.90, 0.63), p=0.73, I^2^=0% |
| - 3 to 4 only | 1(2) | 25/15 | MD 0.64 (-0.55, 1.83), p=0.29, I^2^=0% |
| **Blood Urea Nitrogen (BUN) [mg/dL]** | | | |
| *Modality* |  |  |  |
| - Aerobic Training | 2 | 31/22 | MD 0.96 (-6.22, 8.15), p=0.79, I^2^=0% |
| - Combined Training | 1 | 23/21 | MD -0.20 (-6.85, 6.45), p=0.95, I^2^=N/A |
| - Mind-Body Exercise | 1 | 11/10 | MD -9.17 (-24.39, 6.05), p=0.24, I^2^=N/A |
| *Intervention Duration* |  |  |  |
| - ≤ 12 weeks | 1 | 11/10 | MD -9.17 (-24.39, 6.05), p=0.24, I^2^=N/A |
| - > 12 weeks to ≤ 6 months | 3 | 54/43 | MD 0.34 (-4.54, 5.22), p=0.89, I^2^=0% |
| *Supervision* |  |  |  |
| - Supervised | 1 | 24/18 | MD 1.67 (-5.71, 9.04), p=0.66, I^2^=N/A |
| - Initially supervised, then unsupervised | 2 | 18/14 | MD -9.70 (-23.42, 4.02), p=0.17, I^2^=0% |
| - Both supervised and unsupervised | 1 | 23/21 | MD -0.20 (-6.85, 6.45), p=0.95, I^2^=N/A |
| *CKD stage* |  |  |  |
| - 1 to 5 | 1 | 11/10 | MD -9.17 (-24.39, 6.05), p=0.24, I^2^=N/A |
| - 2 to 4 only | 1 | 7/4 | MD -12.00 (-43.67, 19.67), p=0.46, I^2^=0% |
| - 3 to 4 only | 2 | 47/39 | MD 0.64 (-4.30, 5.58), p=0.80, I^2^=0% |
| **Cardiovascular Risk Factors** | | | |
| **Resting Heat Rate (RHR) [beats/min]** | | | |
| *Modality* |  |  |  |
| - Aerobic Training | 5 | 80/77 | MD -0.65 (-4.41, 3.11), p=0.73, I^2^=12% |
| - Resistance Training | 1 | 30/30 | MD -3.00 (-5.60, -0.40), p=0.02, I^2^= N/A ***** |
| - Combined Training | 3 | 75/80 | MD -1.16 (-5.44, 3.12), p=0.60, I^2^=0% |
| - Mind-Body Exercise | 1 | 11/10 | MD -10.00 (-38.87, 18.87, p=0.50, I^2^=N/A |
| *Intervention Duration* |  |  |  |
| - ≤ 12 weeks | 4 | 59/62 | MD -3.06 (-7.30, 1.17), p=0.16, I^2^=0% |
| - > 12 weeks to ≤ 6 months | 4 | 73/68 | MD -2.05 (-4.33, 0.23), p=0.08, I^2^=34% |
| - > 6 months | 2 | 64/67 | MD -0.05 (-5.10, 5.00), p=0.99, I^2^=0% |
| *Supervision* |  |  |  |
| - Supervised | 3 | 70/67 | MD -0.90 (-5.29, 3.49), p=0.69, I^2^=47% |
| - Initially supervised, then unsupervised | 5 | 99/99 | MD -2.72 (-6.43, 0.98), p=0.15, I^2^=0% |
| - Both supervised and unsupervised | 2 | 27/31 | MD -0.13 (-5.95, 5.69), p=0.97, I^2^=0% |
| *CKD stage* |  |  |  |
| - 1 to 5 | 1 | 11/10 | MD -10.00 (-38.87, 18.87), p=0.50, I^2^=N/A |
| - 2, and 1 to 3 | 1 | 30/30 | MD -3.00 (-5.60, -0.40), p=0.02, I^2^=N/A * |
| - 2 to 4 only | 1 | 7/4 | MD 6.00 (-16.33, 28.33), p=0.60, I^2^=N/A |
| - 3 to 4 only | 4 | 97/103 | MD -1.68 (-5.13, 1.78), p=0.34, I^2^=0% |
| - 3, 4, and 3 to 5 | 3 | 51/50 | MD 0.07 (-4.76, 4.90), p=0.98, I^2^=16% |
| **Blood Pressure** | | | |
| ***Systolic Blood Pressure (SBP) [mmHg]*** | | | |
| *Modality* |  |  |  |
| - Aerobic Training | 5 (6) | 97/74 | MD -5.55 (-12.21, 1.12), p=0.10, I^2^=49% |
| - Resistance Training | 2 | 46/45 | MD -10.35 (-18.57, -2.13), p=0.01, I^2^=86% ***** |
| - Combined Training | 7 | 187/195 | MD 1.89 (-1.91, 5.69), p=0.33, I^2^=0% |
| - Mind-Body Exercise | 1 | 11/10 | MD -17.00 (-68.67, 34.67), p=0.52, I^2^=N/A |
| *Intervention Duration* |  |  |  |
| - ≤ 12 weeks | 2 | 26/26 | MD -7.60 (-20.20, 5.01), p=0.24, I^2^=0% |
| - > 12 weeks to ≤ 6 months | 8 (9) | 197/165 | MD -4.58 (-10.52, 1.36), p=0.13, I^2^=79% |
| - > 6 months | 5 | 118/133 | MD 1.65 (-2.74, 6.04), p=0.46, I^2^=0% |
| *Supervision* |  |  |  |
| - Supervised | 8 | 192/168 | MD -5.48 (-11.11, 0.14), p=0.06, I^2^=75% |
| - Initially supervised, then unsupervised | 6 | 111/109 | MD -0.53 (-8.81, 7.74), p=0.90, I^2^=57% |
| - Both supervised and unsupervised | 2 | 38/47 | MD 6.37 (-1.96, 14.71, p=0.13, I^2^=0% |
| *CKD stage* |  |  |  |
| - 1 to 5 | 1 | 11/10 | MD -17.00 (-68.67, 34.67), p=0.52, I^2^=N/A |
| - 2, and 1 to 3 | 2 | 46/45 | MD -10.35 (-18.57, -2.13), p=0.01, I^2^=83% ***** |
| - 2 to 4 only | 4 | 89/82 | MD 0.98 (-5.31, 7.28), p=0.76, I^2^=0% |
| - 3 to 4 only | 5 (6) | 144/137 | MD -3.04 (-9.93, 3.84), p=0.39, I^2^=68% |
| - 3, 4, and 3 to 5 | 3 | 51/50 | MD 2.49 (-7.80, 12.78), p=0.63, I^2^=47% |
| ***Diastolic Blood Pressure (DBP) [mmHg]*** | | | |
| *Modality* |  |  |  |
| - Aerobic Training | 5 (6) | 97/74 | MD -3.81 (-8.40, 0.78), p=0.10, I^2^=55% |
| - Resistance Training | 2 | 46/45 | MD -8.53 (-21.84, 4.78), p=0.21, I^2^=94 |
| - Combined Training | 6 | 173/177 | MD 0.55 (-1.76, 2.86), p=0.64, I^2^=0% |
| - Mind-Body Exercise | 1 | 11/10 | MD -11.00 (-43.32, 21.32), p=0.50, I^2^=N/A |
| *Intervention Duration* |  |  |  |
| - ≤ 12 weeks | 2 | 26/26 | MD -5.33 (-12.95, 2.29), p=0.17, I^2^=0% |
| - > 12 weeks to ≤ 6 months | 8 (9) | 197/165 | MD -3.59 (-9.56, 2.38), p=0.24, I^2^=89% |
| - > 6 months | 4 | 104/115 | MD 0.38 (-2.31, 3.07), p=0.78, I^2^=0% |
| *Supervision* |  |  |  |
| - Supervised | 8 | 192/168 | MD -3.25 (-9.60, 3.09), p=0.32, I^2^=91% |
| - Initially supervised, then unsupervised | 5 | 97/91 | MD -2.23 (-6.34, 1.87), p=0.29, I^2^=21% |
| - Both supervised and unsupervised | 2 | 38/47 | MD 1.62 (-3.06, 6.30), p=0.50, I^2^=0% |
| *CKD stage* |  |  |  |
| - 1 to 5 | 1 | 11/10 | MD -11.00 (-43.32, 21.32), p=0.50, I^2^=N/A |
| - 2, and 1 to 3 | 2 | 46/45 | MD -8.53 (-21.84, 4.78), p=0.21, I^2^=94% |
| - 2 to 4 only | 3 | 75/64 | MD 1.17 (-3.36, 5.69), p=0.61, I^2^=0% |
| - 3 to 4 only | 5 (6) | 144/137 | MD -2.33 (-6.33, 1.67), p=0.25, I^2^=64% |
| - 3, 4, and 3 to 5 | 3 | 51/50 | MD -0.14 (-5.07, 4.78), p=0.95, I^2^=12% |
| ***Ambulatory 24-hour Systolic Blood Pressure [mmHg]*** | | | |
| *Modality* |  |  |  |
| - Aerobic Training | 1 | 25/21 | MD 4.52 (-4.83, 13.87) p=0.34, I^2^=N/A |
| - Resistance Training | 1 | 30/30 | MD -12.50 (-17.76, -7.24), p<0.00001, I^2^=N/A ***** |
| - Combined Training | 2 | 25/33 | MD 4.65 (-2.82, 12.12), p=0.22, I^2^=0% |
| *Intervention Duration* |  |  |  |
| - > 12 weeks to ≤ 6 months | 3 | 70/73 | MD -1.86 (-14.45, 10.73), p=0.77, I^2^=86% |
| - > 6 months | 1 | 10/11 | MD 5.50 (-5.82, 16.82), p=0.34, I^2^=N/A |
| *Supervision* |  |  |  |
| - Supervised | 3 | 65/62 | MD -1.46 (-14.68, 11.77), p=0.83, I^2^=86% |
| - Initially supervised, then unsupervised | 1 | 15/22 | MD 4.00 (-5.94, 13.94), p=0.43, I^2^=N/A |
| - *CKD stage* |  |  |  |
| - 2, and 1 to 3 | 1 | 30/30 | MD -12.50 (-17.76, -7.24), p<0.00001, I^2^=N/A ***** |
| - 2 to 4 only | 1 | 10/11 | MD 5.50 (-5.82, 16.82), p=0.34, I^2^=N/A |
| - 3, 4, and 3 to 5 | 2 | 40/43 | MD 4.28 (-2.54, 11.09), p=0.22, I^2^=84% |
| ***Ambulatory 24-hour Diastolic Blood Pressure [mmHg]*** | | | |
| *Modality* |  |  |  |
| - Aerobic Training | 1 | 25/21 | MD 4.30 (-2.01, 10.61) p=0.18, I^2^=N/A |
| - Resistance Training | 1 | 30/30 | MD -11.80 (-17.17, -6.43), p<0.0001, I^2^=N/A ***** |
| - Combined Training | 2 | 25/33 | MD 2.28 (-3.22, 7.79), p=0.42, I^2^=0% |
| *Intervention Duration* |  |  |  |
| - > 12 weeks to ≤ 6 months | 3 | 70/73 | MD -1.95 (-12.27, 8.38), p=0.71, I^2^=89% |
| - > 6 months | 1 | 10/11 | MD 3.20 (-8.14, 14.54), p=0.58, I^2^=N/A |
| *Supervision* |  |  |  |
| - Supervised | 3 | 65/62 | MD -1.83 (-13.70, 10.04), p=0.76, I^2^=88% |
| - Initially supervised, then unsupervised | 1 | 15/22 | MD 2.00 (-4.30, 8.30), p=0.53, I^2^=N/A |
| *CKD stage* |  |  |  |
| - 2, and 1 to 3 | 1 | 30/30 | MD -11.80 (-17.17, -6.43), p<0.0001, I^2^=N/A ***** |
| - 2 to 4 only | 1 | 10/11 | MD 3.20 (-8.14, 14.54), p=0.58, I^2^=N/A |
| - 3, 4, and 3 to 5 | 2 | 40/43 | MD 3.15 (-1.31, 7.61), p=0.17, I^2^=0% |
| **Endothelial Function** | | | |
| ***Pulse Wave Velocity (PWV) aortic [m/s]*** | | | |
| *Modality* |  |  |  |
| - Aerobic Training | 3 | 59/58 | MD 0.55 (-0.09, 1.19), p=0.09, I^2^=0% |
| - Combined Training | 3 | 67/69 | MD -0.56 (-2.19, 1.07), p=0.50, I^2^=71% |
| *Intervention Duration* |  |  |  |
| - ≤ 12 weeks | 2 | 34/37 | MD 0.62 (-0.09, 1.33), p=0.09, I^2^=0% |
| - > 12 weeks to ≤ 6 months | 2 | 48/44 | MD 0.17 (-0.99, 1.34), p=0.77, I^2^=0% |
| - > 6 months | 2 | 44/46 | MD -1.00 (-3.82, 1.83), p=0.49, I^2^=86% |
| *Supervision* |  |  |  |
| - Supervised | 2 | 40/37 | MD 0.18 (-0.91, 1.28), p=0.74, I^2^=0% |
| - Unsupervised | 1 | 23/23 | MD 0.10 (-1.74, 1.94), p=0.91, I^2^=N/A |
| - Initially supervised, then unsupervised | 1 | 36/36 | MD 0.30 (-0.50, 1.10), p=0.46, I^2^=N/A |
| - Both supervised and unsupervised | 2 | 27/31 | MD -0.80 (-4.06, 2.47), p=0.63, I^2^=89% |
| *CKD stage* |  |  |  |
| - 3 to 4 only | 3 | 63/67 | MD -0.16 (-1.47, 1.14), p=0.81, I^2^=78% |
| - 3, 4, and 3 to 5 | 3 | 63/60 | MD 0.16 (-0.78, 1.10), p=0.74, I^2^=0% |
| ***Augmentation Index (AIx) central arterial [%]*** | | | |
| *Modality* |  |  |  |
| - Aerobic Training | 3 | 44/47 | MD 3.60 (-2.00, 9.20), p=0.21, I^2^=15% |
| - Combined Training | 2 | 47/47 | MD 0.63 (-3.24, 4.50), p=0.75, I^2^=0% |
| *Intervention Duration* |  |  |  |
| - ≤ 12 weeks | 2 | 34/37 | MD 3.04 (-5.77, 11.85), p=0.50, I^2^=58% |
| - > 12 weeks to ≤ 6 months | 1 | 11/11 | MD -5.00 (-27.42, 17.42), p=0.66, I^2^=NA |
| - > 6 months | 2 | 46/46 | MD 1.10 (-2.63, 4.84), p=0.56, I^2^=0% |
| *Supervision* |  |  |  |
| - Supervised | 1 | 15/16 | MD -2.00 (-11.19, 7.19), p=0.67, I^2^=N/A |
| - Initially supervised, then unsupervised | 2 | 47/47 | MD 0.63 (-3.24, 4.50), p=0.75, I^2^=0% |
| - Both supervised and unsupervised | 2 | 29/31 | MD 6.27 (0.24, 12.30), p=0.04, I^2^=0% ***** |
| *CKD stage* |  |  |  |
| - 3 to 4 only | 2 | 55/57 | MD 3.23 (-2.74, 9.21), p=0.29, I^2^=57% |
| - 3, 4, and 3 to 5 | 3 | 36/37 | MD -0.30 (-7.24, 6.64), p=0.93, I^2^=0% |
| ***Asymmetric dimethylarginine (AMDA) [umol/L]*** | | | |
| *Modality* |  |  |  |
| - Resistance Training | 1 | 16/15 | MD -0.81 (-1.22, - 0.40), p<0.00010, I^2^=N/A * |
| - Combined Training | 2 | 33/32 | MD -0.12 (-0.36, 0.11), p=0.30, I^2^=84% |
| *Intervention Duration* |  |  |  |
| - > 12 weeks to ≤ 6 months | 3 | 49/47 | MD -0.30 (-0.63, 0.02), p=0.07, I^2^=90% |
| *Supervision* |  |  |  |
| - Supervised | 2 | 26/26 | MD -0.50 (-1.04, 0.03), p=0.07, I^2^=83% |
| - Both supervised and unsupervised | 1 | 23/21 | MD -0.02 (-0.07, 0.03), p=0.41, I^2^=N/A |
| *CKD stage* |  |  |  |
| - 2, and 1 to 3 | 1 | 16/15 | MD -0.81 (-1.22, - 0.40), p<0.00010, I^2^=N/A * |
| - 2 to 4 only | 1 | 10/11 | MD -0.26 (-0.44, - 0.08), p=0.005, I^2^=N/A * |
| - 3 to 4 only | 1 | 23/21 | MD -0.02 (-0.07, 0.03), p=0.41, I^2^=N/A |
| **Lipids and Blood Parameters** | | | |
| ***Triglyceride (TG) [mg/dL]*** | | | |
| *Modality* |  |  |  |
| - Aerobic Training | 3 | 44/36 | MD -12.80 (-48.63, 23.04), p=0.48, I^2^=0% |
| - Resistance Training | 2 | 51/50 | MD -13.62 (-18.13, -9.10), p<0.00001, I^2^=0 ***** |
| - Combined Training | 4 | 73/83 | MD -3.55 (-20.75, 13.66), p=0.69, I^2^=0% |
| - Mind-Body Exercise | 1 | 11/10 | MD -15.94 (-186.95, 155.06), p=0.85, I^2^=N/A |
| *Intervention Duration* |  |  |  |
| - ≤ 12 weeks | 1 | 11/10 | MD -15.94 (-186.95, 155.06), p=0.84, I^2^=N/A |
| - > 12 weeks to ≤ 6 months | 5 | 96/938 | MD -13.53 (-18.02, -9.05), p<0.00001, I^2^=0% ***** |
| - > 6 months | 4 | 72/76 | MD -1.96 (-28.10, 24.18), p=0.88, I^2^=19% |
| *Supervision* |  |  |  |
| - Supervised | 4 | 86/82 | MD -13.48 (-17.96, -9.00), p<0.00001, I^2^=0% ***** |
| - Unsupervised | 1 | 12/11 | MD -58.46 (-151.18, 34.27), p=0.22, I^2^=N/A |
| - Initially supervised, then unsupervised | 5 | 81/86 | MD -3.59 (-21.08, 13.91), p=0.69, I^2^=0% |
| *CKD stage* |  |  |  |
| - 1 to 5 | 1 | 10/11 | MD -15.94 (-186.95, 155.06), p=0.84, I^2^=N/A |
| - 2, and 1 to 3 | 2 | 51/50 | MD -13.62 (-18.13, -9.10), p<0.00001, I^2^=0% ***** |
| - 2 to 4 only | 3 | 31/33 | MD 29.26 (-13.83, 72.34), p=0.18, I^2^=0% |
| - 3 to 4 only | 2 | 48/47 | MD -11.92 (-39.29, 15.44), p=0.39, I^2^=9% |
| - 3, 4, and 3 to 5 | 2 | 38/39 | MD -13.81 (-53.70, 26.09), p=0.50, I^2^=0% |
| ***Total Cholesterol (TC) [mg/dL]*** | | | |
| *Modality* |  |  |  |
| - Aerobic Training | 5 | 108/106 | MD 4.50 (-15.24, 24.24), p=0.66, I^2^=77% |
| - Resistance Training | 2 | 51/50 | MD -1.29 (-7.41, 4.84), p=0.68, I^2^=0% |
| - Combined Training | 6 | 139/142 | MD 9.58 (-2.33, 21.49), p=0.11, I^2^=38% |
| - Mind-Body Exercise | 1 | 11/10 | MD -24.75 (-114.57, 65.07), p=0.59, I^2^=N/A |
| *Intervention Duration* |  |  |  |
| - ≤ 12 weeks | 3 | 75/80 | MD -7.75 (-42.13, 26.63), p=0.66, I^2^=87% |
| - > 12 weeks to ≤ 6 months | 5 | 154/141 | MD 1.26 (-3.85, 6.37), p=0.63, I^2^=0% |
| - > 6 months | 5 | 80/96 | MD 15.71 (-2.25, 33.67), p=0.09, I^2^=52% |
| *Supervision* |  |  |  |
| - Supervised | 5 | 144/131 | MD 5.48 (-3.31, 14.27), p=0.22, I^2^=42% |
| - Unsupervised | 2 | 57/60 | MD -4.06 (-50.31, 42.18), p=0.86, I^2^=84% |
| - Initially supervised, then unsupervised | 5 | 81/86 | MD 5.35 (-10.47, 21.17), p=0.51, I^2^=27% |
| - Both supervised and unsupervised | 2 | 27/31 | MD 13.65 (1.92, 25.38), p=0.02, I^2^=0%***** |
| *CKD stage* |  |  |  |
| - 1 to 5 | 1 | 11/10 | MD -24.75 (-114.57, 65.07), p=0.59, I^2^=N/A |
| - 2, and 1 to 3 | 2 | 51/50 | MD -1.29 (-7.41, 4.84), p=0.68, I^2^=0% |
| - 2 to 4 only | 4 | 89/82 | MD 15.20 (1.48, 29.81), p=0.03, I^2^=30%***** |
| - 3 to 4 only | 5 | 120/127 | MD 0.90 (-18.18, 19.97), p=0.93, I^2^=77% |
| - 3, 4, and 3 to 5 | 2 | 38/39 | MD 9.30 (-9.15, 27.74), p=0.32, I^2^=56% |
| ***Low Density Lipoprotein (LDL-C) [mg/dL]*** | | | |
| *Modality* |  |  |  |
| - Aerobic Training | 3 | 51/46 | MD 8.49 (0.72, 16.26), p=0.03, I^2^=0% ***** |
| - Resistance Training | 2 | 51/50 | MD -3.84 (-8.02, 0.35), p=0.07, I^2^=N/A |
| - Combined Training | 7 | 160/159 | MD 12.83 (0.23, 25.43), p=0.05, I^2^=58% ***** |
| - Mind-Body Exercise | 1 | 11/10 | MD -13.92 (-94.30, 66.46), p=0.73, I^2^=N/A |
| *Intervention Duration* |  |  |  |
| - ≤ 12 weeks | 2 | 30/31 | MD 8.56 (-0.36, 17.48), p=0.06, I^2^=0% |
| - > 12 weeks to ≤ 6 months | 7 | 175/159 | MD 0.41 (-4.69, 5.52), p=0.87, I^2^=14% |
| - > 6 months | 4 | 68/75 | MD 18.08 (-10.15, 46.31), p=0.21, I^2^=78% |
| *Supervision* |  |  |  |
| - Supervised | 5 | 142/129 | MD 7.14 (-4.43, 18.70), p=0.23, I^2^=77% |
| - Initially supervised, then unsupervised | 5 | 81/84 | MD 5.70 (-9.01, 20.41), p=0.45, I^2^=39% |
| - Both supervised and unsupervised | 3 | 50/52 | MD 9.21 (0.85, 17.56), p=0.03, I^2^=0% ***** |
| *CKD stage* |  |  |  |
| - 1 to 5 | 1 | 11/10 | MD -13.92 (-94.30, 66.46), p=0.73, I^2^=N/A |
| - 2, and 1 to 3 | 2 | 51/50 | MD -3.84 (-8.02, 0.35), p=0.07, I^2^=0% |
| - 2 to 4 only | 4 | 87/80 | MD 17.37 (-0.20, 34.94), p=0.05, I^2^=68% ***** |
| - 3 to 4 only | 4 | 86/88 | MD 4.48 (-4.93, 13.90), p=0.35, I^2^=19% |
| - 3, 4, and 3 to 5 | 2 | 38/37 | MD 11.91 (-3.72, 27.54, p=0.14, I^2^=0% |
| ***High Density Lipoprotein (HDL-C) [mg/dL]*** | | | |
| *Modality* |  |  |  |
| - Aerobic Training | 3 | 51/46 | MD 3.35 (-0.58, 7.27), p=0.09, I^2^=0% |
| - Resistance Training | 2 | 51/50 | MD 3.82 (0.03, 7.62), p=0.05, I^2^=0% * |
| - Combined Training | 7 | 162/162 | MD 0.02 (-2.04, 2.08), p=0.98, I^2^=0% |
| - Mind-Body Exercise | 1 | 11/10 | MD 5.41 (-51.19, 62.02), p=0.85, I^2^=N/A |
| *Intervention Duration* |  |  |  |
| - ≤ 12 weeks | 2 | 30/31 | MD 3.80 (-1.31, 8.92), p=0.15, I^2^=0% |
| - > 12 weeks to ≤ 6 months | 7 | 177/162 | MD 1.49 (-0.67, 3.64), p=0.18, I^2^=0% |
| - > 6 months | 4 | 68/75 | MD 0.21 (-2.72, 3.14), p=0.89, I^2^=0% |
| *Supervision* |  |  |  |
| - Supervised | 5 | 144/131 | MD 1.36 (-0.91, 3.63), p=0.24, I^2^=0% |
| - Initially supervised, then unsupervised | 5 | 75/75 | MD 0.28 (-2.55, 3.10), p=0.85, I^2^=0% |
| - Both supervised and unsupervised | 3 | 50/52 | MD 4.02 (-0.54, 8.59), p=0.08, I^2^=0% |
| *CKD stage* |  |  |  |
| - 1 to 5 | 1 | 11/10 | MD 5.41 (-51.19, 62.02), p=0.85, I^2^=N/A |
| - 2, and 1 to 3 | 2 | 51/50 | MD 3.82 (0.03, 7.62), p=0.05, I^2^=0% * |
| - 2 to 4 only | 4 | 89/82 | MD 0.12 (-2.37, 2.61), p=0.92, I^2^=0% |
| - 3 to 4 only | 4 | 86/88 | MD 1.83 (-1.57, 5.23), p=0.29, I^2^=15% |
| - 3, 4, and 3 to 5 | 2 | 32/28 | MD 1.23 (-8.78, 11.24), p=0.81, I^2^=0% |
| **Glycosylated Haemoglobin (HbA1c) [%]** | | | |
| *Modality* |  |  |  |
| - Aerobic Training | 3(4) | 51/36 | MD -0.22 (-0.67, 0.22), p=0.32, I^2^=26% |
| - Resistance Training | 2 | 51/50 | MD -0.60 (-0.71, -0.50), p<0.00001, I^2^=0% ***** |
| - Combined Training | 7 | 138/144 | MD 0.02 (-0.30, 0.33), p=0.91, I^2^=18% |
| *Intervention Duration* |  |  |  |
| - ≤ 12 weeks | 1(2) | 25/15 | MD -0.60 (-1.16, -0.04), p=0.04, I^2^=0% ***** |
| - > 12 weeks to ≤ 6 months | 7 | 130/122 | MD -0.22 (-0.53, 0.08), p=0.15, I^2^=79% |
| - > 6 months | 4 | 85/93 | MD -0.02 (-0.75, 0.72), p=0.96, I^2^=47% |
| *Supervision* |  |  |  |
| - Supervised | 5 | 88/78 | MD -0.45 (-0.71, -0.18), p=0.0009, I^2^=67% * |
| - Unsupervised | 1 | 23/23 | MD 0.30 (-0.43, 1.03), p=0.42, I^2^=N/A |
| - Initially supervised, then unsupervised | 5 | 76/72 | MD -0.42 (-0.87, 0.03), p=0.07, I^2^=0% |
| - Both supervised and unsupervised | 2 | 53/57 | MD 0.10 (-0.22, 0.42), p=0.54, I^2^=0% |
| *CKD stage* |  |  |  |
| - 2, and 1 to 3 | 2 | 51/50 | MD -0.60 (-0.71, -0.50), p<0.00001, I^2^=0% ***** |
| - 2 to 4 only | 3 | 26/25 | MD 0.80 (-0.33, 1.93), p=0.16, I^2^=0% |
| - 3 to 4 only | 5 (6) | 133/125 | MD -0.17 (-0.47, 0.13), p=0.27, I^2^=40% |
| - 3, 4, and 3 to 5 | 2 | 30/30 | MD 0.16 (-0.46, 0.77), p=0.62, I^2^=0% |
| **Blood Glucose (BG) [mg/dL]** | | | |
| *Modality* |  |  |  |
| - Aerobic Training | 2 | 69/67 | MD 2.80 (-6.43, 12.03), p=0.55, I^2^=0% |
| - Resistance Training | 1 | 35/35 | MD -18.00 (-22.02, -13.98), p<0.00001, I^2^=N/A ***** |
| - Combined Training | 1 | 58/49 | MD -9.80 (-22.44, 2.84), p=0.13, I^2^=N/A |
| *Intervention Duration* |  |  |  |
| - ≤ 12 weeks | 1 | 45/49 | MD 3.44 (-9.50, 16.38), p=0.60, I^2^=N/A |
| - > 12 weeks to ≤ 6 months | 3 | 117/102 | MD -9.77 (-21.91, 2.37), p=0.11, I^2^=78% |
| *Supervision* |  |  |  |
| - Supervised | 2 | 82/67 | MD -3.98 (-15.67, 7.71), p=0.50, I^2^=39% |
| - Unsupervised | 2 | 80/84 | MD -8.20 (-29.13, 12.73), p=0.44, I^2^=90% |
| *CKD stage* |  |  |  |
| - 2, and 1 to 3 | 1 | 35/35 | MD -18.00 (-22.02, -13.98), p<0.00001, I^2^=N/A ***** |
| - 2 to 4 only | 1 | 58/48 | MD -9.80 (-22.44, 2.84), p=0.13, I^2^=N/A |
| - 3 to 4 only | 1 | 69/67 | MD 2.80 (-6.43, 12.03), p=0.55, I^2^=0% |
| **Haemoglobin (Hb) [g/dL]** | | | |
| *Modality* |  |  |  |
| - Aerobic Training | 3 (4) | 70/62 | MD 0.30 (0.01, 0.59), p=0.04, I^2^=0% ***** |
| - Combined Training | 2 | 59/57 | MD -0.06 (-0.66, 0.54), p=0.85, I^2^=0% |
| *Intervention Duration* |  |  |  |
| - ≤ 12 weeks | 2 (3) | 63/58 | MD 0.31 (0.02, 0.61), p=0.04, I^2^=0% ***** |
| - > 12 weeks to ≤ 6 months | 2 | 30/25 | MD 0.11 (-0.83, 1.05), p=0.82, I^2^=0% |
| - > 6 months | 1 | 36/36 | MD -0.19 (-0.93, 0.55), p=0.61, I^2^=N/A |
| *Supervision* |  |  |  |
| - Supervised | 1 | 10/5 | MD 0.20 (-0.17, 0.57), p=0.28, I^2^=N/A |
| - Unsupervised | 1 | 45/49 | MD 0.54 (-0.22, 1.30), p=0.16, I^2^=N/A |
| - Initially supervised, then unsupervised | 3 | 51/44 | MD 0.18 (-0.32, 0.67), p=0.49, I^2^=6% |
| - Both supervised and unsupervised | 1 | 23/21 | MD 0.20 (-0.84, 1.24), p=0.71, I^2^=N/A |
| *CKD stage* |  |  |  |
| - 2 to 4 only | 1 | 7/4 | MD -0.30 (-2.50, 1.90), p=0.79, I^2^=N/A |
| - 3 to 4 only | 4 (5) | 122/115 | MD 0.24 (-0.02, 0.51), p=0.07, I^2^=0% |
| **Body composition parameters** | | | |
| ***Waist Circumference [cm]*** | | | |
| *Modality* |  |  |  |
| - Aerobic Training | 1 (2) | 18/9 | MD -3.14 (-4.67, -1.60), p<0.0001, I^2^=0% ***** |
| - Combined Training | 4 | 77/80 | MD -3.08 (-5.26, -0.89), p=0.006, I^2^=0% ***** |
| *Intervention Duration* |  |  |  |
| - ≤ 12 weeks | 1 (2) | 18/9 | MD -3.14 (-4.67, -1.60), p<0.0001, I^2^=0% ***** |
| - > 12 weeks to ≤ 6 months | 1 | 23/23 | MD -1.70 (-8.54, 5.14), p=0.63, I^2^=N/A |
| - > 6 months | 3 | 54/57 | MD -3.23 (-5.54, -0.93), p=0.006, I^2^=0% ***** |
| *Supervision* |  |  |  |
| - Supervised | 2 | 20/16 | MD -3.38 (-5.57, -1.18), p=0.003, I^2^=0% ***** |
| - Unsupervised | 1 | 23/23 | MD -1.70 (-8.54, 5.14), p=0.63, I^2^=N/A |
| - Initially supervised, then unsupervised | 2 | 44/40 | MD -2.94 (-4.54, -1.35), p=0.0003, I^2^=0% ***** |
| - Both supervised and unsupervised | 1 | 8/10 | MD -7.20 (-16.83, 2.43), p=0.14, I^2^=N/A |
| *CKD stage* |  |  |  |
| - 2 to 4 only | 1 | 10/11 | MD -2.80 (-13.95,8.353), p=0.62, I^2^=N/A |
| - 3 to 4 only | 3 (4) | 62/55 | MD -3.17 (-4.46, -1.88), p<0.00001, I^2^=0% ***** |
| - 3, 4, and 3 to 5 | 1 | 23/23 | MD -1.70 (-8.54, 5.14), p=0.63, I^2^=N/A |
| ***Body Weight [kg]*** | | | |
| *Modality* |  |  |  |
| - Aerobic Training | 2 | 31/19 | MD 0.26 (-6.80, 7.32), p=0.94, I^2^=0% |
| - Resistance Training | 2 | 49/47 | MD -0.35 (-8.93, 8.23), p=0.94, I^2^=93% |
| - Combined Training | 5 | 160/152 | MD -2.20 (-3.99, -0.42), p=0.02, I^2^=10% ***** |
| *Intervention Duration* |  |  |  |
| - ≤ 12 weeks | 1 | 14/12 | MD 3.80 (2.20, 5.40), p<0.00001, I^2^=N/A ***** |
| - > 12 weeks to ≤ 6 months | 5 | 134/117 | MD -1.57 (-4.41, 1.26), p=0.28, I^2^=18% |
| - > 6 months | 3 | 92/89 | MD -3.16 (-4.88, -1.43), p=0.0003, I^2^=0% ***** |
| *Supervision* |  |  |  |
| - Supervised | 4 | 117/108 | MD -0.58 (-5.06, 3.90), p=0.80, I^2^=85% |
| Unsupervised | 1 | 24/15 | MD 0.03 (-7.31, 7.38), p=0.99, I^2^=N/A |
| - Initially supervised, then unsupervised | 3 | 91/85 | MD -1.12 (-7.63, 5.38), p=0.74, I^2^=19% |
| - Both supervised and unsupervised | 1 | 8/10 | MD -5.60 (-18.94, 7.74), p=0.41, I^2^=N/A |
| *CKD stage* |  |  |  |
| - 2, and 1 to 3 | 1 | 35/35 | MD -4.97 (-9.27, -0.67), p=0.02, I^2^=N/A * |
| - 2 to 4 only | 3 | 75/65 | MD -0.78 (-3.41, 1.86), p=0.56, I^2^=0% |
| - 3 to 4 only | 4 | 120/105 | MD -0.35 (-5.59, 4.89), p=0.90, I^2^=91% |
| - 3, 4, and 3 to 5 | 1 | 10/13 | MD 10.30 (-7.10, 27.70), p=0.25, I^2^=N/A |
| ***Body Mass Index (BMI) [kg/m^2^]*** | | | |
| *Modality* |  |  |  |
| - Aerobic Training | 4 (5) | 94/75 | MD -0.75 (-1.52, 0.01), p=0.05, I^2^=0% ***** |
| - Resistance Training | 2 | 49/47 | MD -0.24 (-2.91, 2.44), p=0.86, I^2^=96% |
| - Combined Training | 7 | 169/180 | MD -1.05 (-1.63, -0.47), p=0.0004, I^2^=0% ***** |
| *Intervention Duration* |  |  |  |
| - ≤ 12 weeks | 2 | 33/33 | MD 0.18 (-1.66, 2.03), p=0.85, I^2^=93% |
| - > 12 weeks to ≤ 6 months | 7 | 143/125 | MD -1.27 (-2.08, -0.46), p=0.002, I^2^=0% ***** |
| - > 6 months | 5 | 136/144 | MD -1.13 (-1.73, -0.53), p=0.0002, I^2^=0% ***** |
| *Supervision* |  |  |  |
| - Supervised | 6 | 122/105 | MD -0.33 (-2.15, 1.48), p=0.72, I^2^=80% |
| - Unsupervised | 1 | 23/23 | MD -0.40 (-3.02, 2.22), p=0.76, I^2^=N/A |
| - Initially supervised, then unsupervised | 4 | 110/106 | MD -1.01 (-1.62, -0.40), p=0.001, I^2^=0% ***** |
| - Both supervised and unsupervised | 3 | 57/68 | MD -0.93 (-1.71, -0.14), p=0.02, I^2^=0% ***** |
| *CKD stage* |  |  |  |
| - 2, and 1 to 3 | 1 | 35/35 | MD -1.63 (-2.57, -0.69), p=0.0007, I^2^=N/A ***** |
| - 2 to 4 only | 2 | 24/29 | MD 0.13 (-3.07, 3.33), p=0.94, I^2^=0% |
| - 3 to 4 only | 7 (8) | 198/181 | MD -0.62 (-1.78, 0.54), p=0.30, I^2^=79% |
| - 3, 4, and 3 to 5 | 3 | 58/57 | MD 0.07 (-2.02, 2.16), p=0.95, I^2^=0% |
| ***Body Fat [%]*** | | | |
| *Modality* |  |  |  |
| - Aerobic Training | 3 | 58/51 | MD 0.10 (-3.23, 3.42), p=0.96, I^2^=0% |
| - Resistance Training | 1 | 35/35 | MD -3.95 (-5.85, - 2.05), p<0.0001, I^2^=N/A ***** |
| - Combined Training | 1 | 14/18 | MD -0.90 (-5.60, 3.80), p=0.71, I^2^=N/A |
| *Intervention Duration* |  |  |  |
| - ≤ 12 weeks | 1 | 14/15 | MD 0.70 (-6.44, 7.84), p=0.85, I^2^=N/A |
| - > 12 weeks to ≤ 6 months | 3 | 79/71 | MD -2.15 (-5.32, 1.03), p=0.19, I^2^=48% |
| - > 6 months | 1 | 14/18 | MD -0.90 (-5.60, 3.80), p=0.71, I^2^=N/A |
| *Supervision* |  |  |  |
| - Supervised | 3 | 79/71 | MD -2.15 (-5.32, 1.03), p=0.19, I^2^=48% |
| - Initially supervised, then unsupervised | 2 | 28/33 | MD -0.42 (-4.34, 3.51), p=0.84, I^2^=0% |
| *CKD stage* |  |  |  |
| - 2, and 1 to 3 | 1 | 35/35 | MD -3.95 (-5.85, -2.05), p<0.0001, I^2^=N/A ***** |
| - 2 to 4 only | 1 | 14/18 | MD -0.90 (-5.60, 3.80), p=0.71, I^2^=N/A |
| - 3 to 4 only | 2 | 33/30 | MD 1.15 (-3.14, 5.43), p=0.60, I^2^=0% |
| - 3, 4, and 3 to 5 | 1 | 25/21 | MD -1.50 (-6.78, 3.78), p=0.58, I^2^=N/A |
| ***Lean Body Mass (LBM) [kg]*** | | | |
| *Modality* |  |  |  |
| - Aerobic Training | 2(3) | 43/30 | MD 0.46 (-0.63, 1.55), p=0.40, I^2^=69% |
| - Resistance Training | 2 | 51/50 | MD 1.41 (0.41, 2.42), p=0.006, I^2^=0% ***** |
| - Combined Training | 2 | 24/31 | MD 2.99 (-2.09, 8.07), p=0.25, I^2^=0% |
| *Intervention Duration* |  |  |  |
| - ≤ 12 weeks | 1(2) | 18/9 | MD 0.44 (-0.73, 1.61), p=0.46, I^2^=84% |
| - > 12 weeks to ≤ 6 months | 4 | 86/84 | MD 1.47 (0.48, 2.47), p=0.004, I^2^=0% ***** |
| - > 6 months | 1 | 14/18 | MD 1.80 (-3.91, 7.51), p=54, I^2^= N/A |
| *Supervision* |  |  |  |
| - Supervised | 4 | 86/76 | MD 1.24 (0.59, 1.88), p=0.0002, I^2^=0% ***** |
| - Initially supervised, then unsupervised | 3 | 32/35 | MD 0.20 (-1.42, 1.82), p=0.81, I^2^=10% |
| *CKD stage* |  |  |  |
| - 2, and 1 to 3 | 1 | 51/50 | MD 1.41 (0.41, 2.42), p=0.006, I^2^=0% ***** |
| - 2 to 4 only | 1 | 14/18 | MD 1.80 (-3.91, 7.51), p=0.54, I^2^=N/A |
| - 3 to 4 only | 1 (2) | 18/9 | MD 0.44 (-0.73, 1.61), p=0.46, I^2^=84% |
| - 3, 4, and 3 to 5 | 2 | 35/34 | MD 4.84 (-2.53, 12.21), p=0.20, I^2^=0% |
| ***Interleukin-6 (IL-6) [pg/mL]*** | | | |
| *Modality* |  |  |  |
| - Aerobic Training | 2 | 48/33 | MD -1.08 (-2.29, 0.12), p=0.08, I^2^=0% |
| - Resistance Training | 1 | 16/15 | MD -3.64 (-6.06, -1.22), p=0.003, I^2^=N/A * |
| - Combined Training | 2 | 33/34 | MD -3.60 (-9.25, 2.06), p=0.21, I^2^=89% |
| *Intervention Duration* |  |  |  |
| - > 12 weeks to ≤ 6 months | 4 | 87/71 | MD -2.74 (-4.87, - 0.62), p=0.01, I^2^=74% * |
| - > 6 months | 1 | 10/11 | MD -0.90 (-2.51, 0.71), p=0.27, I^2^=NA |
| *Supervision* |  |  |  |
| - Supervised | 3 | 50/44 | MD -1.64 (-3.10, - 0.18), p=0.03, I^2^=0% * |
| - Unsupervised | 2 | 47/38 | MD -3.67 (-9.19, 1.84), p=0.19, I^2^=88% |
| *CKD stage* |  |  |  |
| - 2, and 1 to 3 | 1 | 16/15 | MD -3.64 (-6.06, -1.22), p=0.003, I^2^=N/A * |
| - 2 to 4 only | 1 | 10/11 | MD -0.90 (-2.51, 0.71), p=0.27, I^2^=NA |
| - 3 to 4 only | 2 | 48/33 | MD -1.08 (-2.29, 0.12), p=0.08, I^2^=0% |
| - 3, 4, and 3 to 5 | 1 | 23/23 | MD -6.68 (-10.05, -3.32), p<0.0001, I^2^=N/A * |
| ***C-Reactive-Protein (CRP) [mg/L]*** | | | |
| *High sensitivity C-Reactive-Protein (hs-CRP) [mg/L]* | | | |
|  | 6 | 157/136 | MD 0.00 (-0.01, 0.01), p=0.97, I^2^=28% |
| *C-Reactive-Protein (non hS-CRP) [mg/L]* | | | |
|  | 6 | 98/108 | MD 0.57 (-2.38, 3.52), p=0.71, I^2^=3% |
| *hs-CRP and non hs-CRP combined Modality* |  |  |  |
| - Aerobic Training | 4 | 75/61 | MD -0.00 (-0.02, 0.01), p=0.65, I^2^=0% |
| - Combined Training | 8 | 180/183 | MD -0.33 (-1.96, 1.29), p=0.69, I^2^=37% |
| *hs-CRP and non hs-CRP combined Intervention Duration* | |  |  |
| ≤ 12 weeks | 1 | 19/21 | MD -0.00 (-0.02, 0.01), p=0.65, I^2^=N/A |
| > 12 weeks to ≤ 6 months | 7 | 168/148 | MD -0.16 (-1.53, 1.21), p=0.82, I^2^=34% |
| > 6 months | 4 | 68/75 | MD 0.00 (-1.80, 1.80), p=1.00, I^2^=0% |
| *hs-CRP and non hs-CRP combined Supervision* |  |  |  |
| - Supervised | 3 | 91/79 | MD -1.25 (-4.27, 1.76), p=0.42, I^2^=67% |
| - Unsupervised | 2 | 47/38 | MD 0.14 (-2.30, 2.57), p=0.91, I^2^=0% |
| - Initially supervised, then unsupervised | 4 | 67/75 | MD 1.93 (-4.02, 7.88), p=0.52, I^2^=40% |
| - Both supervised and unsupervised | 3 | 50/52 | MD 0.00 (-0.00, 0.01), p=0.25, I^2^=0% |
| *CKD stage* |  |  |  |
| - 2 to 4 only | 4 | 87/80 | MD -0.93 (-6.30, 4.44), p=0.73, I^2^=61% |
| - 3 to 4 only | 5 | 110/103 | MD 0.00 (-0.00, 0.01), p=0.25, I^2^=0% |
| - 3, 4, and 3 to 5 | 3 | 58/61 | MD 0.00 (-1.84, 2.49), p=0.77, I^2^=1% |

**CI** = confidence interval; **I²** =percentage of variation across studies due to heterogeneity; **MD=**mean difference; **N/A**=not applicable; **SMD=**standard mean difference

***** =significant p value of 0.5 or less
