# Supplementary material for: The effect of exercise training in people with pre-dialysis chronic kidney disease: a systematic review with meta-analysis
Source: J Nephrol. 2024 Oct 17;37(8):2063–98. doi: 10.1007/s40620-024-02081-9 (PMC11649798; doi:10.1007/s40620-024-02081-9)
Supplement: Supplementary file 8 — Supplementary file8 (DOCX 47 KB) [file 40620_2024_2081_MOESM8_ESM.docx]

**The effect of exercise training in people with pre-dialysis chronic kidney disease. A systematic review with meta-analysis.**

Annette Traise*, Gudrun Dieberg, Melissa J Pearson, Neil A Smart

Clinical Exercise Physiology, School of Science and Technology, University of New England, NSW 2351, Australia

* Corresponding author

**Online Resource 1**

**Supplemental material: Table 1: Search strategy, Table 2: Excluded studies**

**Supplemental Table 1** Search strategy and results

| **#** | **MEDLINE Query** | **Results 24 August 2023** |
| --- | --- | --- |
| 1 | (Chronic kidney disease or renal disease or CKD).mp. | 117,978 |
| 2 | (exercise or physical activit* or fitness).mp. | 507,508 |
| 3 | 1 and 2 | 2,366 |
| 4 | limit 3 to randomized controlled trial | 240 |
|  | **EMBASE Query** | **Results 24 August 2023** |
| 1 | (chronic AND ('kidney'/exp OR kidney) AND ('disease'/exp OR disease) OR 'renal'/exp OR renal) AND ('disease'/exp OR disease) OR ckd | 1,218,000 |
| 2 | (exercise OR physical) AND activit* OR fitness | 743,423 |
| 3 | #1 AND #2 | 13,634 |
| 4 | #3 AND 'randomized controlled trial'/de | 658 |
| **#** | **Cochrane Library of Controlled Trials Query** | **Results 24 August 2023** |
| 1 | (Chronic kidney disease or renal disease or CKD):ti,ab,kw | 39117 |
| 2 | Exercise or physical activit* or fitness | 169214 |
| 3 | Combine #1 and #2 | 470 |
| 4 | Limit #3 to randomised control trial | 88 |
|  | **CINAHL Query** | **Results 24 August 2023** |
| 1 | (“chronic kidney disease” or “chronic renal failure” or ckd) | 39,240 |
| 2 | (exercise or “physical activity” or fitness) | 33,776 |
| 3 | (“randomized controlled trials” or rtc or “randomised control trials”) | 175,004 |
| 4 | #1 AND #2 AND #3 | 149 |
|  | **SPORTDiscus Query** | **Results 24 August 2023** |
| 1 | (“chronic kidney disease” or “chronic renal failure” or ckd) | 1,332 |
| 2 | (exercise or “physical activity” or fitness) | 413,230 |
| 3 | (“randomized controlled trials” or rtc or “randomised control trials”) | 21,064 |
| 4 | #1 AND #2 AND #3 | 26 |

**Supplemental Table 2** Excluded studies

| **Randomised Control Trial** | **Reason for exclusion** | **Randomised Control Trial** | **Reason for exclusion** |
| --- | --- | --- | --- |
| Abreu, 2017 | No relevant outcome data | Lazarus, 2018 | No relevant outcome data |
| Akiba, 1995 | No relevant outcome data | Lewis, 2015 | Control not CKD |
| Afshar, 2010 | No relevant outcome data | Li, 2012 | No relevant outcome data |
| Afshar, 2011 | No relevant outcome data | Liang, 2016 | Not easily translated |
| Amany, 2014 | Control not CKD | Liang, 2018 | Not easily translated |
| Aydemir, 2020 | Duplicate, Ikizler, 2018 | Liao, 2016 | No relevant outcome data |
| Bagetta, 2018 | No relevant outcome data | Lopes, 2019 | No relevant outcome data |
| Balakrishnan, 2010 | No relevant outcome data | Macdonald, 2007 | No control group |
| Barbosa, 2018 | No relevant outcome data | Makhlough, 2012 | No relevant outcome data |
| Beetham, 2019 | No sedentary control group | Majchrzak, 2007 | No relevant outcome data |
| Beetham, 2022 | Duplicate, Beetham., 2019 | Malfatti, 2010 | Trial less than 2 weeks |
| Bennett, 2013 | No sedentary control group | Manfredini, 2009 | No relevant outcome data |
| Birdee, 2015 | No sedentary control group | Manfredini, 2015 | No relevant outcome data |
| Bohm, 2014 | No sedentary control group | Manfredini, 2017 | No relevant outcome data |
| Bohm, 2017 | Trial less than 2 weeks | Marchesan, 2014 | No relevant outcome data |
| Brown, 2017 | No relevant outcome data | Marinho, 2016 | No relevant outcome data |
| Bruggemann 2017 | No control group | Martin, 2018 | No relevant outcome data |
| Carmack, 1995 | No relevant outcome data | Martin- Alemañy, 2016 | No relevant outcome data |
| Carney, 1987 | No relevant outcome data | Matsufuji, 2015 | No relevant outcome data |
| Castaneda, 2001 | No relevant outcome data | Matsumoto, 2007 | No relevant outcome data |
| Chang, 2010 | No relevant outcome data | Maynard, 2019 | No relevant outcome data |
| Cheema, 2006 | No relevant outcome data | McGregor, 2018 | No relevant outcome data |
| Cheema, 2007 | No relevant outcome data | McMahon, 1999 | No relevant outcome data |
| Cheema, 2011 | No relevant outcome data | Messonnier, 2012 | No relevant outcome data |
| Chen JL, 2010 | No relevant outcome data | Mohseni, 2013 | No relevant outcome data |
| Cooke, 2018 | No relevant outcome data | Molsted, 2004 | No relevant outcome data |
| Cupisti, 2004 | Trial less than 2 weeks | Momeni, 2014 | No relevant outcome data |
| Daniilidis 2004 | No relevant outcome data | Mortazavi, 2013 | No relevant outcome data |
| Dashtidehkordi, 2019 | No relevant outcome data | Motedayen, 2014 | No relevant outcome data |
| de Lima, 2013 | No relevant outcome data | Nylen, 2015 | No control group |
| De Paul, 2002 | No relevant outcome data | Olvera-Soto, 2016 | No relevant outcome data |
| Deligiannis, 1999a | No relevant outcome data | Orcy, 2012 | No control group |
| Deligiannis, 1999b | No relevant outcome data | Ouzouni, 2009 | No relevant outcome data |
| Dias, 2020 | No control group | Painter, 2012 | No relevant outcome data |
| Dobask, 2011 | No relevant outcome data | Paluchamy, 2018 | No relevant outcome data |
| Dong, 2011 | No relevant outcome data | Pandey, 2017 | No relevant outcome data |
| Dong, 2019 | No relevant outcome data | Parsons, 2004 | No relevant outcome data |
| Doyle, 2017 | No control group | Pechter, 2003 | Not easily translated |
| Dungey, 2015 | No relevant outcome data | Pellizaro, 2013 | No relevant outcome data |
| Dziubek, 2016 | No control group | Petraki, 2008 | No relevant outcome data |
| El-Nahas, 2013 | No control group | Pomidori, 2016 | No relevant outcome data |
| Figueiredo, 2012 | No relevant outcome data | Poorsaadet, 2018 | No relevant outcome data |
| Figueiredo, 2018 | No control group | Pupim, 2004 | No relevant outcome data |
| Fitts, 1999 | No relevant outcome data | Pupim, 2007 | No relevant outcome data |
| Flesher, 2011 | No relevant outcome data | Rahimimoghadam, 2019 | No relevant outcome data |
| Frey, 1999 | No relevant outcome data | Razaei, 2014 | No relevant outcome data |
| Frih, 2017 | No relevant outcome data | Reboredo, 2014 | No relevant outcome data |
| Fuhro, 2017 | No control group | Rouchon, 2016 | No relevant outcome data |
| Fuzari, 2018 | No relevant outcome data | Rosa, 2018 | No relevant outcome data |
| Giannaki, 2013 | No relevant outcome data | Santhi, 2018 | No relevant outcome data |
| Goldberg, 1983 | No relevant outcome data | Sarmento, 2017 | No control group |
| Goldberg, 1986 | No relevant outcome data | Segura-Orti, 2009 | No relevant outcome data |
| Gordon, 2012 | Inconsistent data reporting | Sevick, 2018 | No relevant outcome data |
| Gordon, 2014 | No relevant outcome data | Shahgholian, 2012 | No relevant outcome data |
| Groussard, 2015 | No relevant outcome data | Shardong, 2017 | No relevant outcome data |
| Harter, 1985 | Duplicate, Goldberg, 1983 | Small, 2017 | Duplicate, Howden, 2013 |
| Hase, 1985 | Control group not CKD | Soares, 2017 | No relevant outcome data |
| Headley, 2008 | No relevant outcome data | Soliman, 2015 | No relevant outcome data |
| Hellberg, 2017 | No control group | Song, 2012 | No relevant outcome data |
| Hellberg, 2019 | No control group | Suhardjono, 2019 | No relevant outcome data |
| Henrique, 2010 | No relevant outcome data | Svarstad, 2012 | No relevant outcome data |
| Howden, 2012 | No relevant outcome data | Szulinska, 2016 | No control group |
| Hristea, 2016 | No relevant outcome data | Tao, 2015 | No relevant outcome data |
| Jazi, 2012 | No relevant outcome data | Teng, 2013 | No relevant outcome data |
| Jeong, 2018 | No relevant outcome data | Thompson, 2016 | No relevant outcome data |
| Johansen, 2006 | No relevant outcome data | Toussaint, 2008 | No relevant outcome data |
| Kao, 2012 | Duplicate, Chen PY, 2010 | Tsuyuki, 2003 | No relevant outcome data |
| Kaur, 2016 | Trial less than 2 weeks | Uchiyama, 2019 | No relevant outcome data |
| Kirkman, 2014 | No relevant outcome data | Van Vilsteren, 2005 | No relevant outcome data |
| Kharbteng, 2020 | No relevant outcome data | Watson, 2014 | No relevant outcome data |
| Koh, 2010 | No relevant outcome data | Watson, 2017 | No relevant outcome data |
| Kong, 1999 | Trial less than 2 weeks | Watson, 2018 | No control group |
| Konstantinou, 2002 | No relevant outcome data | Watson, 2022 | Control group not CKD |
| Kopple, 2007 | No relevant outcome data | Wilkinson, 2019 | No control group |
| Koufaki, 2002 | No relevant outcome data | Wilund, 2010 | No relevant outcome data |
| Kouidi, 2004 | No sedentary control group | Yurthuran, 2006 | No relevant outcome data |
| Kouidi, 2009 | No relevant outcome data | Zang, 2019 | No control group |
| Kumar, 2020 | No relevant outcome data | Zhao, 2017 | No control group |
|  |  | Zhou, 2020 | No control group |
