# Supplementary material for: The effect of exercise training in people with pre-dialysis chronic kidney disease: a systematic review with meta-analysis
Source: J Nephrol. 2024 Oct 17;37(8):2063–98. doi: 10.1007/s40620-024-02081-9 (PMC11649798; doi:10.1007/s40620-024-02081-9)
Supplement: Supplementary file 9 — Supplementary file9 (DOCX 635 KB) [file 40620_2024_2081_MOESM9_ESM.docx]

**The effect of exercise training in people with pre-dialysis chronic kidney disease. A systematic review with meta-analysis.**

Annette Traise*, Gudrun Dieberg, Melissa J Pearson, Neil A Smart

Clinical Exercise Physiology, School of Science and Technology, University of New England, NSW 2351, Australia

* Corresponding author

**Online Resource 4**

**Supplemental material: Tables 4 and 5** Study quality

**Supplemental Table 4** Cochrane risk of bias tool for randomised controlled trials (Rob2) for Effect of Exercise on pre-dialysis CKD

**Supplemental Table 5** Assessment of study quality and reporting using the Tool for the assEssment of Study qualiTy and reporting in EXercise (TESTEX)

**Supplemental Table 4** Cochrane risk of bias tool for randomised controlled trials (Rob2) for Effect of Exercise on pre-dialysis CKD

| **Study ID** | **Randomisation process** | **Deviations from the intended** | **Missing outcome** | **Measurement of the outcome** | **Selection of the reported result** | **Overall** |
| --- | --- | --- | --- | --- | --- | --- |
| Aoike 2015 [1] |  |  |  |  |  |  |
| Aioke 2018 [2] |  |  |  |  |  |  |
| Barcellos 2018 [3] |  |  |  |  |  |  |
| Baria 2014 [4] |  |  |  |  |  |  |
| Beetham 2018 [5] |  |  |  |  |  |  |
| Castaneda 2004 [6] |  |  |  |  |  |  |
| Chen 2010 [7] |  |  |  |  |  |  |
| Correa 2021 [8] |  |  |  |  |  |  |
| de Araujo 2023 [9] |  |  |  |  |  |  |
| Deus 2022 [10] |  |  |  |  |  |  |
| Eidemak 1997 [11] |  |  |  |  |  |  |
| Gomes 2017 [12] |  |  |  |  |  |  |
| Greenwood 2015 [13] |  |  |  |  |  |  |
| Gregory 2011 [14] |  |  |  |  |  |  |
| Headley 2012 [15] |  |  |  |  |  |  |
| Headley 2014 [16] |  |  |  |  |  |  |
| Headley 2017 [17] |  |  |  |  |  |  |
| Hiraki 2017 [18] |  |  |  |  |  |  |
| Howden 2013 [19] |  |  |  |  |  |  |
| Howden 2015 [20] |  |  |  |  |  |  |
| Huppertz 2020 [21] |  |  |  |  |  |  |
| Ikizler 2018 [22] |  |  |  |  |  |  |
| Kirkman 2019 [23] |  |  |  |  |  |  |
| Kirkman 2021 [24] |  |  |  |  |  |  |
| Leehey 2009 [25] |  |  |  |  |  |  |
| Leehey 2016 [26] |  |  |  |  |  |  |
| Miele 2017 [27] |  |  |  |  |  |  |
| Mustata 2011 [28] |  |  |  |  |  |  |
| Nixon 2021 [29] |  |  |  |  |  |  |
| Otobe 2021 [30] |  |  |  |  |  |  |
| Rossi 2014 [31] |  |  |  |  |  |  |
| Shi 2014 [32] |  |  |  |  |  |  |
| Tang 2017 [33] |  |  |  |  |  |  |
| Thompson 2022 [34] |  |  |  |  |  |  |
| Uchiyama 2021 [35] |  |  |  |  |  |  |
| Van Craenenbroeck 2015 [36] |  |  |  |  |  |  |
| Weiner 2022 [37] |  |  |  |  |  |  |

|  | = Low risk; |  | = Some concerns |
| --- | --- | --- | --- |

**Supplemental Table 5** Assessment of study quality and reporting using the Tool for the assEssment of Study qualiTy and reporting in EXercise (TESTEX)

| Study | Eligibility Criteria specified | Random-isation  Details Specified | Allocation concealed | Groups similar at baseline | Assessors blinded | Outcomes measures assessed >85% participants^#^ | Intention to treat analysis | Reporting between group statistical comparison* | Point measures & measures of variability | Activity monitoring in control group | Relative exercise intensity constant | Exercise volume & Energy expenditure | | Overall TESTEX (/15) |
| --- | --- | --- | --- | --- | --- | --- | --- | --- | --- | --- | --- | --- | --- | --- |
| Aoike (2015) [1] | 1 | 0 | 1 | 1 | 0 | 2 | 0 | 2 | 1 | 0 | 1 | 1 | | 10 |
| Aoike (2018) [2] | 1 | 1 | 1 | 1 | 0 | 2 | 0 | 2 | 1 | 0 | 1 | 1 | | 11 |
| Barcellos (2018) [3] | 1 | 1 | 1 | 1 | 1 | 2 | 1 | 2 | 1 | 0 | 1 | 1 | | 13 |
| Baria (2014) [4] | 1 | 1 | 0 | 1 | 0 | 1 | 0 | 1 | 1 | 1 | 1 | 1 | | 9 |
| Beetham (2018) [5] | 1 | 1 | 1 | 1 | 0 | 2 | 0 | 2 | 1 | 0 | 1 | 1 | | 11 |
| Castaneda (2004) [6] | 1 | 1 | 1 | 1 | 1 | 0 | 0 | 2 | 1 | 1 | 1 | 1 | | 11 |
| Chen (2010) [7] | 0 | 1 | 0 | 1 | 0 | 1 | 0 | 1 | 0 | 0 | 1 | 0 | | 5 |
| Correa (2021) [8] | 1 | 1 | 0 | 1 | 0 | 3 | 0 | 2 | 1 | 0 | 1 | 1 | | 11 |
| de Araujo (2023) [9] | 1 | 1 | 0 | 1 | 0 | 2 | 0 | 2 | 1 | 0 | 1 | 1 | | 10 |
| Deus (2022) [10] | 1 | 1 | 0 | 1 | 0 | 3 | 0 | 2 | 1 | 0 | 1 | 1 | | 11 |
| Eidemak (1997) [11] | 1 | 0 | 0 | 1 | 0 | 1 | 0 | 1 | 0 | 0 | 1 | 1 | | 6 |
| Gomes (2017) [12] | 1 | 1 | 0 | 1 | 0 | 1 | 0 | 2 | 1 | 0 | 1 | 1 | | 9 |
| Greenwood (2015) [13] | 1 | 1 | 1 | 0 | 1 | 2 | 0 | 2 | 1 | 0 | 1 | 1 | | 11 |
| Gregory (2011) [14] | 1 | 0 | 0 | 0 | 0 | 2 | 0 | 1 | 1 | 0 | 1 | 1 | | 7 |
| Headley (2012) [15] | 1 | 0 | 0 | 0 | 0 | 2 | 0 | 1 | 1 | 0 | 1 | 1 | | 7 |
| Headley (2014) [16] | 1 | 0 | 1 | 1 | 0 | 2 | 0 | 2 | 1 | 0 | 1 | 1 | | 10 |
| Headley (2017) [17] | 1 | 0 | 1 | 1 | 0 | 2 | 0 | 2 | 1 | 0 | 1 | 1 | | 10 |
| Hiraki (2017) [18] | 1 | 1 | 0 | 1 | 0 | 2 | 0 | 2 | 1 | 1 | 0 | 1 | | 10 |
| Howden (2013) [19] | 1 | 1 | 1 | 1 | 0 | 2 | 0 | 2 | 1 | 0 | 1 | 1 | | 11 |
| Howden (2015) [20] | 1 | 1 | 1 | 1 | 0 | 2 | 0 | 2 | 1 | 0 | 1 | 1 | | 11 |
| Huppertz (2020) [21] | 1 | 1 | 1 | 1 | 0 | 2 | 0 | 2 | 1 | 0 | 1 | 1 | | 11 |
| Ikizler (2018) [22] | 1 | 1 | 1 | 1 | 0 | 2 | 1 | 2 | 1 | 0 | 1 | 1 | | 12 |
| Kirkman (2019) [23] | 1 | 1 | 0 | 1 | 0 | 2 | 0 | 2 | 1 | 0 | 1 | 1 | | 10 |
| Kirkman (2021) [24] | 1 | 1 | 0 | 1 | 0 | 2 | 0 | 2 | 1 | 0 | 1 | 1 | | 10 |
| Leehey (2009) [25] | 1 | 0 | 1 | 1 | 0 | 0 | 0 | 1 | 1 | 0 | 1 | 1 | | 7 |
| Leehey (2016) [26] | 1 | 1 | 1 | 1 | 1 | 2 | 0 | 2 | 1 | 0 | 1 | 1 | | 12 |
| Miele (2017) [27] | 1 | 0 | 0 | 1 | 0 | 3 | 0 | 2 | 1 | 0 | 1 | 0 | | 9 |
| Mustata (2011) [28] | 1 | 1 | 1 | 1 | 1 | 3 | 1 | 2 | 1 | 0 | 1 | 1 | | 14 |
| Nixon (2021) [29] | 1 | 1 | 1 | 1 | 0 | 2 | 0 | 2 | 1 | 0 | 1 | 1 | | 11 |
| Otobe (2021) [30] | 1 | 1 | 0 | 0 | 1 | 2 | 0 | 2 | 1 | 0 | 1 | 1 | | 10 |
| Rossi (2014) [31] | 1 | 1 | 0 | 0 | 0 | 2 | 0 | 1 | 1 | 0 | 1 | 0 | | 8 |
| Shi (2014) [32] | 1 | 0 | 0 | 1 | 0 | 2 | 0 | 1 | 1 | 0 | 0 | 0 | | 6 |
| Tang (2017) [33] | 1 | 1 | 0 | 1 | 0 | 2 | 0 | 2 | 1 | 0 | 0 | 0 | | 8 |
| Thompson (2022) [34] | 1 | 1 | 1 | 1 | 0 | 3 | 0 | 2 | 1 | 0 | 1 | 1 | | 12 |
| Uchiyama (2021) [35] | 1 | 1 | 1 | 1 | 1 | 3 | 1 | 2 | 1 | 0 | 1 | 1 | | 14 |
| Van Craenenbroeck (2015) [36] | 1 | 1 | 0 | 1 | 1 | 1 | 0 | 2 | 1 | 0 | 1 | 1 | | 10 |
| Weiner (2022) [37] | 1 | 1 | 0 | 1 | 0 | 0 | 0 | 2 | 1 | 0 | 1 | 1 | | 8 |
| Totals out of 37 | 37 | 28 | 18 | 32 | 8 | 34 | 4 | 37 | 35 | 3 | 34 | 32 | Median score = 10 | |

Key: total out of 15 points. Legend: #three points possible—one point if adherence >85%, one point if adverse events reported, one point if exercise attendance is reported.

*Two points possible—one point if primary outcome is reported, one point if all other outcomes reported. 0 awarded if no mention was made of this criterion, or if it was unclear.

**References**

1. Aoike DT, Baria F, Kamimura MA, Ammirati A, de Mello MT, Cuppari L (2015). Impact of home-based aerobic exercise on the physical capacity of overweight patients with chronic kidney disease. Int Urol Nephrol 47:359-367. <https://doi.org/10.1007/s11255-014-0894-8>

2. Aoike DT, Baria F, Kamimura MA, Ammirati A, Cuppari L (2018). Home-based versus center-based aerobic exercise on cardiopulmonary performance, physical function, quality of life and quality of sleep of overweight patients with chronic kidney disease. Clin Exp Nephrol 22:87-98. <https://doi.org/10.1007/s10157-017-1429-2>

3. Barcellos FC, Del Vecchio FB, Reges A, Mielke G, Santos IS, Umpierre D, Bohlke M, Hallal PC (2018). Exercise in patients with hypertension and chronic kidney disease: a randomized controlled trial. J Hum Hypertens 32:397-407. <https://doi.org/10.1038/s41371-018-0055-0>

4. Baria F, Kamimura MA, Aoike DT, Ammirati A, Rocha ML, de Mello MT, Cuppari L (2014). Randomized controlled trial to evaluate the impact of aerobic exercise on visceral fat in overweight chronic kidney disease patients. Nephrol Dial Transplant 29:857-864. <https://doi.org/10.1093/ndt/gft529>

5. Beetham KS, Howden EJ, Isbel NM, Coombes JS (2018). Agreement between cystatin-C and creatinine based eGFR estimates after a 12-month exercise intervention in patients with chronic kidney disease. BMC Nephrol 19:366. <https://doi.org/10.1186/s12882-018-1146-4>

6. Castaneda C, Gordon PL, Parker RC, Uhlin KL, Roubenoff R, Levey AS (2004). Resistance training to reduce the malnutrition-inflammation complex syndrome of chronic kidney disease. Am J Kidney Dis 43:607-616. <https://doi.org/10.1053/j.ajkd.2003.12.025>

7. Chen PY, Huang YC, Kao YH, Chen JY (2010). Effects of an exercise program on blood biochemical values and exercise stage of chronic kidney disease patients. J Nurs Res 18:98-107. <https://doi.org/10.1097/JNR.0b013e3181dda726>

8. Correa HL, Neves RVP, Deus LA, Maia BCH, Maya AT, Tzanno-Martins C, Souza MK, Silva JAB, Haro AS, Costa F, Moraes MR, Simoes HG, Prestes J, Stone W, Rosa TS (2021). Low-load resistance training with blood flow restriction prevent renal function decline: The role of the redox balance, angiotensin 1-7 and vasopressin. Physiol Behav 230:113295. <https://doi.org/10.1016/j.physbeh.2020.113295>

9. de Araujo TB, de Luca Correa H, de Deus LA, Neves RVP, Reis AL, Honorato FS, da SBJM, Palmeira TRC, Aguiar SS, Sousa CV, Santos CAR, Neto LSS, Amorim CEN, Simoes HG, Prestes J, Rosa TS (2023). The effects of home-based progressive resistance training in chronic kidney disease patients. Exp Gerontol 171:112030. <https://doi.org/10.1016/j.exger.2022.112030>

10. Deus LA, Correa HL, Neves RVP, Reis AL, Honorato FS, Araujo TB, Souza MK, Haro AS, Silva VL, Barbosa J, Padula IA, Andrade RV, Simoes HG, Prestes J, Stone WJ, Melo GF, Rosa TS (2022). Metabolic and hormonal responses to chronic blood-flow restricted resistance training in chronic kidney disease: a randomized trial. Appl Physiol Nutr Metab 47:183-194. <https://doi.org/10.1139/apnm-2021-0409>

11. Eidemak I, Haaber AB, Feldt-Rasmussen B, Kanstrup IL, Strandgaard S (1997). Exercise training and the progression of chronic renal failure. Nephron 75:36-40. <https://doi.org/10.1159/000189497>

12. Gomes TS, Aoike DT, Baria F, Graciolli FG, Moyses RMA, Cuppari L (2017). Effect of Aerobic Exercise on Markers of Bone Metabolism of Overweight and Obese Patients With Chronic Kidney Disease. J Ren Nutr 27:364-371. <https://doi.org/10.1053/j.jrn.2017.04.009>

13. Greenwood SA, Koufaki P, Mercer TH, MacLaughlin HL, Rush R, Lindup H, O'Connor E, Jones C, Hendry BM, Macdougall IC, Cairns HS (2015). Effect of exercise training on estimated GFR, vascular health, and cardiorespiratory fitness in patients with CKD: a pilot randomized controlled trial. Am J Kidney Dis 65:425-434. <https://doi.org/10.1053/j.ajkd.2014.07.015>

14. Gregory SM, Headley SA, Germain M, Flyvbjerg A, Frystyk J, Coughlin MA, Milch CM, Sullivan S, Nindl BC (2011). Lack of circulating bioactive and immunoreactive IGF-I changes despite improved fitness in chronic kidney disease patients following 48 weeks of physical training. Growth Horm IGF Res 21:51-56. <https://doi.org/10.1016/j.ghir.2010.12.005>

15. Headley S, Germain M, Milch C, Pescatello L, Coughlin MA, Nindl BC, Cornelius A, Sullivan S, Gregory S, Wood R (2012). Exercise training improves HR responses and V O2peak in predialysis kidney patients. Med Sci Sports Exerc 44:2392-2399. <https://doi.org/10.1249/MSS.0b013e318268c70c>

16. Headley S, Germain M, Wood R, Joubert J, Milch C, Evans E, Poindexter A, Cornelius A, Brewer B, Pescatello LS, Parker B (2014). Short-term aerobic exercise and vascular function in CKD stage 3: a randomized controlled trial. Am J Kidney Dis 64:222-229. <https://doi.org/10.1053/j.ajkd.2014.02.022>

17. Headley S, Germain M, Wood R, Joubert J, Milch C, Evans E, Cornelius A, Brewer B, Taylor B, Pescatello LS (2017). Blood pressure response to acute and chronic exercise in chronic kidney disease. Nephrology (Carlton) 22:72-78. <https://doi.org/10.1111/nep.12730>

18. Hiraki K, Shibagaki Y, Izawa KP, Hotta C, Wakamiya A, Sakurada T, Yasuda T, Kimura K (2017). Effects of home-based exercise on pre-dialysis chronic kidney disease patients: a randomized pilot and feasibility trial. BMC Nephrol 18:198. <https://doi.org/10.1186/s12882-017-0613-7>

19. Howden EJ, Leano R, Petchey W, Coombes JS, Isbel NM, Marwick TH (2013). Effects of exercise and lifestyle intervention on cardiovascular function in CKD. Clin J Am Soc Nephrol 8:1494-1501. <https://doi.org/10.2215/CJN.10141012>

20. Howden EJ, Coombes JS, Strand H, Douglas B, Campbell KL, Isbel NM (2015). Exercise training in CKD: efficacy, adherence, and safety. Am J Kidney Dis 65:583-591. <https://doi.org/10.1053/j.ajkd.2014.09.017>

21. Huppertz N, Beetham KS, Howden EJ, Leicht AS, Isbel NM, Coombes JS (2020). A 12-month lifestyle intervention does not improve cardiac autonomic function in patients with chronic kidney disease. Auton Neurosci 224:102642. <https://doi.org/10.1016/j.autneu.2020.102642>

22. Ikizler TA, Robinson-Cohen C, Ellis C, Headley SAE, Tuttle K, Wood RJ, Evans EE, Milch CM, Moody KA, Germain M, Limkunakul C, Bian A, Stewart TG, Himmelfarb J (2018). Metabolic Effects of Diet and Exercise in Patients with Moderate to Severe CKD: A Randomized Clinical Trial. J Am Soc Nephrol 29:250-259. <https://doi.org/10.1681/ASN.2017010020>

23. Kirkman DL, Ramick MG, Muth BJ, Stock JM, Pohlig RT, Townsend RR, Edwards DG (2019). Effects of aerobic exercise on vascular function in nondialysis chronic kidney disease: a randomized controlled trial. Am J Physiol Renal Physiol 316:F898-F905. <https://doi.org/10.1152/ajprenal.00539.2018>

24. Kirkman DL, Ramick MG, Muth BJ, Stock JM, Townsend RR, Edwards DG (2021). A randomized trial of aerobic exercise in chronic kidney disease: Evidence for blunted cardiopulmonary adaptations. Ann Phys Rehabil Med 64:101469. <https://doi.org/10.1016/j.rehab.2020.101469>

25. Leehey DJ, Moinuddin I, Bast JP, Qureshi S, Jelinek CS, Cooper C, Edwards LC, Smith BM, Collins EG (2009). Aerobic exercise in obese diabetic patients with chronic kidney disease: a randomized and controlled pilot study. Cardiovasc Diabetol 8:62. <https://doi.org/10.1186/1475-2840-8-62>

26. Leehey DJ, Collins E, Kramer HJ, Cooper C, Butler J, McBurney C, Jelinek C, Reda D, Edwards L, Garabedian A, O''Connell S (2016). Structured Exercise in Obese Diabetic Patients with Chronic Kidney Disease: A Randomized Controlled Trial. Am J Nephrol 44:54-62. <https://doi.org/10.1159/000447703>

27. Miele EM, Headley SAE, Germain M, Joubert J, Herrick S, Milch C, Evans E, Cornelius A, Brewer B, Taylor B, Wood RJ (2017). High-density lipoprotein particle pattern and overall lipid responses to a short-term moderate-intensity aerobic exercise training intervention in patients with chronic kidney disease. Clin Kidney J 10:524-531. <https://doi.org/10.1093/ckj/sfx006>

28. Mustata S, Groeneveld S, Davidson W, Ford G, Kiland K, Manns B (2011). Effects of exercise training on physical impairment, arterial stiffness and health-related quality of life in patients with chronic kidney disease: a pilot study. Int Urol Nephrol 43:1133-1141. <https://doi.org/10.1007/s11255-010-9823-7>

29. Nixon AC, Bampouras TM, Gooch HJ, Young HML, Finlayson KW, Pendleton N, Mitra S, Brady ME, Dhaygude AP (2021). Home-based exercise for people living with frailty and chronic kidney disease: A mixed-methods pilot randomised controlled trial. PLoS One 16:e0251652. <https://doi.org/10.1371/journal.pone.0251652>

30. Otobe Y, Yamada M, Hiraki K, Onari S, Taki Y, Sumi H, Hachisuka R, Han W, Takahashi M, Suzuki M, Kimura Y, Koyama S, Masuda H, Shibagaki Y, Tominaga N (2021). Physical Exercise Improves Cognitive Function in Older Adults with Stage 3-4 Chronic Kidney Disease: A Randomized Controlled Trial. Am J Nephrol 52:929-939. <https://doi.org/10.1159/000520230>

31. Rossi AP, Burris DD, Lucas FL, Crocker GA, Wasserman JC (2014). Effects of a renal rehabilitation exercise program in patients with CKD: a randomized, controlled trial. Clin J Am Soc Nephrol 9:2052-2058. <https://doi.org/10.2215/CJN.11791113>

32. Shi ZM, Wen HP, Liu FR, Yao CX (2014). The effects of tai chi on the renal and cardiac functions of patients with chronic kidney and cardiovascular diseases. J Phys Ther Sci 26:1733-1736. <https://doi.org/10.1589/jpts.26.1733>

33. Tang Q, Yang B, Fan F, Li P, Yang L, Guo Y (2017). Effects of individualized exercise program on physical function, psychological dimensions, and health-related quality of life in patients with chronic kidney disease: A randomized controlled trial in China. Int J Nurs Pract 23:1-8. <https://doi.org/10.1111/ijn.12519>

34. Thompson S, Wiebe N, Stickland MK, Gyenes GT, Davies R, Vallance J, Graham M (2022). Physical Activity in Renal Disease and the Effect on Hypertension: A Randomized Controlled Trial. Kidney Blood Press Res 47:475-485. <https://doi.org/10.1159/000524518>

35. Uchiyama K, Adachi K, Muraoka K, Nakayama T, Oshida T, Yasuda M, Hishikawa A, Minakuchi H, Miyashita K, Tokuyama H, Wakino S, Itoh H (2021). Home-based aerobic exercise and resistance training for severe chronic kidney disease: a randomized controlled trial. J Cachexia Sarcopenia Muscle 12:1789-1802. <https://doi.org/10.1002/jcsm.12775>

36. Van Craenenbroeck AH, Van Craenenbroeck EM, Van Ackeren K, Vrints CJ, Conraads VM, Verpooten GA, Kouidi E, Couttenye MM (2015). Effect of Moderate Aerobic Exercise Training on Endothelial Function and Arterial Stiffness in CKD Stages 3-4: A Randomized Controlled Trial. Am J Kidney Dis 66:285-296. <https://doi.org/10.1053/j.ajkd.2015.03.015>

37. Weiner DE, Liu CK, Miao S, Fielding R, Katzel LI, Giffuni J, Well A, Seliger SL (2023). Effect of Long-term Exercise Training on Physical Performance and Cardiorespiratory Function in Adults With CKD: A Randomized Controlled Trial. Am J Kidney Dis 81:59-66. <https://doi.org/10.1053/j.ajkd.2022.06.008>
